# Combined transcriptomic and proteomic analyses define conserved host signatures during innate immune perturbations in DF-1 chicken fibroblasts

**DOI:** 10.3389/fcimb.2026.1813484

**Published:** 2026-05-29

**Authors:** Frederic Sorgeloos, Efstathios S. Giotis, Kate Heesom, Michael A. Skinner, Ian G. Goodfellow

**Affiliations:** 1 Division of Virology, Department of Pathology, University of Cambridge, Addenbrooke’s Hospital, Cambridge, United Kingdom; 2 de Duve Institute, Université catholique de Louvain, Brussels, Belgium; 3 Institut National de la Recherche Scientifique, Centre Armand-Frappier Santé Biotechnologie, Laval, QC, Canada; 4 Department of Infectious Disease, Section of Virology, Imperial College London, London, United Kingdom; 5 Department of Medicine, Section of Virology, Imperial College London, London, United Kingdom; 6 School of Life Sciences, University of Essex, Colchester, United Kingdom; 7 Proteomics facility, School of Biochemistry, University of Bristol, Bristol, United Kingdom

**Keywords:** DF-1 chicken fibroblasts, dsRNA, innate immunity, interferon (IFN), interferon-stimulated genes (ISGs), RNA-Seq, SILAC-based quantitative proteomics, transcription-translation correlation

## Abstract

The interferon system, first discovered in chicken embryonated eggs, plays a pivotal role in defending vertebrates against pathogens, yet comprehensive multi-omics analyses of immune responses in avian species remain scarce. In this study, we employed an integrative approach combining RNA-Seq transcriptomics with SILAC-based quantitative proteomics to characterize global changes in gene and protein expression following immune stimulation in DF-1 chicken fibroblasts. Cells were treated with three distinct immune stimuli: chicken type I interferon, the synthetic double-stranded RNA (dsRNA) analog poly(I:C), and Infectious Bursal Disease Virus (IBDV), a birnavirus with a bi-segmented dsRNA genome. Our analysis revealed a core set of over 110 genes consistently upregulated across all conditions, representing conserved components of the antiviral response. Each stimulus also triggered distinct transcriptional programs, with poly(I:C) eliciting the most robust cytokine induction. At the proteomic level, we quantified more than 2,400 proteins, uncovering asymmetric regulatory patterns in which upregulated proteins exhibited greater fold changes than those that were downregulated. Notably, analysis of transcription-translation coupling revealed a moderate overall correlation between mRNA and protein abundance, which was significantly attenuated following poly(I:C) stimulation, likely reflecting PKR-mediated translational arrest. Ontological analysis corroborated the upregulation of interferon-stimulated genes and proteins involved in inhibiting viral genome replication, as expected. Intriguingly, proteins associated with mitochondrial metabolism and protein synthesis were consistently downregulated, suggesting a coordinated suppression of cellular bioenergetic and translational functions during the antiviral response. These findings underscore the critical role of post-transcriptional regulation in shaping the innate immune landscape, as numerous proteins exhibited altered expression without corresponding mRNA changes. Collectively, this study provides a comprehensive resource for dissecting cellular avian immune signaling and demonstrates the power of integrative multi-omics approaches in uncovering the complex regulatory networks governing host defense.

## Introduction

Like mammals, chickens possess a robust innate immune system that serves as the first line of defense against viral infections (Goossens et al., 2013). Central to this defense are interferons (IFNs), a group of cytokines classified into three types: Type I, II, and III, based on sequence homology and receptor specificity (Negishi et al., 2018; Mesev et al., 2019). IFNs play a critical role in both innate and adaptive immunity by inducing the expression of numerous interferon-stimulated genes (ISGs), which mediate antiviral responses, activate antigen-presenting cells, promote T and B lymphocyte differentiation, and enhance antibody production (Schoggins et al., 2011; Barrat et al., 2019). Notably, chicken IFN-α, first identified for its ability to inhibit influenza virus replication, was also the first IFN ever discovered (Isaacs and Lindenmann, 1957; Isaacs et al., 1957). Despite this early milestone, research on chicken IFNs has lagged behind that of mammals, particularly in elucidating their antiviral mechanisms and immune regulatory roles (Goossens et al., 2013; Giotis et al., 2016; Santhakumar et al., 2017).

In chickens, Type I IFNs include IFN-α and IFN-β, while Type II (IFN-γ) and Type III (IFN-λ) are each represented by a single gene (Masuda et al., 2012; Goossens et al., 2013; Reuter et al., 2014). IFNs themselves do not directly neutralize viruses but instead activate signaling pathways such as the JAK-STAT cascade, leading to ISG transcription. These ISGs are crucial for controlling viral infections through various immune mechanisms, including viral replication inhibition, immune modulation, and enhanced antigen presentation (Silvennoinen et al., 1993; Qu et al., 2013; Anjum et al., 2020). While studies have attempted to catalogue chicken ISGs using microarrays, RNA sequencing (RNA-Seq), and qPCR (Giotis et al., 2016; Giotis et al., 2017; Röll et al., 2017; Giotis et al., 2019; Santhakumar et al., 2019; Dai et al., 2020; Yang et al., 2020), their response remains less understood compared to their human and mouse counterparts. As in mammals, ISG profiles in chickens vary based on cell type, stimulation duration, and methodological approaches (Röll et al., 2017), making it challenging to fully assess their role in how chickens combat zoonotic influenza viruses and other avian pathogens (Masuda et al., 2012; Shah et al., 2019; Yang et al., 2020; Layton et al., 2022; Lai et al., 2024; Wang et al., 2024).

High-throughput technologies such as RNA-Seq have significantly advanced our understanding of ISG regulation during immune responses in both chickens and non-model organisms. However, while RNA-Seq provides insights into transcriptomic changes, quantitative proteomics, widely used in mammalian studies, directly measures protein abundance, offering a clearer picture of ISG functionality at the protein level. This distinction is critical, as proteins, rather than mRNA, execute antiviral defense mechanisms. Post-transcriptional regulation and protein turnover can lead to discrepancies between mRNA and protein expression, highlighting the importance of integrating transcriptomic and proteomic data for a comprehensive understanding of ISG responses. Despite these advancements, substantial gaps remain in understanding how chicken ISG transcriptomes translate into functional proteomes during innate immune activation. While some chicken ISGs have been characterized, many remain poorly understood, and novel ISGs continue to be identified. Additionally, recent research suggests that non-coding RNAs, microRNAs, and long non-coding RNAs (lncRNAs) may modulate ISG responses, but their roles in chickens remain largely unexplored. This underscores the need for integrative approaches to fully map ISG regulatory networks in avian immunity.

In this study, we aimed to address these knowledge gaps by conducting a comprehensive analysis of the transcriptomic and proteomic responses in chicken DF-1 fibroblasts following stimulation with Infectious Bursal Disease Virus (IBDV), Type I IFN, and polyinosinic:polycytidylic acid (poly(I:C)). By integrating RNA-Seq and SILAC-based quantitative proteomics, we sought to identify cell-specific conserved ISGs signatures and immune-related genes, examine the relationship between mRNA and protein abundances, and explore regulatory processes governing the fibroblast-mediated antiviral defense. This study provides new insights into the molecular mechanisms underlying the cellular innate immune response and highlights critical differences between transcriptomic and proteomic landscapes, offering a more comprehensive understanding of avian cellular innate immunity.

## Materials and methods

### Cell culture and SILAC media

DF-1 (ATCC CRL-12203) cells and primary chicken embryonic fibroblasts (The Pirbright Institute, Pirbright, UK) were maintained in DMEM supplemented with 10% fetal calf serum, 100 U/ml penicillin, 100 ug/ml streptomycin, and 1% Non-Essential Amino Acids (PAA). For SILAC labelling, DF-1 cells were cultured in DMEM (Dundee Cell Products, UK) without L-arginine and L-lysine supplemented with 10% fetal calf serum dialyzed against a 10, 000-molecular-weight cut-off membrane, 100 U/ml penicillin, 100 ug/ml streptomycin, 1% Non-Essential Amino Acids (lacking arginine and lysine) and Light (R0K0), Medium (R6K4) or Heavy (R10K8) L-arginine and L-lysine (Cambridge Isotopes Laboratories, USA). All cells were maintained at 37 °C with 5% CO2.

### Chicken IFN-α and viruses

Recombinant chicken IFN-α was prepared as previously reported (Buttigieg et al., 2013). Briefly, a plasmid encoding the chicken IFN-α was transfected into HEK293T cells and the supernatant was collected after 48 h of incubation. Cleared supernatant was titrated by 50% plaque reduction assay on CEFs using Semliki Forest virus (SFV) strain A7 and was used at a final concentration of 1000 U/ml. For poly(I:C) stimulation, cells were transfected with poly(I:C) at a concentration of 4.25 µg/mL using PolyFect Transfection Reagent (QIAGEN, Germany) according to the manufacturer’s instructions. In brief, poly(I:C) was diluted in serum-free SILAC-matched medium, and PolyFect was added at a ratio of 2 µL per 1 µg of poly(I:C). The mixture was incubated for 15 min at room temperature to allow complex formation. Subsequently, the complexes were added to the cells in SILAC culture medium containing serum and antibiotics. Cells were incubated under standard conditions for 6 h before further analysis. For virus infection studies, we used the attenuated IBDV vaccine strain PBG98 (Brown and Skinner, 1996), a strong inducer of innate immune response (Aricibasi et al., 2010). IBDV was propagated and titrated by classical plaque assay using freshly isolated CEFs.

### RNA extraction and RT-qPCR

Total cellular RNA from DF-1 cells was extracted using the RNeasy Mini Kit (Qiagen) following the manufacturer’s protocol. RNA integrity was analyzed using RNA Nano 6000 microfluidics chips run on a 2100 Bioanalyzer (Agilent Technologies) resulting in RNA integrity numbers (RIN) ranging from 8.4 to 10. For reverse transcription, 1 ug of DNase I-treated RNA was reverse transcribed using the QuantiTect Reverse Transcription Kit (QIAGEN) for 15 min at 42 °C and stored at -20 °C until further use. Real-time quantitative PCR was performed in a total volume of 25 μL containing 12.5 μL of 2x MESA Blue qPCR MasterMix Plus for SYBR Assay (Eurogentec), 0.2 mM of each primer, and 5 μL of cDNA (diluted 10-fold). After assembly, plates were sealed with optical tape and centrifuged at 3,000 x g for 30 sec to remove any potential bubbles and to collect the PCR reactions in the bottom of the wells. Data were collected using a ViiA7 Real-Time PCR System (Applied Biosystems) with the following cycling parameters: 1 cycle of 50 °C for 120s and 95 °C for 600s, then 40 cycles of 95 °C for 15s and 60 °C for 60s. At the end of amplification, a melting curve analysis was performed starting form 60 °C to 95 °C with increments of 0.05 °C every second. Relative quantification of gene expression was performed using the ΔCt method (Livak and Schmittgen, 2001) with GAPDH (glyceraldehyde-3-phosphate dehydrogenase) as the reference gene for cDNA quantification. Quantitative RT-PCR assays were performed in accordance with MIQE (Minimum Information for Publication of Quantitative Real-Time PCR Experiments) guidelines (Bustin et al., 2009), and detailed information on primer sequences, amplification efficiencies, and validation data are provided in **Supplementary File 1**.

#### RNA-seq analysis

Library preparation and sequencing were conducted by the Beijing Genomics Institute (BGI, Shenzhen, China). Briefly, poly-adenylated RNA was purified using oligo-dT conjugated beads and cDNA libraries were prepared using the TruSeq RNA Sample Prep Kit (v2) (Illumina Inc. San Diego, USA). cDNA libraries were then combined and sequenced from both ends (100 bp) using Illumina HiSeq 4000 Sequencer (Illumina Inc.). To reduce sequencing variably and to obtain a high sequencing depth, each library was sequenced twice using independent lanes.

#### Transcript abundance quantification and differential expression analysis

Raw RNA-Seq reads were inspected for quality using FastQC. Adapters and low-quality sequences were removed with Trimmomatic (Bolger et al., 2014) version 0.33 (ILLUMINACLIP: TruSeq3- PE:2:30:10 LEADING:3 TRAILING:3 SLIDINGWINDOW:4:15 MINLEN:36). Transcript quantification was performed using kallisto (Bray et al., 2016) against the latest chicken transcriptome (bGalGal1.mat.broiler.GRCg7b, GCF_016699485.2). Genes with an average expression of less than 1 transcript per million under both control and treated conditions were excluded from downstream analysis. Differential expression was evaluated using the negative binomial distribution model within edgeR (Robinson et al., 2010). To ensure the robustness of the identified gene sets, the analysis was cross-validated using the DESeq2 package. Library sizes were normalized using the Trimmed Mean of M-values (TMM) method. *P*-values were corrected for multiple testing using the Benjamini–Hochberg false discovery rate (FDR) method (Benjamini and Hochberg, 1995). Genes were considered differentially expressed if the adjusted *p*-value (FDR) was < 0.01 and the absolute log_2_ fold change was ≥ 1. Re-analyzed RNA-Seq datasets from Giotis et al. and Shaw et al. are available under accession numbers PRJEB7620 and PRJEB21332, respectively.

### Western-blot analysis

Cell lysates were prepared in radioimmunoprecipitation assay (RIPA) buffer (150 mM NaCl, 0.5% sodium deoxycholate, 0.1% SDS, 1 mM EDTA, 1% Triton X-100, and 50 mM Tris, pH 8) supplemented with protease and phosphatase inhibitors (Calbiochem; Cat. No. 539134 and 524625). Protein concentrations were determined by bicinchoninic acid (BCA) assay (ThermoFisher Scientific). Equal amounts of total proteins were resolved by SDS-PAGE and transferred to nitrocellulose membranes. Non-specific binding was blocked by incubating membranes in 5% non-fat dried milk in Phosphate Buffered Saline with Tween-20 (PBST) for 1 h at 4 °C. Primary antibodies were diluted in blocking buffer and incubated overnight at 4 °C with gentle rocking. Membranes were then washed three times in PBST for 5 min at room temperature. Species-matched HRP-linked antibodies were diluted in blocking buffer as before and incubated for 1h at room temperature. After three additional washes in PBST, membranes were developed using an enhanced chemiluminescence (ECL) substrate and detected by autoradiography. Antibodies used were: IBDV anti-VP3, characterized in (Phenix et al., 2001) (clone BA11, mouse monoclonal, 1:250), anti-β-actin (clone AC-40, mouse monoclonal, 1:2, 000, Sigma Cat No: A4700), STAT1 (rabbit polyclonal, 1:1000, Cell Signaling Technology, Cat #9172), eIF2α (mouse monoclonal, clone L57A5, 1:1000, Cell Signaling Technology, Cat #2103), anti-phospho eIF2α (rabbit monoclonal, clone D9G8, 1:1000, Cell Signaling Technology, Cat #3398), goat anti-mouse IgG HRP-conjugated (1:5,000, Sigma Cat No A4416-1ML), and goat anti-rabbit IgG HRP-conjugated (1:5, 000, Sigma Cat No A0545-1ML).

### SILAC sample preparation

Cells were harvested at indicated time points, washed three times with ice-cold PBS, and lysed in radioimmunoprecipitation assay (RIPA) buffer (150 mM NaCl, 0.5% sodium deoxycholate, 0.1% SDS, 1 mM EDTA, 1% Triton X-100, and 50 mM Tris, pH 8), supplemented with protease and phosphatase inhibitors (Calbiochem; Cat. No. 539134 and 524625). Lysates were incubated on ice for 30 min with occasional vortexing and clarified by centrifugation at 15,000 × g for 15 min at 4 °C. Protein concentrations were determined using the bicinchoninic acid (BCA) assay (ThermoFisher Scientific), and equal amounts of light- medium- and heavy-labelled proteins were mixed in a 1:1:1 ratio.

### LC-MS-MS analysis

Gel lanes were cut into 10 slices and each slice subjected to in-gel tryptic digestion using a DigestPro automated digestion unit (Intavis Ltd.). The resulting peptides were fractionated using an Ultimate 3000 nanoHPLC system in line with an LTQ-Orbitrap Velos mass spectrometer (Thermo Scientific). In brief, peptides in 1% (vol/vol) formic acid were injected onto an Acclaim PepMap C18 nano-trap column (Thermo Scientific). After washing with 0.5% (vol/vol) acetonitrile 0.1% (vol/vol) formic acid peptides were resolved on a 250 mm × 75 μm Acclaim PepMap C18 reverse phase analytical column (Thermo Scientific) over a 150 min organic gradient, using 7 gradient segments (1-6% solvent B over 1min., 6-15% B over 58min, 15-32% B over 58min, 32-40% B over 5min, 40-90% B over 1min, held at 90% B for 6min and then reduced to 1%B over 1min) with a flow rate of 300 nl min^−1^. Solvent A was 0.1% formic acid and Solvent B was aqueous 80% acetonitrile in 0.1% formic acid. Peptides were ionized by nano-electrospray ionization at 2.1 kV using a stainless-steel emitter with an internal diameter of 30 μm (Thermo Scientific) and a capillary temperature of 250°C. Tandem mass spectra were acquired using an LTQ-Orbitrap Velos mass spectrometer controlled by Xcalibur 2.1 software (Thermo Scientific) and operated in data-dependent acquisition mode. The Orbitrap was set to analyze the survey scans at 60,000 resolution (at m/z 400) in the mass range m/z 300 to 2000 and the top ten multiply charged ions in each duty cycle selected for MS/MS in the LTQ linear ion trap. Charge state filtering, where unassigned precursor ions were not selected for fragmentation, and dynamic exclusion (repeat count, 1; repeat duration, 30s; exclusion list size, 500) were used. Fragmentation conditions in the LTQ were as follows: normalized collision energy, 40%; activation q, 0.25; activation time 10ms; and minimum ion selection intensity, 500 counts.

### Mass spectrometry data analysis

Raw mass spectrometry data files were analyzed using the MaxQuant software (version 2.6.5.0) (Tyanova et al., 2016). Spectra were searched against predicted proteins inferred from the chicken bGalGal1.mat.broiler.GRCg7b (GCF_016699485.2) reference proteome, supplemented with IBDV protein sequences, as well as a contaminant database provided in MaxQuant. The following parameters were used for data analysis: Triple SILAC labels were defined as R0K0, R6K4 and R10K8. Enzyme specificity was set to trypsin/P and a maximum of two missed tryptic cleavages were allowed. Minimum peptide length was set to 7 and protein quantification ratio count was set to 2. Oxidation of methionine and N-terminal acetylation were selected as variable modifications and carbamidomethylation of cysteine was selected as fixed modification. Precursor mass error tolerance and fragment mass error tolerance were set to 4.5 ppm and 0.5 Da, respectively. Maximum false discovery rates were set to 1% at peptide and protein levels. The label-free quantification iBAQ option was enabled.

### Data sharing

The raw sequencing data associated with this study have been submitted to the NCBI Short Read Archive (SRA) (https://www.ncbi.nlm.nih.gov/sra) under the accession number PRJNA418693. The mass spectrometry proteomics data have been deposited to the ProteomeXchange Consortium (https://www.proteomexchange.org) via the PRIDE partner repository (Vizcaíno et al., 2013; Deutsch et al., 2023) with the dataset identifier PXD057686.

## Results

### Experimental design (outline and validation of the experimental approach)

**Figure 1:**
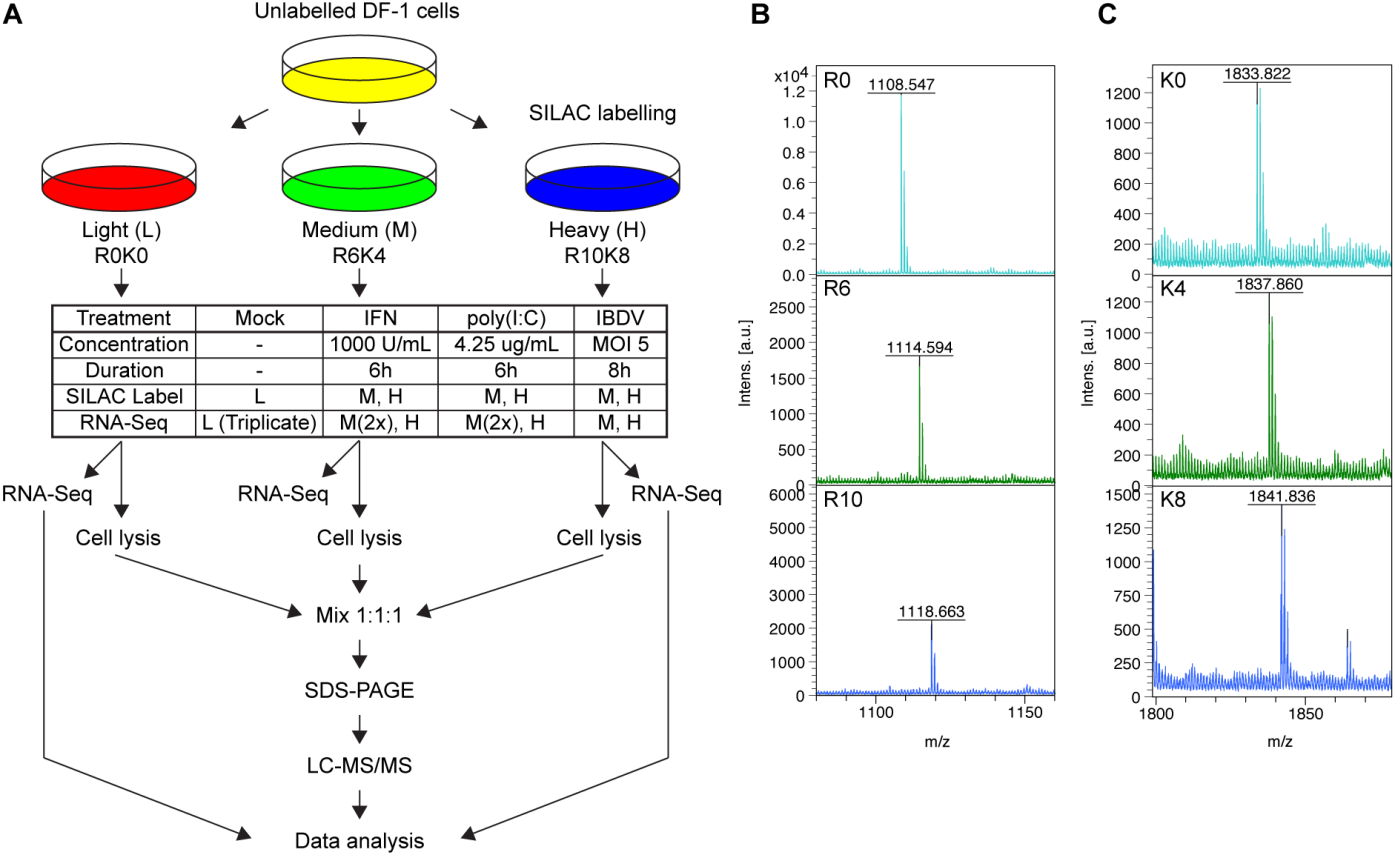
Schematic representation of the combined transcriptomic and SILAC-based quantitative proteomics experiments, including validation of SILAC labelling. **(A)** Experimental design and sample workflow of the combined transcriptomic and proteomic analyses. Stimuli, duration and replicate distribution are indicated for each condition. DF-1 cells cultured into light (R0K0), medium (R6K4) or heavy (R10K8) amino acid-containing media were harvested after 6 passages. Protein lysates were resolved by SDS-PAGE and LC-MS/MS analysis was performed on tryptic peptides eluted from three separate bands of the same apparent molecular weight. **(B, C)** Examples of SILAC-labelled peptides. An arginine-labelled peptide **(B)** shows a mass difference of 6 Da and 10 Da between light and medium or heavy labels, respectively, while a lysine-labelled peptide **(C)** shows a mass difference of 4 Da and 8 Da between light and medium or heavy labels. Near-complete incorporation of labelled amino acids is indicated by the absence of a peak at the m/z position corresponding to the light isotope.

To analyze changes in the chicken transcriptome and proteome following innate immune stimulations, we adopted a genome-wide approach based on RNA-Seq transcriptome profiling coupled with SILAC based quantitative proteomics (**Figure 1A**). We selected DF-1 chicken fibroblasts, a spontaneously immortalized cell line derived from chicken embryo fibroblasts (CEFs), as they have been widely used as a reliable model and have gradually become the standard avian cell substrate for virological and immunological studies (Himly et al., 1998; Maas et al., 2006; Barber et al., 2010; Tan et al., 2016). To this end, chicken DF-1 fibroblasts were cultured with SILAC media to incorporate amino acids for light (Lys0; Arg0), medium (Lys4; Arg6), and heavy (Lys8; Arg10) protein labelling achieving a near, if not complete, incorporation of labelled amino acids (**Figures 1B, C**).

To reflect representative host innate immune responses, labelled cells were stimulated with three innate immune inducers. The Infectious Bursal Disease Virus (IBDV), a double-stranded bi-segmented RNA virus from the *Birnaviridae* family, is an attenuated vaccine strain that induces high levels of innate immune responses and was used in this study as an example of a highly immunogenic RNA virus (Giotis et al., 2019; Dulwich et al., 2020). To avoid bias that might arise from virally-encoded innate immune antagonists, cells were also stimulated with chicken type I interferon and poly(I:C), a synthetic dsRNA analogue acting as a pathogen-associated molecular pattern produced in response to viral infection (Stollar and Stollar, 1970; Weber et al., 2006; Son et al., 2015). Short kinetics was applied to reflect direct intracellular response to stimulation and in the case of viral infections to avoid cell lysis that might modify protein contents upon synchronized infection at high multiplicity. The specific stimulation durations (6h for IFN and poly(I:C); 8h for IBDV) were selected based on the peak kinetics of avian ISG induction and the IBDV replication cycle (Giotis et al., 2016; Shaw et al., 2017; Niu et al., 2024), capturing robust antiviral signatures while remaining early enough to avoid confounding effects from viral-mediated host shutoff or cytopathic effect. After indicated incubation periods, cells were collected and proceeded to concomitantly isolate total cellular RNA and proteins as outlined in **Figure 1A**. For each condition two and three biological replicates were performed for proteomics and transcriptomics studies, respectively.

**Figure 2:**
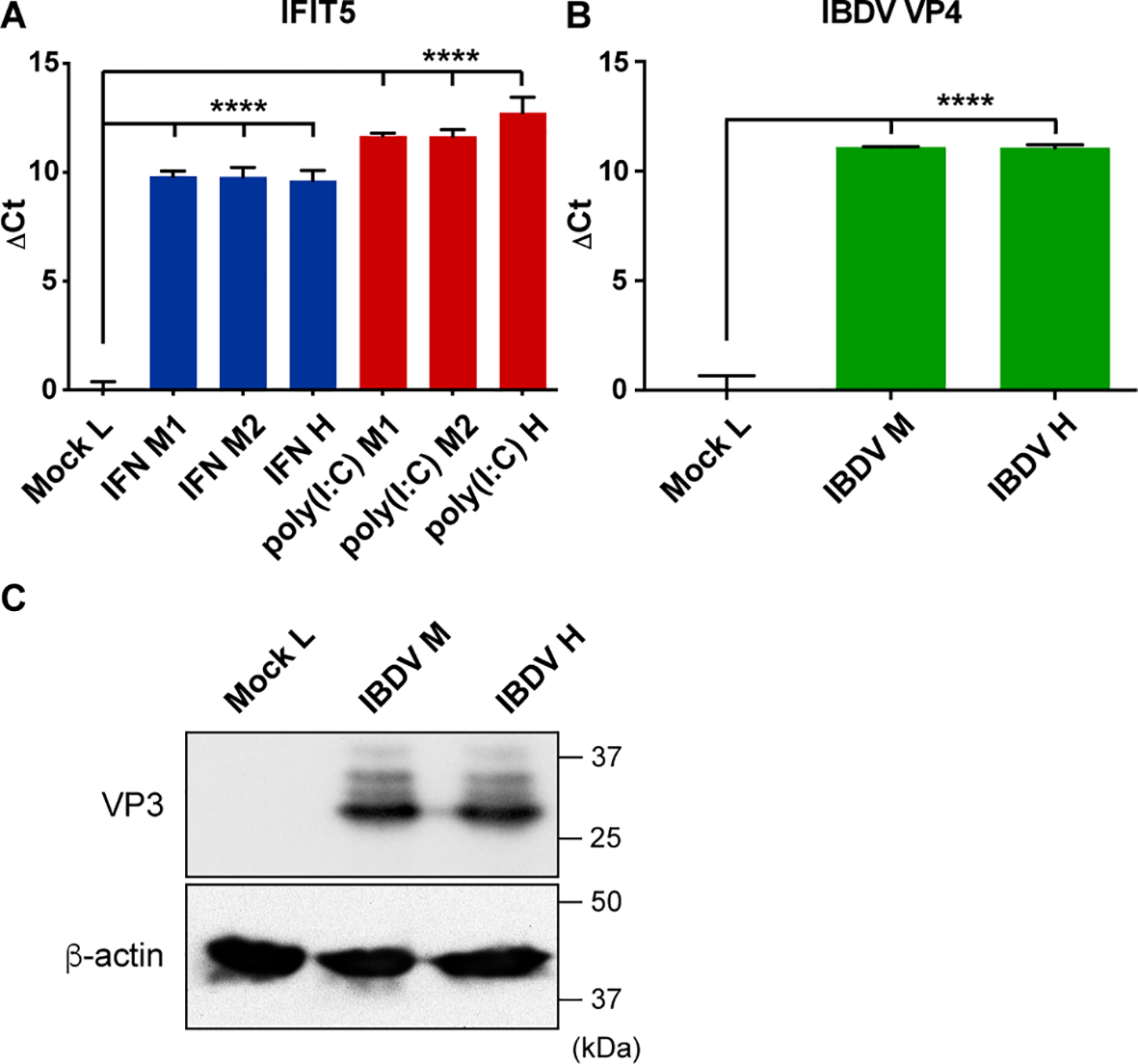
RT-qPCR and western blot analysis of stimulation outcomes in SILAC-labelled DF-1 cells. **(A)** Quantitative expression analysis of IFIT5 mRNA following IFN treatment or poly(I:C) transfection of SILAC-labelled DF-1 cells. IFIT5 mRNA levels were measured by real-time PCR and normalized to GAPDH mRNA levels for each sample. Bar graphs show relative cycle-threshold differences (∆Ct) between treated and mock-treated samples. Error bars represent the standard deviation from one experiment performed in technical triplicates. Statistical analysis was conducted on ΔCt values from each condition using two-tailed unpaired Student’s t-tests relative to the mock condition. **(B)** IBDV VP4 viral RNA levels detected and analyzed as in **(A)**. **(C)** DF-1 cells labelled as above were infected with IBDV at a MOI of 5 or left uninfected (Mock L). After 8 hours, total protein extracts were prepared, and Western blotting was performed using an anti-IBDV VP3 monoclonal antibody. β-actin was used as a loading control. Statistical significance was defined as follows: **** : p < 0.0001.

Successful treatment of the SILAC-labelled cells was assessed by RT-qPCR analysis. Treating cells with IFN or transfecting poly(I:C) led to robust transcription of IFIT5 mRNA, a well characterized Interferon-stimulated gene (Barber et al., 2013; Santhakumar et al., 2018). Although the levels of IFIT5 induction after IFN treatment were lower than those observed following poly(I:C) treatment, a clear response was observed after direct stimulations of interferon induction and receptor signaling pathways (**Figure 2A**). Similarly, a significant increase in cycle-threshold differences for viral mRNA expression between mock and IBDV-infected cells was measured as well as the translation of the VP3 capsid protein (**Figures 2B, C**). Taken together, these results indicate that both direct stimulations of the innate immune system and viral infection were effective.

### Transcriptomic responses to innate immune stimulations in immortalized chicken fibroblasts

**Figure 3:**
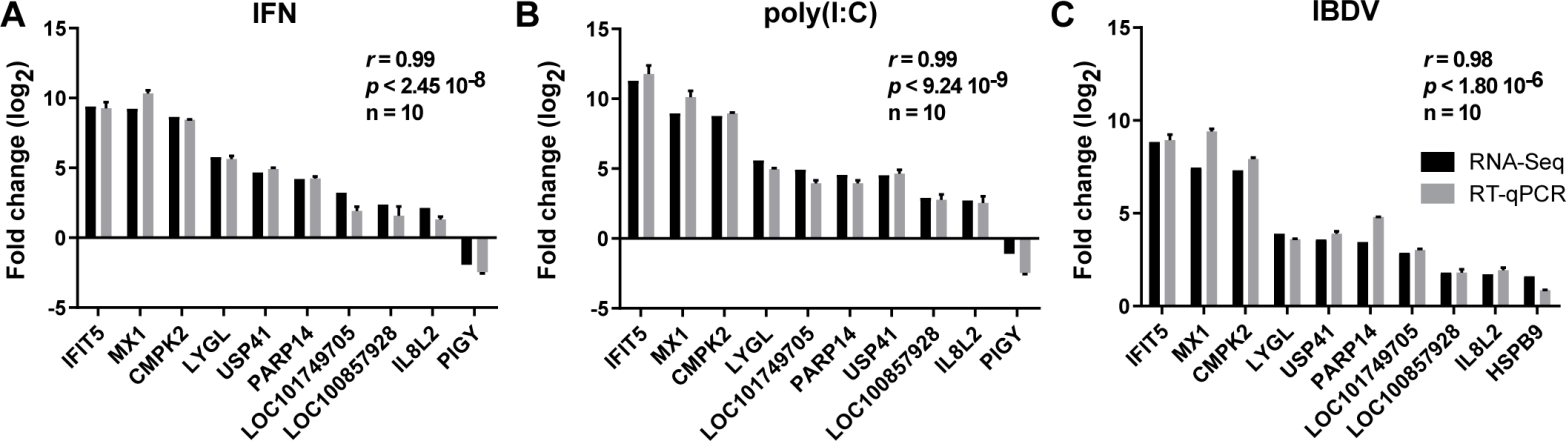
Fold change correlation between RNA-Seq and RT-qPCR analysis. **(A–C)** Comparison of log_2_ fold change of selected genes measured by RNA-Seq and RT-qPCR after IFN **(A)**, poly(I:C) **(B)**, IBDV **(C)** treatments. Error bars represent the standard deviation of biological replicate experiments analyzed in triplicate reactions. The Pearson correlation coefficient (r) and the number of pairs analysed (n) are indicated on each graph.

High-throughput sequencing yielded an average of 44 million raw reads per sample, with over 96% of reads maintaining a Phred quality score of Q30 or higher. Mapping to the chicken reference genome (GRCg7b) was highly efficient (average 88%). Principal Component Analysis (PCA) demonstrated high reproducibility between biological replicates and clear separation between treatment groups (**Supplementary File 2**). Innate immune stimulations induced widespread transcriptional remodeling in DF-1 chicken fibroblasts. In line with this, numerous genes were identified in each condition for which RNA expression was significantly altered at a false-discovery rate (FDR) lower than 0.01. In order to validate the transcriptional changes inferred from RNA-Seq, a subset of differentially expressed genes, selected on the basis of fold change and gene type, was confirmed by RT-qPCR using MIQE compliant qPCR assays. Importantly, we observed a high level of agreement between RNA-Seq and RT-qPCR analysis with Pearson’s correlation coefficients greater than 0.98, confirming the reliability of the RNA-Seq analysis (**Figures 3A–C**).

**Figure 4:**
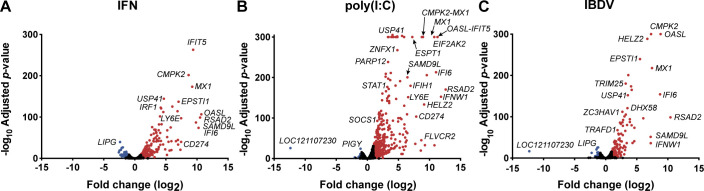
Summary of differentially regulated genes following innate immune stimulations. **(A–C)** Volcano plots of differentially expressed genes from RNA-Seq analysis comparing gene expression in IFN-treated **(A)**, poly(I:C)-transfected **(B)** and IBDV-infected **(C)** DF-1 cells with mock-treated cells. Significantly up- or downregulated genes (FDR < 0.01 and |log2 fold change| ≥1) are represented in red or blue, respectively.

To identify high-confidence innate immune-regulated genes, we applied relatively stringent criteria (FDR < 0.01 and |log_2_ fold change| ≥ 1) to the differentially expressed gene (DEG) sets obtained from each condition. Interestingly, only a minority of DEGs exhibited expression changes greater than 2-fold in either direction, suggesting that the stimulation of the innate immune responses has a modest effect on the whole transcriptional landscape of chicken fibroblasts (**Figures 4A–C**). As anticipated, IFN treatment triggered a robust antiviral response. Compared to mock-treated cells, IFN stimulation led to the upregulation of 209 protein-coding genes while 114 were repressed. Among the most strongly induced transcripts were canonical interferon-stimulated genes (ISGs) including RSAD2 (viperin), IFIT5, OASL, MX1, IFI6, and CMPK2. In contrast, the downregulated genes were predominantly associated with lipid metabolism (FADS1/2, LIPG), cholesterol biosynthesis (HMGCR, HMGCS1, AACS), and intermediate metabolism (PSAT1, KYAT3), coherent with an induced antiviral state. Poly(I:C) transfection induced a very similar innate immune signature, but on a broader scale, with 418 protein coding genes upregulated and 15 repressed. Notably, poly(I:C) stimulation led to the marked induction of multiple cytokine-related transcripts, including interferons, interleukins, chemokines, and members of the tumor necrosis factor family. This broader profile likely reflects the activation of both type I IFN signaling and additional pattern recognition receptor (PRR)-dependent pathways such as RIG-I-like receptor signaling. Although chickens and other galliform species lack the canonical RIG-I gene present in mammals and many other avian lineages, they rely on alternative cytosolic sensors such as MDA5 and LGP2 to detect double-stranded RNA and trigger downstream antiviral signaling (Barber et al., 2010; Liniger et al., 2012; Krchlíková et al., 2022; Sid et al., 2025). In DF-1 fibroblasts infected with infectious bursal disease virus (IBDV), we observed a more modest transcriptional response relative to IFN and poly(I:C) treatments. By 8 hours post-infection, 178 genes showed significant differential expression. Among these, key ISGs such as RSAD2, IFIT5, MX1, and OASL were again significantly upregulated, albeit with lower fold-changes than observed following direct IFN or poly(I:C) stimulation. These DEGs were primarily associated with immune activation, apoptosis, and cellular stress responses, consistent with early stages of host antiviral defense.

**Figure 5:**
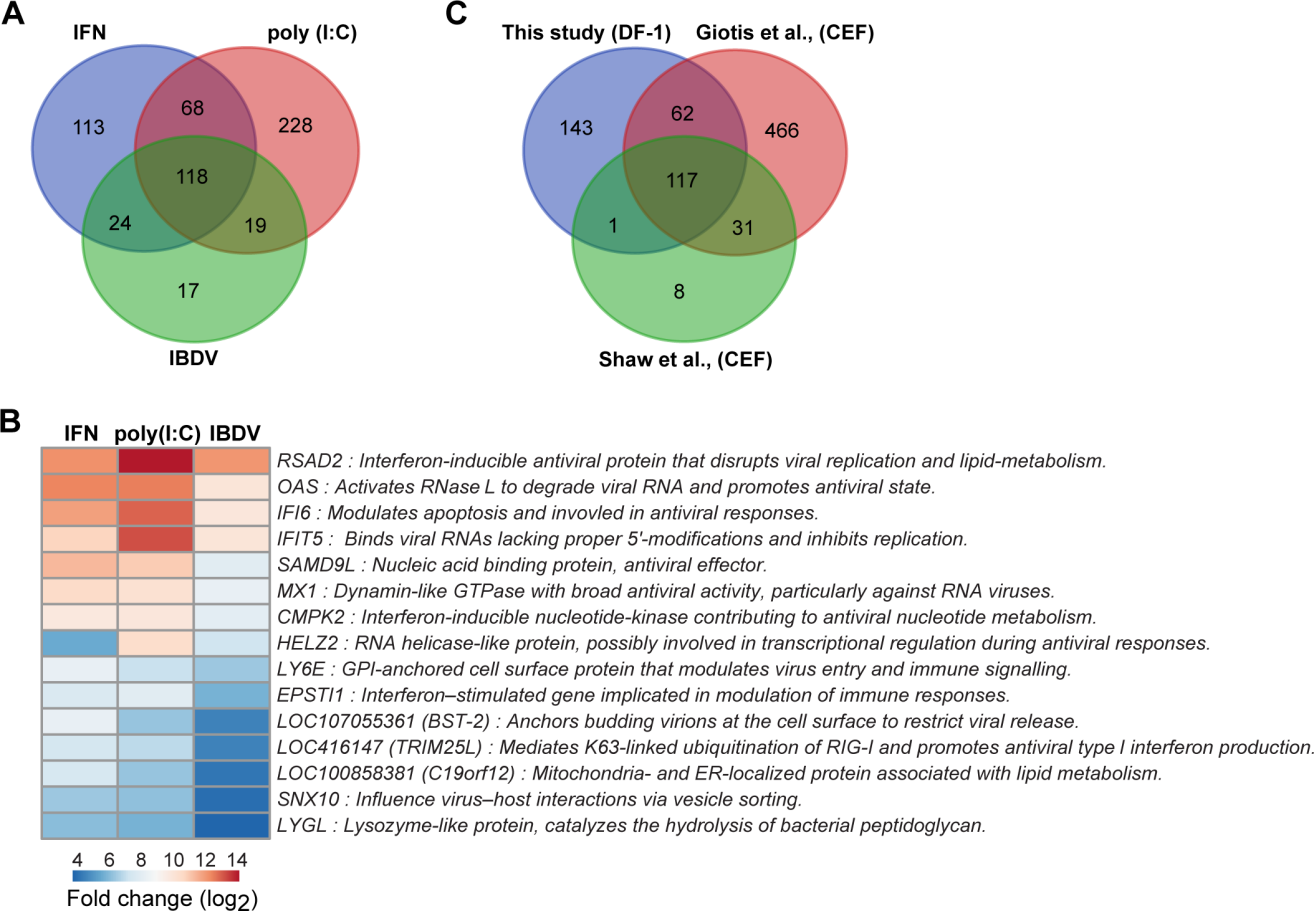
Comparative analysis of gene expression in DF-1 fibroblasts across innate immune stimulations and experimental setups. **(A)** Venn diagrams illustrating the direct comparison of the number of significantly regulated protein-coding genes in DF1 fibroblasts after treatment with type I IFN, poly(I:C) transfection or IBDV infection. **(B)** Heatmap showing the expression changes of the top 10 upregulated genes in DF1 fibroblasts after three different innate immune stimulations. Genes, along with their functional descriptions, are arranged by decreasing average enrichment for a false discovery rate (FDR) value lower than 0.01. **(C)** Venn diagrams representing the overlap of differentially regulated protein-coding genes between in DF-1 cells (this study) and CEFs from both the studies of Giotis et al., and Shaw et al., following IFN treatment.

A comparative analysis of differentially regulated protein-coding genes across conditions revealed a core set of 118 genes that were consistently regulated in response to all three stimuli (**Figure 5A**). These shared genes likely represent fundamental effectors of the chicken innate immune response, constituting a conserved antiviral response activated irrespective of the specific immune agonist. In addition, a large overlap of 137 regulated protein-coding genes was observed between poly(I:C) transfection and IBDV infection, suggesting convergence on common regulatory pathways. The most highly induced transcripts shared among all conditions are presented in **Figure 5B**. Finally, despite this substantial overlap, each treatment also elicited a unique transcriptional signature, highlighting distinct regulatory pathways that reflect specific responses to IFN treatment, poly(I:C) transfection or viral infection.

To evaluate the specificity of our results and to identify potential cell-specific differences in transcriptional activity following IFN stimulation, we compared our IFN dataset with relevant published studies aimed at the identification of chicken ISG. Two other studies were chosen on the basis of the interferon subtype, target cells and the availability of raw sequencing data (Giotis et al., 2016; Shaw et al., 2017). To circumvent annotation inconsistencies between different genome assemblies used in these studies, high quality sequence reads were re-quantified against the bona fide GRCg7b genome assembly and differentially expressed genes were identified as before. We observed a high degree of concordance between our results and that of both Shaw et al., and Giotis et al., with 117 differentially regulated protein-coding genes commonly identified between the three datasets and an additional 94 protein-coding genes differentially regulated in at least two studies (**Figure 5C**). In addition to protein-coding genes, we also analyzed our resource for non-coding RNA (ncRNA) as these are increasingly shown to be involved in innate immunity and viral infections, including in chickens (Basavappa et al., 2019; Duan et al., 2020). We observed fewer ncRNA being commonly regulated not only between the three innate immune stimulations but also across different studies, suggesting that the transcriptional landscape of non-coding RNA is more cell or stimulus-specific than for protein-coding RNA (**Supplementary Figure 1**). Yet, we detected the long non-coding RNA LOC101749705, recently identified as a positive regulator of STAT1 (Huang et al., 2024), as upregulated across all three innate immune stimulations tested but also across cell types. A full list of differentially regulated genes and expression statistics are provided in **Supplementary Table 1** and gene overlapping expression analyses are provided in **Supplementary Table 2** and **3**).

### Proteomic responses to innate immune stimulations in chicken

**Figure 6:**
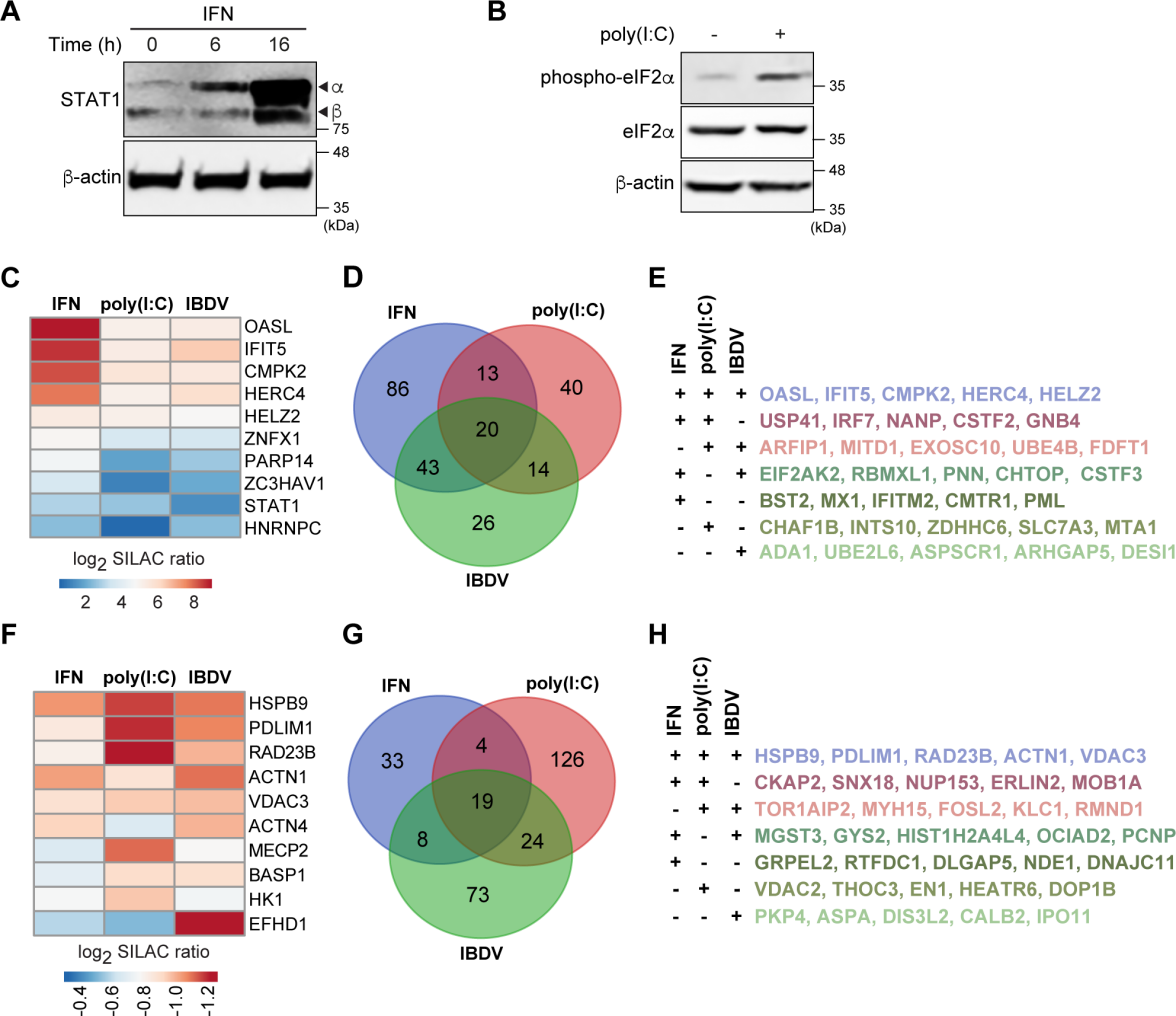
Protein abundance changes following innate immune stimulations in chicken DF-1 fibroblasts. **(A)** Western blot analysis showing increased STAT1 abundance following type I interferon stimulation for 6 h and 16 h in DF-1 chicken fibroblasts. The upper and lower bands correspond to the STAT1α and STAT1β isoforms, respectively. β-actin served as a loading control to confirm equal protein loading. **(B)** Western blot analysis of phosphorylated and total eIF2α following 6 h of poly(I:C) transfection in DF-1 chicken fibroblasts. β-actin was used as a loading control. **(C, F)** Heatmaps showing expression changes of the top 10 upregulated **(C)** or downregulated **(F)** proteins following IFN treatment, poly(I:C) transfection, and IBDV infection. Proteins are ranked by decreasing **(C)** or increasing **(F)** average abundances, with a colour gradient representing log_2_ SILAC ratios from red to blue. **(D, G)** Venn diagrams illustrating shared and unique proteins that were significantly upregulated **(D)** or downregulated **(G)** across the three conditions. **(E, H)** Lists of the top 5 proteins from each subset shown in **(D, G)**.

Analysis of gene expression during innate immune stimulations in mammalian and avian species has been extensively performed at the transcriptome level. However, post-transcriptional processes regulating protein abundances including mRNA stability, translation efficiency and protein degradation are responsible for large divergences between transcript and protein levels in most systems studied (Schwanhäusser et al., 2011; Buccitelli and Selbach, 2020; Munro et al., 2024). As a consequence, mRNA abundance is a weak predictor of protein levels, an important aspect of the genotype-phenotype relationship (Gstaiger and Aebersold, 2013; Liu et al., 2016; Lum et al., 2024). In line with this, the influence of innate immunity perturbations on the proteome landscape remains largely unknown in most species (Yan et al., 2004; Megger et al., 2017; Zhao et al., 2020). To gain insight in chicken proteome dynamics during innate immune responses, protein lysates from the SILAC labelled DF-1 cells treated in parallel for transcriptome profiling were analyzed in biological duplicates through high-resolution tandem mass spectrometry. Proteomic data quality was assessed using PTXQC analysis of MaxQuant outputs (Bielow et al., 2016). LC–MS/MS runs showed stable acquisition, low mass error, and reproducible peptide and protein identifications across biological replicates (**Supplementary File 2**). To probe for the reproducibility of protein quantitation between SILAC labels, we compared, for each condition, the quantitation ratios obtained for the medium label with that measured for the heavy label. Globally, we observed a high correlation between the two isotopic labels, with Pearson’s coefficient ranging from 0.82 to 0.99 thus validating the reproducibility of the labelling and quantification strategy (**Supplementary Figure 2**). To orthogonally validate the effectiveness of our stimulation conditions at the proteomic level, we confirmed by western blot the upregulation of STAT1 following IFN treatment and the phosphorylation of eIF2α in response to poly(I:C) (**Figures 6A, B**). However, owing to the strong positive selection and substantial protein divergence that characterize avian innate immune genes relative to their mammalian counterparts (Shultz and Sackton, 2019), most ISGs identified by mass spectrometry could not be validated using available commercial antibodies. Using a conservative approach that required at least two unique peptides per protein for the determination of the SILAC ratios, we confidently quantified 2,643, 2,417, and 2,666 proteins in response to IFN, poly(I:C), and IBDV treatments, respectively. Next, to identify proteins differentially regulated in response to innate immune stimulations, log_2_-transformed SILAC ratios from duplicate experiments were averaged and subjected to z-score analysis with proteins presenting a z-score lower or greater than 2.58 (corresponding to p-value lower than 0.01) considered as differentially expressed (**Supplementary Figure 3**).

Consistent with our transcriptomic analysis, the abundance of most quantified proteins remained stable, with each stimulus inducing a distinct proteomic signature. IFN treatment led to the most pronounced upregulation of proteins, both in number and magnitude with 162 proteins showing a statistically significant increase in abundance following IFN treatment, while 64 were downregulated compared to the mock condition (**Figures 6C–H**). As expected, IFN exposure strongly induced well-characterized ISGs including OASL, IFIT5, and CMPK2, consistent with the activation of the JAK/STAT signaling pathway. In contrast, poly(I:C) triggered the most extensive downregulation (87 proteins upregulated and 173 downregulated), strongly suppressing metabolic regulators like HK1–2 and VDAC1-3. IBDV infection resulted in a mixed response (103 proteins upregulated and 124 downregulated), affecting stress response proteins (HSPB9, PDLIM1) and components of the actin cytoskeleton. The distinct patterns observed across conditions highlight differential cellular outcomes in response to viral infection and immune stimulation. IFN treatment predominantly activates antiviral pathways, poly(I:C) induces a subset of genes shared with IFN but also exerts a suppressive effect on metabolic processes, and IBDV infection modulates stress responses, cytoskeletal organization, and immune-related pathways. In summary, our findings illustrate the extensive impact of innate immune activation on cellular metabolism and gene expression. The upregulation of antiviral effectors and interferon-stimulated genes underscores a coordinated host response to infection, while the downregulation of mitochondrial and translational machinery reflects a strategic cellular adaptation to restrict viral replication and propagation. Together, these data provide a comprehensive overview of how cells reprogram their proteome in response to innate immune stimuli, highlighting the balance between antiviral defense mechanisms and cellular homeostasis. A complete list of differentially regulated proteins with relevant statistics is shown in additional **Supplementary Table 4**.

### Transcription-translation coupling during innate immune response in DF-1 fibroblasts

**Figure 7:**
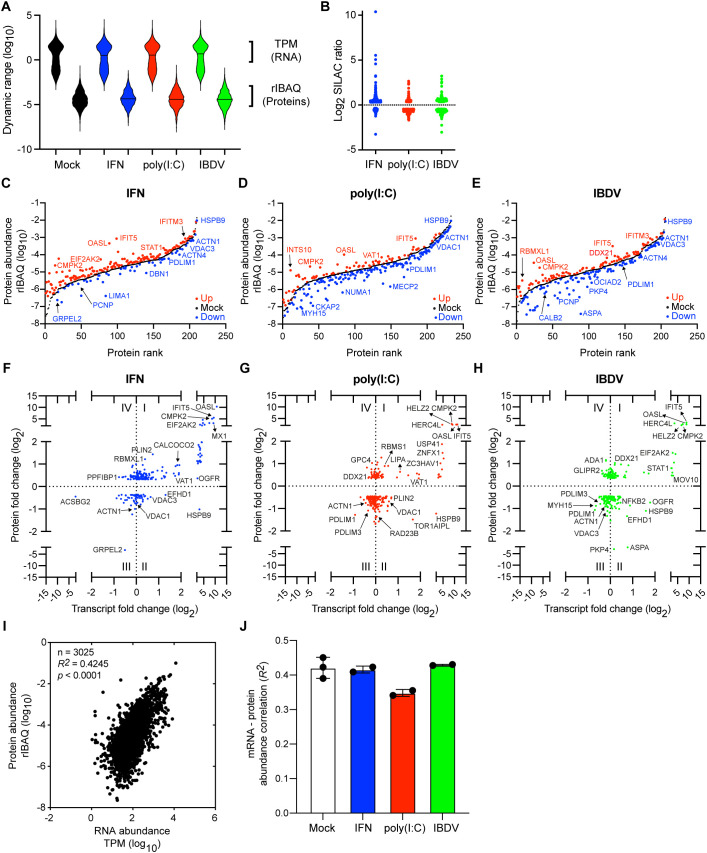
Transcription and translation integration in chicken DF-1 fibroblast. **(A)** Dynamic range of RNA (TPM) and protein abundances (riBAQ) displayed on a log_10_ scale. **(B)** Log_2_-transformed SILAC ratios showing statistically significant protein fold changes in response to distinct immune stimulation conditions. **(C–E)** Rank-ordered protein abundances (log_10_ riBAQ) in response to IFN **(C)**, poly(I:C) **(D)**, or IBDV **(E)**, sorted from lowest to highest abundance in mock-treated cells; black dots indicate basal (Mock) proteins, and red and blue dots indicate statistically significant up- and downregulated proteins, respectively. **(F–H)** Scatterplots of mRNA and protein fold-changes (log_2_) following IFN **(F)**, poly(I:C) **(G)**, and IBDV **(H)** treatments. Each point represents a statistically significantly regulated protein, with corresponding transcript fold-change data. Quadrants are designated with Roman numerals indicating concordant (I-III) or discordant (II-IV) regulation between transcript and protein levels. **(I)** mRNA and protein levels in mock-infected cells. Scatterplot comparing the mean mRNA and protein abundance expressed as log_10_-transformed TPM and relative iBAQ, respectively. The coefficient of determination (*R^2^*), number of pairs analyzed (n) and statistical significance (*p*) are indicated. **(J)** Impact of various innate immune challenges on transcription-translation correlation in chicken DF-1 fibroblasts. Error bars represent the standard deviation across biological replicates.

To dissect the regulatory mechanisms driving these proteomic shifts, we quantified steady-state transcript levels using RNA-Seq and measured corresponding protein abundances via mass spectrometry. RNA abundances were expressed in TPM (Transcripts Per Million), a metric that normalizes read counts based on transcript length and sequencing depth, providing a measure analogous to molar concentration (Li and Dewey, 2011). Protein abundances were estimated using the relative iBAQ (Intensity-Based Absolute Quantification) metric, in which iBAQ intensities were normalized to the total iBAQ sum within each replicate, ensuring accurate cross-sample comparisons (Schwanhäusser et al., 2011; Shin et al., 2013; He et al., 2019). The measured abundances of transcripts and proteins spanned a wide dynamic range, covering approximately seven orders of magnitude in a bimodal distribution for RNA and six for proteins across all experimental conditions. This extensive dynamic range highlights the complexity of the DF-1 fibroblast transcriptome and proteome (**Figure 7A** and **Supplementary Figures 4A–C**). Analysis of differentially expressed proteins revealed a broad distribution of protein regulation and abundances, reflecting the overall distribution across the proteome. Upon immune stimulation, upregulated proteins exhibited a more pronounced increase in abundance compared to the decrease observed for downregulated proteins (**Figure 7B**). Notably, the proteins most significantly upregulated in response to immune stimulation tended to display lower basal abundance levels compared to those that were downregulated (**Figures 7C–E**). Direct comparison of mRNA and protein fold-changes revealed that the relationship between transcription and translation was stimulus-dependent (**Figures 7F–H**). A common feature across all stimuli was the concordant upregulation of a core set of ISGs (quadrant I), including OASL, IFIT5, CMPK2, and STAT1, confirming a transcriptionally-driven antiviral response. Symmetrically, quadrant III highlighted widespread concordant downregulation of mitochondrial and energy metabolism following IFN treatment. Poly(I:C) transfection induced the most dramatic effect, with suppression of numerous ribosomal proteins, metabolic enzymes (HK1, PDPR), and cytoskeletal components (ACTN1, LPP, PDLIM1), consistent with a PKR-mediated global shutdown of biosynthetic pathways. IBDV infection, however, produced a more selective reduction of cytoskeletal (PDLIM1-3, MYH15) and metabolism-related proteins, presumably indicating a targeted host–pathogen interaction. Analysis of the discordant quadrants II and IV further revealed extensive post-transcriptional regulation. Quadrant II contained genes that were transcriptionally upregulated but suppressed at the protein level, consistent with translational repression or targeted degradation. Examples include the heat shock protein HSPB9 and the methyl-CpG-binding protein MECP2, both strongly induced at the transcript level yet depleted as proteins. Conversely, quadrant IV comprised proteins that accumulated despite stable or decreasing mRNA levels, pointing to enhanced translational efficiency or protein stabilization. This was particularly evident during IBDV infection, where proteins such as PPFIBP1 increased despite transcript downregulation, suggesting selective preservation of pro-host factors. A table comparing transcript and protein regulation following innate immune perturbations of DF-1 chicken fibroblasts is provided in **Supplementary Table 5**. To investigate the biological processes associated the observed host response, we performed a functional enrichment analysis on the pooled gene sets from all stimuli across the four quadrants of RNA-protein correlations (**Supplementary Figure 5**). Quadrant I (concordant upregulation) was highly enriched for RNA processing as well as antiviral functions, including ‘Defense response to virus’ and ‘Negative regulation of viral genome replication.’ Conversely, quadrant III (concordant downregulation) revealed a significant suppression of biosynthetic processes and translational machinery, with high enrichment in terms such as ‘Translation’ and ‘Ribosome Biogenesis, ‘ a signature consistent with the global host-shutoff occurring during viral infection. Quadrant II (mRNA Up/Proteins Down) was modestly enriched for processes related to the cytoskeleton and cell mobility but also for stress granules formation. Finally, quadrant IV (mRNA Down/Proteins Up) showed enrichment for processes related to the nucleus and its envelope. Integration of mRNA and protein abundances revealed a moderate correlation across conditions, with coefficients of determination (R²) ranging from 0.34 to 0.44 (log–log scale), indicating that mRNA levels account for approximately 40% of the variability in protein expression in DF-1 chicken fibroblasts (**Figures 7I–J** and **Supplementary Table 6**). Interestingly, transcriptional-translational coupling was weaker following poly(I:C) transfection compared to mock-treated cells, IFN-treated cells, or IBDV-infected cells. This reduction is likely due to PKR-dependent translational arrest as evidenced by the phosphorylation of the eIF2α subunit (**Figure 6B**). These values are in line with findings from other eukaryotic systems (reviewed in (Buccitelli and Selbach, 2020)), including yeast (*R²* ≈ 0.455) (Marguerat et al., 2012), maize (*R²* ≈ 0.4121) (Ponnala et al., 2014), mouse (*R²* ≈ 0.41) (Schwanhäusser et al., 2011), and humans (average *R²* = 0.4389 ± 0.1261 across > 20 cell lines and tissues) (Edfors et al., 2016). Together, these results underscore the complex interplay between transcriptional and post-transcriptional mechanisms that shape proteome dynamics in naïve cells but also during innate immune responses. While mRNA levels provide a moderate predictive value for protein abundance, post-transcriptional regulation, particularly translational control and protein turnover, plays a sizeable role in modulating proteins expression during innate immune responses in chicken DF-1 fibroblasts, especially following poly(I:C) treatment.

## Discussion

The avian innate immune system constitutes a critical first line of defense against viral infections. Nevertheless, it remains poorly characterized, and its molecular components and regulatory mechanisms continue to drive active investigations. In this study, we analyzed the transcriptomic and proteomic responses of DF-1 chicken fibroblasts to three distinct immune stimuli: type I interferon, poly(I:C) (a synthetic double-stranded RNA analog), and Infectious Bursal Disease Virus (IBDV), a dsRNA genome virus. At the transcriptome level, we identified a core set of over 100 genes consistently upregulated across all conditions, representing conserved components of the antiviral response. Each stimulus induced unique transcriptional programs, with poly(I:C) triggering the most robust cytokine induction. The consistent upregulation of interferon-stimulated genes (ISGs), lncRNAs and antiviral effectors, such as OASL, IFIT5, and CMPK2, aligns with known mechanisms of Type I interferon signaling in chickens and mammals. To further contextualize our findings, we performed a comparative meta-analysis using publicly available transcriptomic datasets from chicken innate immune studies. This analysis revealed a robust set of immune-related genes consistently regulated across diverse cell types and experimental conditions. The strong overlap between our data and these external datasets reinforces the reproducibility of cellular antiviral signatures and supports the broader relevance of our findings to avian immune biology. Nonetheless, while these signatures define a fundamental intrinsic antiviral response in a stromal context, we acknowledge that this landscape represents a baseline cellular state. In the context of a whole organism, this response would be integrated with the specialized functions of professional immunological sentinels, such as lymphocytes and macrophages. Furthermore, while the attenuated PBG98 strain provided a robust stimulus to map the host’s functional capacity, wild-type pathogenic strains may further modulate these signatures through enhanced immune evasion or complex interferon antagonism. Consequently, our findings establish a high-resolution reference map of the avian fibroblast response, providing a benchmark against which the specific effects of viral virulence and specialized immune cell signaling can be measured in future *in vivo* studies.

Beyond the transcriptome, our proteomic analyses provide additional insights into the cellular response to immune stimulation that cannot be captured by RNA-Seq alone. Using SILAC-based quantitative proteomics, we quantified over 2,400 proteins across treatment conditions and identified extensive changes in protein abundance, many of which were not mirrored at the transcript level. While this coverage is substantial, it does not represent the entire chicken proteome, particularly low-abundance or small proteins that may play a role in innate immune signaling. Consequently, our conclusions are based on the subset of proteins that were reliably quantified. These findings underscore the value of proteomic data in capturing regulation responses concealed to RNA-based approaches, highlighting extensive post-transcriptional regulation during the early antiviral response. In line with this, our data reveal a large-scale post-translational response characterized by repression of host proteins, particularly those involved in mitochondrial metabolism (VDAC1-3) and protein synthesis (small and large ribosomal proteins, G3BP), two energy-intensive processes. This coordinated suppression suggests a conserved immunometabolic reprogramming in avian cells, similar to the shifts from energy production to defense-oriented metabolism reported in mammalian systems (Jovanovic et al., 2015; Fritsch and Weichhart, 2016; O’Neill et al., 2016; Olson et al., 2021).This reallocation of cellular resources likely prioritizes the synthesis of antiviral effectors over routine house-keeping functions. Accordingly, upregulated ISGs and stress-related proteins may contribute to this shift by promoting protein turnover and deprioritizing non-essential cellular functions in favor of antiviral defenses. Whether this remodeling is mediated by the proteasome, autophagy, or other quality-control mechanisms remains an important avenue for future investigation. While transcriptomic analyses have historically dominated immune profiling efforts, our findings demonstrate that proteomic data are essential for uncovering additional layers of immune control (Yan et al., 2004; Megger et al., 2017; Busnadiego et al., 2025). Our results also confirm and extend previous reports on conserved features of the antiviral response in birds, notably the strong upregulation of RSAD2, OASL, IFI6, IFIT5 and SAMD9L at the transcriptomic level (Giotis et al., 2016; Shaw et al., 2017; Santhakumar et al., 2019; Dai et al., 2020). Importantly, our work adds a systematic, quantitative proteomic dimension, which has been largely missing from prior chicken innate immunity studies and identifies OASL, IFIT5, CMPK2, HERC4 and HELZ2 as the most upregulated proteins across all stimulation conditions, revealing regulatory layers that are not captured by transcriptome data alone. Ontological analyses of proteins, stratified by their basal abundance (as estimated by relative intensity-based absolute quantification, riBAQ), revealed widespread presence of immune, stress response, and metabolic processes among the most dynamically regulated proteins. These findings place our study in strong alignment with existing avian transcriptomic and proteomic literature, reinforcing the conservation of core antiviral programs across taxa (Megger et al., 2017; Shaw et al., 2017; Ye et al., 2022).

We also explored the relationship between mRNA and protein abundance and found a moderate transcription–translation correlation comparable to values reported in other eukaryotes such as yeast, plant, and mammals (Schwanhäusser et al., 2011; Marguerat et al., 2012; Ponnala et al., 2014; Edfors et al., 2016; Buccitelli and Selbach, 2020). Notably, this correlation was weakened following poly(I:C) transfection, likely due to translational arrest mediated by PKR-dependent phosphorylation of eIF2α (Taylor et al., 2005; Cesaro and Michiels, 2021). In contrast, IBDV infection and IFN treatment preserved transcriptional–translational coupling at the time points analyzed (6 and 8 hours, respectively). These observations underscore the importance of translational control as a context-dependent mechanism shaping immune response (Jovanovic et al., 2015).

Overall, our findings not only validate the utility of integrative omics approaches in characterizing immune responses within avian models but also provide valuable resources for functional genomics and systems biology. The presented datasets constitute a high-confidence reference for future studies on chicken immunity and may aid in the identification of markers and targets relevant to improving poultry resistance to infectious diseases. While our study provides a comprehensive catalog of transcriptomic and proteomic changes, the mechanistic basis of the observed post-transcriptional regulation remains to be experimentally validated in future studies. In addition, future work could leverage this dataset to investigate the temporal dynamics of ISG regulation, characterize non-coding RNA functions, or explore the functional impact of the most strongly up- and downregulated proteins, particularly those stratified by basal expression using riBAQ estimates. Additionally, mechanistic studies dissecting the role of post-translational repression and the identity of the responsible degradation pathways would offer deeper insights into host–pathogen interactions and immune stress adaptation in avian systems.

In conclusion, while this integrative multi-omics analysis provides a precise map of the innate immune response in DF-1 fibroblasts, it represents a foundational step in characterizing avian antiviral immunity. The conserved host signatures and regulatory patterns identified here establish a high-confidence benchmark for further verification in primary cell types and *in vivo* models. Such future studies will be essential to elucidate how these cell-intrinsic signatures are modulated by systemic physiological factors and how they drive disease outcomes in target organs.

## Data Availability

The datasets presented in this study can be found in online repositories. The names of the repository/repositories and accession number(s) can be found below: https://www.ncbi.nlm.nih.gov/, PRJNA418693 https://www.ebi.ac.uk/pride/archive/, PXD057686.
