# Supplementary material for: Combined transcriptomic and proteomic analyses define conserved host signatures during innate immune perturbations in DF-1 chicken fibroblasts
Source: Front Cell Infect Microbiol. 2026 May 29;16:1813484. doi: 10.3389/fcimb.2026.1813484 (PMC13259848; doi:10.3389/fcimb.2026.1813484)
Supplement: Supplementary file 1 [file DataSheet1.pdf]

Supplementary Material

Combined transcriptomic and proteomic analyses define conserved host signatures during innate immune perturbations in DF-1 chicken fibroblasts

Frederic Sorgeloos[1,2,5*], Efstathios S. Giotis[3,6], Kate Heesom[4], Michael A. Skinner[3], Ian G. Goodfellow[1*]

[1] Division of Virology, Department of Pathology, University of Cambridge, Addenbrooke's Hospital, Hills Road, Cambridge CB2 2QQ, United Kingdom

[2] de Duve Institute, Université catholique de Louvain, Brussels, Belgium

[3] Section of Virology, Department of Infectious Disease, Department of Medicine, Imperial College London, South Kensington Campus, London SW7 2AZ, United Kingdom

[4] Proteomics facility, School of Biochemistry, University of Bristol, Biomedical Sciences Building, University Walk, Bristol BS8 1TD, United Kingdom

[5] Institut National de la Recherche Scientifique, Centre Armand-Frappier Santé Biotechnologie, Laval, Québec, Canada

[6] School of Life Sciences, University of Essex, Colchester CO4 3SQ, United Kingdom

- **Supplementary Figures 1 to 5**
- **Supplementary Tables 1 to 6**
- **Supplementary File 1**
- **Supplementary File 2**

Supplementary Figure 1.

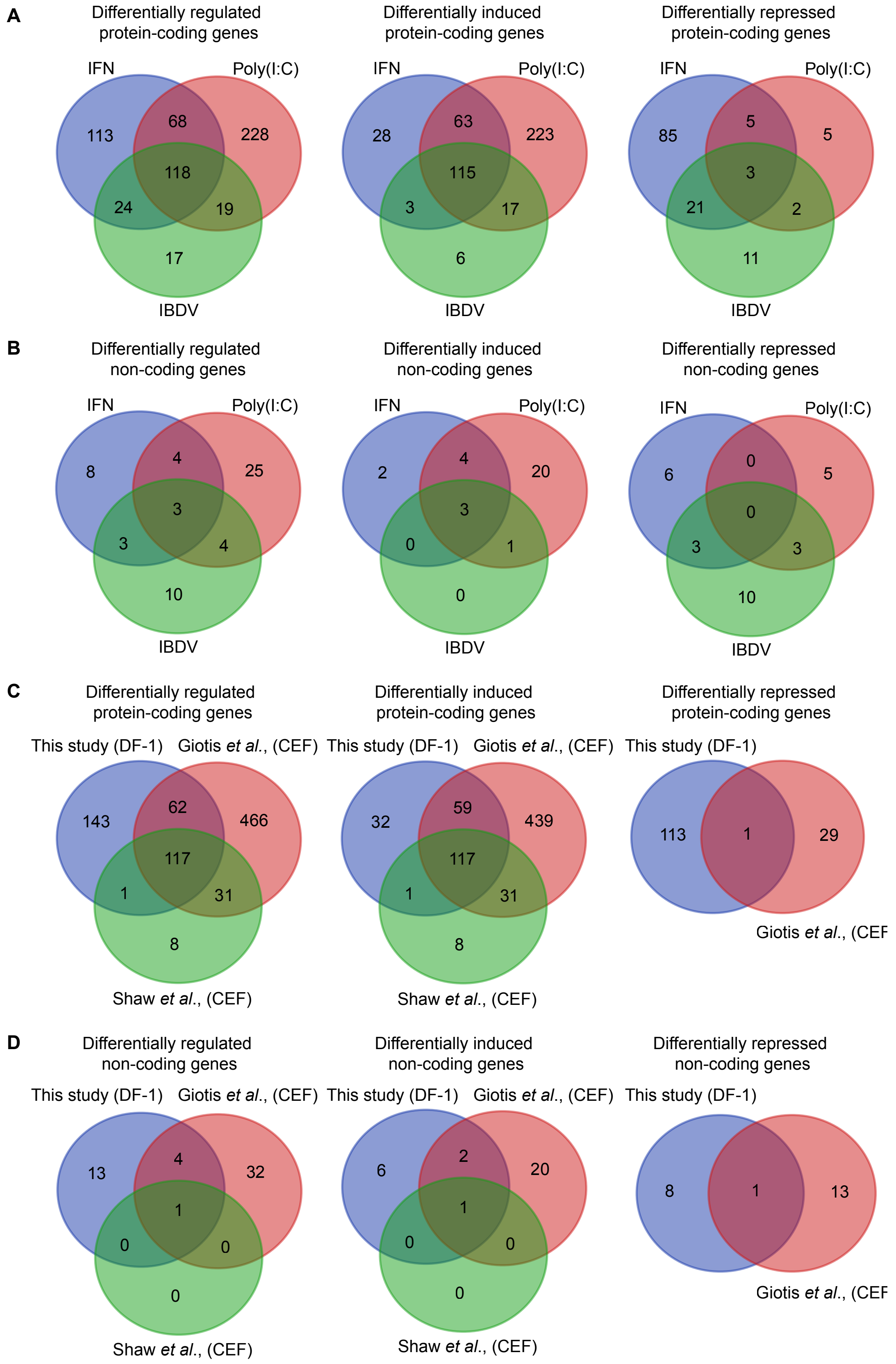

Supplementary Figure 1. Commonalities in transcriptional responses between different innate immune stimulations and across different studies.

(A) Venn diagrams showing the overlap of differentially regulated protein-coding genes (upper left), differentially induced protein-coding genes (upper middle) or differentially expressed protein-coding genes (upper right) after treatment with type I IFN, poly(I:C) transfection or IBDV infection. (B) As in (A) but showing the overlap of non-coding genes. (C) Venn diagrams representing the overlap, following IFN treatment, of differentially regulated protein-coding genes between in DF-1 cells (this study, upper left) and CEFs from both the studies of Giotis *et al.,* (upper middle) and Shaw *et al.,* (upper right). (D) As in (C) but showing the overlap of non-coding genes.

Supplementary Figure 2.

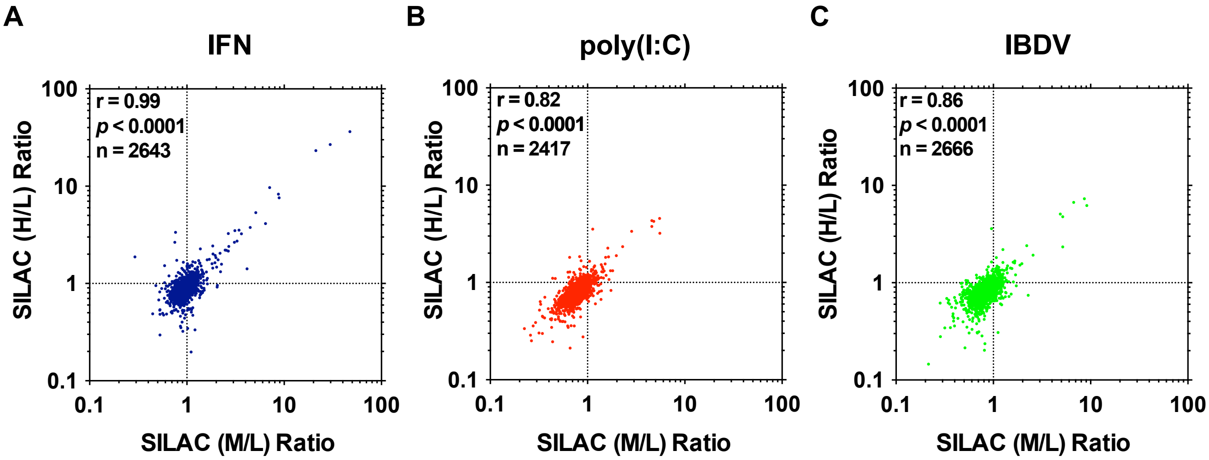

Supplementary Figure 2. Reproducibility of SILAC-based protein quantitation between biological replicates.

(A-C) Scatterplots of SILAC ratios from two biological replicates following IFN treatment (A), poly(I:C) transfection (B) and IBDV infection (C). The Pearson correlation coefficient (r), p-value (p) and the number of pairs analysed (n) are indicated on each scatterplot.

Supplementary Figure 3.

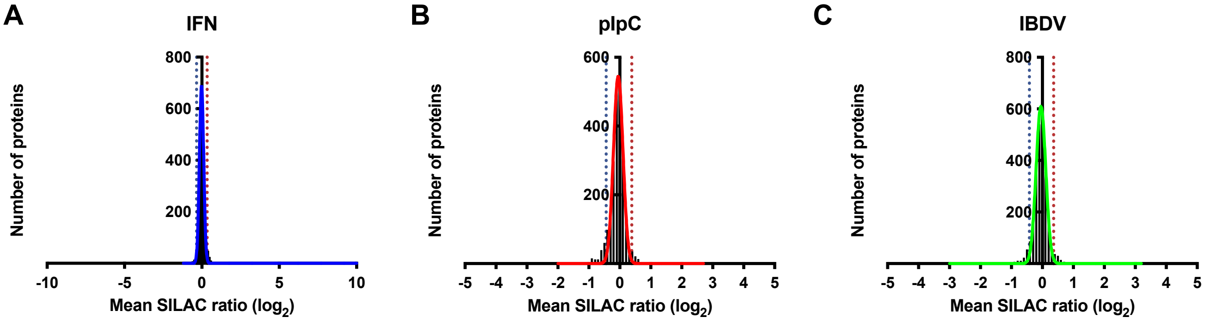

Supplementary Figure 3. Z-score analysis of protein abundance changes following innate immune stimulations of DF-1 cells.

The $\log_2$ SILAC ratios from duplicate experiments were averaged and subjected to z-score analysis to assess deviations in protein abundance levels from the mean. The dashed red and blue lines represent positive and negative 2.58 standard deviation from the mean, respectively, corresponding to significantly upregulated (red) and downregulated (blue) proteins at a *p*-value lower than 0.01.

Supplementary Figure 4.

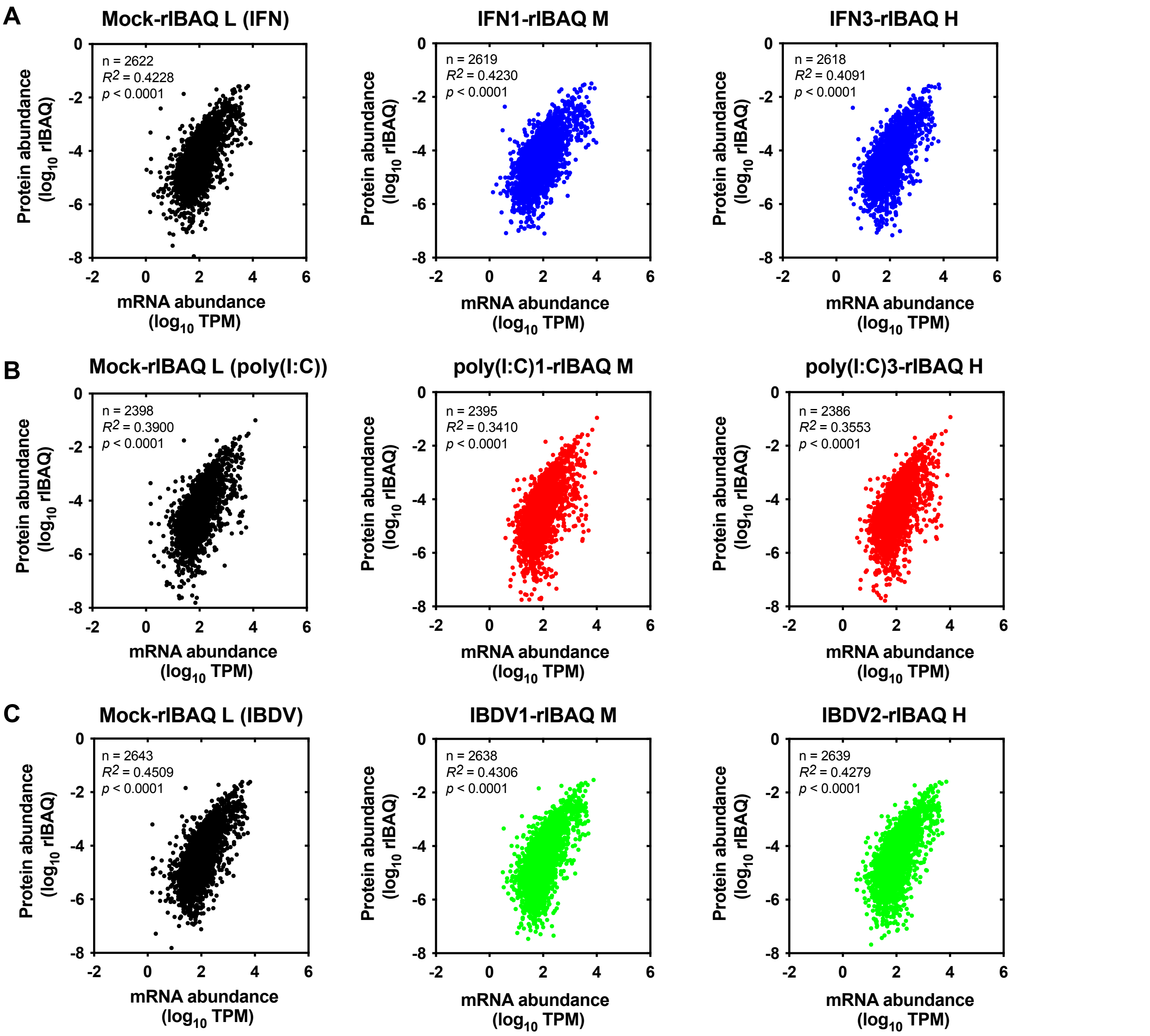

Supplementary Figure 4. Integration of genome-wide transcriptome and proteome data in chicken DF-1 fibroblasts during innate immune stimulations.

Scatterplots showing the correlation between matched mRNA and protein expression levels under individual conditions: (A) interferon-treated, (B) poly(I:C)-transfected, and (C) IBDV-infected cells. For each condition, the coefficient of determination (R^2), number of matched gene-protein pairs (n), and statistical significance (p-value) are indicated.

Supplementary Figure 5.

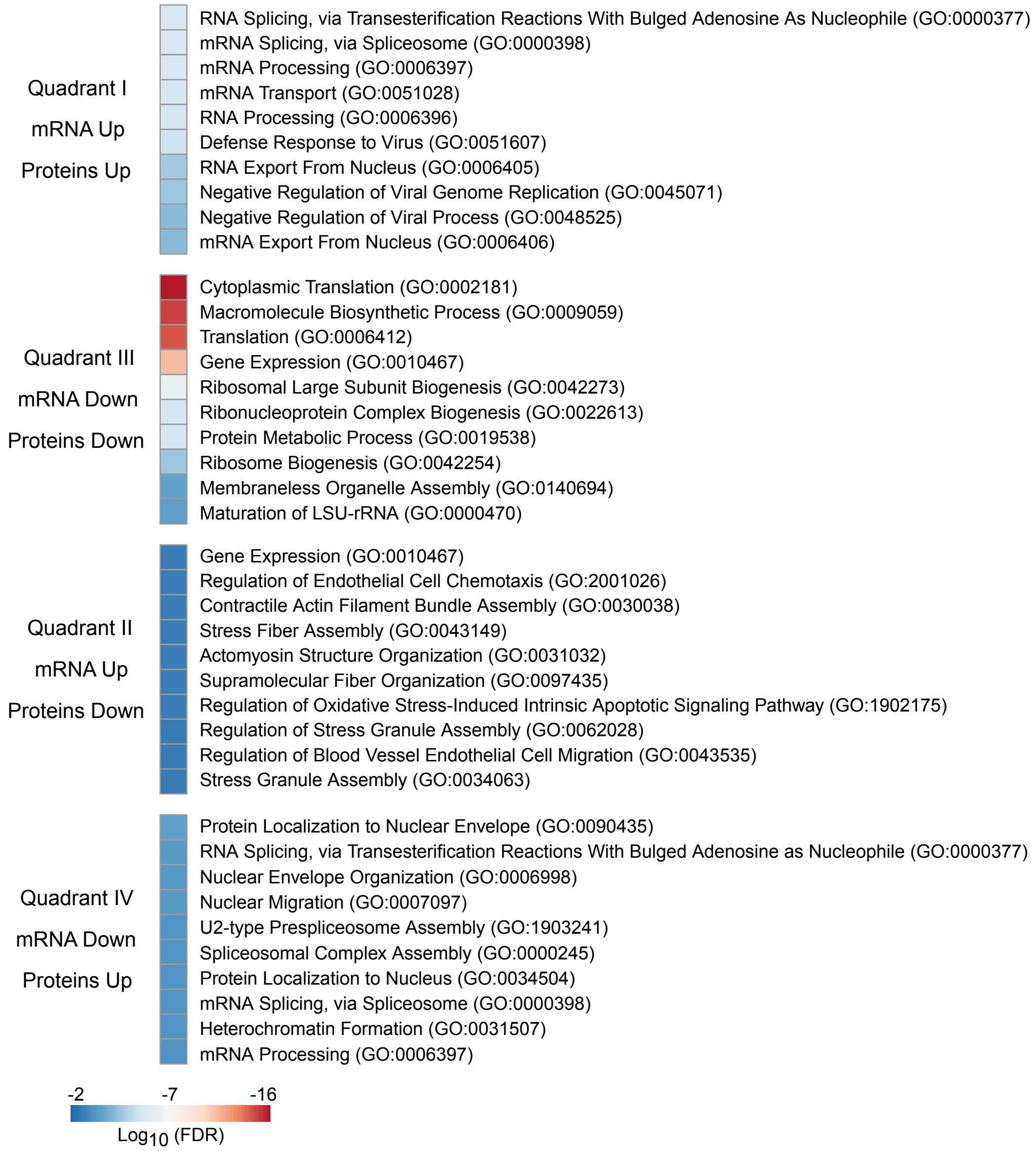

Supplementary Figure 5. Functional landscape of conserved concordant and discordant RNA-protein profiles.

Functional enrichment analysis of Gene Ontology (GO) Biological Process terms (top10) for genes identified across four quadrants of expression. To identify regulatory signatures, gene sets from all stimuli (IFN, poly(I:C), and IBDV infection) were pooled by quadrant for enrichment analysis. The color scale represents the statistical significance of enrichment expressed as $\log_{10}$ False Discovery Rate (FDR). Terms were considered significant at a FDR < 0.05.

Supplementary Tables

Supplementary-Table-1-RNASeq-Differential-Expression-Analysis.xlsx

Supplementary-Table-2-RNASeq-Overlap-IFN-pIpC-IBDV.xlsx

Supplementary-Table-3-RNASeq-IFN-Meta-analysis.xlsx

Supplementary-Table-4-SILAC-Analysis.xlsx

Supplementary-Table-5-Transcripts-Proteins-Regulation.xlsx

Supplementary-Table-6-RNA-Protein-Integration-TPM-riBAQ.xlsx

Supplementary Files

Supplementary-File-1.pdf

Supplementary-File-2.zip
