# Supplementary material for: Combined transcriptomic and proteomic analyses define conserved host signatures during innate immune perturbations in DF-1 chicken fibroblasts
Source: Front Cell Infect Microbiol. 2026 May 29;16:1813484. doi: 10.3389/fcimb.2026.1813484 (PMC13259848; doi:10.3389/fcimb.2026.1813484)
Supplement: Supplementary file 2 [file DataSheet2.zip › Supplementary-File-2/RNASeq-PTXQC-MS-Quality-Control.pdf]

Transcriptomics Data Quality Control

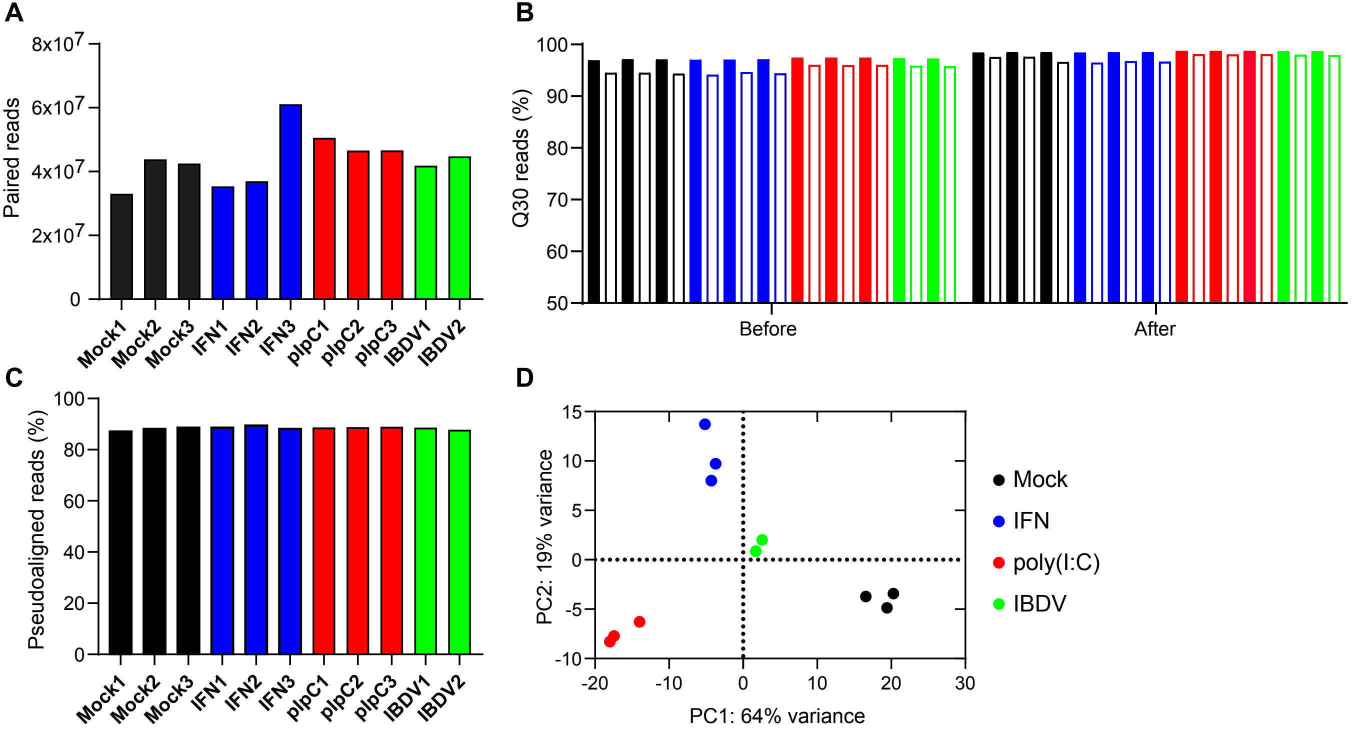

RNA-seq quality control metrics.

(A) Sequencing library sizes. (B) Base quality scores (Q30) before and after read trimming using the Trimmomatic pipeline. Solid bars represent forward reads, whereas open bars represent reverse reads. (C) Summary of read mapping statistics following read pseudo-alignment. (D) Principal component analysis (PCA) of normalized gene expression

Proteomics Data Quality Control
(PTXQC)

IFN

Performance overview

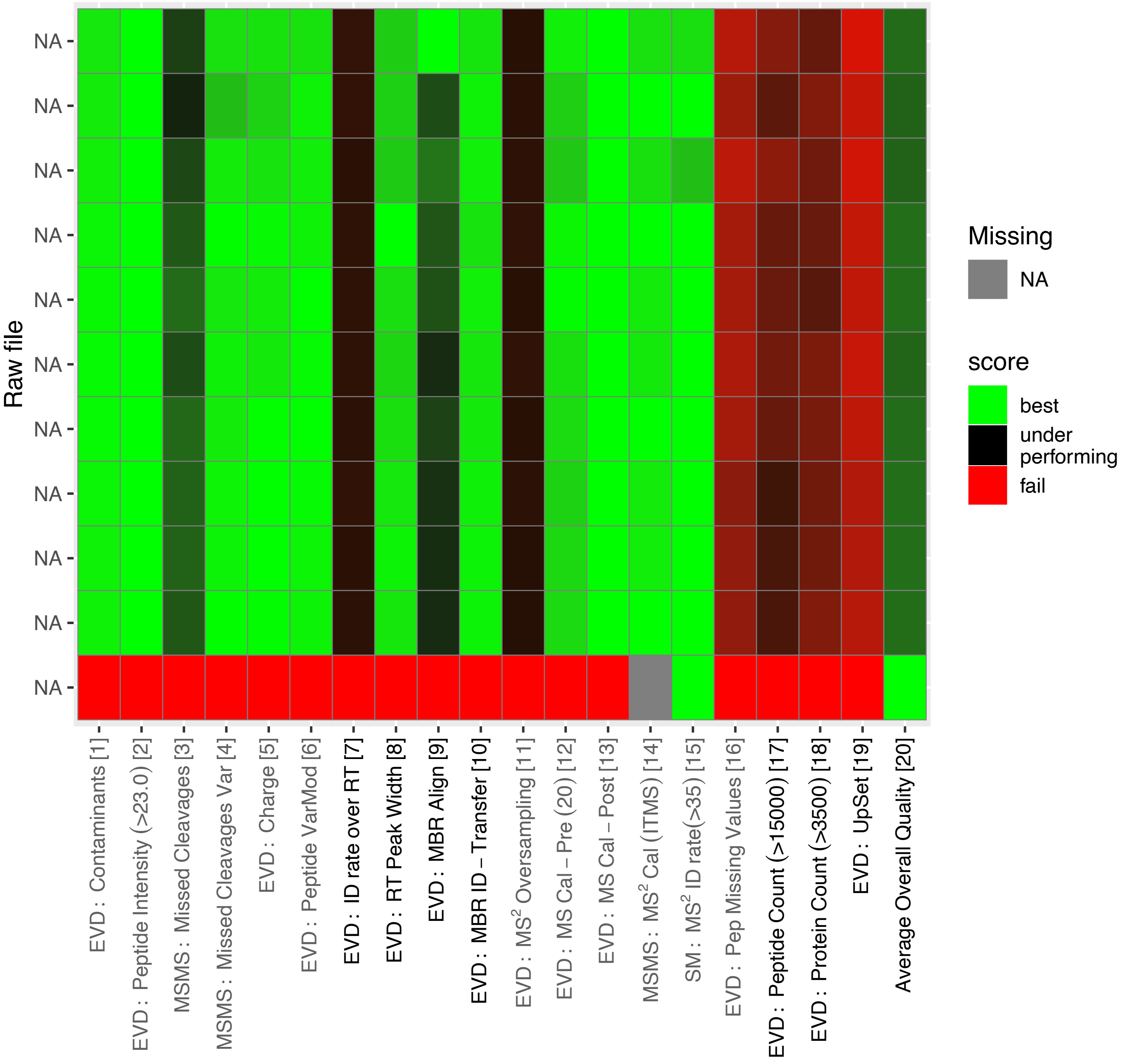

Mapping of Raw files to their short names
Mapping source: file (user–defined)

original	short name	best effort
IFN_slice_1	IFN_slice_1	IFN_slice_1
IFN_slice_10	IFN_slice_10	IFN_slice_10
IFN_slice_2	IFN_slice_2	IFN_slice_2
IFN_slice_3	IFN_slice_3	IFN_slice_3
IFN_slice_4	IFN_slice_4	IFN_slice_4
IFN_slice_5	IFN_slice_5	IFN_slice_5
IFN_slice_6	IFN_slice_6	IFN_slice_6
IFN_slice_7	IFN_slice_7	IFN_slice_7
IFN_slice_8	IFN_slice_8	IFN_slice_8
IFN_slice_9	IFN_slice_9	IFN_slice_9
Total	Total	Total

PAR: parameters

parameter	value	parameter	value
Advanced ratios	True	MS/MS deisotoping tolerance ..	7
Advanced site intensities	True	MS/MS deisotoping tolerance ..	ppm
Alignment ion mobility windo..	1	MS/MS dependent losses (ASTR..	True
Alignment time window [min]	20	MS/MS dependent losses (FTMS..	True
Calculate peak properties	False	MS/MS dependent losses (ITMS..	True
Da interval. (ASTRAL)	100	MS/MS dependent losses (TOF)	True
Da interval. (FTMS)	100	MS/MS dependent losses (UNKN..	True
Da interval. (ITMS)	100	MS/MS higher charges (ASTRAL..	True
Da interval. (TOF)	100	MS/MS higher charges (FTMS)	True
Da interval. (UNKNOWN)	100	MS/MS higher charges (ITMS)	True
Date of writing	04/15/2025 08:13:34	MS/MS higher charges (TOF)	True
Decoy mode	revert	MS/MS higher charges (UNKNOW..	True
Disable MD5	False	MS/MS recalibration (ASTRAL)	False
Discard unmodified counterpa..	True	MS/MS recalibration (FTMS)	False
Epsilon score for mutations	True	MS/MS recalibration (ITMS)	False
Evaluate variant peptides se..	True	MS/MS recalibration (TOF)	False
Find dependent peptides	False	MS/MS recalibration (UNKNOWN..	False
Fixed andromeda index folder		MS/MS tol. (ASTRAL)	25 ppm
iBAQ	True	MS/MS tol. (FTMS)	20 ppm
iBAQ log fit	True	MS/MS tol. (ITMS)	0.5 Da
Include contaminants	True	MS/MS tol. (TOF)	25 ppm
Label min. ratio count	2	MS/MS tol. (UNKNOWN)	20 ppm

protein.faa

PAR: parameters

parameter	value	parameter	value
Machine name	DESKTOP−SUMVKIL	MS/MS water loss (ASTRAL for..	False
Main search max. combination..	200	MS/MS water loss (ASTRAL)	True
Match between runs	True	MS/MS water loss (FTMS for c..	False
Match ion mobility window [i..	0.05	MS/MS water loss (FTMS)	True
Match unidentified features	True	MS/MS water loss (ITMS for c..	False
Matching time window [min]	0.8	MS/MS water loss (ITMS)	True
Max mods in site table	3	MS/MS water loss (TOF for cr..	False
Max. peptide length for unsp..	25	MS/MS water loss (TOF)	True
Max. peptide mass [Da]	4600	MS/MS water loss (UNKNOWN fo..	False
Min. delta score for modifie..	6	MS/MS water loss (UNKNOWN)	True
Min. delta score for unmodif..	0	Peptides used for protein qu..	Razor
Min. peptide Length	7	Protein FDR	0.01
Min. peptide length for unsp..	8	PSM FDR	0.01
Min. peptides	1	PSM FDR Crosslink	0.01
Min. razor peptides	1	Razor protein FDR	True
Min. score for modified pept..	40	Require MS/MS for LFQ compar..	True
Min. score for unmodified pe..	0	Second peptides	True
Min. unique peptides	0	Separate LFQ in parameter gr..	False
Modifications included in pr..	Oxidation (M)<br>Acetyl (Protein N−term)	Site FDR	0.01
MS/MS ammonia loss (ASTRAL f..	False	Site tables	Oxidation (M)Sites.txt
MS/MS ammonia loss (ASTRAL)	True	Stabilize large LFQ ratios	True
MS/MS ammonia loss (FTMS for..	False	Top MS/MS peaks per Da inter..	12

protein.faa

PAR: parameters

parameter	value	parameter	value
MS/MS ammonia loss (FTMS)	True	Top MS/MS peaks per Da inter..	8
MS/MS ammonia loss (ITMS for..	False	Top MS/MS peaks per Da inter..	16
MS/MS ammonia loss (ITMS)	True	Top MS/MS peaks per Da inter..	16
MS/MS ammonia loss (TOF for ..	False	Top MS/MS peaks per Da inter..	12
MS/MS ammonia loss (TOF)	True	Use delta score	False
MS/MS ammonia loss (UNKNOWN ..	False	Use Normalized Ratios For Oc..	True
MS/MS ammonia loss (UNKNOWN)	True	Use only unmodified peptides..	True
MS/MS deisotoping (ASTRAL)	True	User name	tosor
MS/MS deisotoping (FTMS)	True	Variation mode	None
MS/MS deisotoping (ITMS)	False	Version	2.6.5.0
MS/MS deisotoping (TOF)	True	Write accumulatedMsmsScans t..	False
MS/MS deisotoping (UNKNOWN)	True	Write allPeptides table	False
MS/MS deisotoping tolerance ..	7	Write DIA fragments quant ta..	False
MS/MS deisotoping tolerance ..	ppm	Write DIA fragments table	False
MS/MS deisotoping tolerance ..	0.15	Write ms3Scans table	False
MS/MS deisotoping tolerance ..	Da	Write msmsScans table	False
MS/MS deisotoping tolerance ..	0.01	Write msScans table	False
MS/MS deisotoping tolerance ..	Da	Write mzRange table	False
MS/MS deisotoping tolerance ..	0.01	Write pasefMsmsScans table	False
MS/MS deisotoping tolerance ..	Da		

protein.faa

PG: PCA of 'raw intensity'

(excludes contaminants)

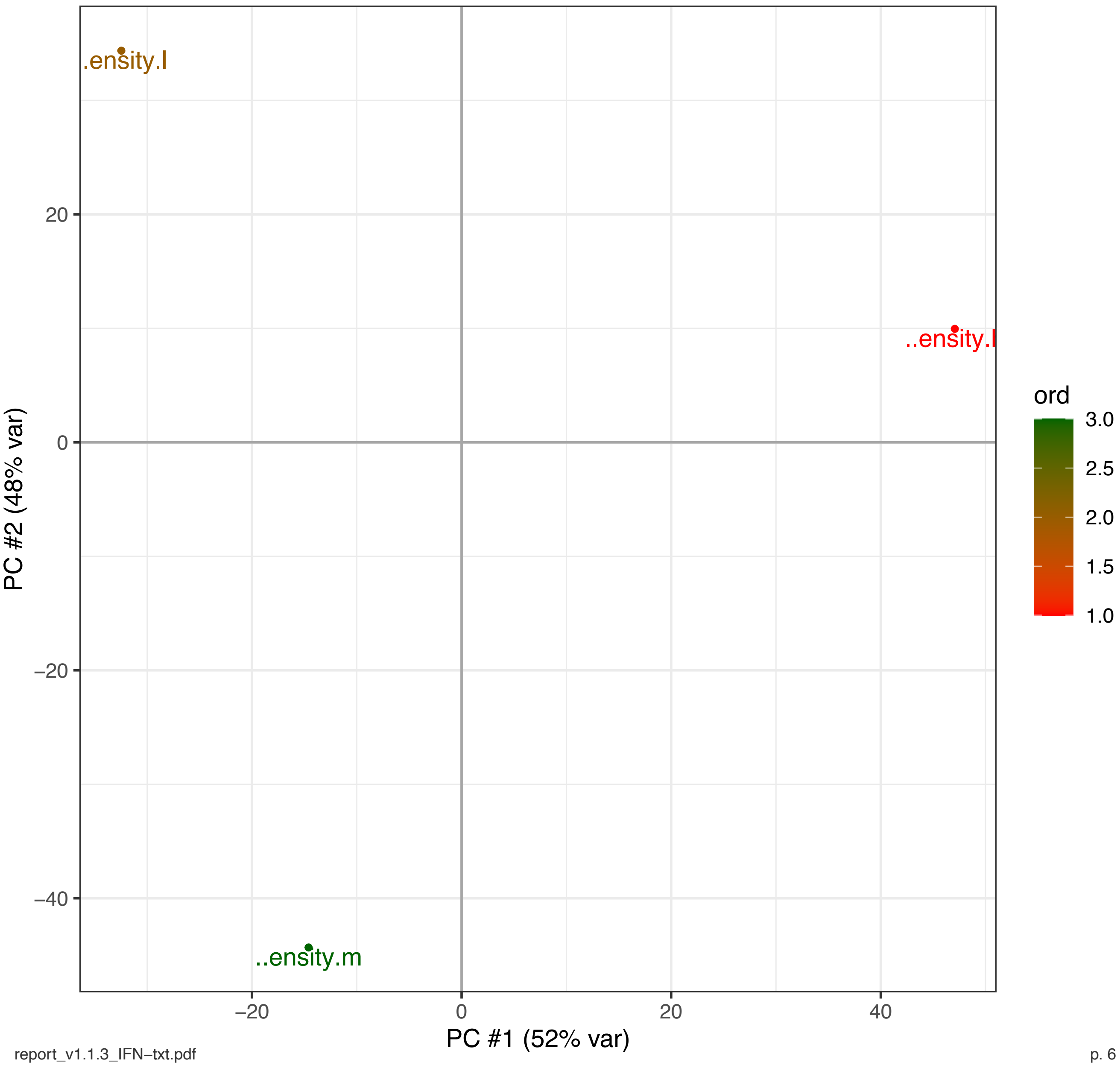

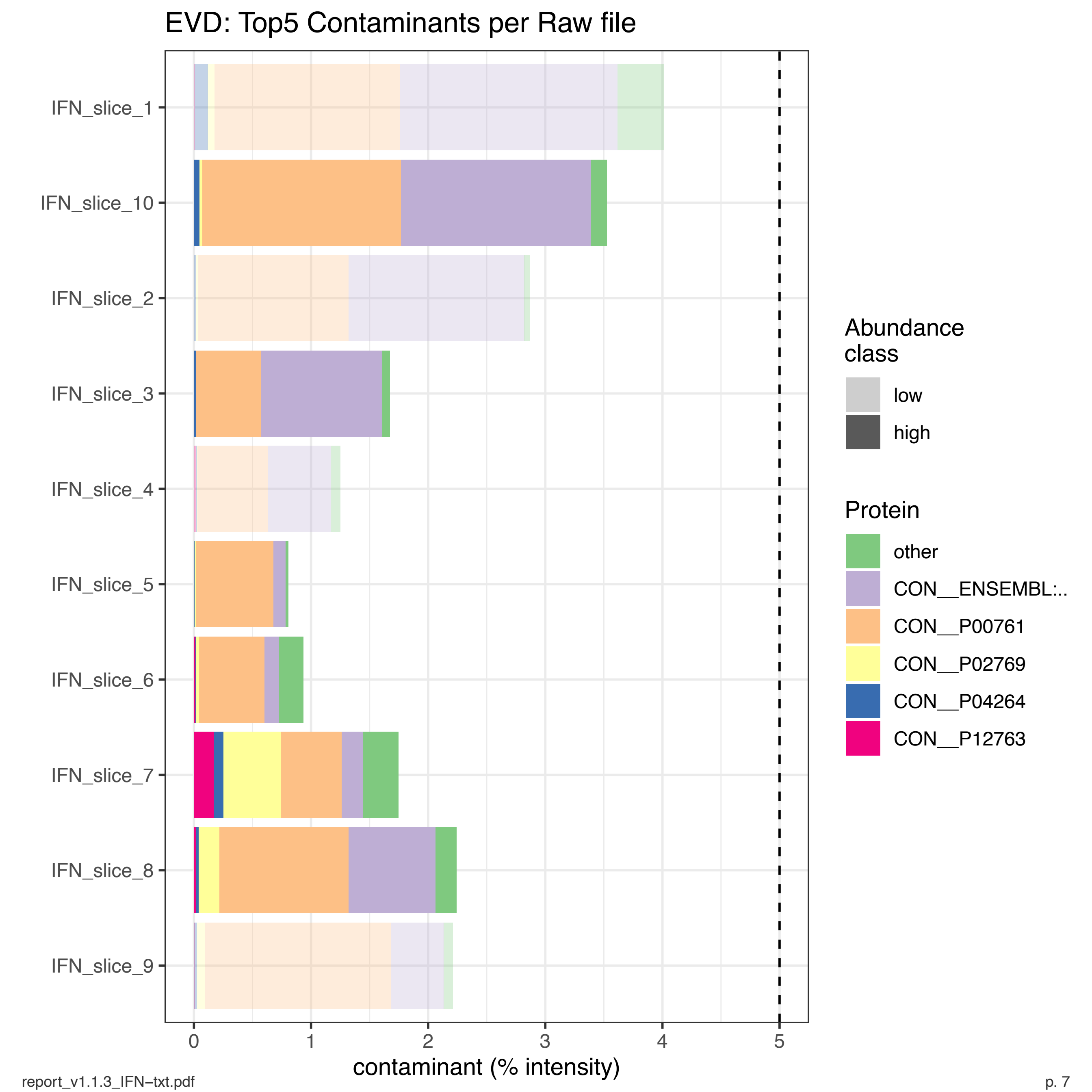
EVD: Top5 Contaminants per Raw file
IFN_slice_1
IFN_slice_10
IFN_slice_2
IFN_slice_3
IFN_slice_4
IFN_slice_5
IFN_slice_6
IFN_slice_7
IFN_slice_8
IFN_slice_9
0
1
2
3
4
5
contaminant (% intensity)
Abundance class
low
high
Protein
other
CON__ENSEMBL:..
CON__P00761
CON__P02769
CON__P04264
CON__P12763

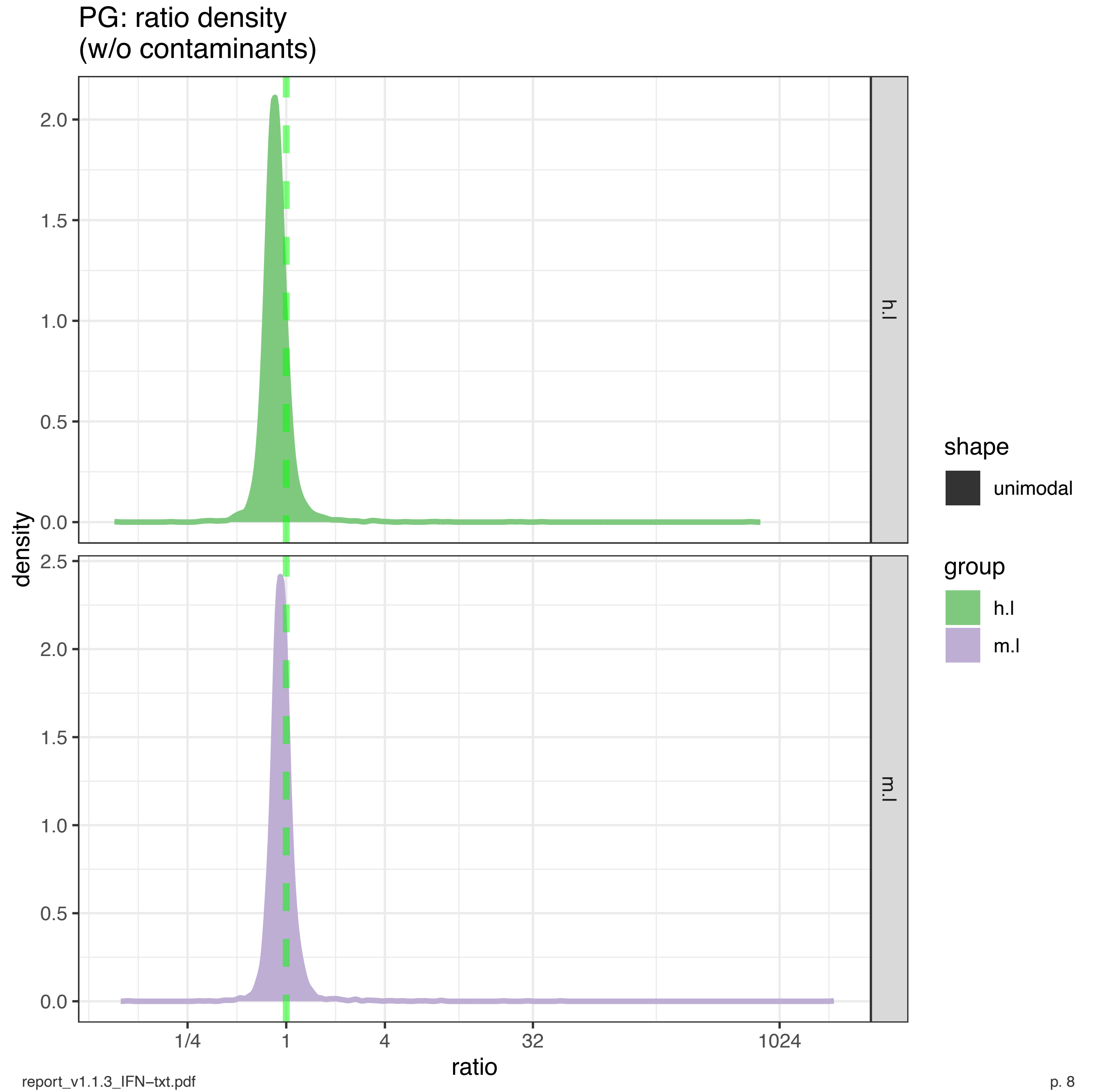
PG: ratio density
(w/o contaminants)
density
2.0
1.5
1.0
0.5
0.0
2.5
2.0
1.5
1.0
0.5
0.0
h.l
m.l
1/4
1
4
32
1024
ratio
shape
unimodal
group
h.l
m.l

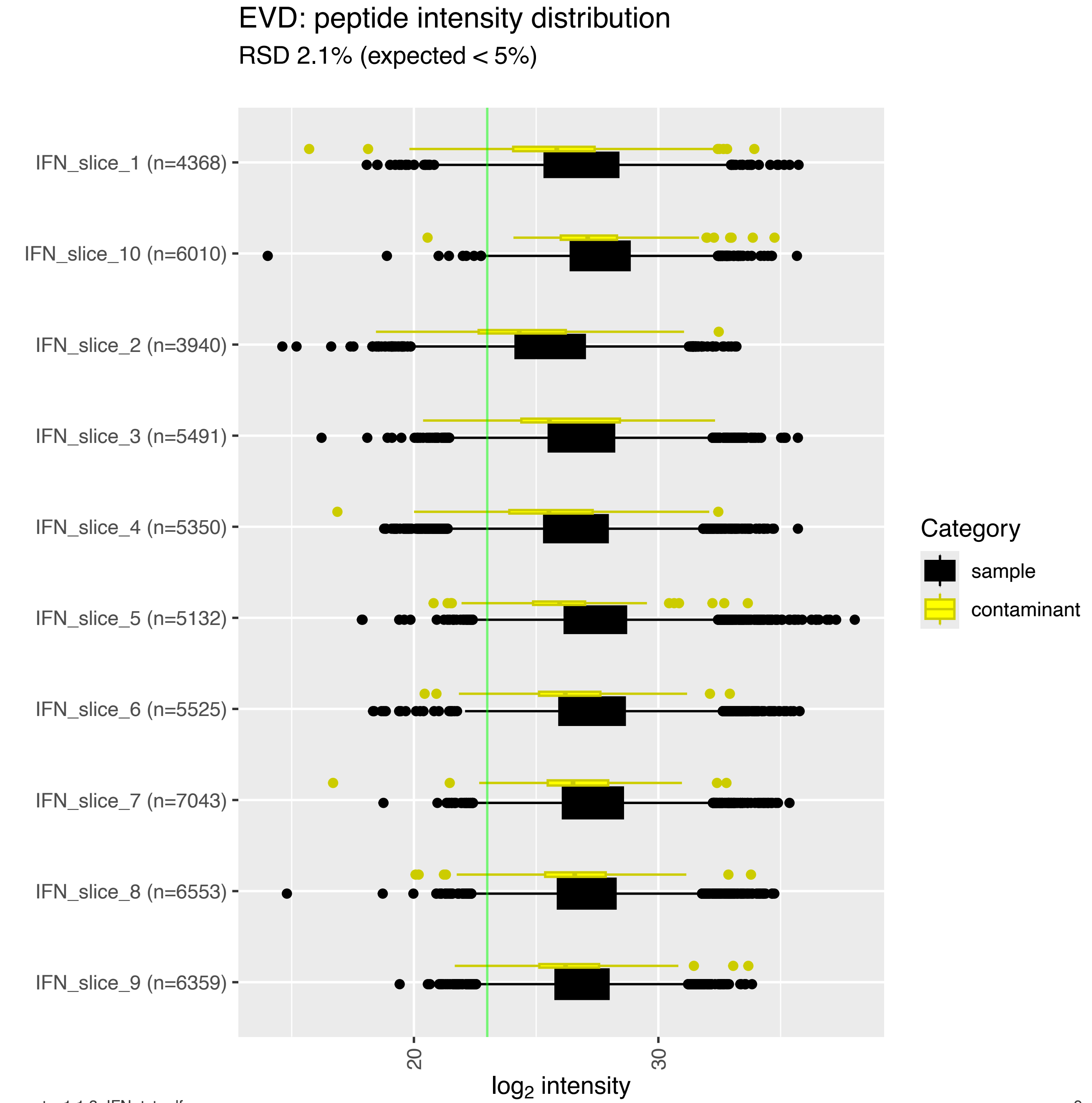
EVD: peptide intensity distribution
RSD 2.1% (expected < 5%)
IFN_slice_1 (n=4368)
IFN_slice_10 (n=6010)
IFN_slice_2 (n=3940)
IFN_slice_3 (n=5491)
IFN_slice_4 (n=5350)
IFN_slice_5 (n=5132)
IFN_slice_6 (n=5525)
IFN_slice_7 (n=7043)
IFN_slice_8 (n=6553)
IFN_slice_9 (n=6359)
20
30
log2 intensity
Category
sample
contaminant

PG: intensity distribution

RSD 0.2% (w/o zero int.; expected < 5%)
RSD 0.2% [high RSD --> few peptides])

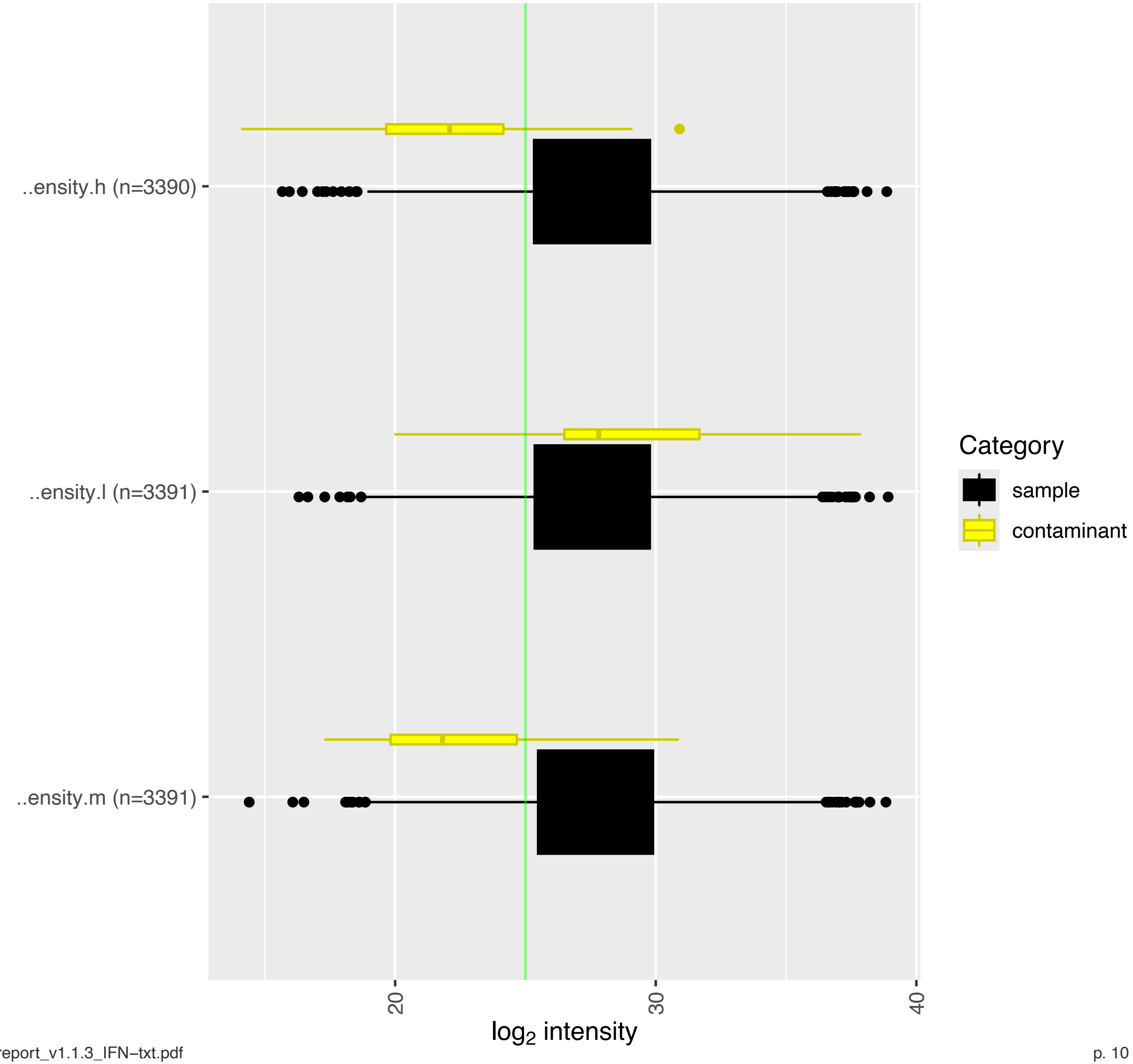

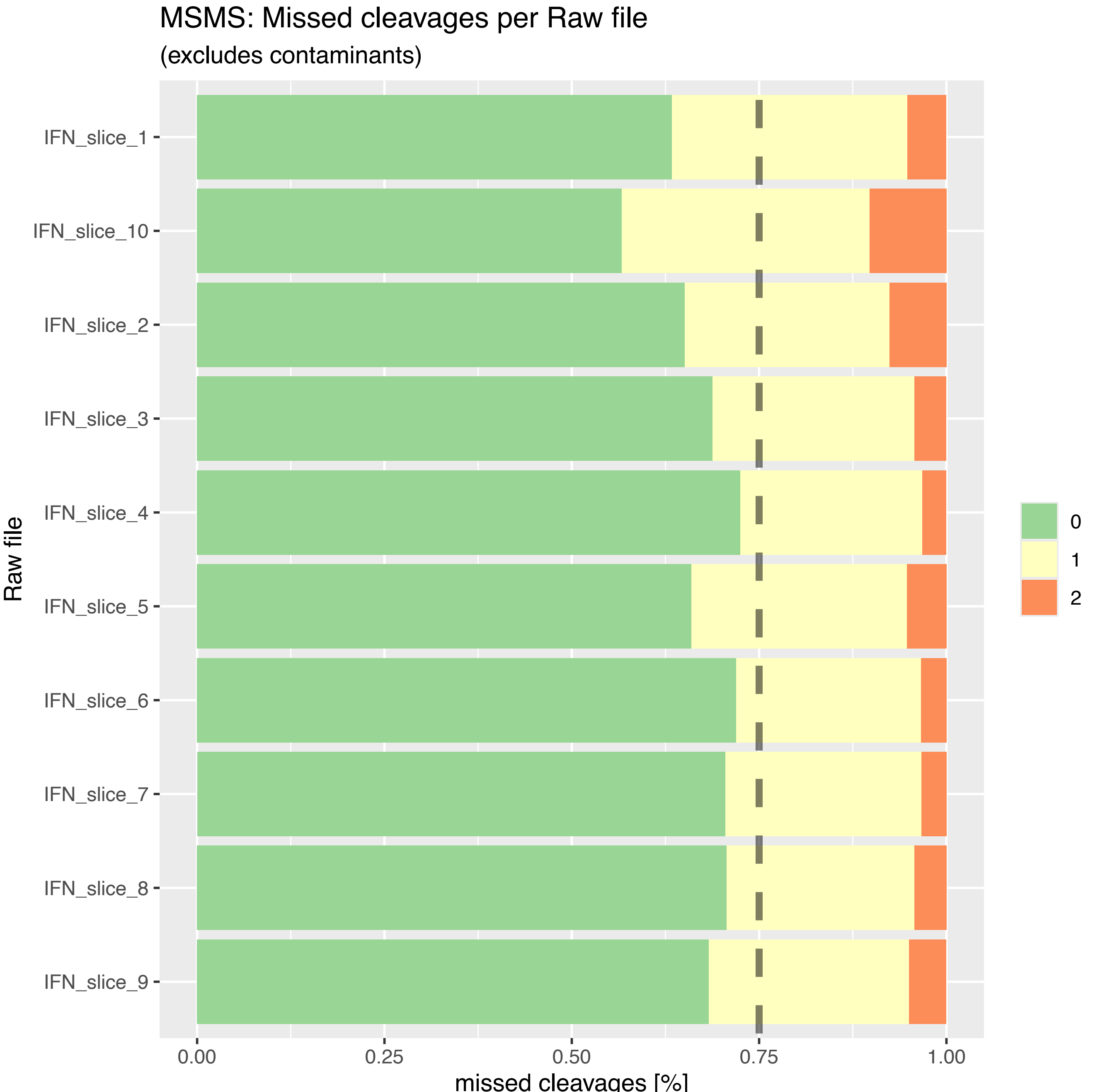
MSMS: Missed cleavages per Raw file
(excludes contaminants)
Raw file
IFN_slice_1
IFN_slice_10
IFN_slice_2
IFN_slice_3
IFN_slice_4
IFN_slice_5
IFN_slice_6
IFN_slice_7
IFN_slice_8
IFN_slice_9
0.00
0.25
0.50
0.75
1.00
missed cleavages [%]
0
1
2

EVD: charge distribution

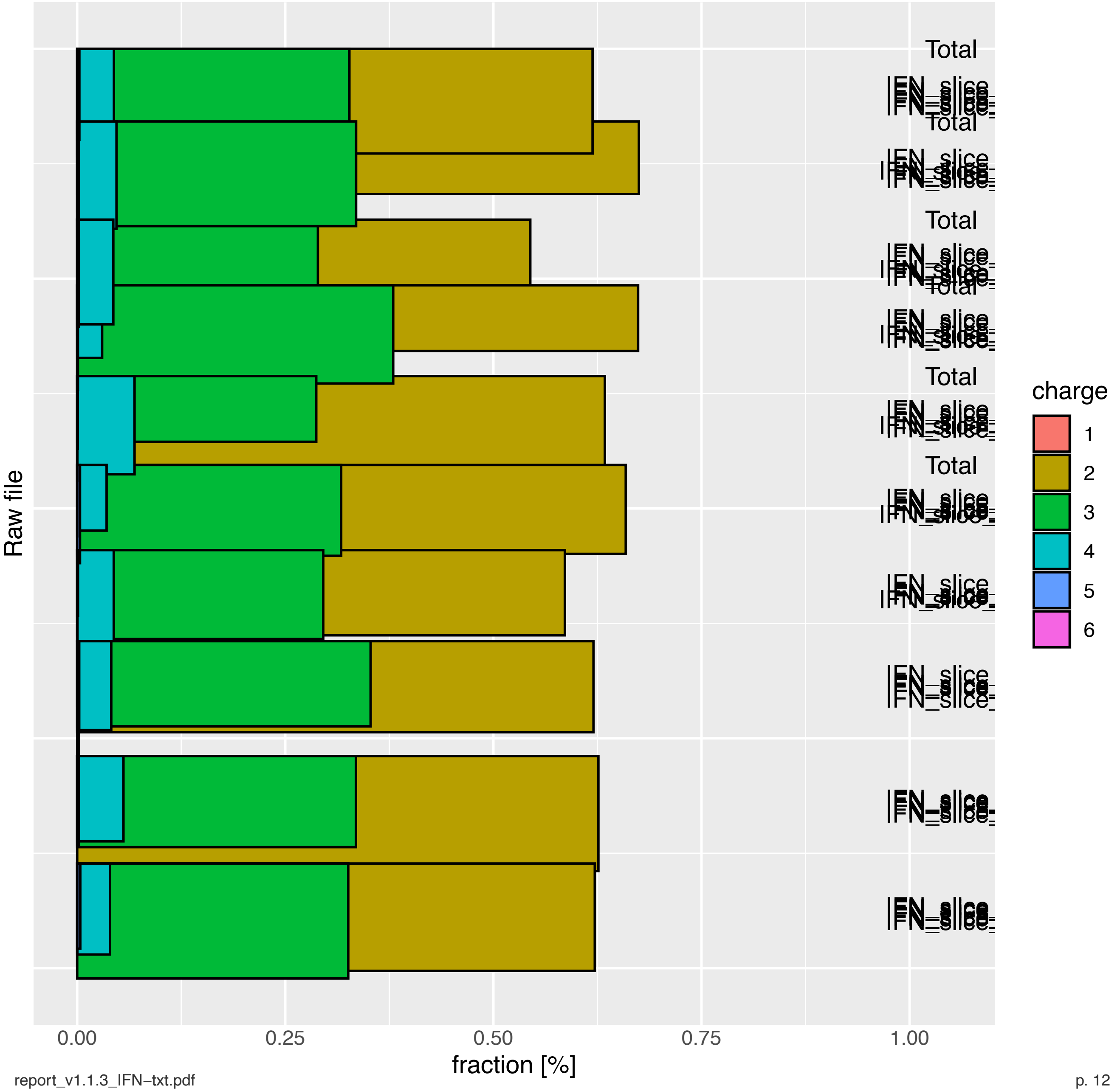

EVD: variable modifications per Raw file

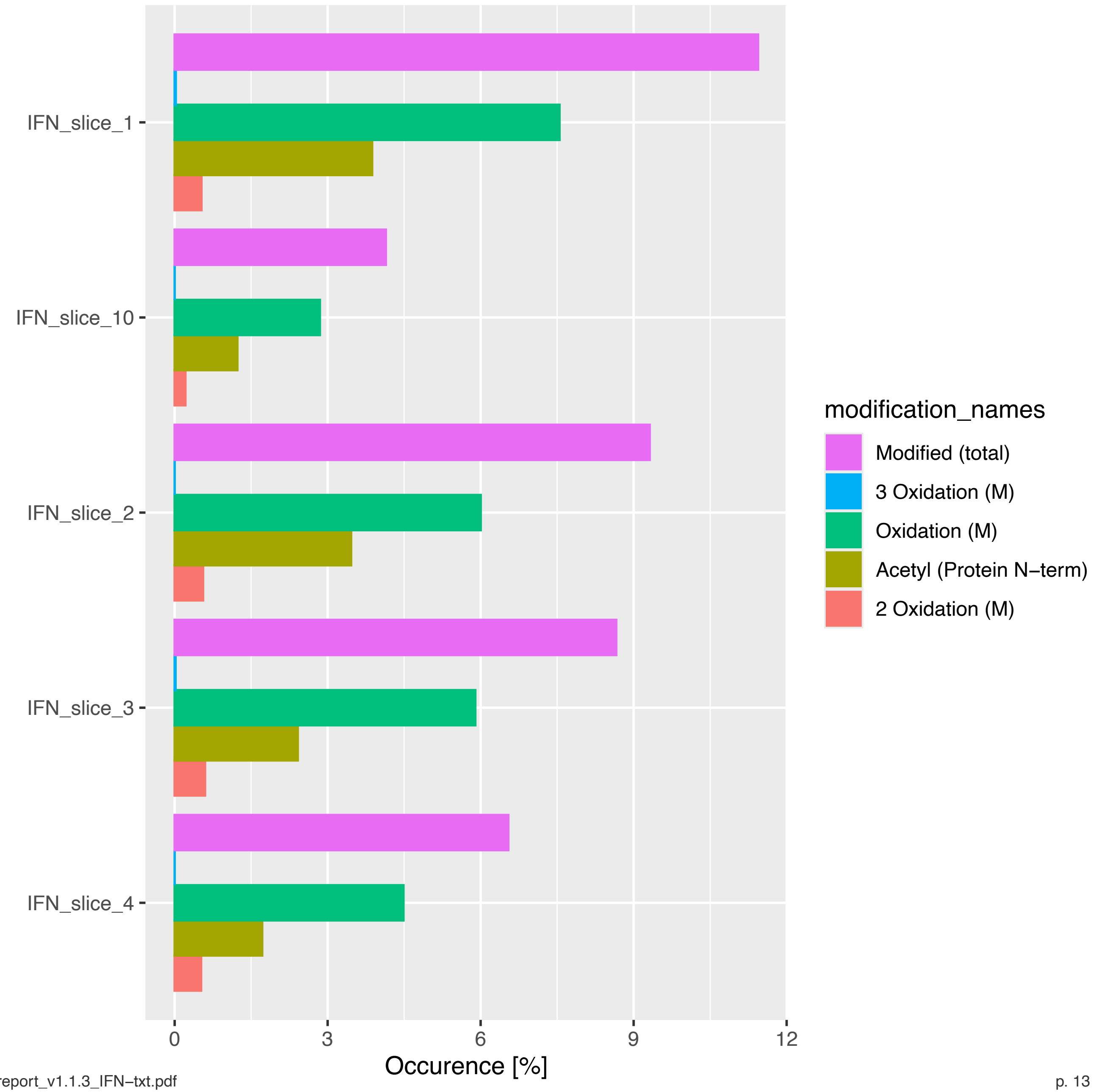

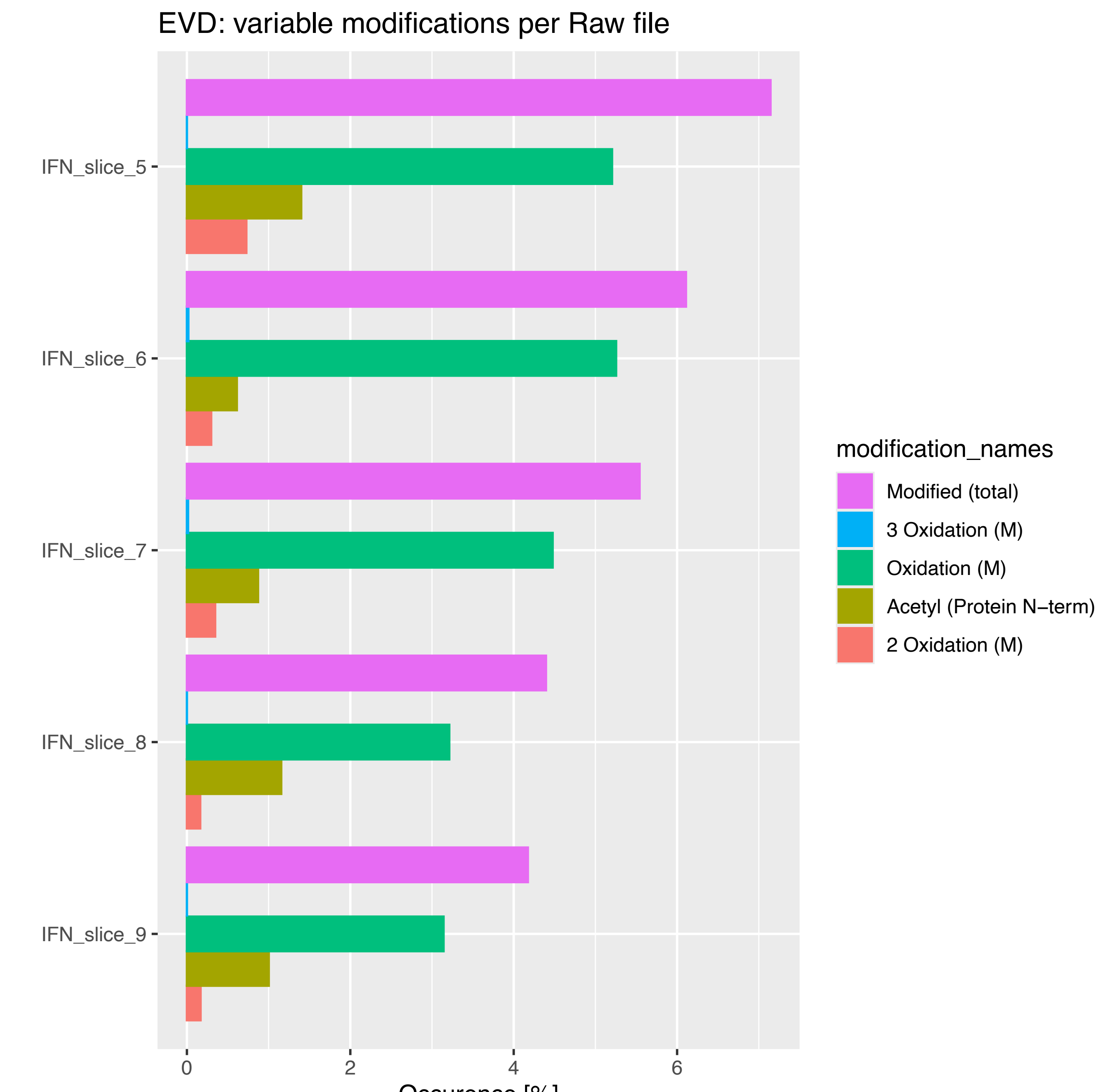
EVD: variable modifications per Raw file
IFN_slice_5
IFN_slice_6
IFN_slice_7
IFN_slice_8
IFN_slice_9
0
2
4
6
Occurence [%]
modification_names
Modified (total)
3 Oxidation (M)
Oxidation (M)
Acetyl (Protein N−term)
2 Oxidation (M)

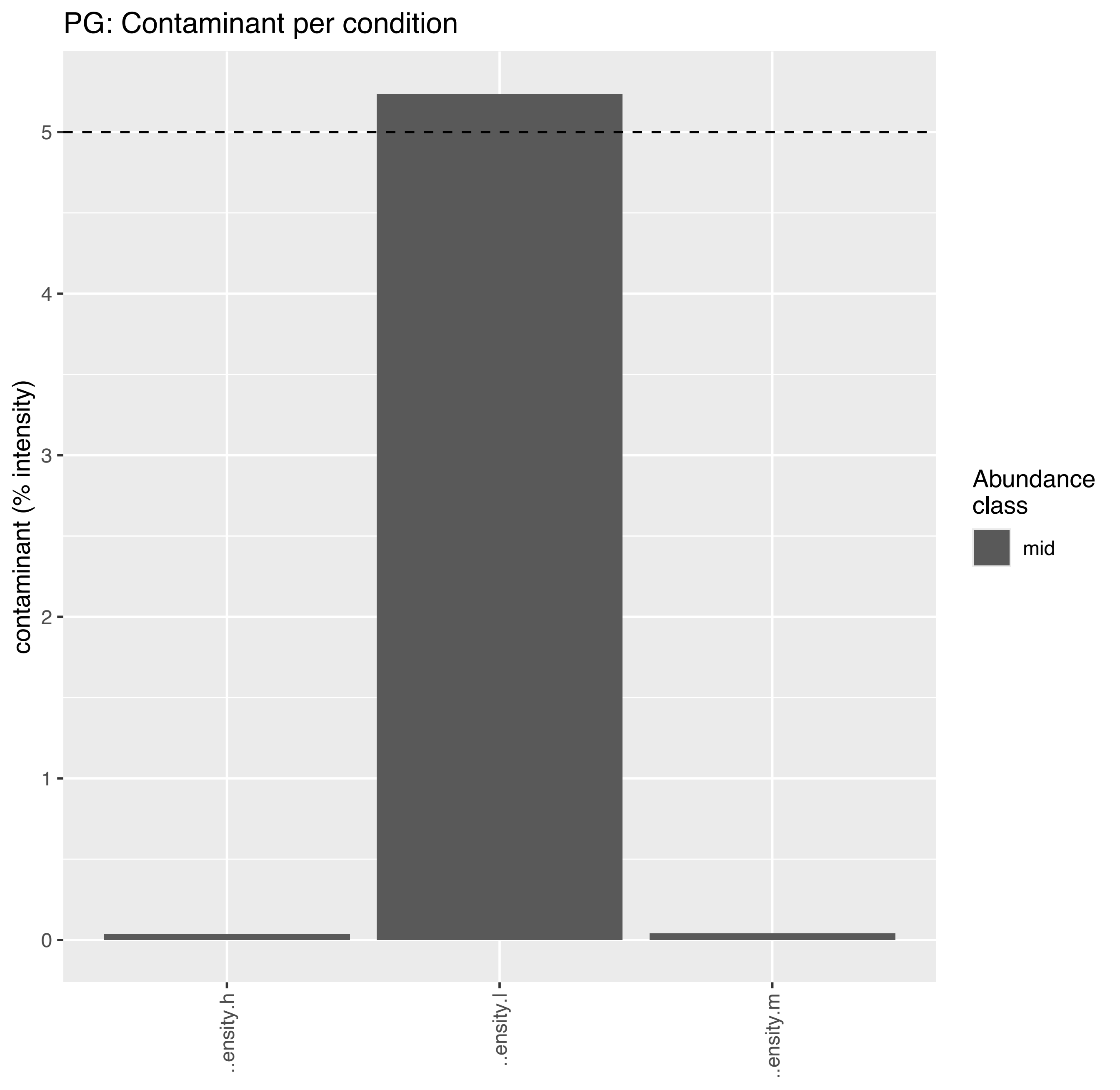
PG: Contaminant per condition
contaminant (% intensity)
0
1
2
3
4
5
..ensity.h
..ensity.l
..ensity.m
Abundance class
mid

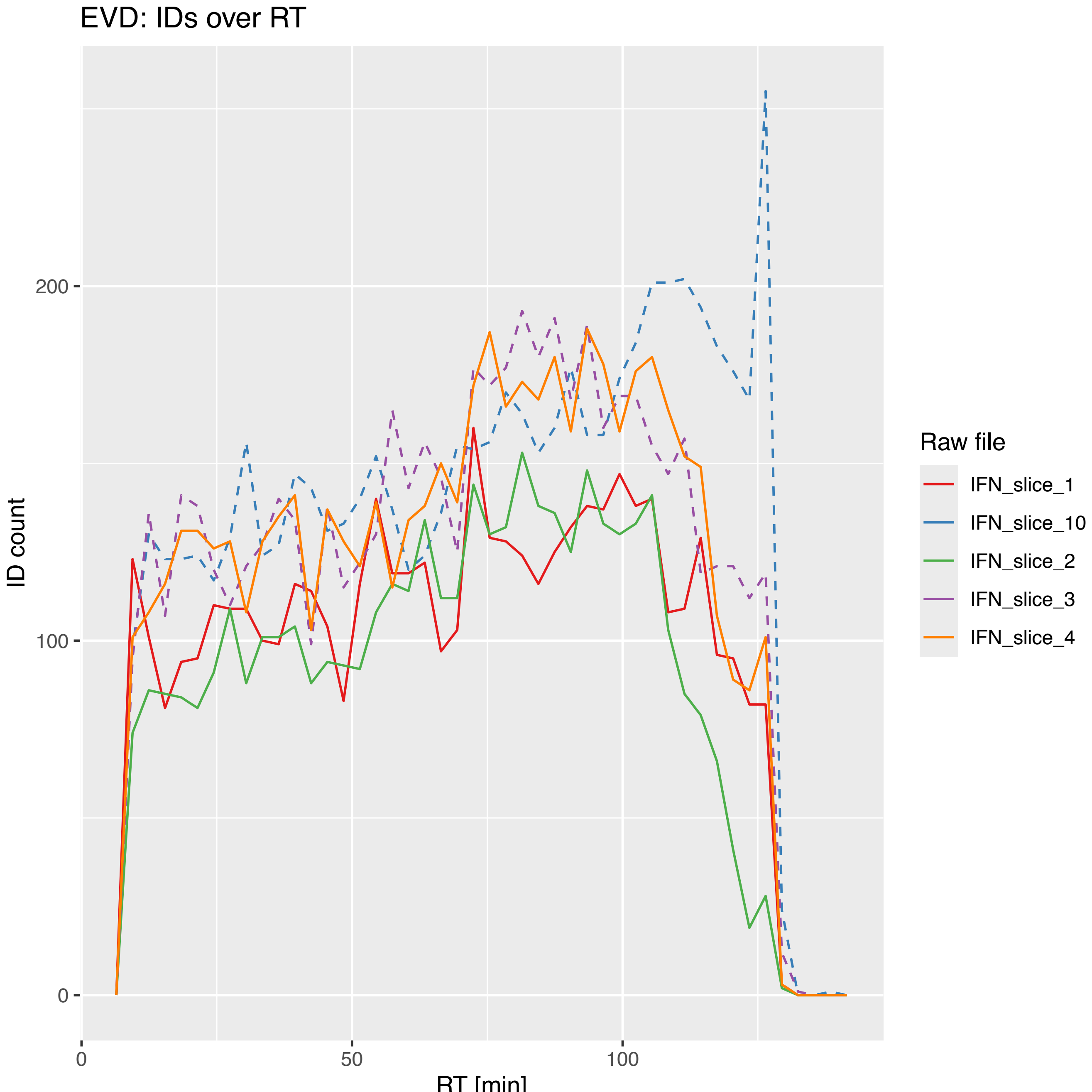
EVD: IDs over RT
ID count
200
100
0
0
50
100
RT [min]
Raw file
IFN_slice_1
IFN_slice_10
IFN_slice_2
IFN_slice_3
IFN_slice_4

EVD: IDs over RT

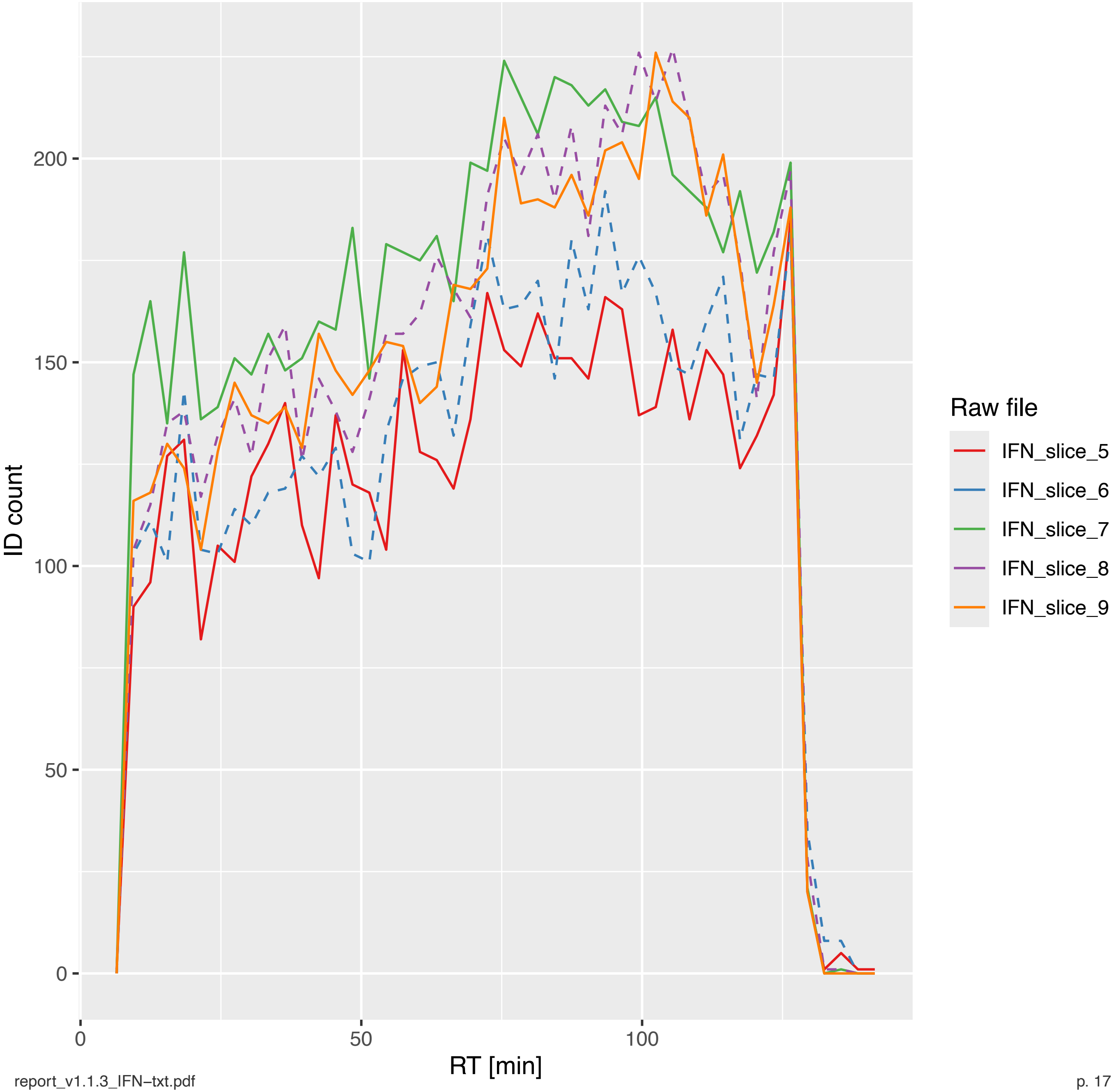

EVD: Peak width over RT

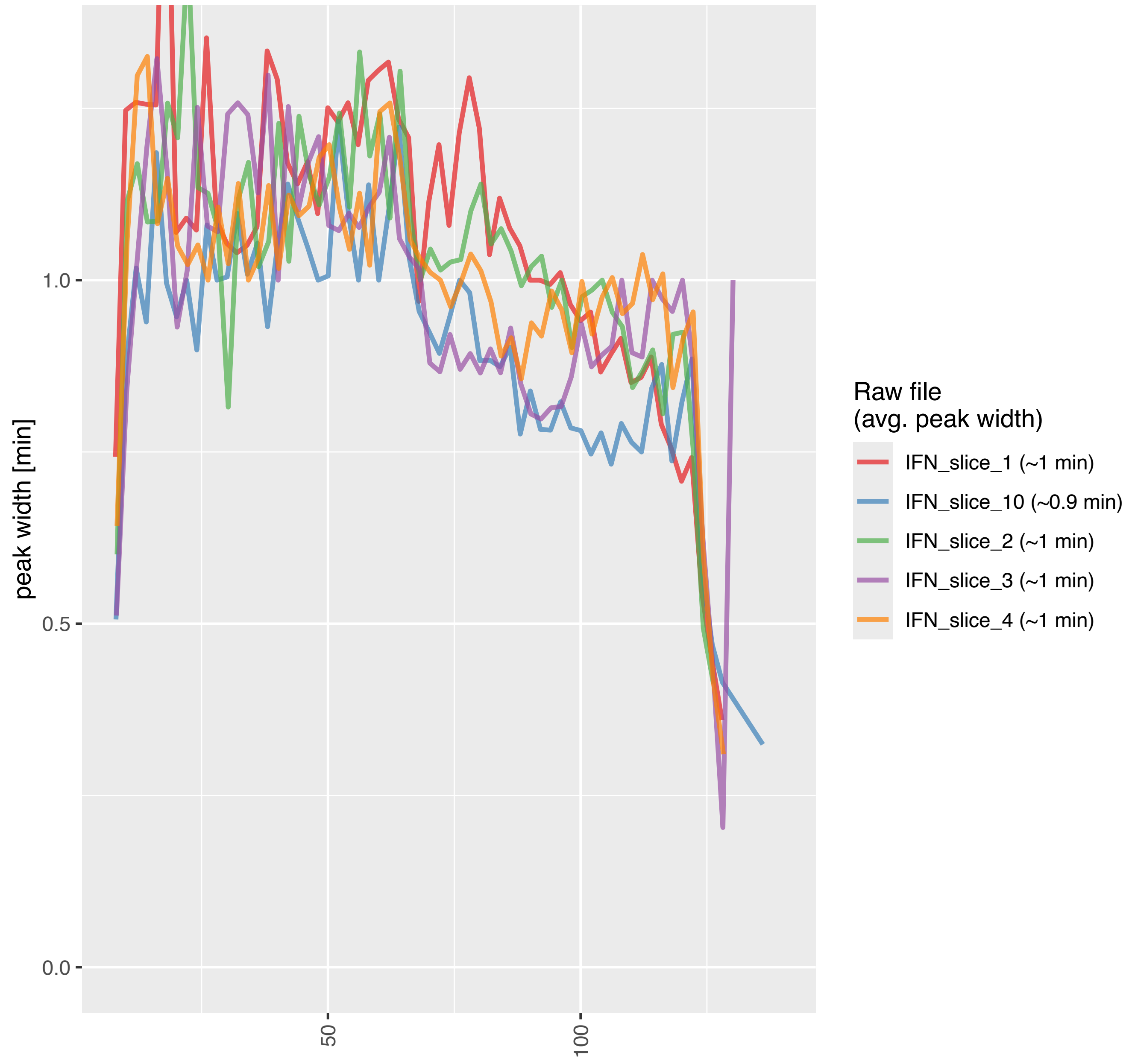

EVD: Peak width over RT

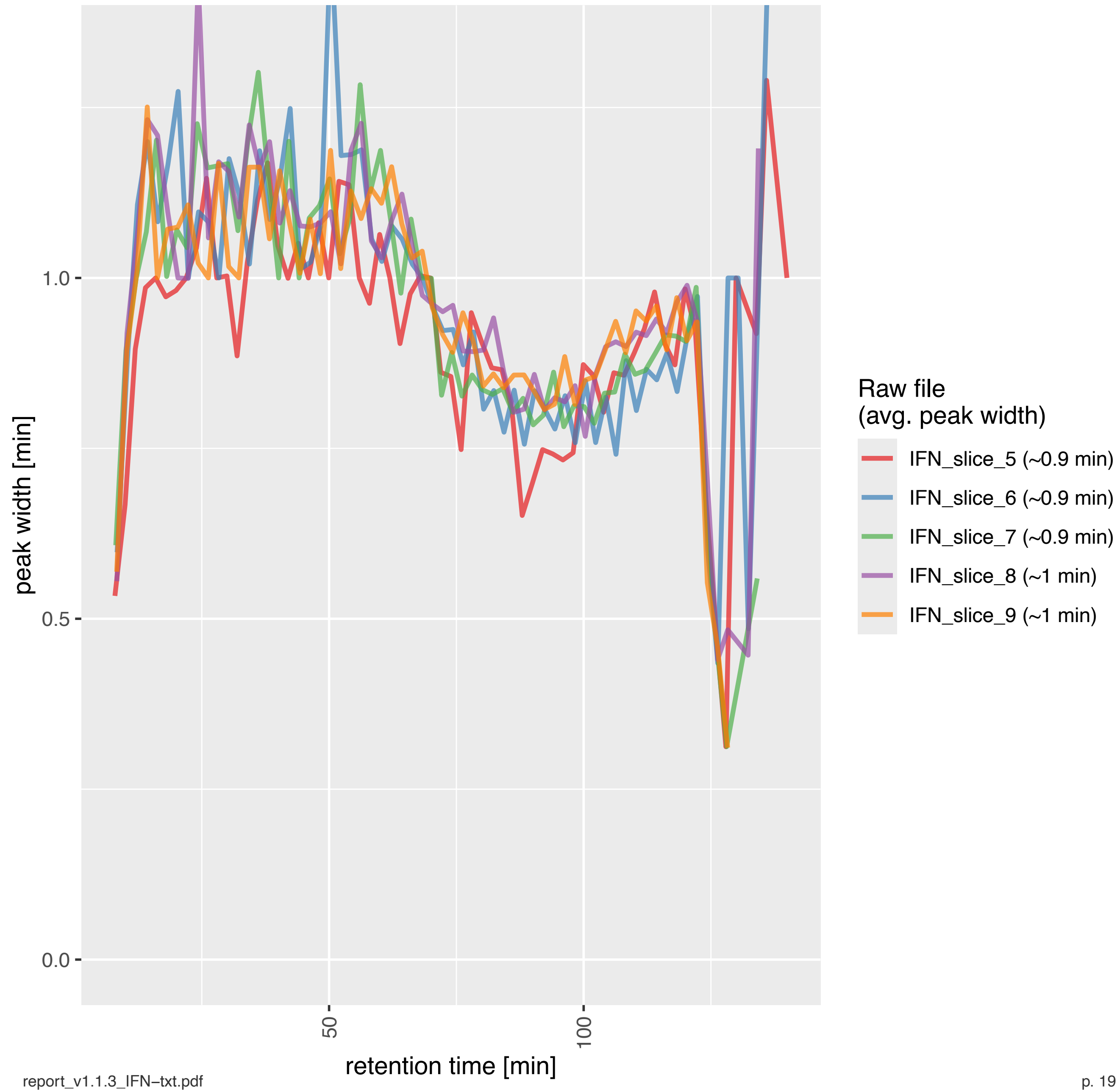

EVD: MBR – alignment

alignment reference: IFN_slice_1

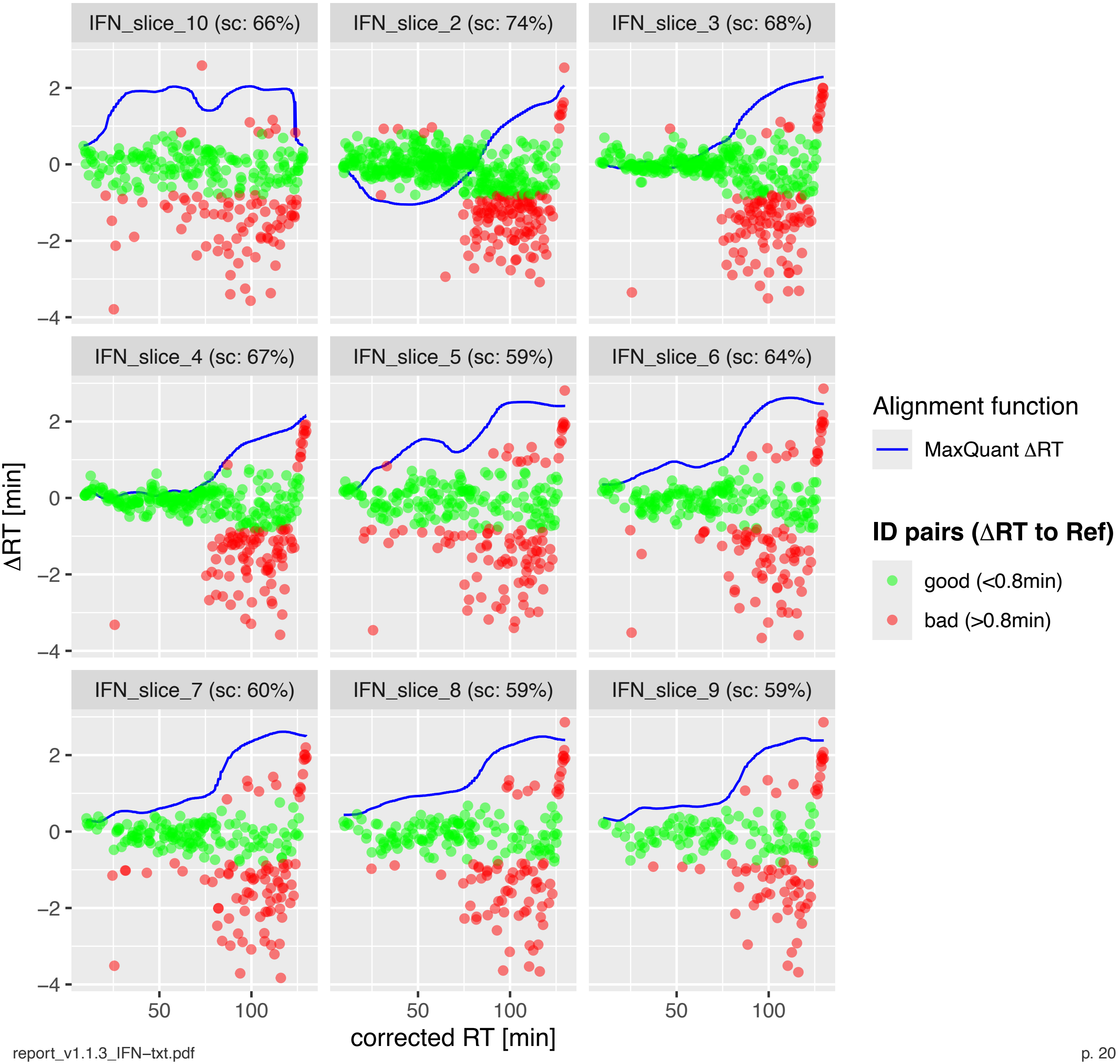

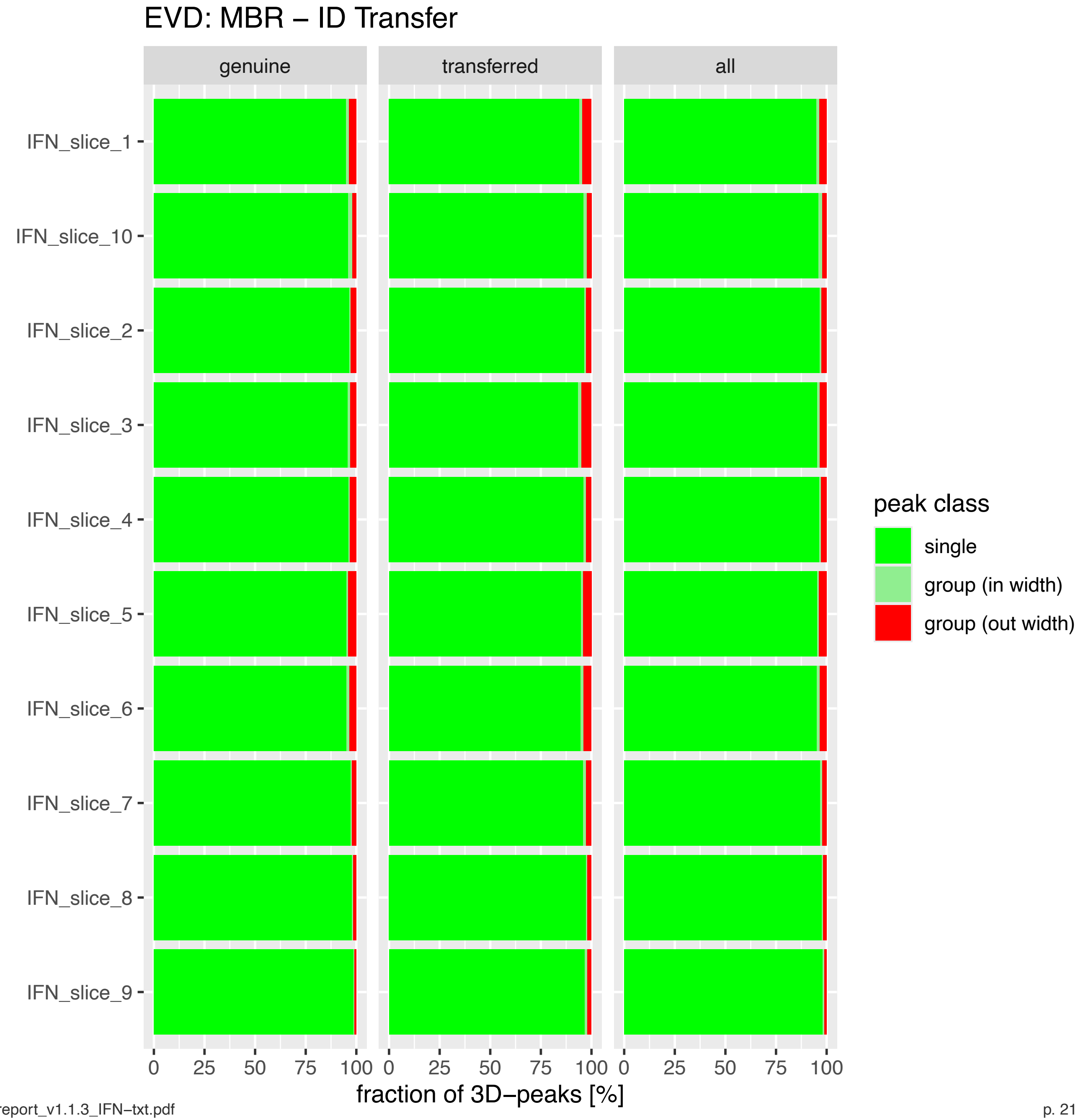

EVD: MBR – ID Transfer
genuine
transferred
all
IFN_slice_1
IFN_slice_10
IFN_slice_2
IFN_slice_3
IFN_slice_4
IFN_slice_5
IFN_slice_6
IFN_slice_7
IFN_slice_8
IFN_slice_9
0
25
50
75
100
fraction of 3D−peaks [%]
peak class
single
group (in width)
group (out width)

[experimental] EVD: Clustering Tree of Raw files

by Correlation of Corrected Retention Times

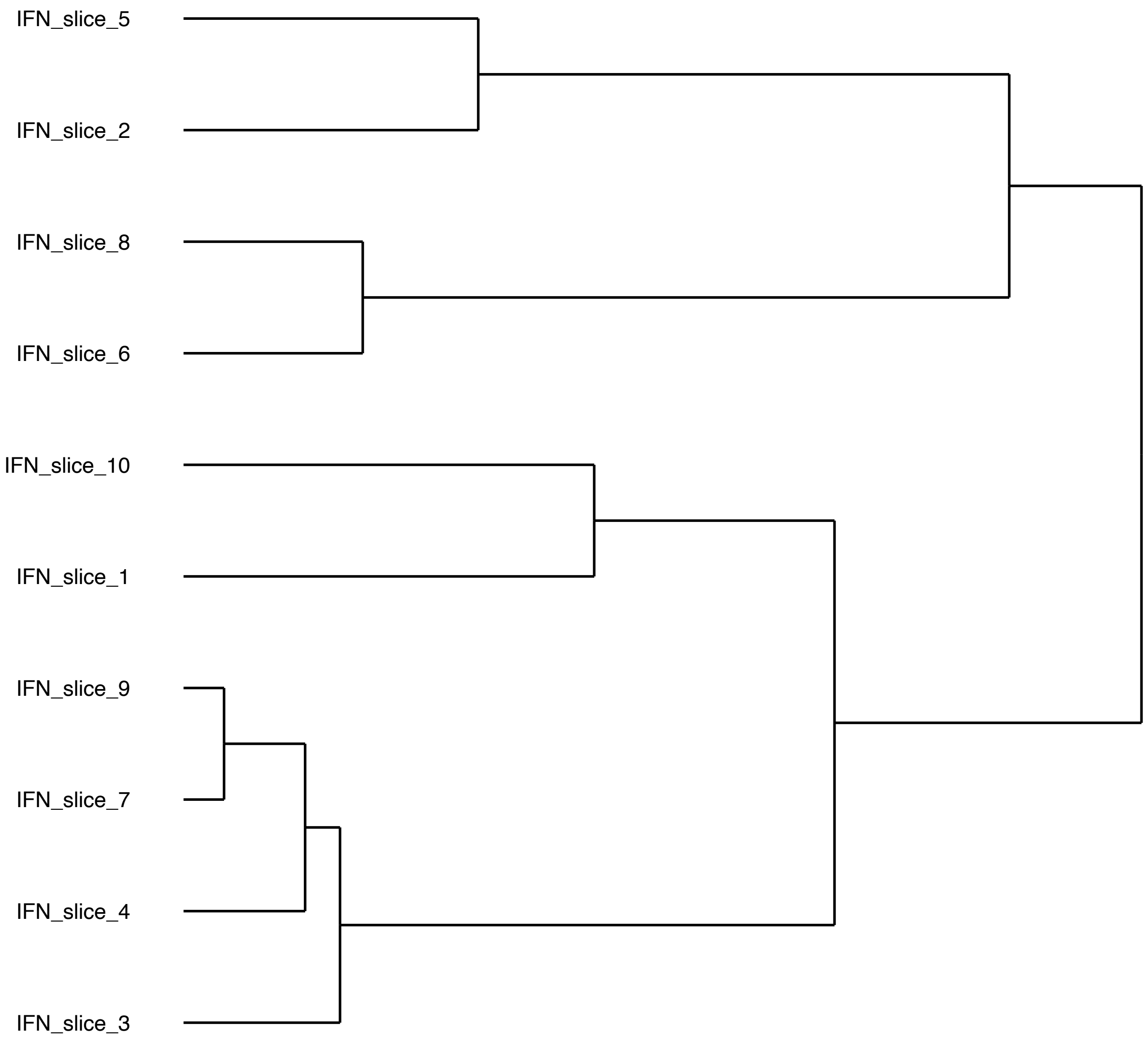

EVD: Peptides inferred by MBR

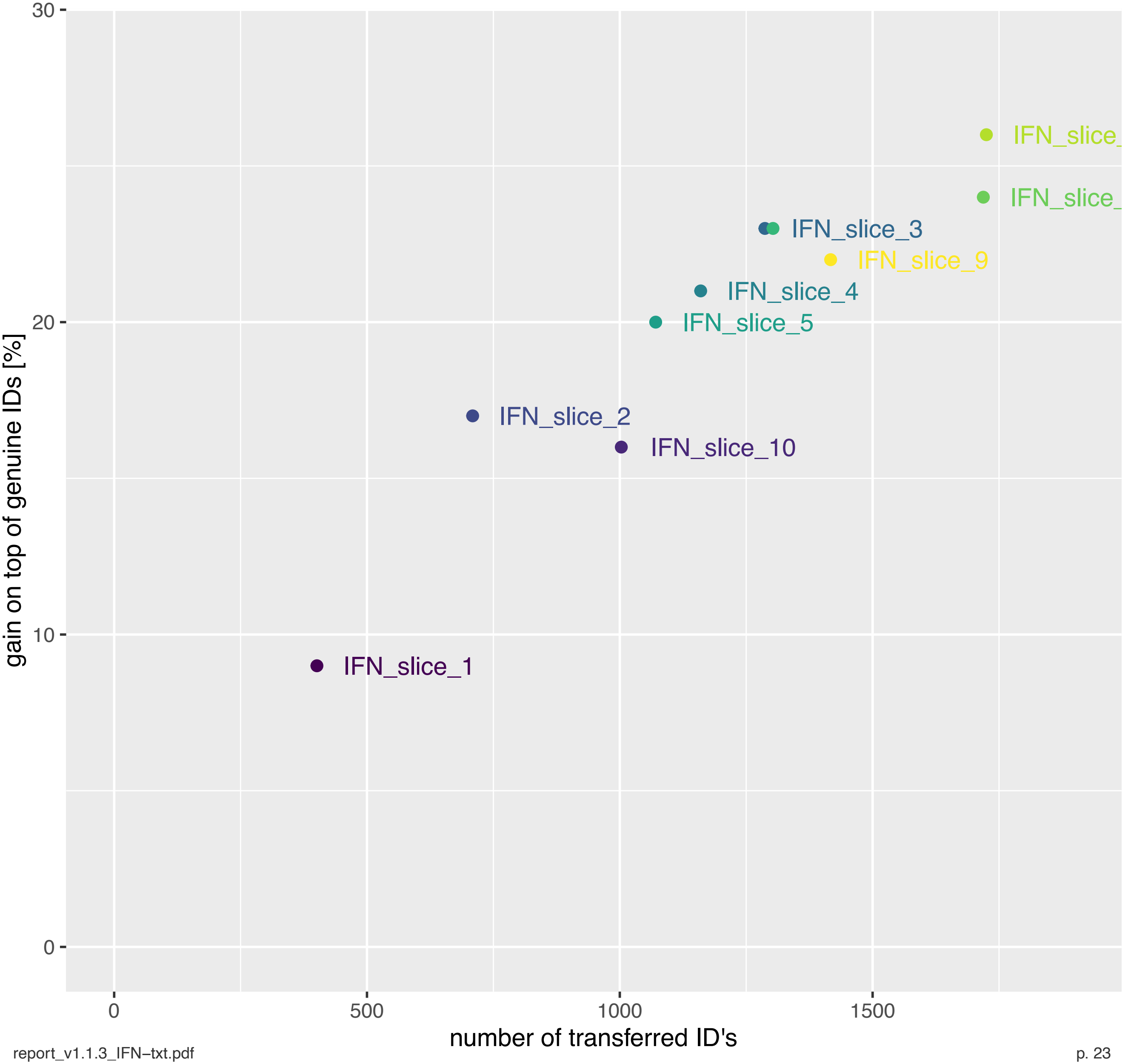

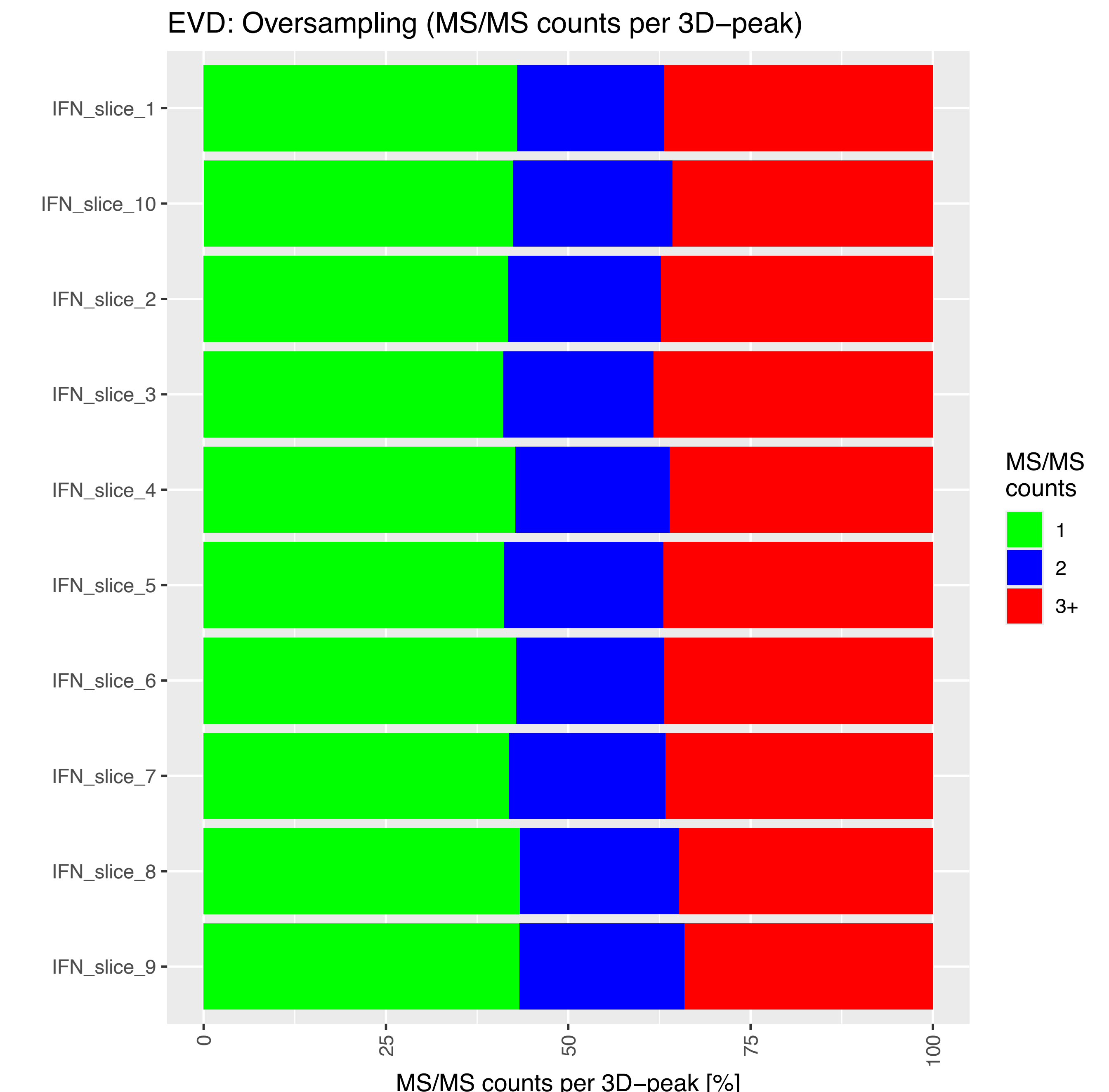
EVD: Oversampling (MS/MS counts per 3D−peak)
IFN_slice_1
IFN_slice_10
IFN_slice_2
IFN_slice_3
IFN_slice_4
IFN_slice_5
IFN_slice_6
IFN_slice_7
IFN_slice_8
IFN_slice_9
0
25
50
75
100
MS/MS counts per 3D−peak [%]
MS/MS counts
1
2
3+

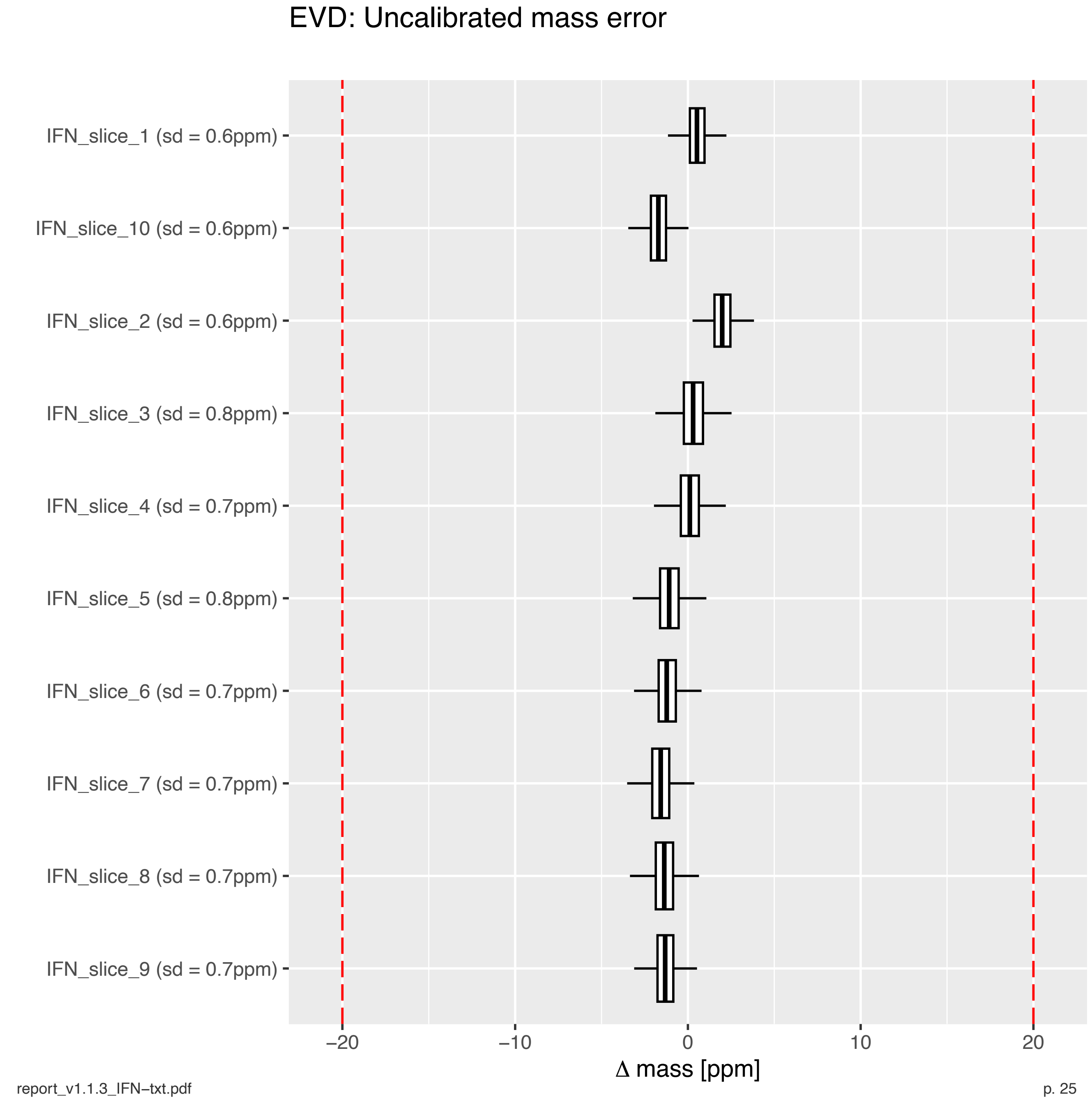
EVD: Uncalibrated mass error
IFN_slice_1 (sd = 0.6ppm)
IFN_slice_10 (sd = 0.6ppm)
IFN_slice_2 (sd = 0.6ppm)
IFN_slice_3 (sd = 0.8ppm)
IFN_slice_4 (sd = 0.7ppm)
IFN_slice_5 (sd = 0.8ppm)
IFN_slice_6 (sd = 0.7ppm)
IFN_slice_7 (sd = 0.7ppm)
IFN_slice_8 (sd = 0.7ppm)
IFN_slice_9 (sd = 0.7ppm)
−20
−10
0
10
20
Δ mass [ppm]

EVD: Calibrated mass error

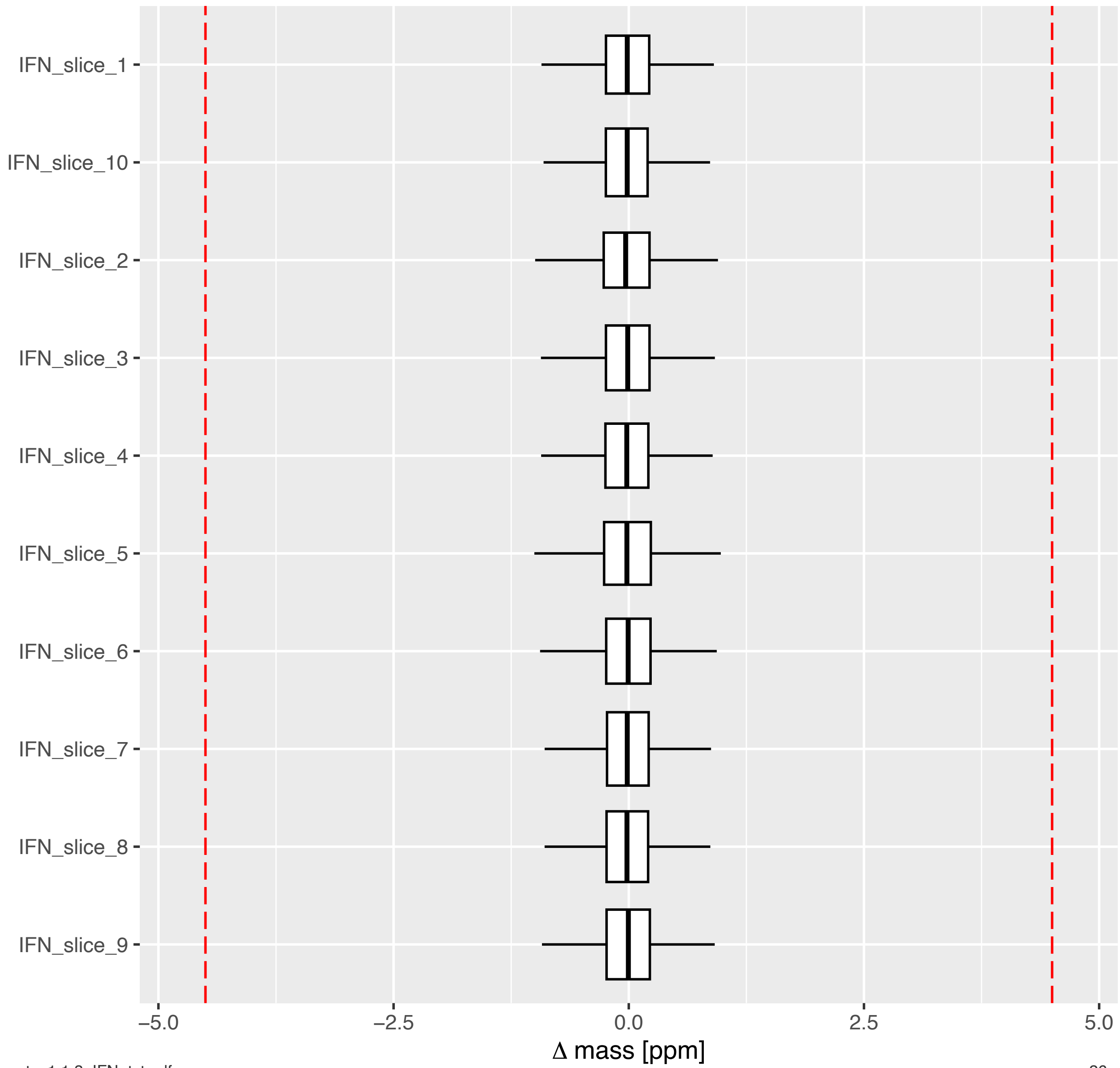

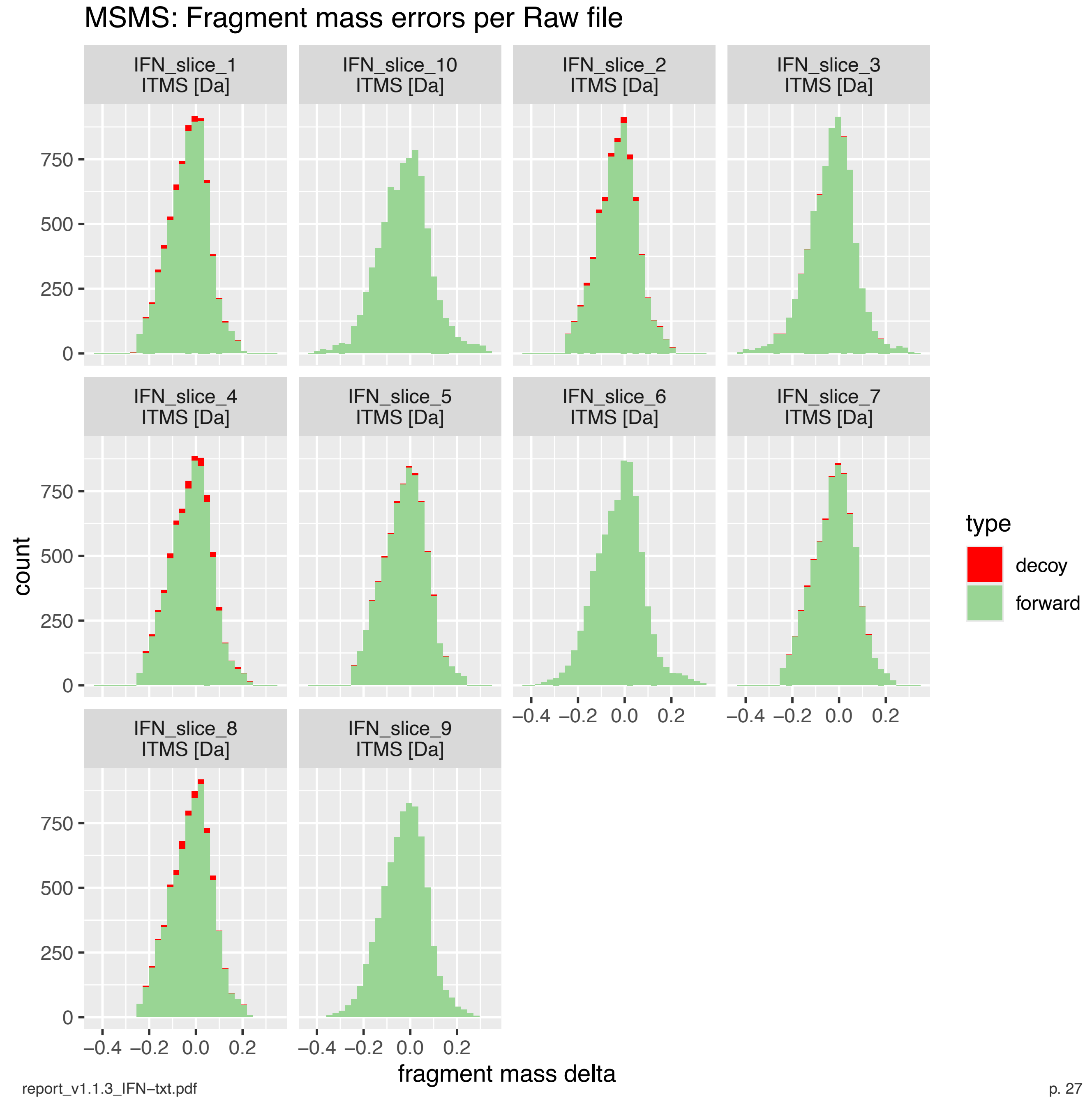

MSMS: Fragment mass errors per Raw file
IFN_slice_1
ITMS [Da]
IFN_slice_10
ITMS [Da]
IFN_slice_2
ITMS [Da]
IFN_slice_3
ITMS [Da]
IFN_slice_4
ITMS [Da]
IFN_slice_5
ITMS [Da]
IFN_slice_6
ITMS [Da]
IFN_slice_7
ITMS [Da]
IFN_slice_8
ITMS [Da]
IFN_slice_9
ITMS [Da]
count
0
250
500
750
−0.4
−0.2
0.0
0.2
fragment mass delta
type
decoy
forward

SM: MS/MS identified per Raw file

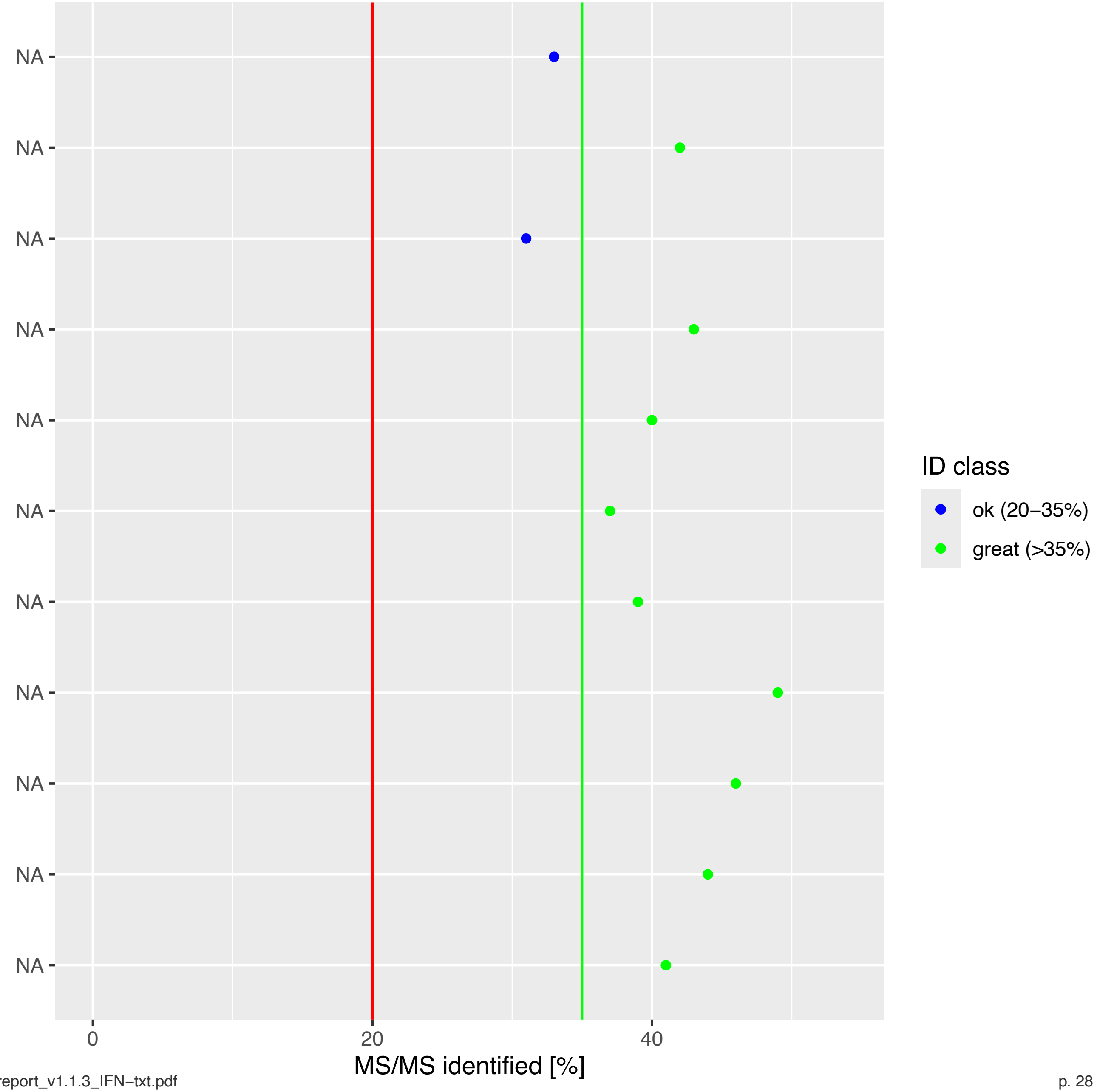

[experimental] EVD: Non−Missing Peptides

compared to all peptides seen in experiment

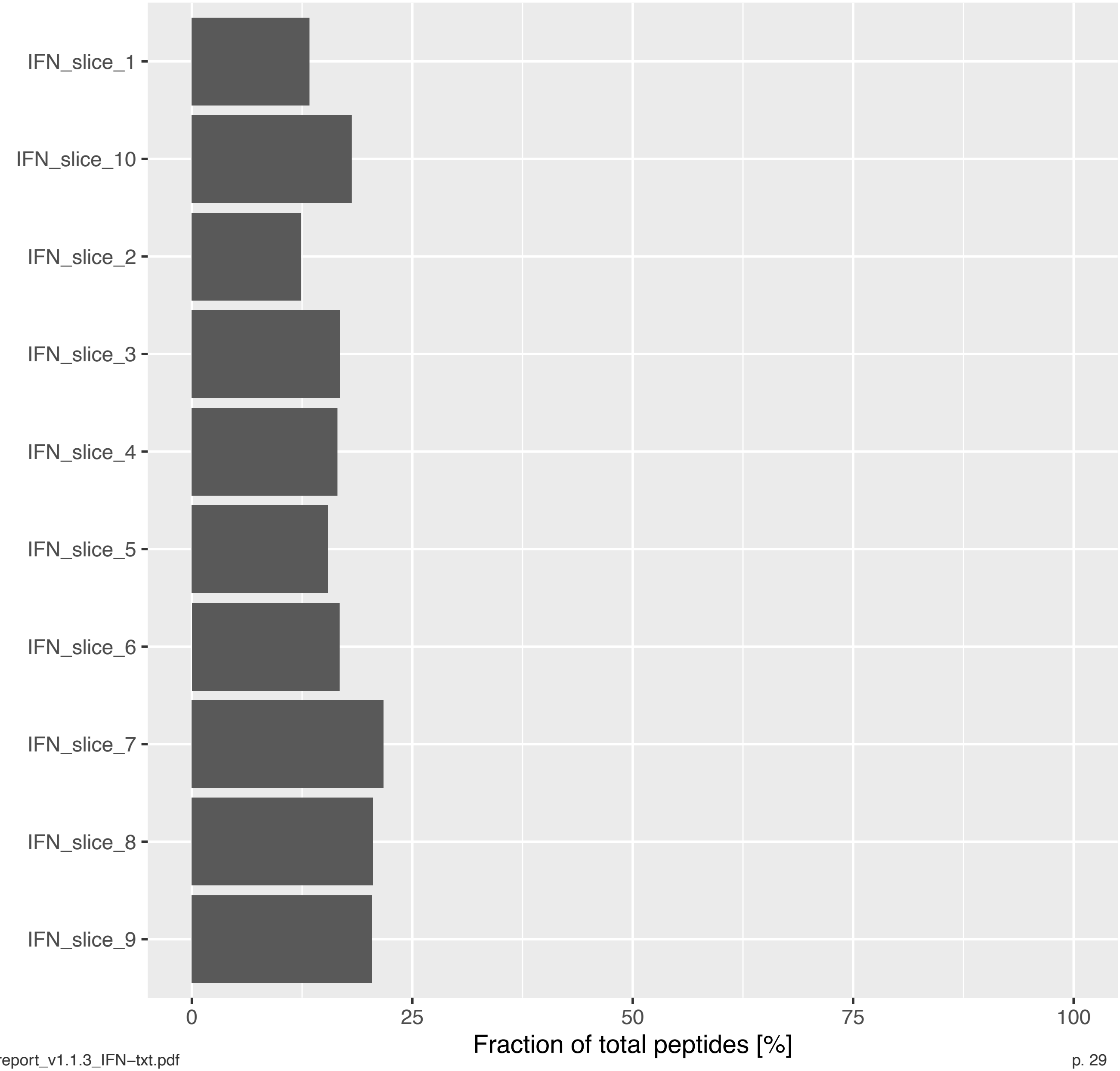

[experimental] EVD: Non−missing by set

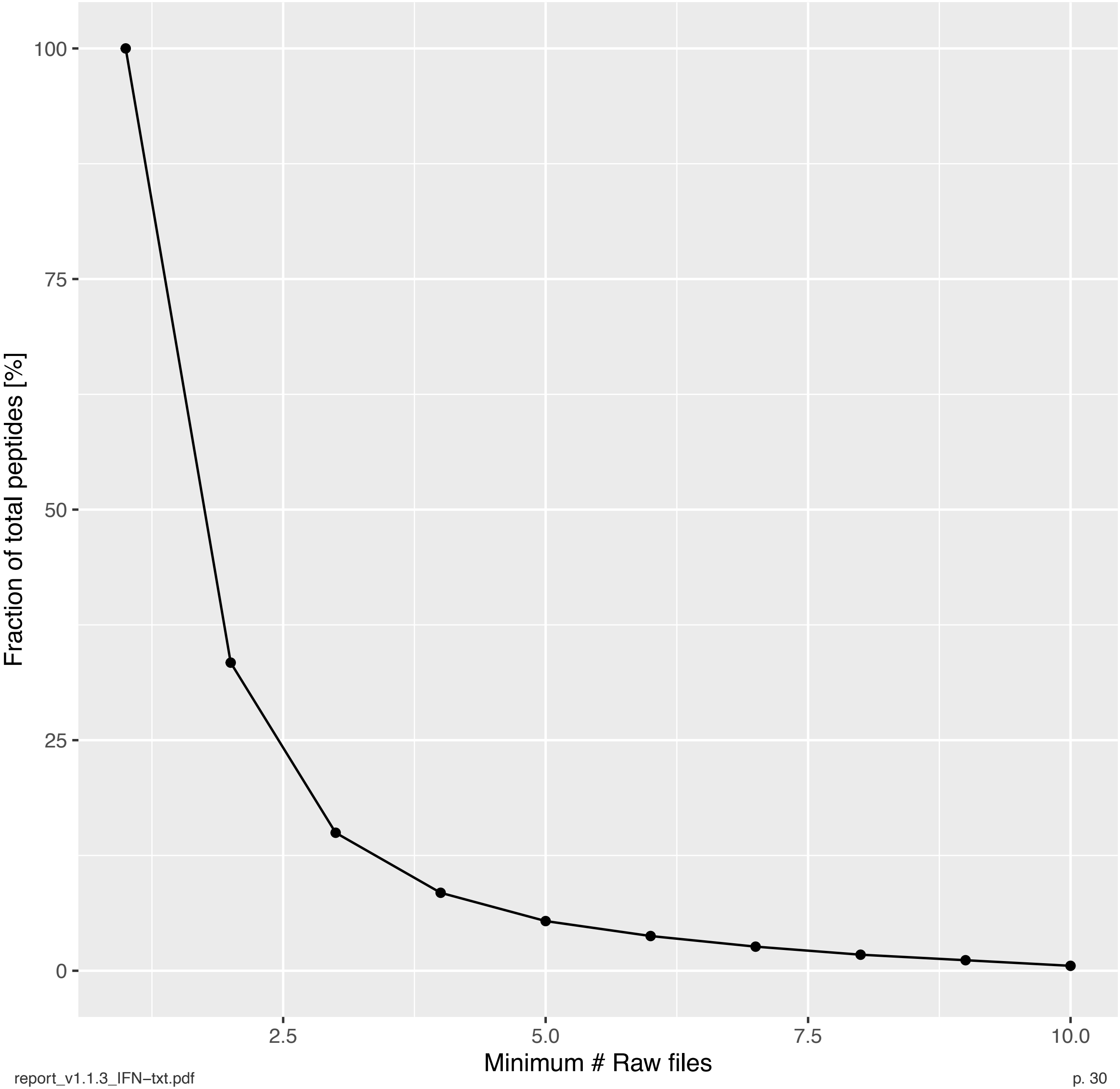

[experimental] EVD: Imputed Peptide Intensity Distribution of Missing Values

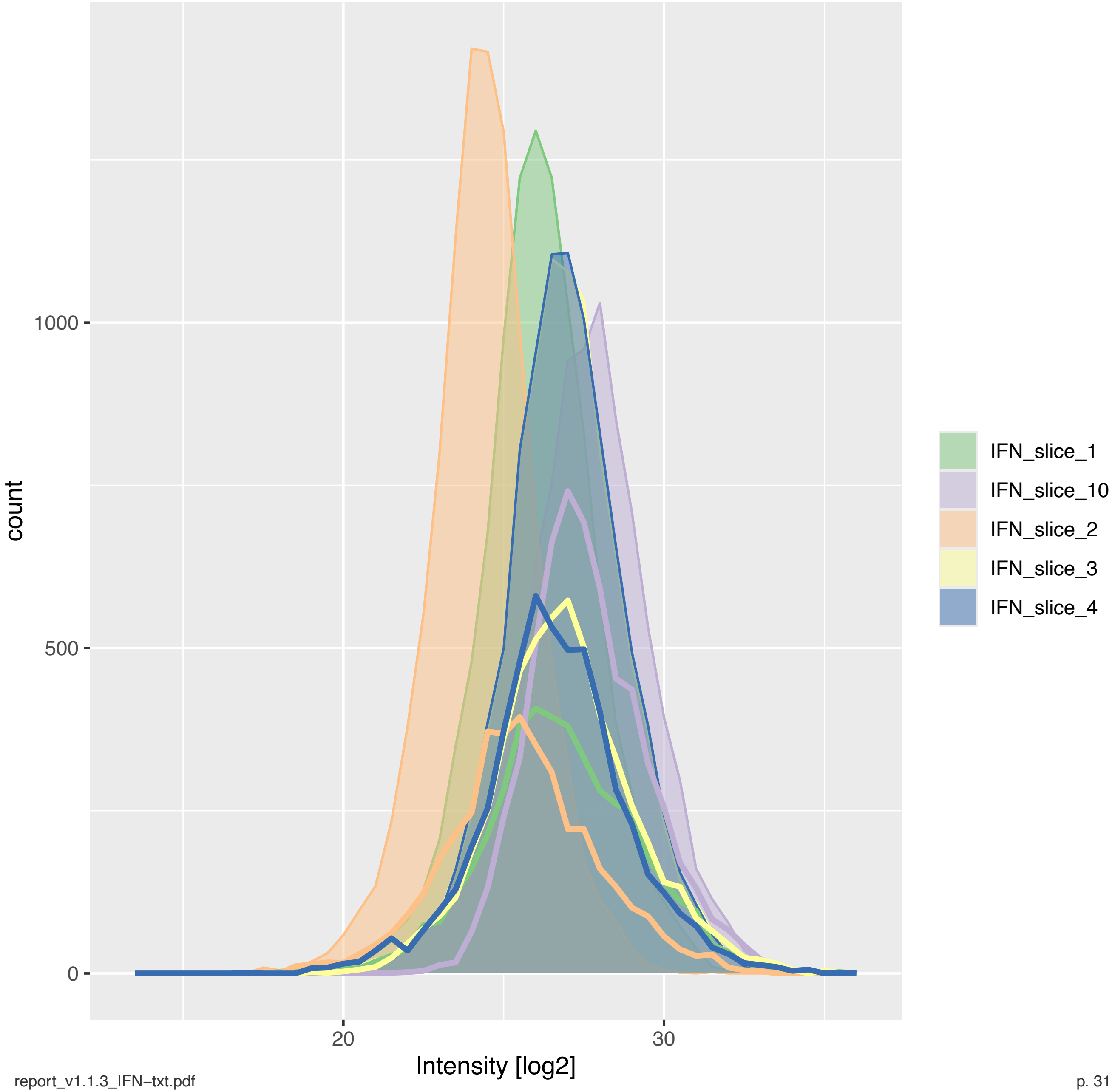

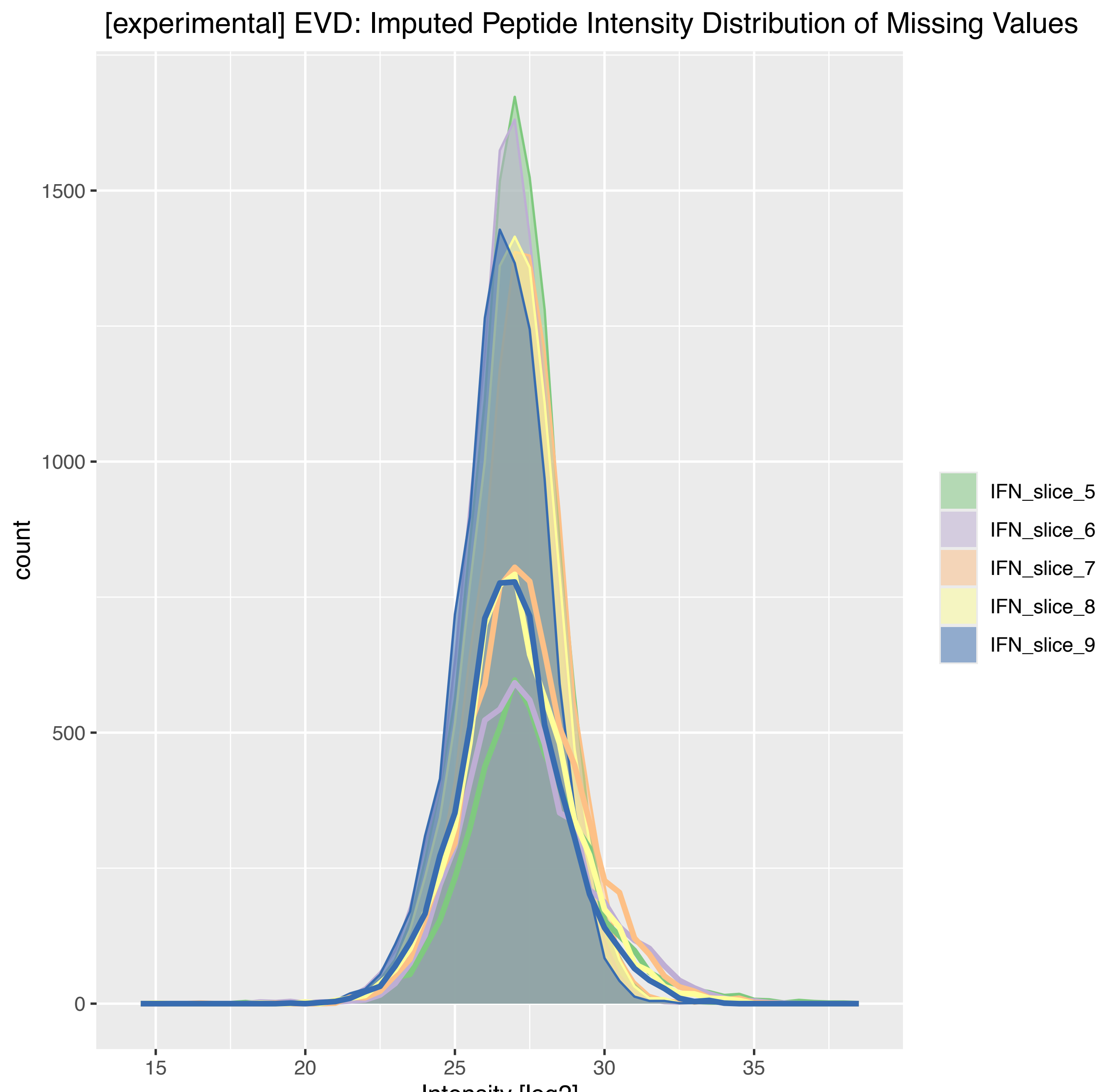
[experimental] EVD: Imputed Peptide Intensity Distribution of Missing Values
count
1500
1000
500
0
15
20
25
30
35
Intensity [log2]
IFN_slice_5
IFN_slice_6
IFN_slice_7
IFN_slice_8
IFN_slice_9

EVD: Peptide ID count

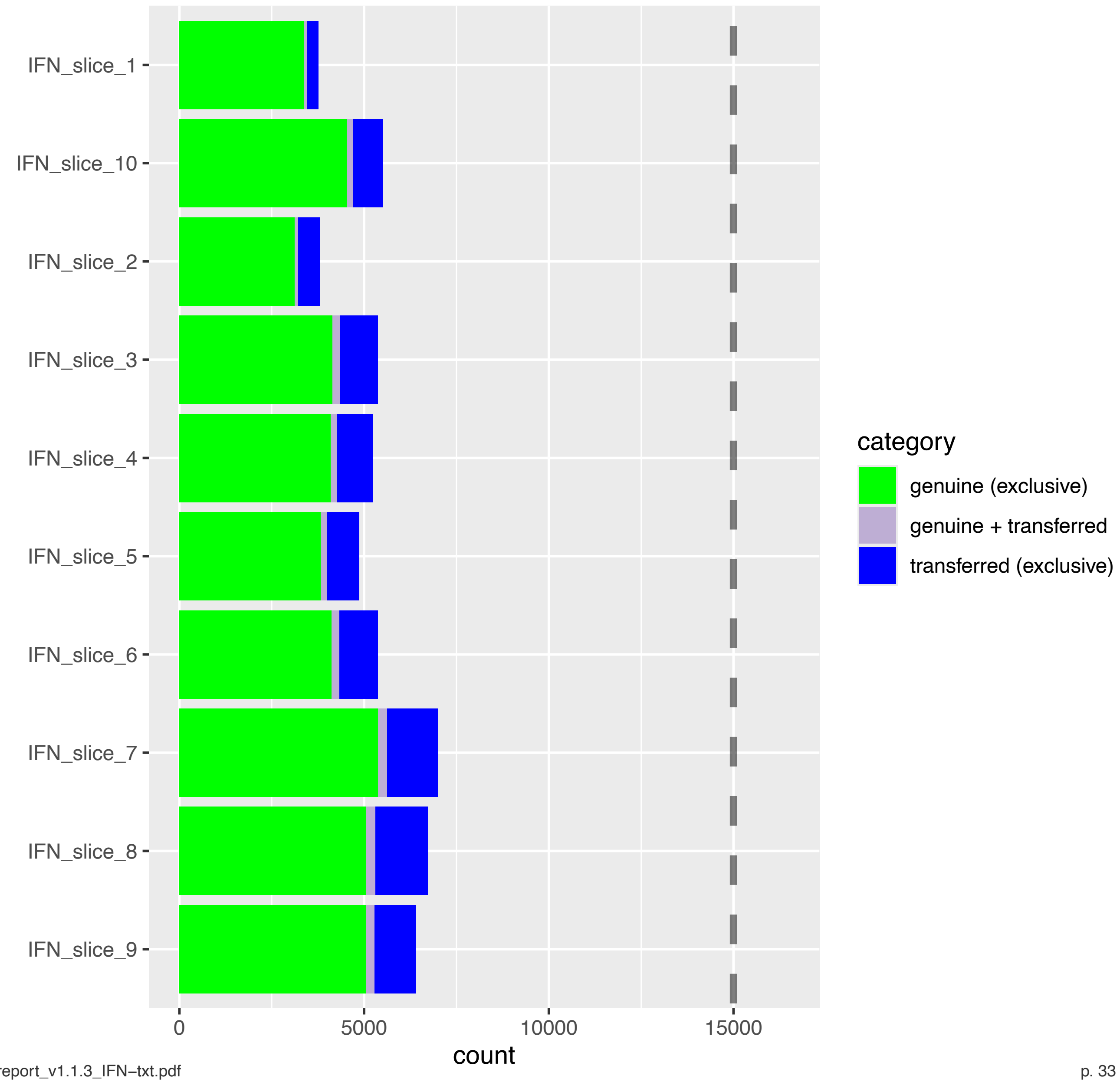

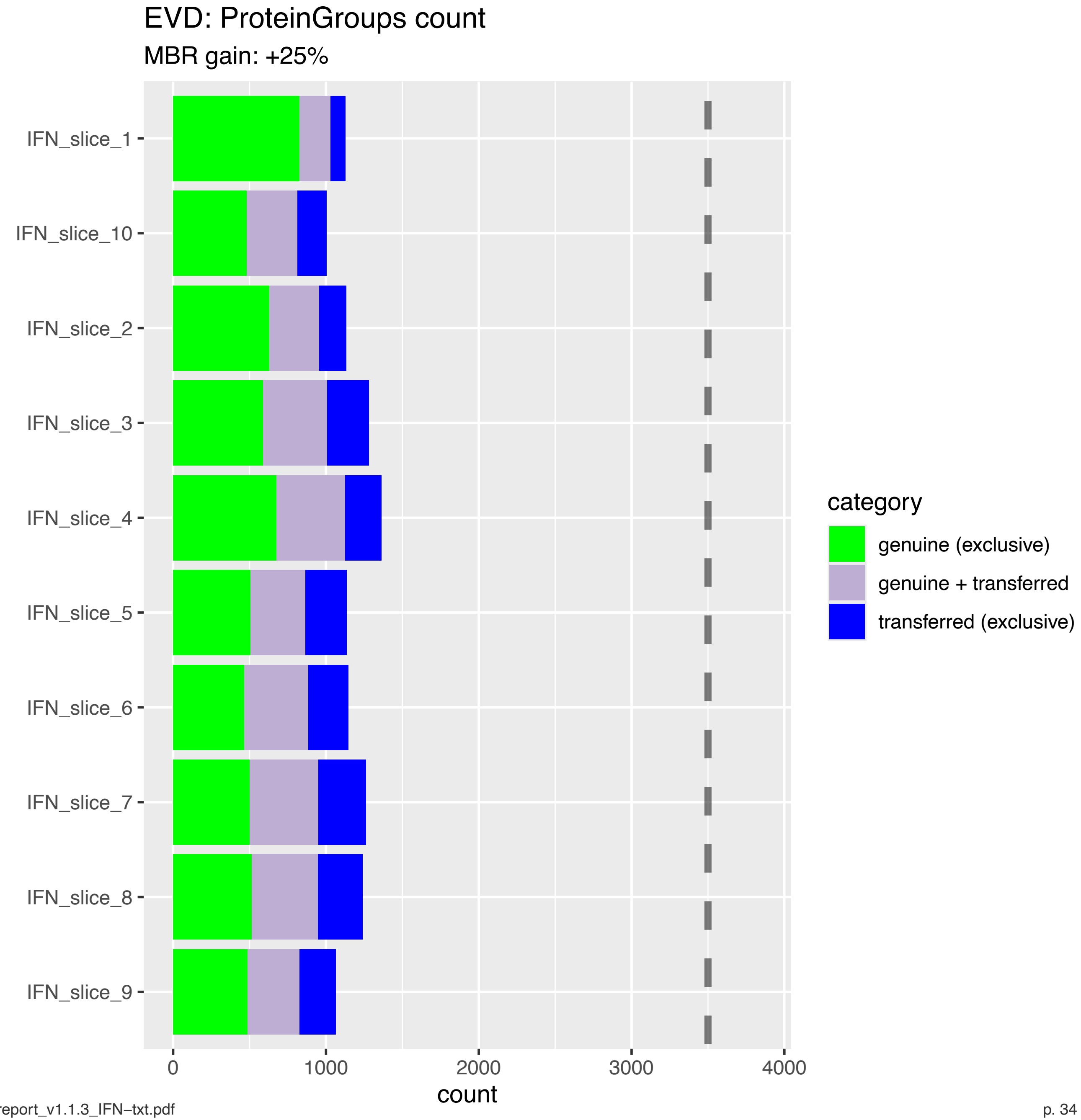

EVD: ProteinGroups count
MBR gain: +25%
IFN_slice_1
IFN_slice_10
IFN_slice_2
IFN_slice_3
IFN_slice_4
IFN_slice_5
IFN_slice_6
IFN_slice_7
IFN_slice_8
IFN_slice_9
0
1000
2000
3000
4000
count
category
genuine (exclusive)
genuine + transferred
transferred (exclusive)

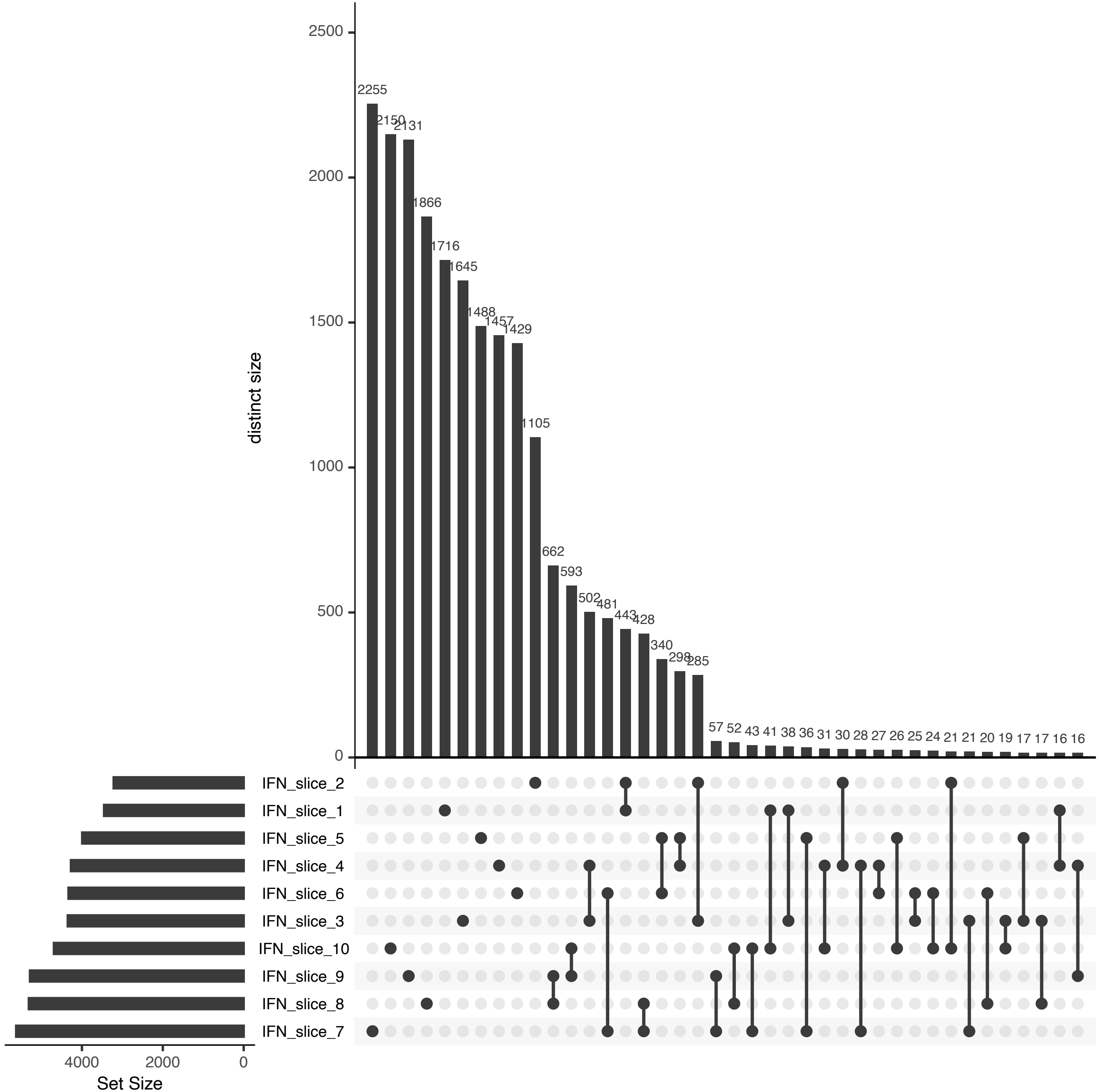

distinct size
2500
2000
1500
1000
500
0
2255
2150
2131
1866
1716
1645
1488
1457
1429
1105
662
593
502
481
443
428
340
298
285
57
52
43
41
38
36
31
30
28
27
26
25
24
21
21
20
19
17
17
16
16
IFN_slice_2
IFN_slice_1
IFN_slice_5
IFN_slice_4
IFN_slice_6
IFN_slice_3
IFN_slice_10
IFN_slice_9
IFN_slice_8
IFN_slice_7
4000
2000
0
Set Size

pIpC

Performance overview

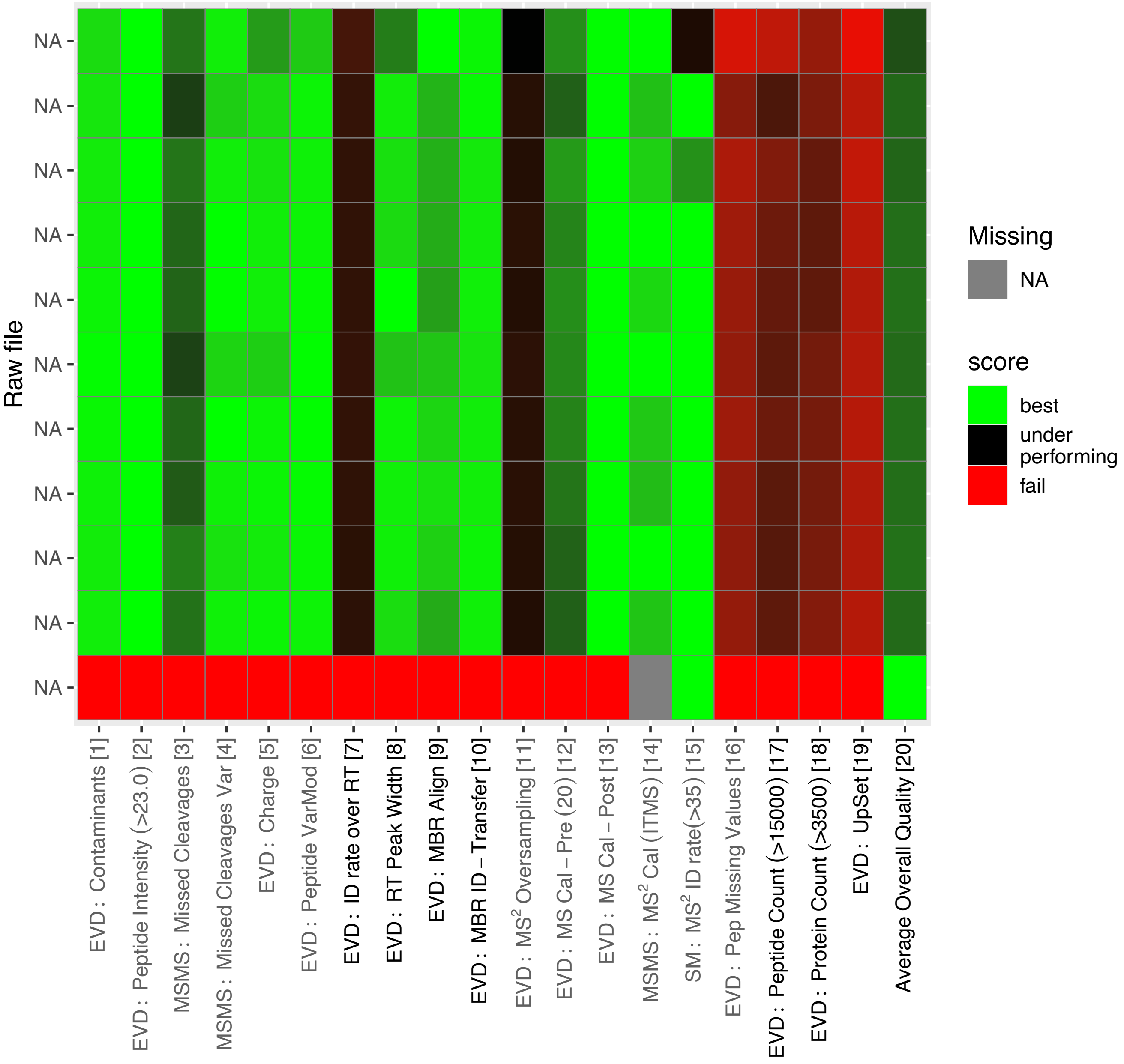

Mapping of Raw files to their short names
Mapping source: automatic

original	short name	best effort
plpC_slice_1	plpC_slice_1	plpC_slice_1
plpC_slice_10	plpC_slice_10	plpC_slice_10
plpC_slice_2	plpC_slice_2	plpC_slice_2
plpC_slice_3	plpC_slice_3	plpC_slice_3
plpC_slice_4	plpC_slice_4	plpC_slice_4
plpC_slice_5	plpC_slice_5	plpC_slice_5
plpC_slice_6	plpC_slice_6	plpC_slice_6
plpC_slice_7	plpC_slice_7	plpC_slice_7
plpC_slice_8	plpC_slice_8	plpC_slice_8
plpC_slice_9	plpC_slice_9	plpC_slice_9
Total	Total	Total

PAR: parameters

parameter	value	parameter	value
Advanced ratios	True	MS/MS deisotoping tolerance ..	7
Advanced site intensities	True	MS/MS deisotoping tolerance ..	ppm
Alignment ion mobility windo..	1	MS/MS dependent losses (ASTR..	True
Alignment time window [min]	20	MS/MS dependent losses (FTMS..	True
Calculate peak properties	False	MS/MS dependent losses (ITMS..	True
Da interval. (ASTRAL)	100	MS/MS dependent losses (TOF)	True
Da interval. (FTMS)	100	MS/MS dependent losses (UNKN..	True
Da interval. (ITMS)	100	MS/MS higher charges (ASTRAL..	True
Da interval. (TOF)	100	MS/MS higher charges (FTMS)	True
Da interval. (UNKNOWN)	100	MS/MS higher charges (ITMS)	True
Date of writing	04/12/2025 04:26:39	MS/MS higher charges (TOF)	True
Decoy mode	revert	MS/MS higher charges (UNKNOW..	True
Disable MD5	False	MS/MS recalibration (ASTRAL)	False
Discard unmodified counterpa..	True	MS/MS recalibration (FTMS)	False
Epsilon score for mutations	True	MS/MS recalibration (ITMS)	False
Evaluate variant peptides se..	True	MS/MS recalibration (TOF)	False
Find dependent peptides	False	MS/MS recalibration (UNKNOWN..	False
Fixed andromeda index folder		MS/MS tol. (ASTRAL)	25 ppm
iBAQ	True	MS/MS tol. (FTMS)	20 ppm
iBAQ log fit	True	MS/MS tol. (ITMS)	0.5 Da
Include contaminants	True	MS/MS tol. (TOF)	25 ppm
Label min. ratio count	2	MS/MS tol. (UNKNOWN)	20 ppm

protein.faa

PAR: parameters

parameter	value	parameter	value
Machine name	DESKTOP−SUMVKIL	MS/MS water loss (ASTRAL for..	False
Main search max. combination..	200	MS/MS water loss (ASTRAL)	True
Match between runs	True	MS/MS water loss (FTMS for c..	False
Match ion mobility window [i..	0.05	MS/MS water loss (FTMS)	True
Match unidentified features	True	MS/MS water loss (ITMS for c..	False
Matching time window [min]	0.8	MS/MS water loss (ITMS)	True
Max mods in site table	3	MS/MS water loss (TOF for cr..	False
Max. peptide length for unsp..	25	MS/MS water loss (TOF)	True
Max. peptide mass [Da]	4600	MS/MS water loss (UNKNOWN fo..	False
Min. delta score for modifie..	6	MS/MS water loss (UNKNOWN)	True
Min. delta score for unmodif..	0	Peptides used for protein qu..	Razor
Min. peptide Length	7	Protein FDR	0.01
Min. peptide length for unsp..	8	PSM FDR	0.01
Min. peptides	1	PSM FDR Crosslink	0.01
Min. razor peptides	1	Razor protein FDR	True
Min. score for modified pept..	40	Require MS/MS for LFQ compar..	True
Min. score for unmodified pe..	0	Second peptides	True
Min. unique peptides	0	Separate LFQ in parameter gr..	False
Modifications included in pr..	Oxidation (M)<br>Acetyl (Protein N−term)	Site FDR	0.01
MS/MS ammonia loss (ASTRAL f..	False	Site tables	Oxidation (M)Sites.txt
MS/MS ammonia loss (ASTRAL)	True	Stabilize large LFQ ratios	True
MS/MS ammonia loss (FTMS for..	False	Top MS/MS peaks per Da inter..	12

protein.faa

PAR: parameters

parameter	value	parameter	value
MS/MS ammonia loss (FTMS)	True	Top MS/MS peaks per Da inter..	8
MS/MS ammonia loss (ITMS for..	False	Top MS/MS peaks per Da inter..	16
MS/MS ammonia loss (ITMS)	True	Top MS/MS peaks per Da inter..	16
MS/MS ammonia loss (TOF for ..	False	Top MS/MS peaks per Da inter..	12
MS/MS ammonia loss (TOF)	True	Use delta score	False
MS/MS ammonia loss (UNKNOWN ..	False	Use Normalized Ratios For Oc..	True
MS/MS ammonia loss (UNKNOWN)	True	Use only unmodified peptides..	True
MS/MS deisotoping (ASTRAL)	True	User name	tosor
MS/MS deisotoping (FTMS)	True	Variation mode	None
MS/MS deisotoping (ITMS)	False	Version	2.6.5.0
MS/MS deisotoping (TOF)	True	Write accumulatedMsmsScans t..	False
MS/MS deisotoping (UNKNOWN)	True	Write allPeptides table	False
MS/MS deisotoping tolerance ..	7	Write DIA fragments quant ta..	False
MS/MS deisotoping tolerance ..	ppm	Write DIA fragments table	False
MS/MS deisotoping tolerance ..	0.15	Write ms3Scans table	False
MS/MS deisotoping tolerance ..	Da	Write msmsScans table	False
MS/MS deisotoping tolerance ..	0.01	Write msScans table	False
MS/MS deisotoping tolerance ..	Da	Write mzRange table	False
MS/MS deisotoping tolerance ..	0.01	Write pasefMsmsScans table	False
MS/MS deisotoping tolerance ..	Da		

protein.faa

PG: PCA of 'raw intensity'

(excludes contaminants)

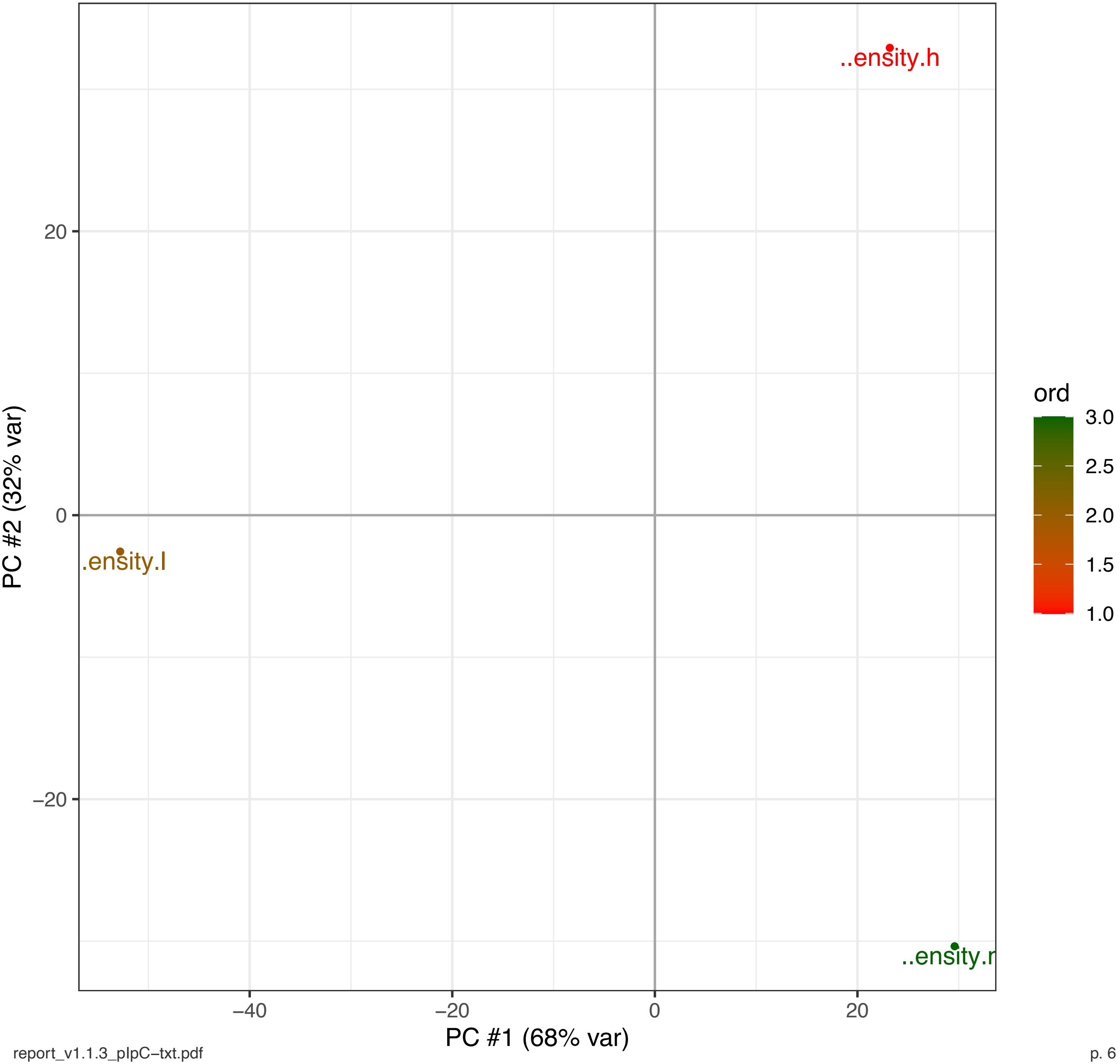

EVD: Top5 Contaminants per Raw file

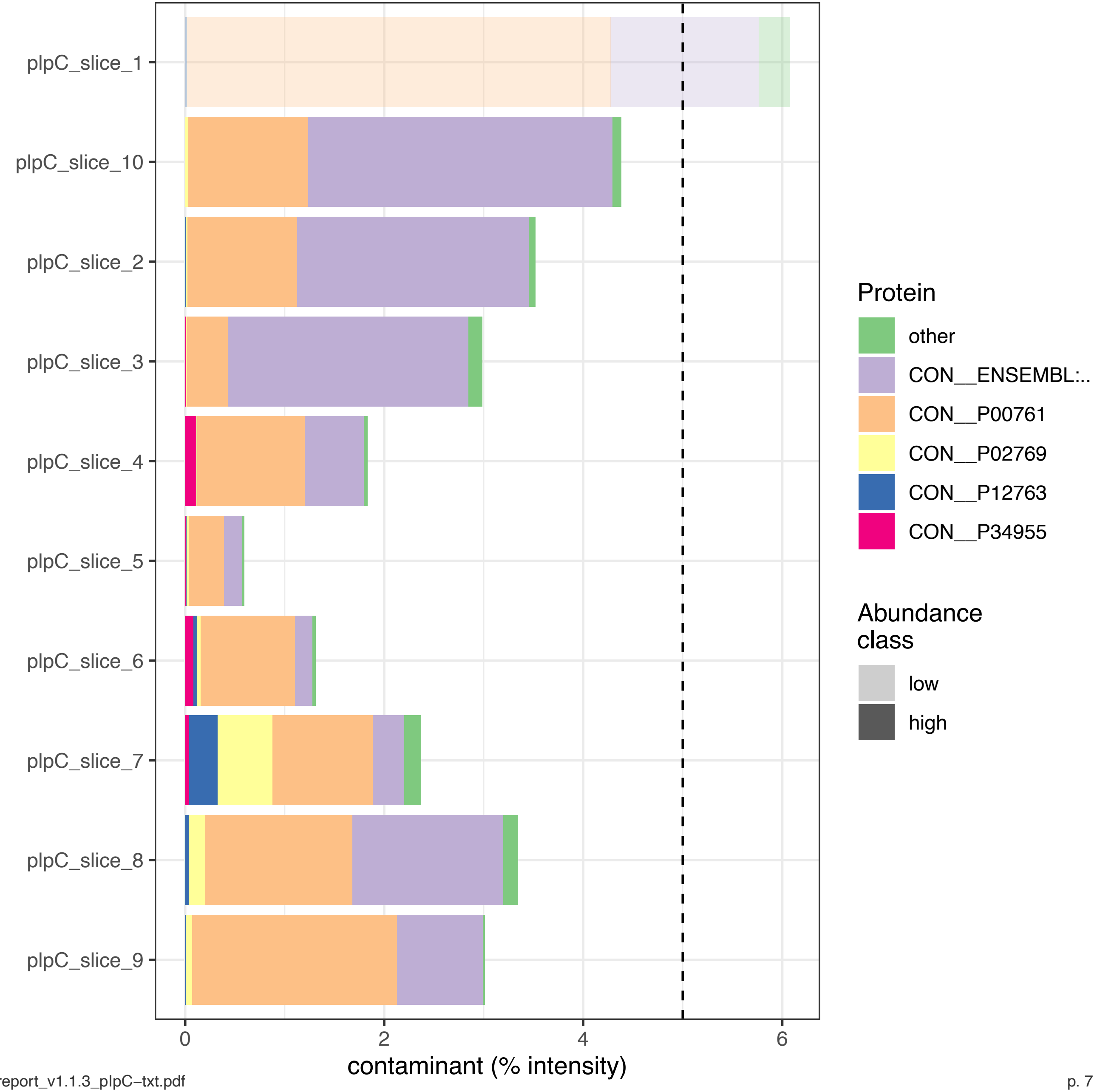

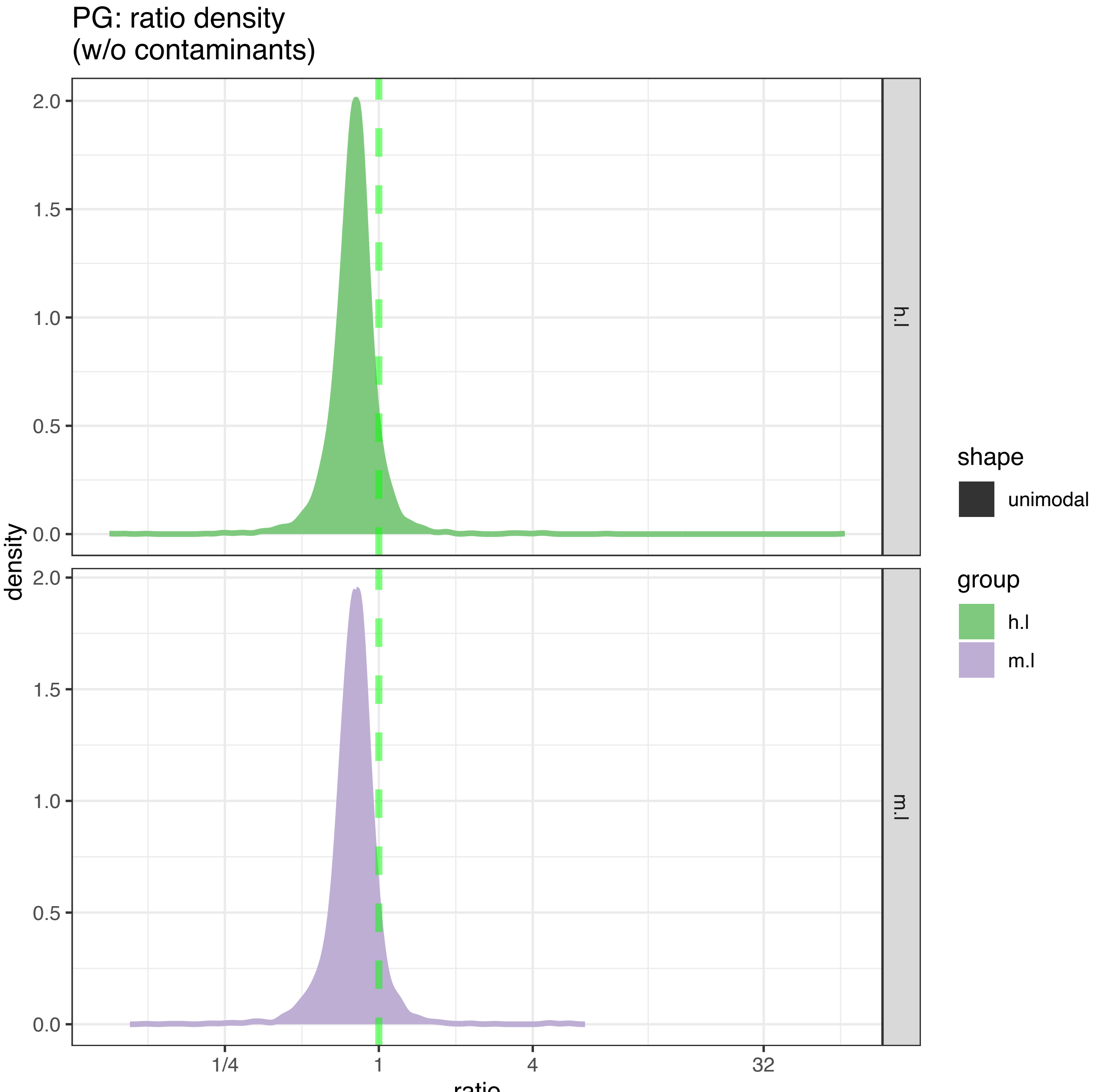
PG: ratio density
(w/o contaminants)
density
ratio
h.l
m.l
shape
unimodal
group
h.l
m.l
2.0
1.5
1.0
0.5
0.0
1/4
1
4
32

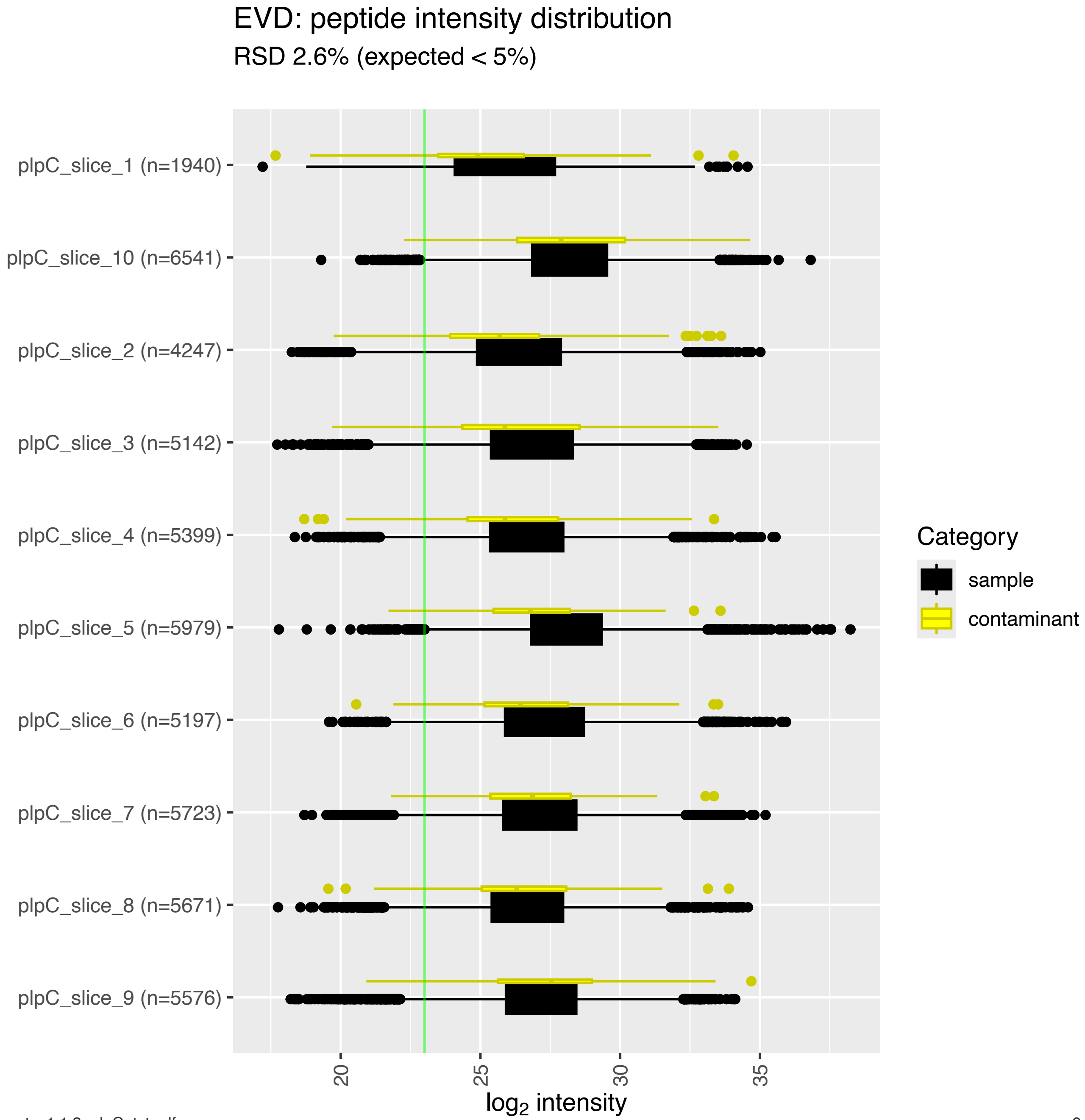
EVD: peptide intensity distribution
RSD 2.6% (expected < 5%)
plpC_slice_1 (n=1940)
plpC_slice_10 (n=6541)
plpC_slice_2 (n=4247)
plpC_slice_3 (n=5142)
plpC_slice_4 (n=5399)
plpC_slice_5 (n=5979)
plpC_slice_6 (n=5197)
plpC_slice_7 (n=5723)
plpC_slice_8 (n=5671)
plpC_slice_9 (n=5576)
20
25
30
35
log2 intensity
Category
sample
contaminant

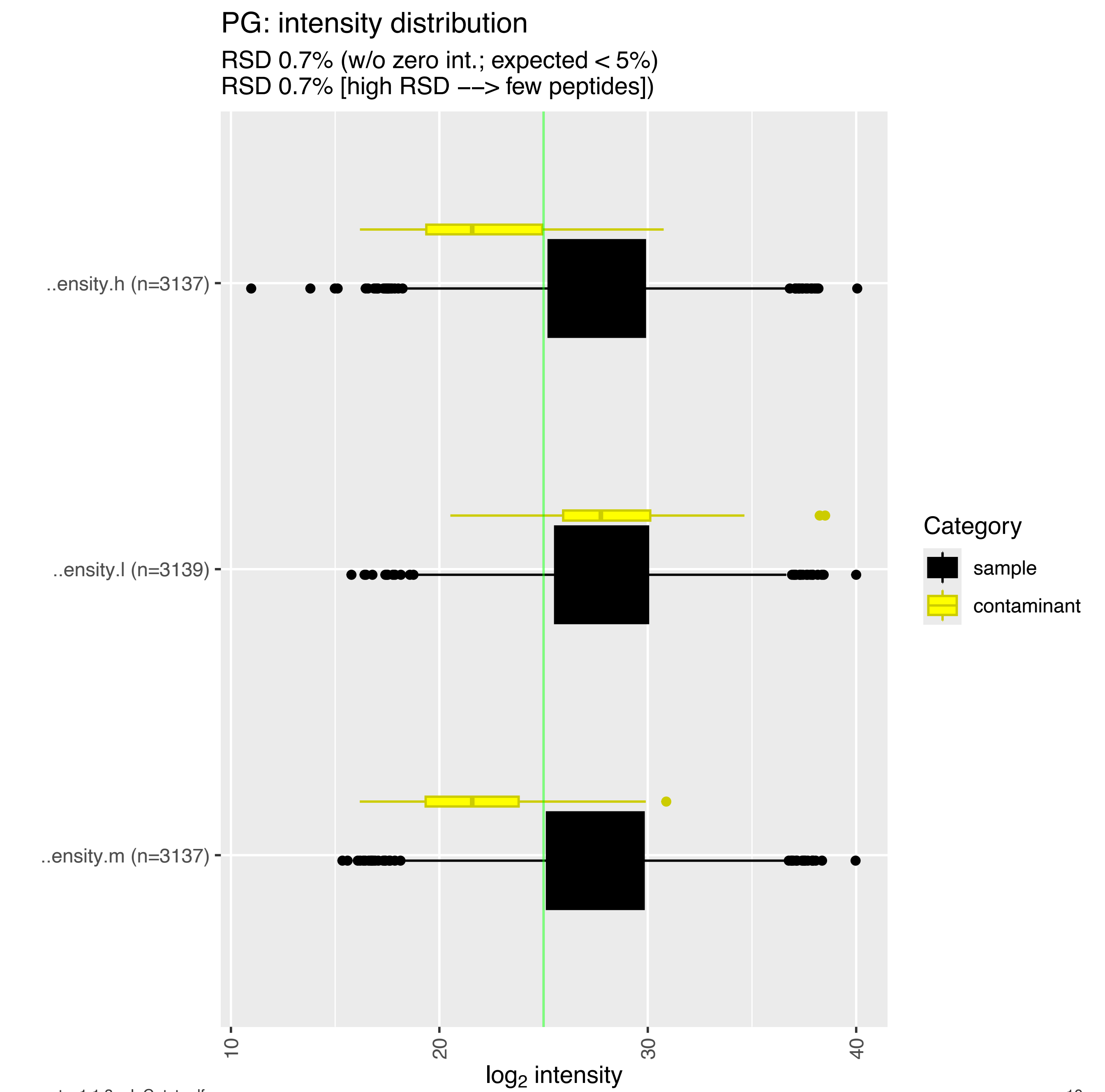
PG: intensity distribution
RSD 0.7% (w/o zero int.; expected < 5%)
RSD 0.7% [high RSD --> few peptides])
..ensity.h (n=3137)
..ensity.l (n=3139)
..ensity.m (n=3137)
10
20
30
40
log2 intensity
Category
sample
contaminant

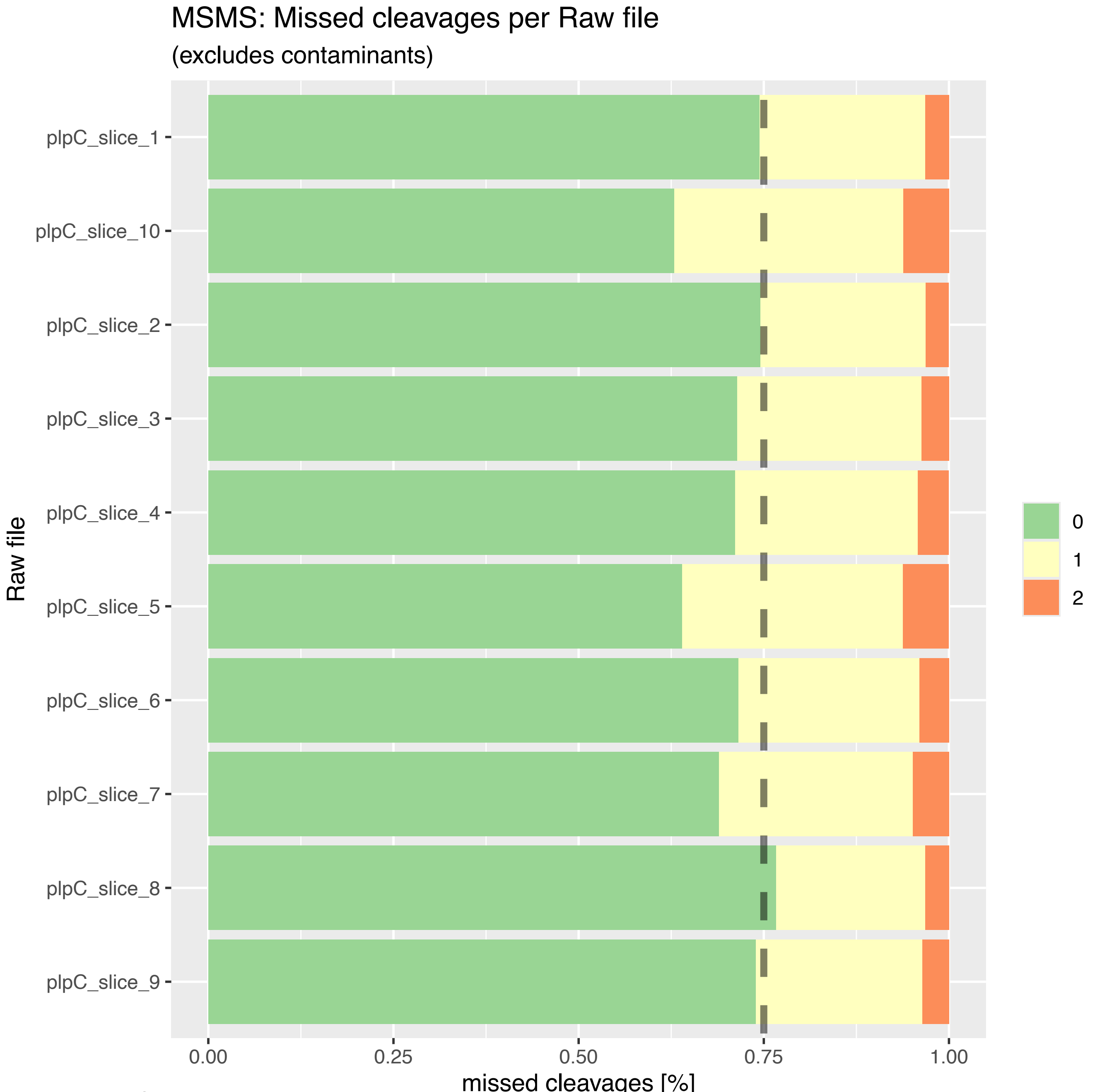
MSMS: Missed cleavages per Raw file
(excludes contaminants)
Raw file
plpC_slice_1
plpC_slice_10
plpC_slice_2
plpC_slice_3
plpC_slice_4
plpC_slice_5
plpC_slice_6
plpC_slice_7
plpC_slice_8
plpC_slice_9
0.00
0.25
0.50
0.75
1.00
missed cleavages [%]
0
1
2

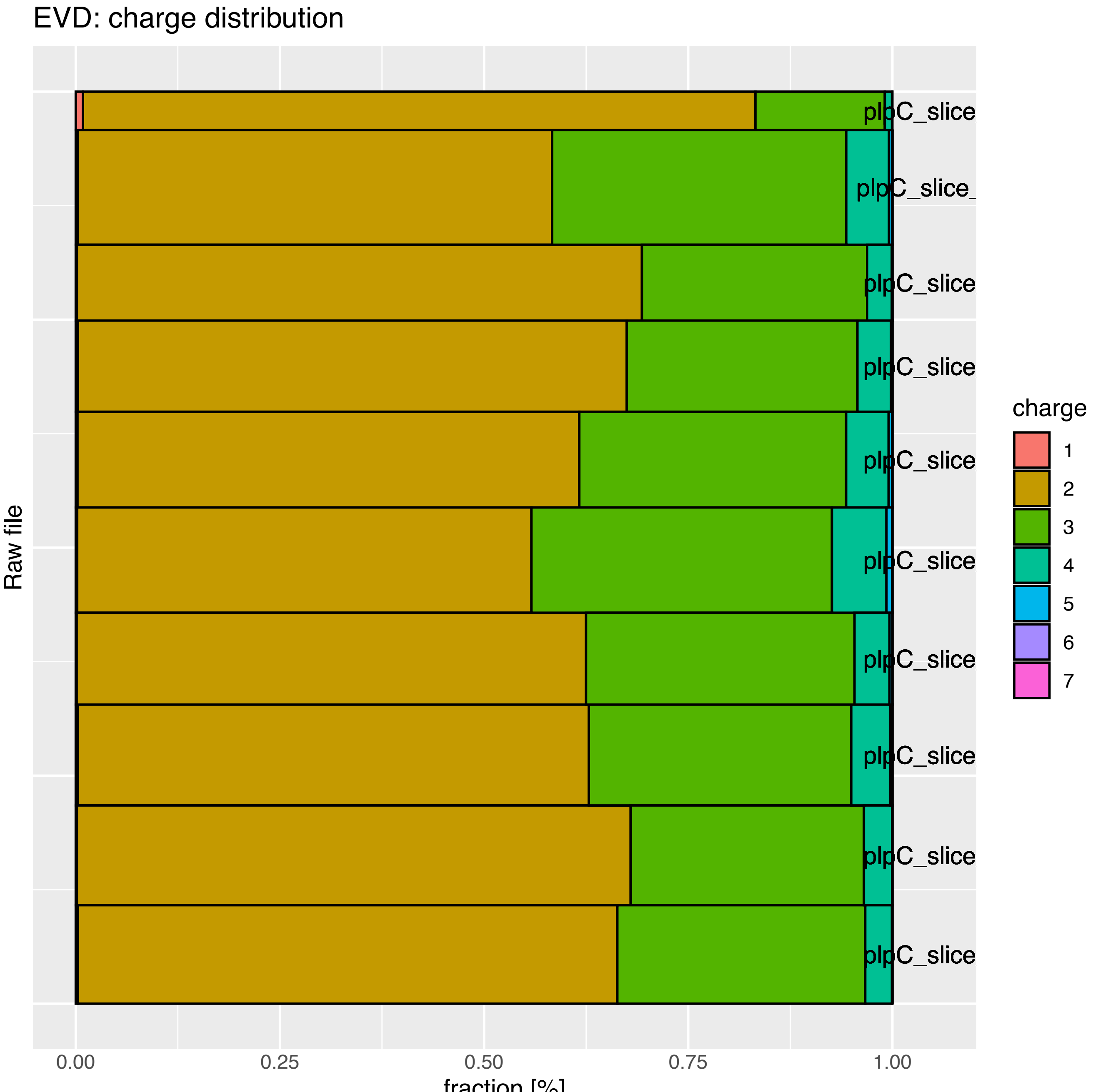
EVD: charge distribution
plpC_slice_
plpC_slice_
plpC_slice_
plpC_slice_
plpC_slice_
plpC_slice_
plpC_slice_
plpC_slice_
plpC_slice_
plpC_slice_
Raw file
0.00
0.25
0.50
0.75
1.00
fraction [%]
charge
1
2
3
4
5
6
7

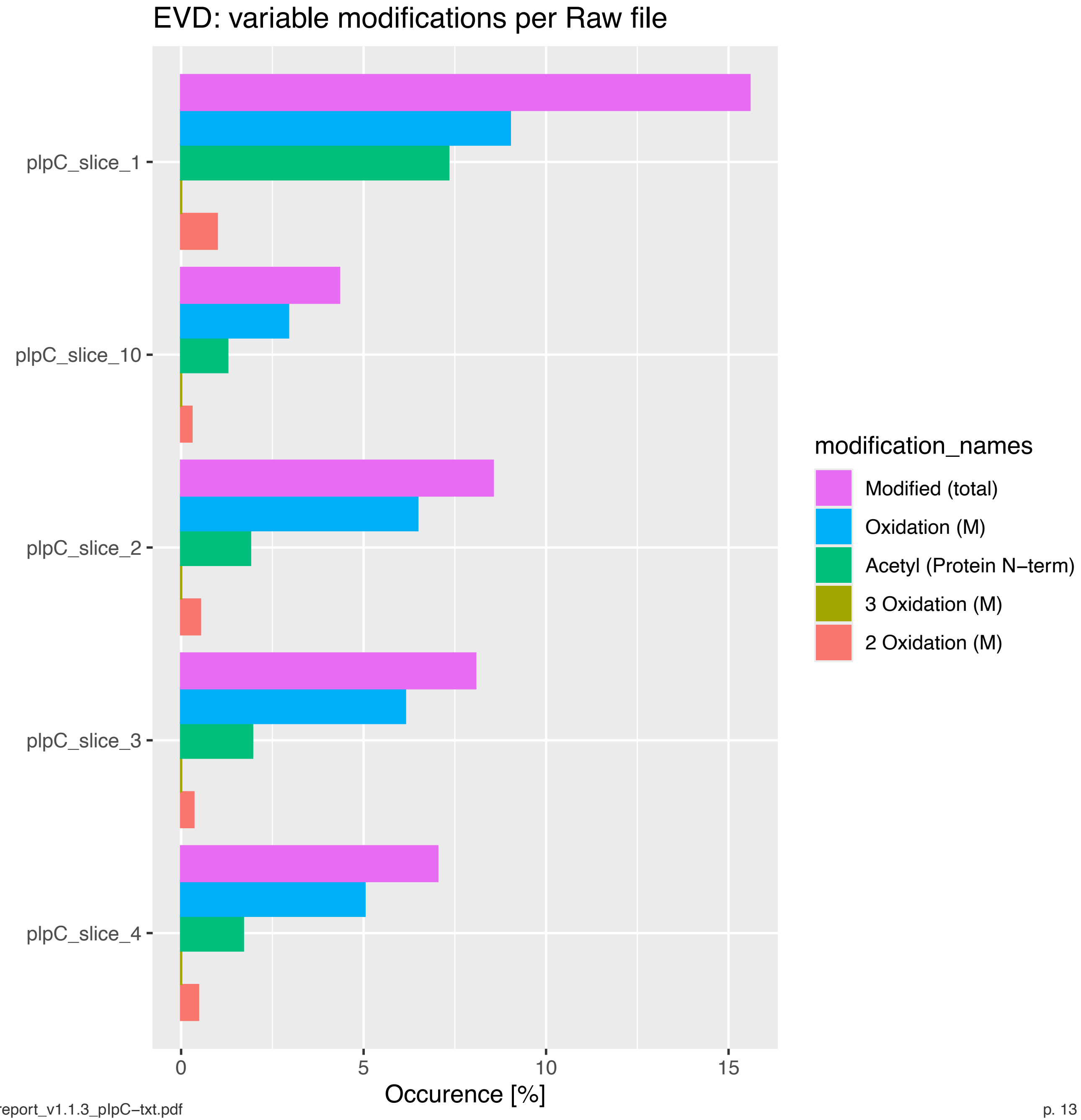
EVD: variable modifications per Raw file
plpC_slice_1
plpC_slice_10
plpC_slice_2
plpC_slice_3
plpC_slice_4
0
5
10
15
Occurence [%]
modification_names
Modified (total)
Oxidation (M)
Acetyl (Protein N−term)
3 Oxidation (M)
2 Oxidation (M)

EVD: variable modifications per Raw file

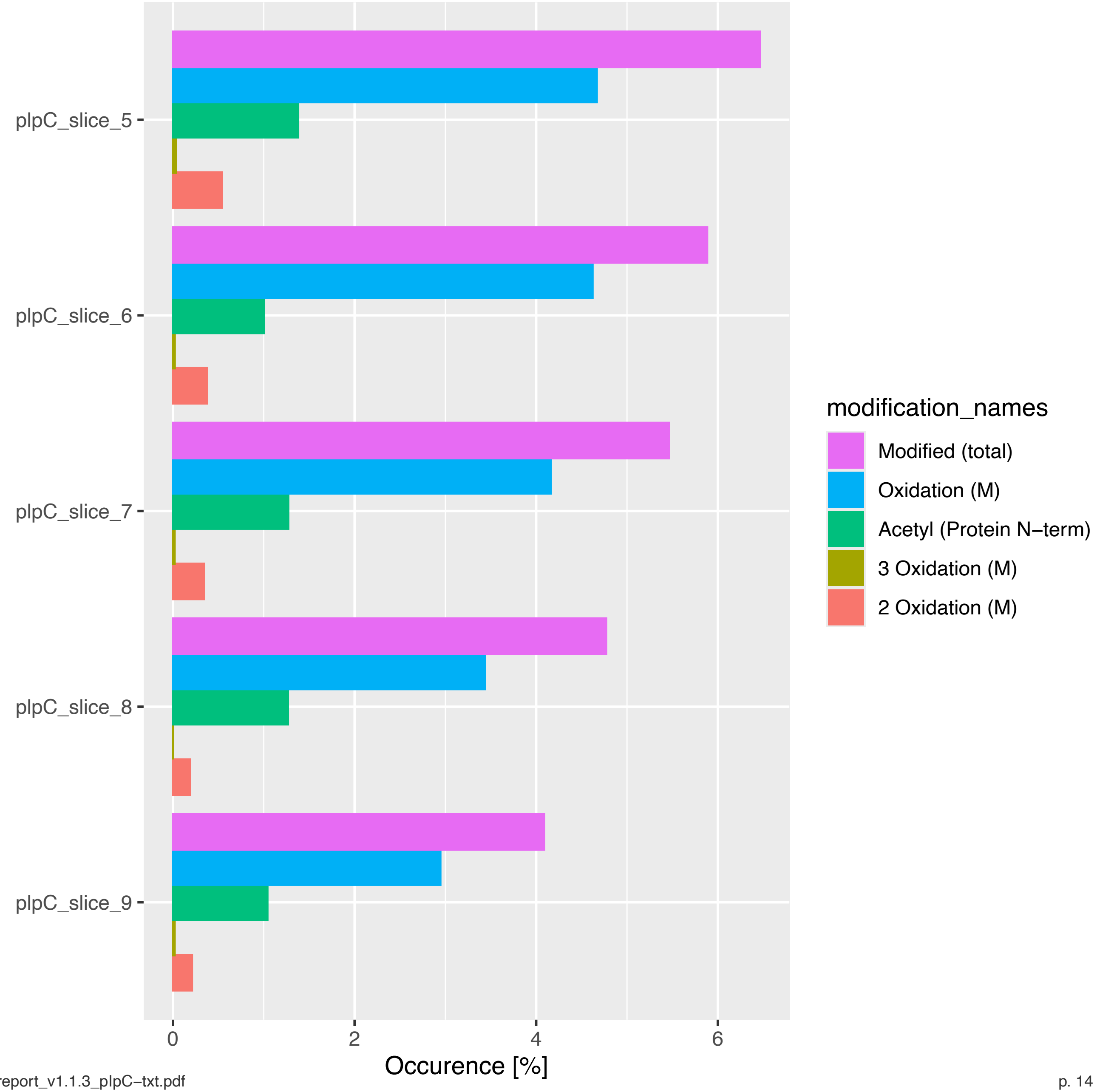

PG: Contaminant per condition

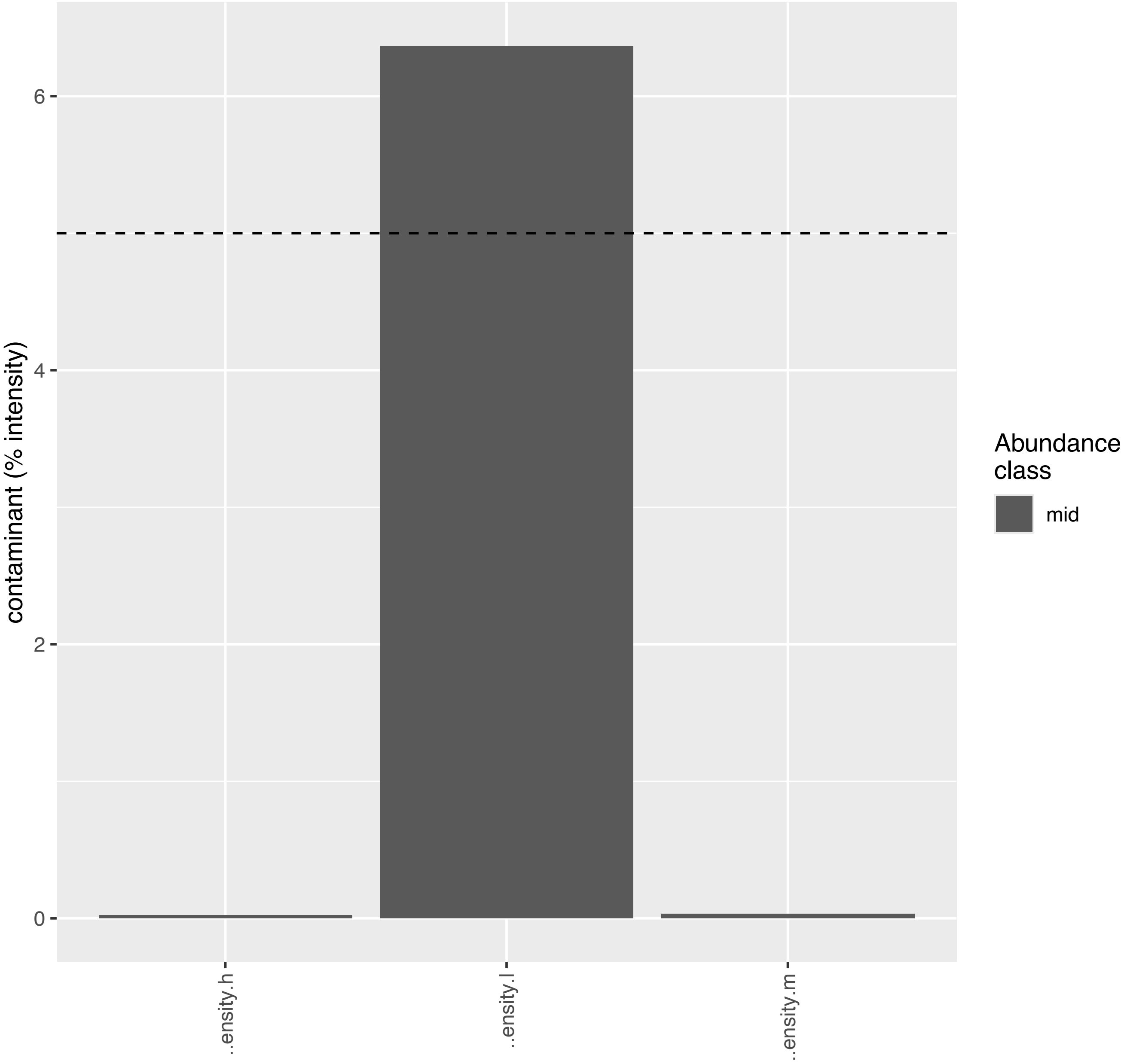

EVD: IDs over RT

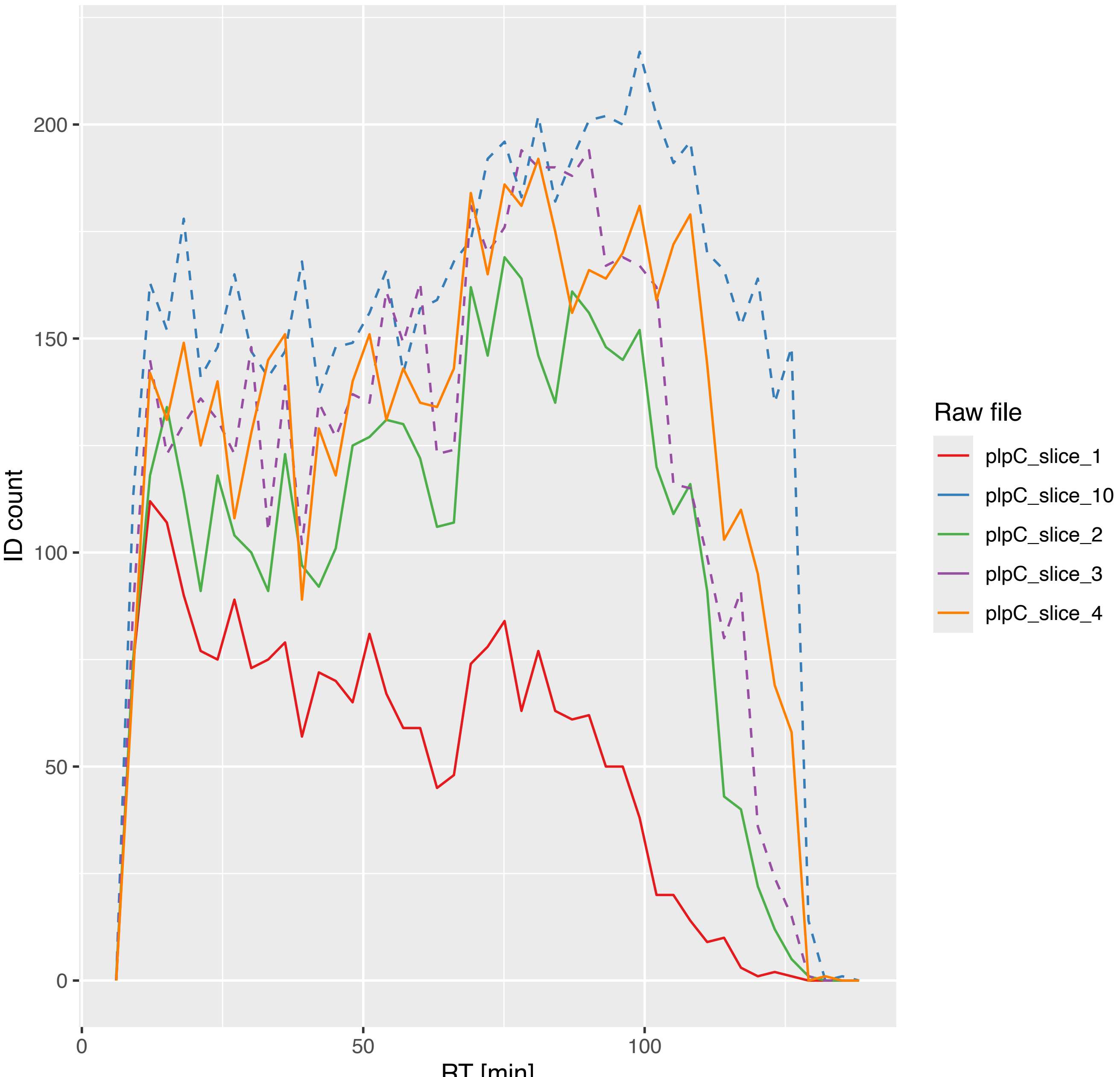

EVD: IDs over RT

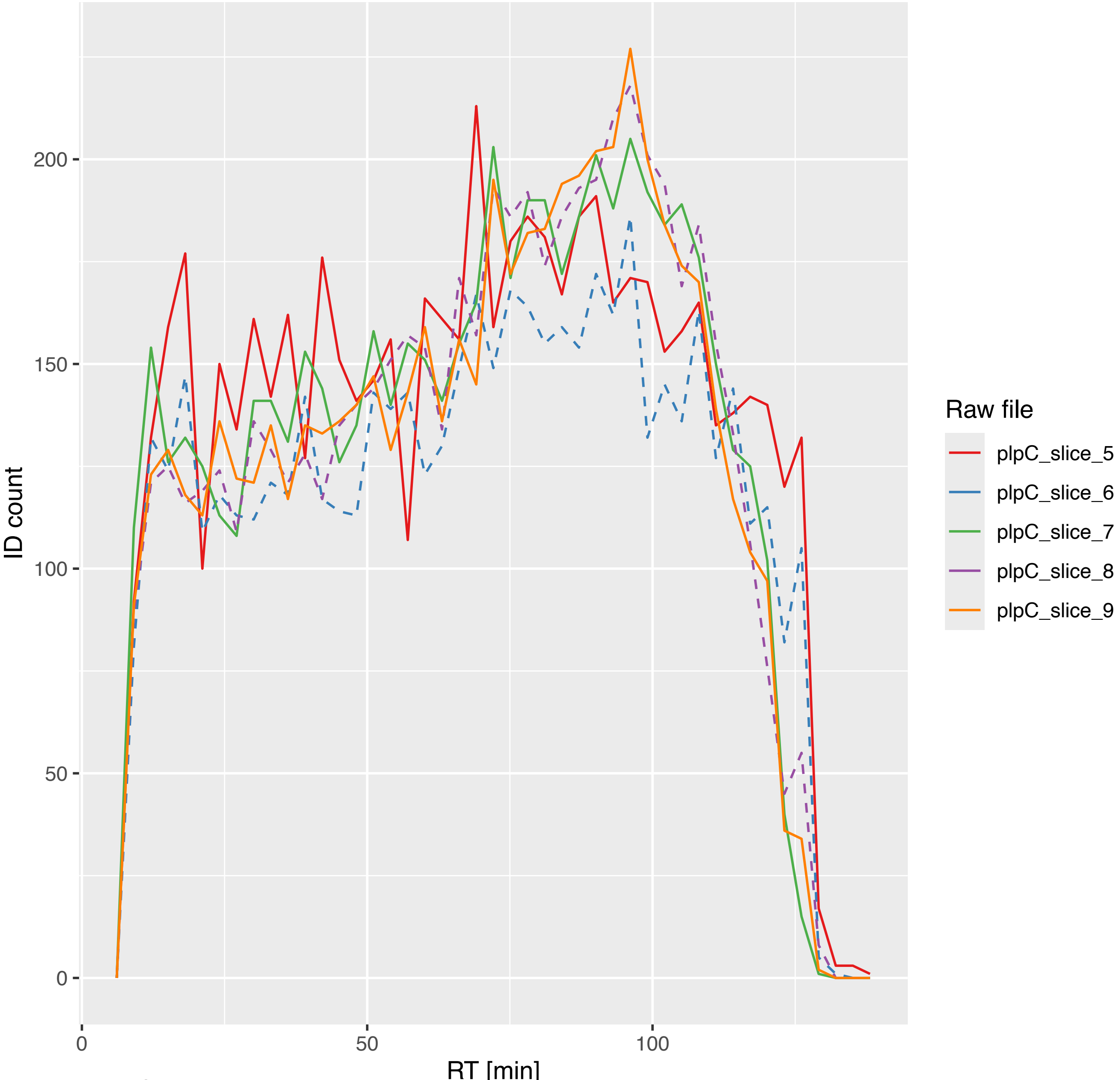

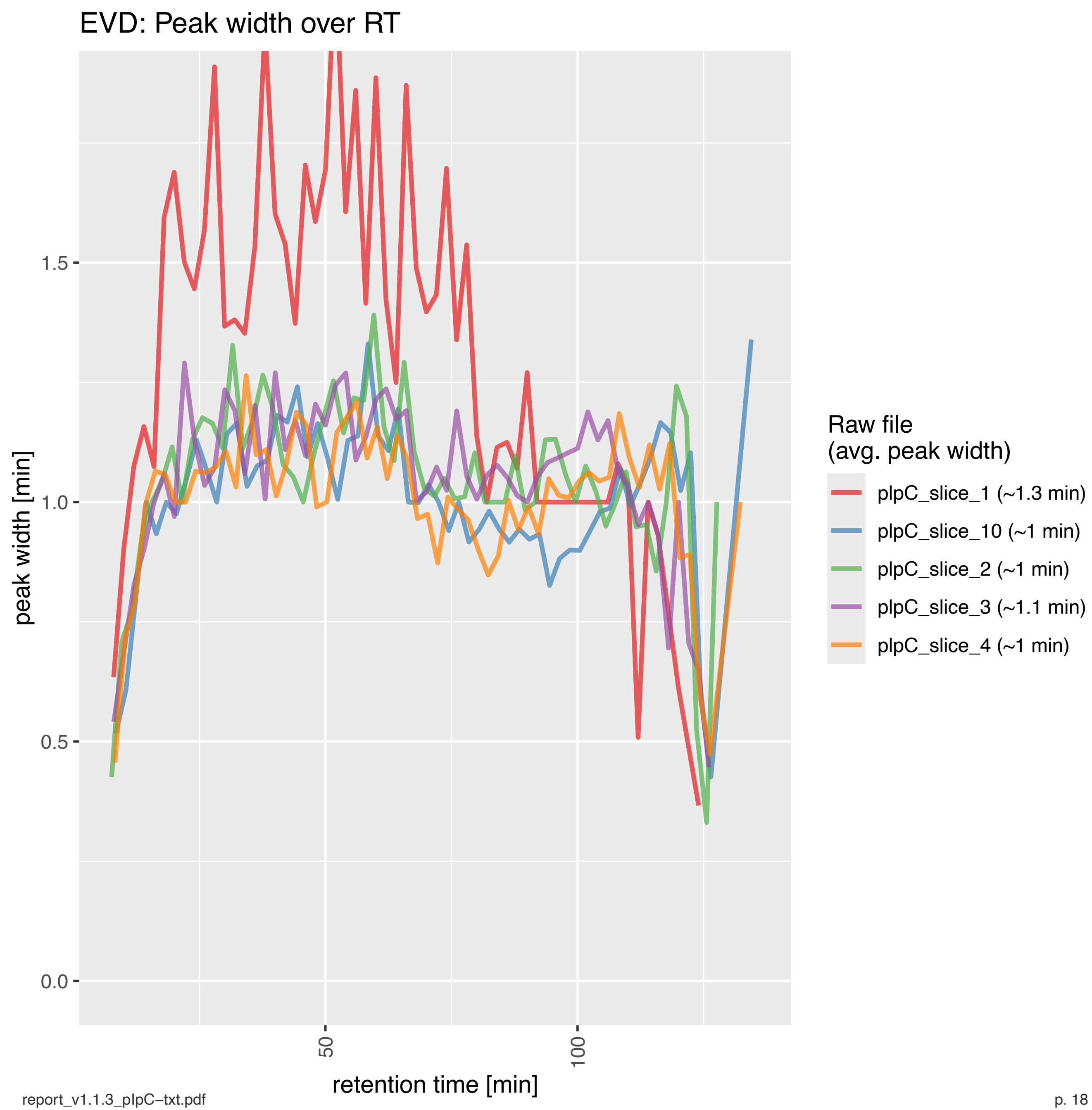

EVD: Peak width over RT
peak width [min]
1.5
1.0
0.5
0.0
50
100
retention time [min]
Raw file
(avg. peak width)
plpC_slice_1 (~1.3 min)
plpC_slice_10 (~1 min)
plpC_slice_2 (~1 min)
plpC_slice_3 (~1.1 min)
plpC_slice_4 (~1 min)

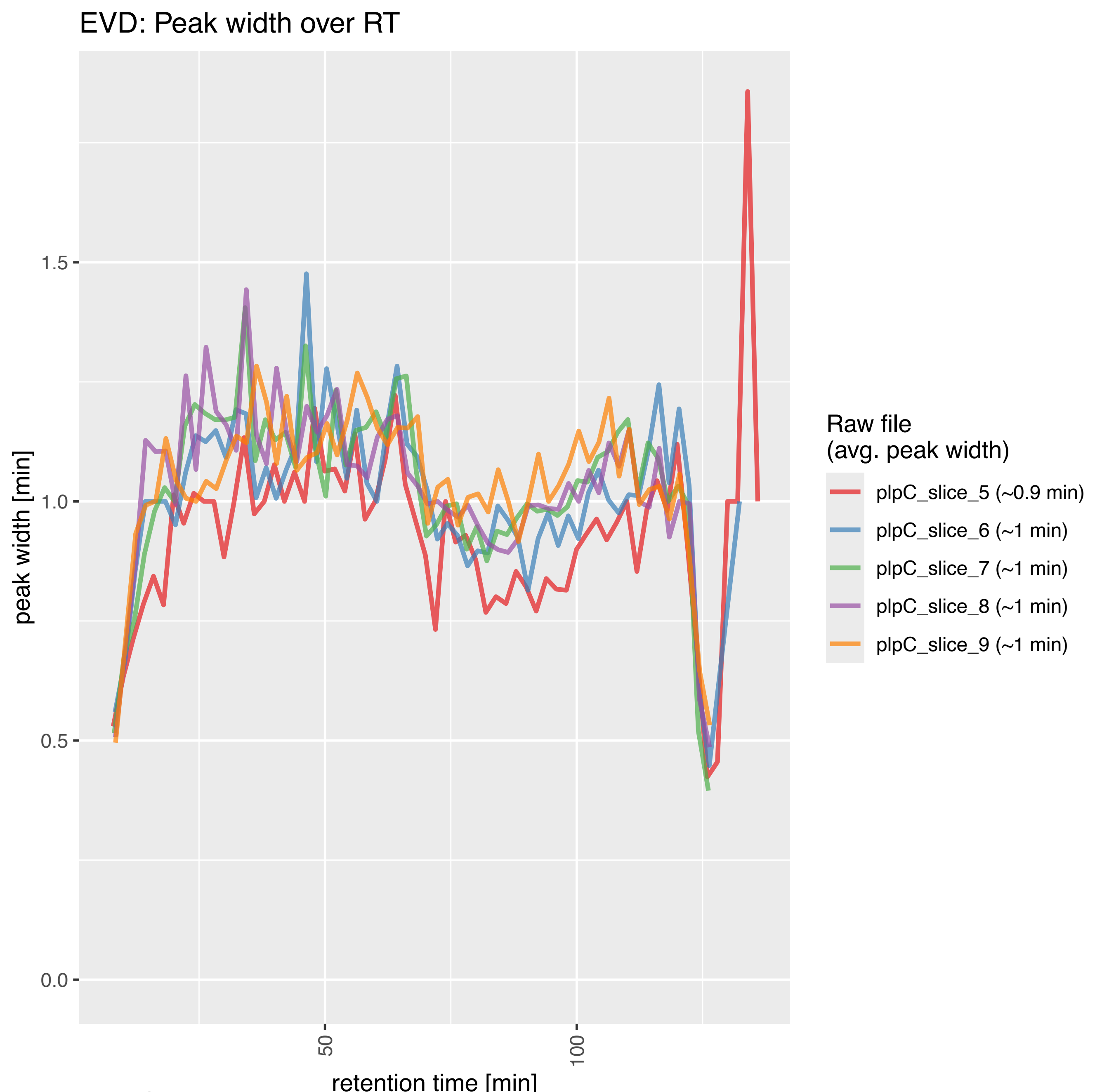

EVD: Peak width over RT
peak width [min]
0.0
0.5
1.0
1.5
50
100
retention time [min]
Raw file
(avg. peak width)
plpC_slice_5 (~0.9 min)
plpC_slice_6 (~1 min)
plpC_slice_7 (~1 min)
plpC_slice_8 (~1 min)
plpC_slice_9 (~1 min)

EVD: MBR – alignment

alignment reference: plpC_slice_1

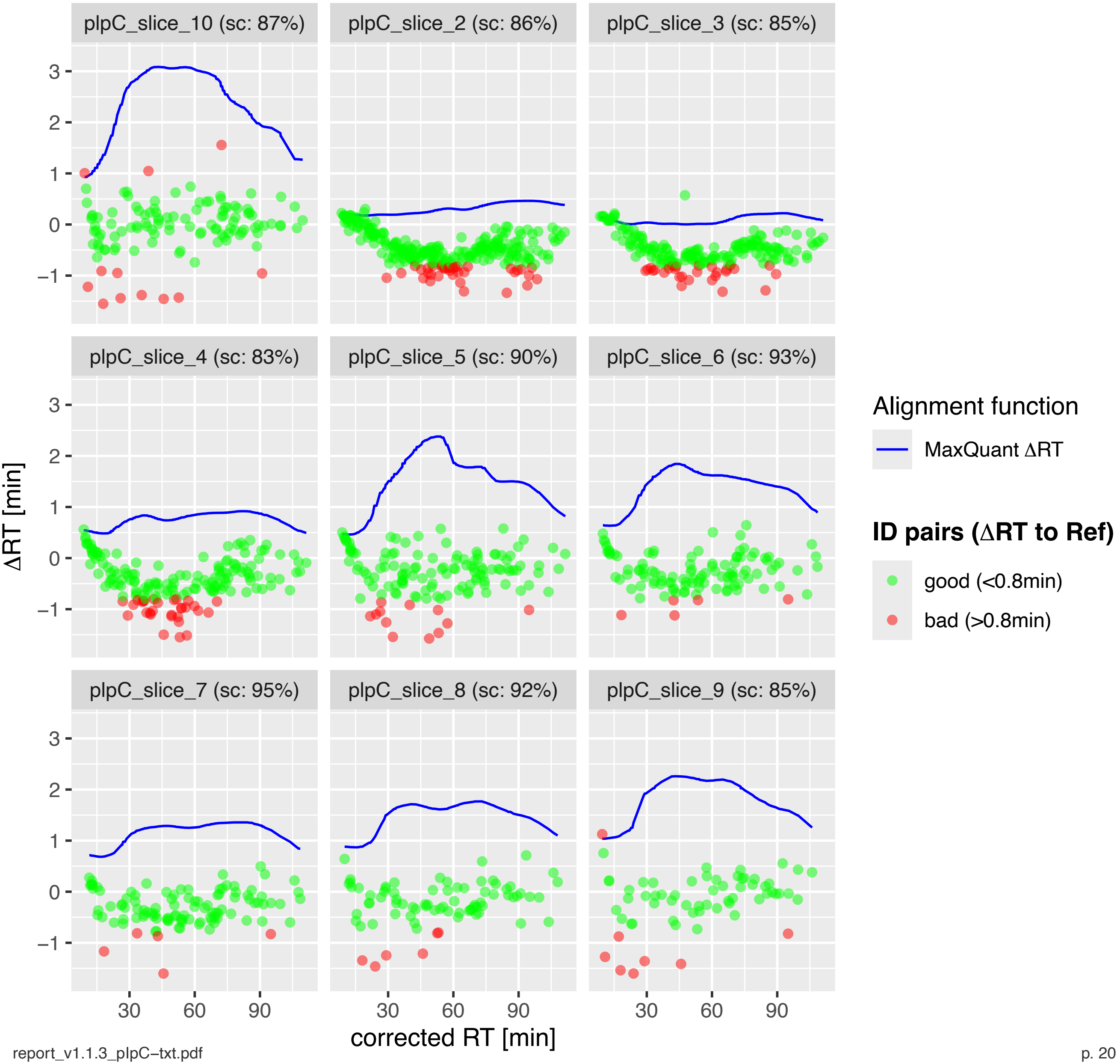

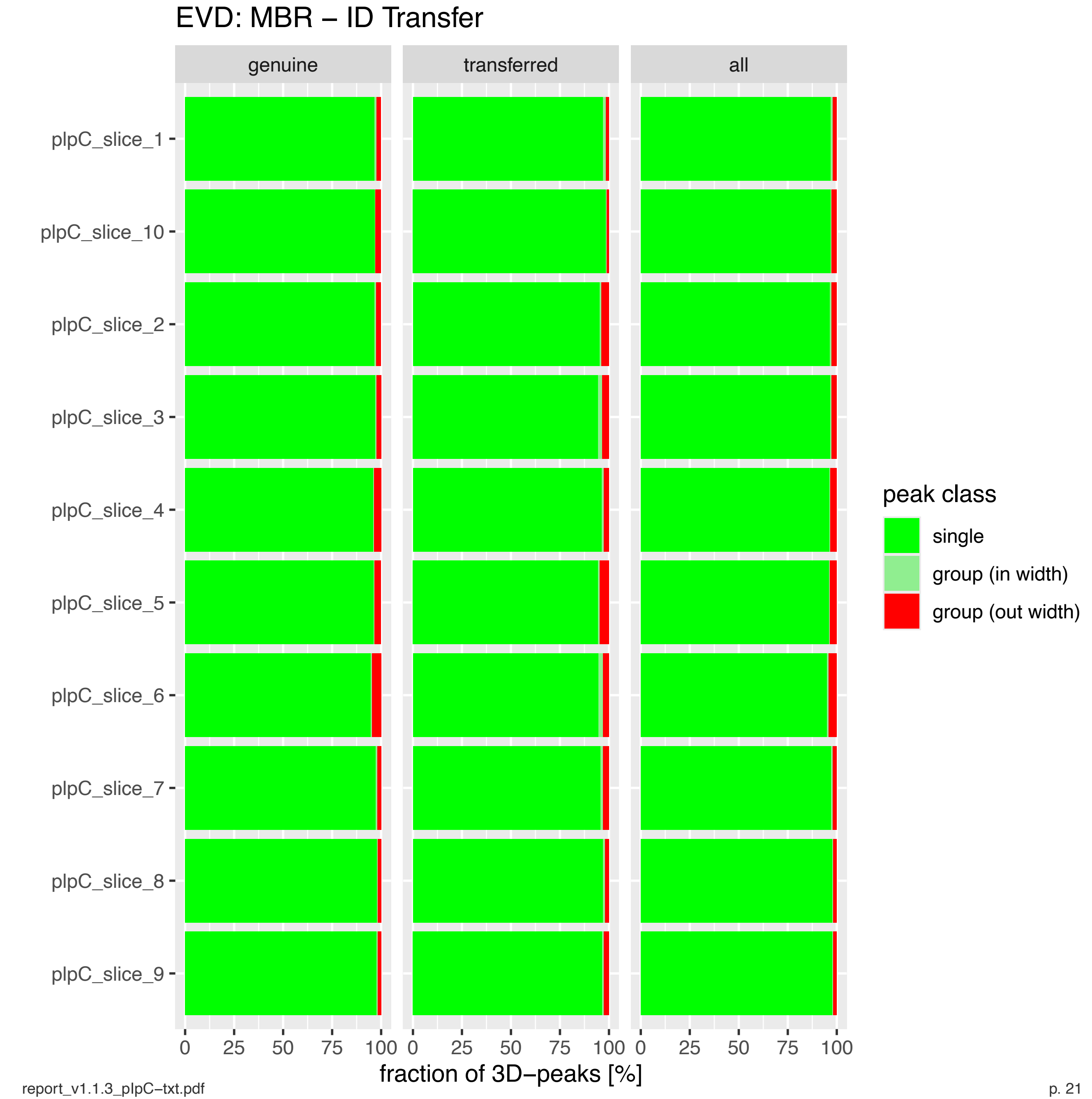
EVD: MBR – ID Transfer
genuine
transferred
all
plpC_slice_1
plpC_slice_10
plpC_slice_2
plpC_slice_3
plpC_slice_4
plpC_slice_5
plpC_slice_6
plpC_slice_7
plpC_slice_8
plpC_slice_9
0
25
50
75
100
fraction of 3D−peaks [%]
peak class
single
group (in width)
group (out width)

[experimental] EVD: Clustering Tree of Raw files

by Correlation of Corrected Retention Times

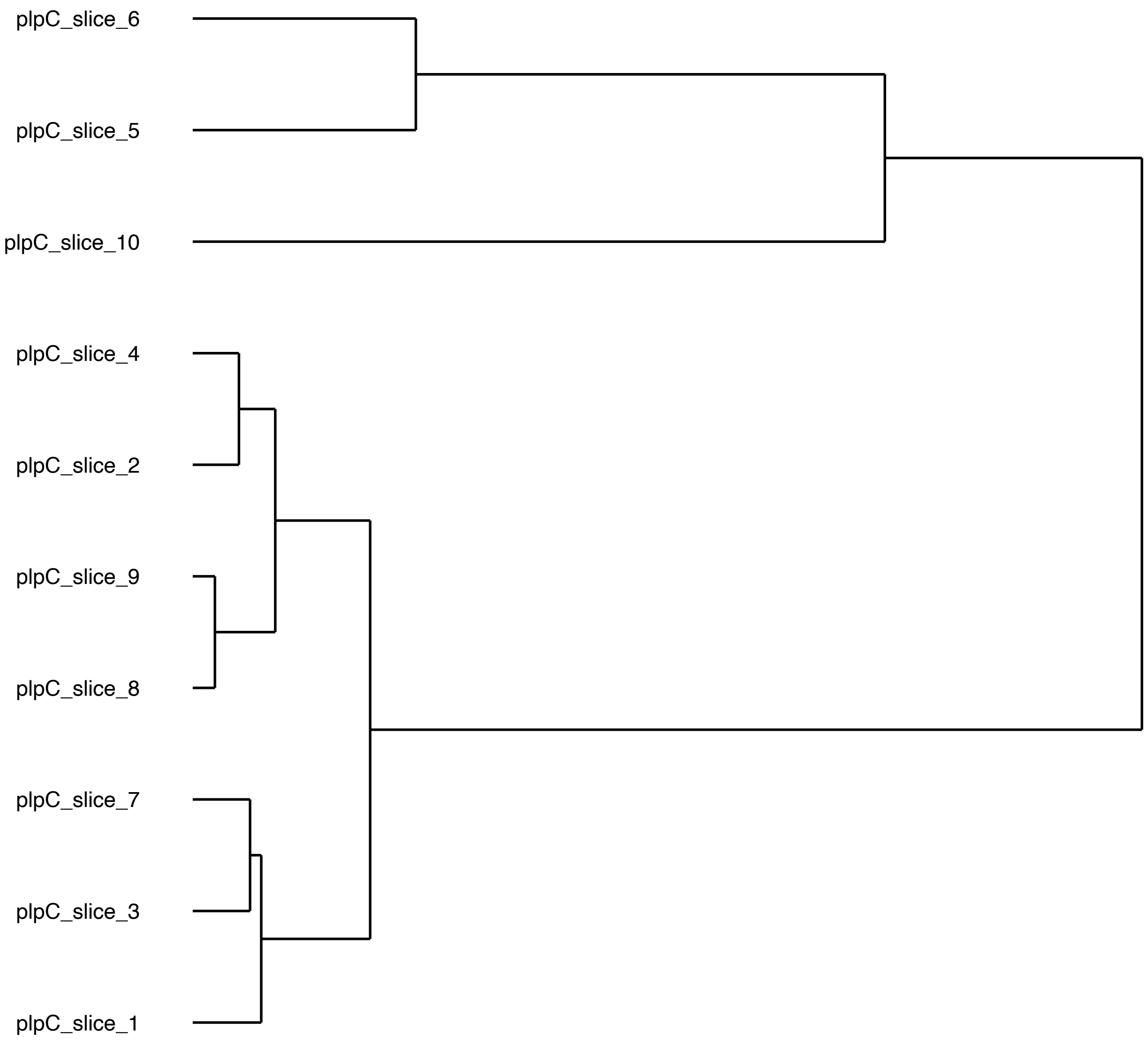

EVD: Peptides inferred by MBR

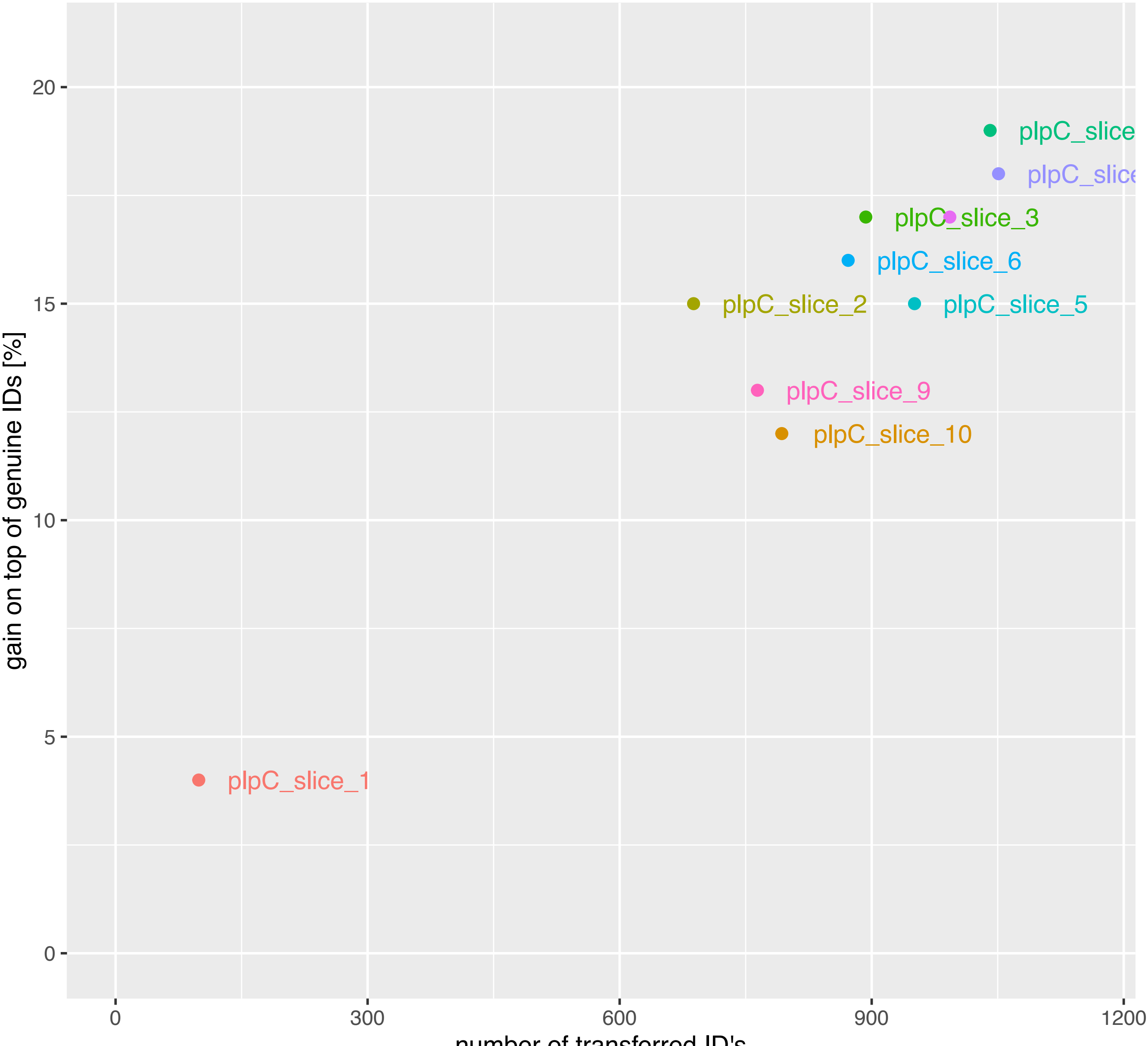

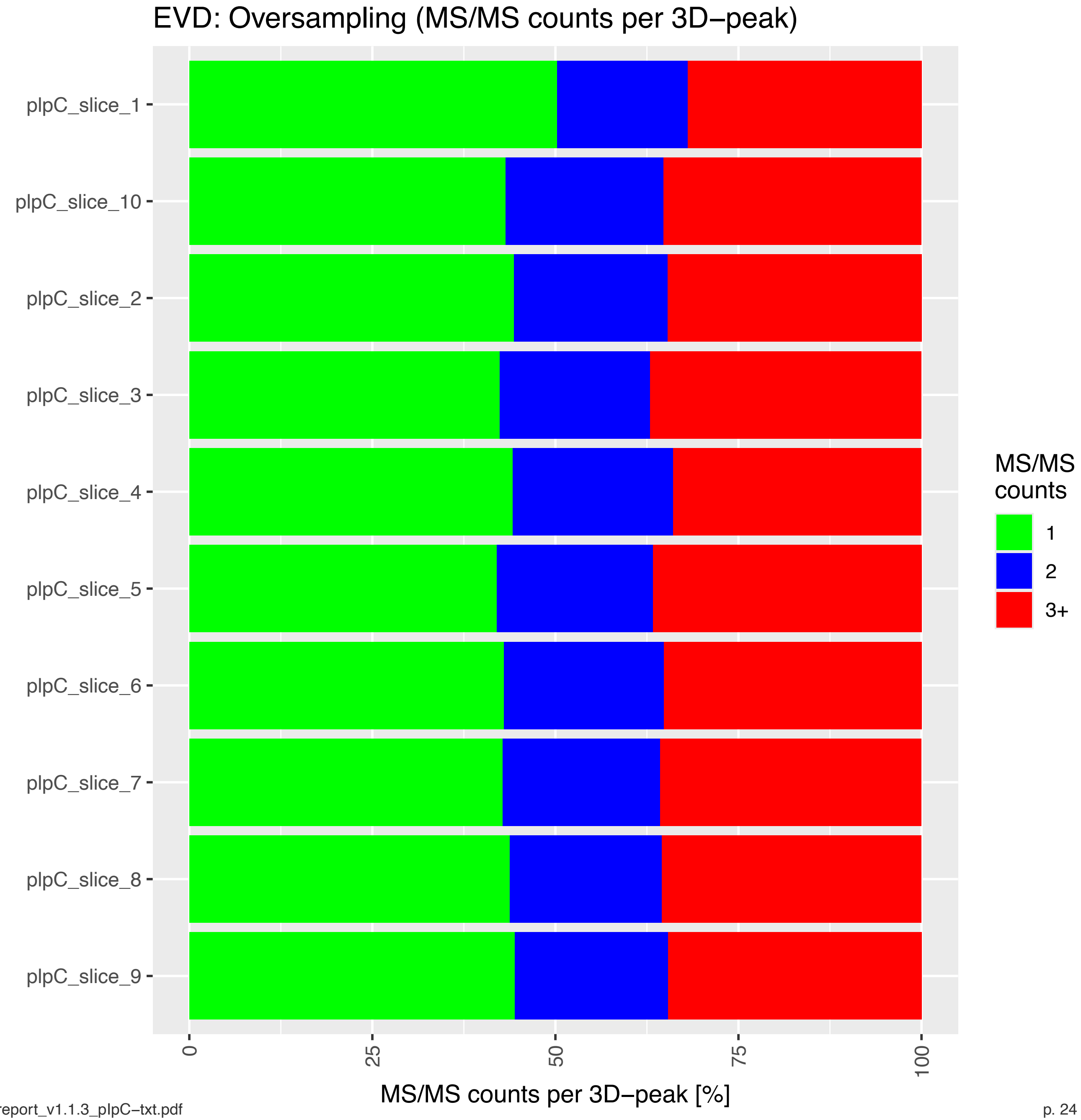
EVD: Oversampling (MS/MS counts per 3D−peak)
plpC_slice_1
plpC_slice_10
plpC_slice_2
plpC_slice_3
plpC_slice_4
plpC_slice_5
plpC_slice_6
plpC_slice_7
plpC_slice_8
plpC_slice_9
0
25
50
75
100
MS/MS counts per 3D−peak [%]
MS/MS counts
1
2
3+

EVD: Uncalibrated mass error

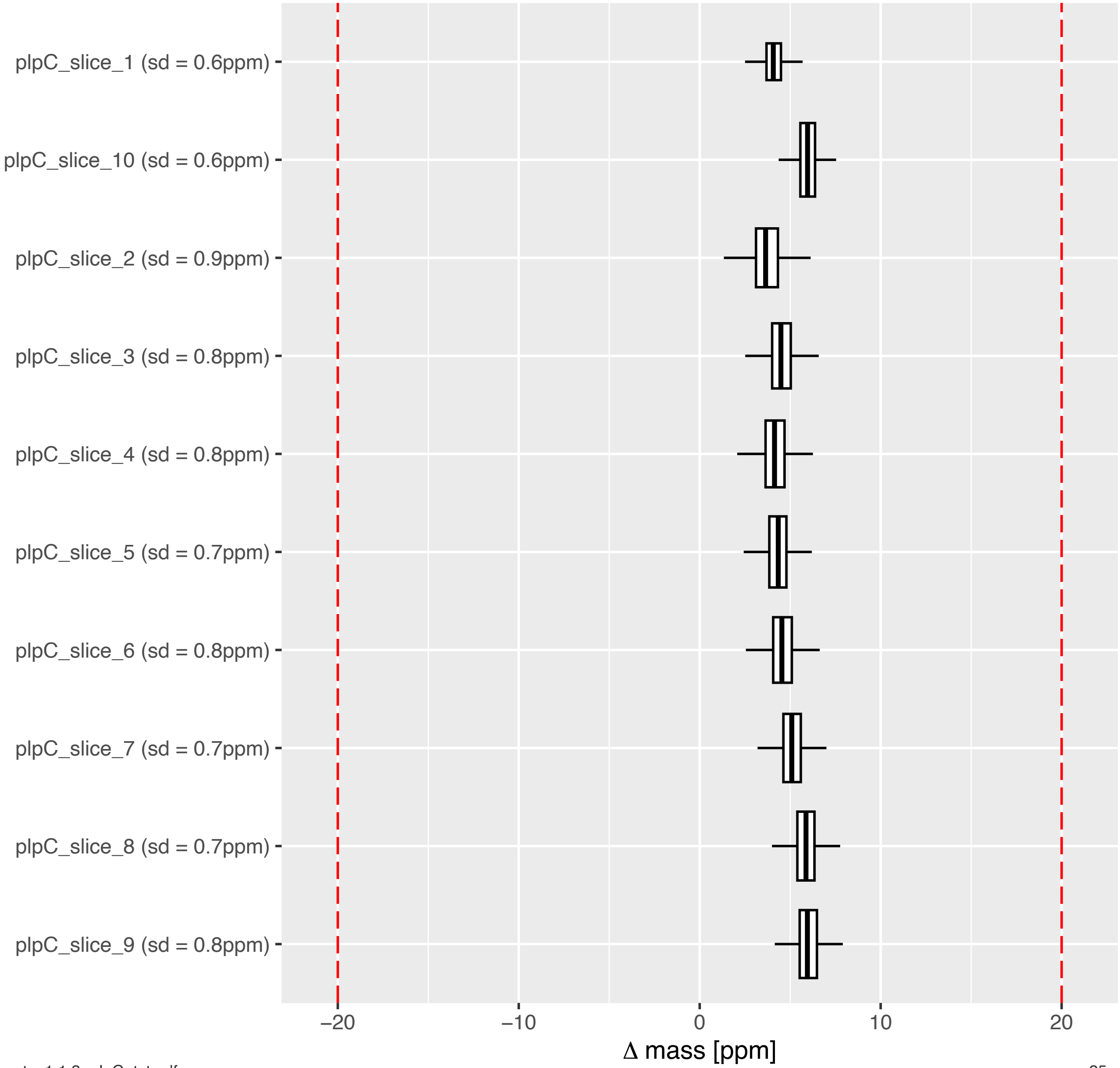

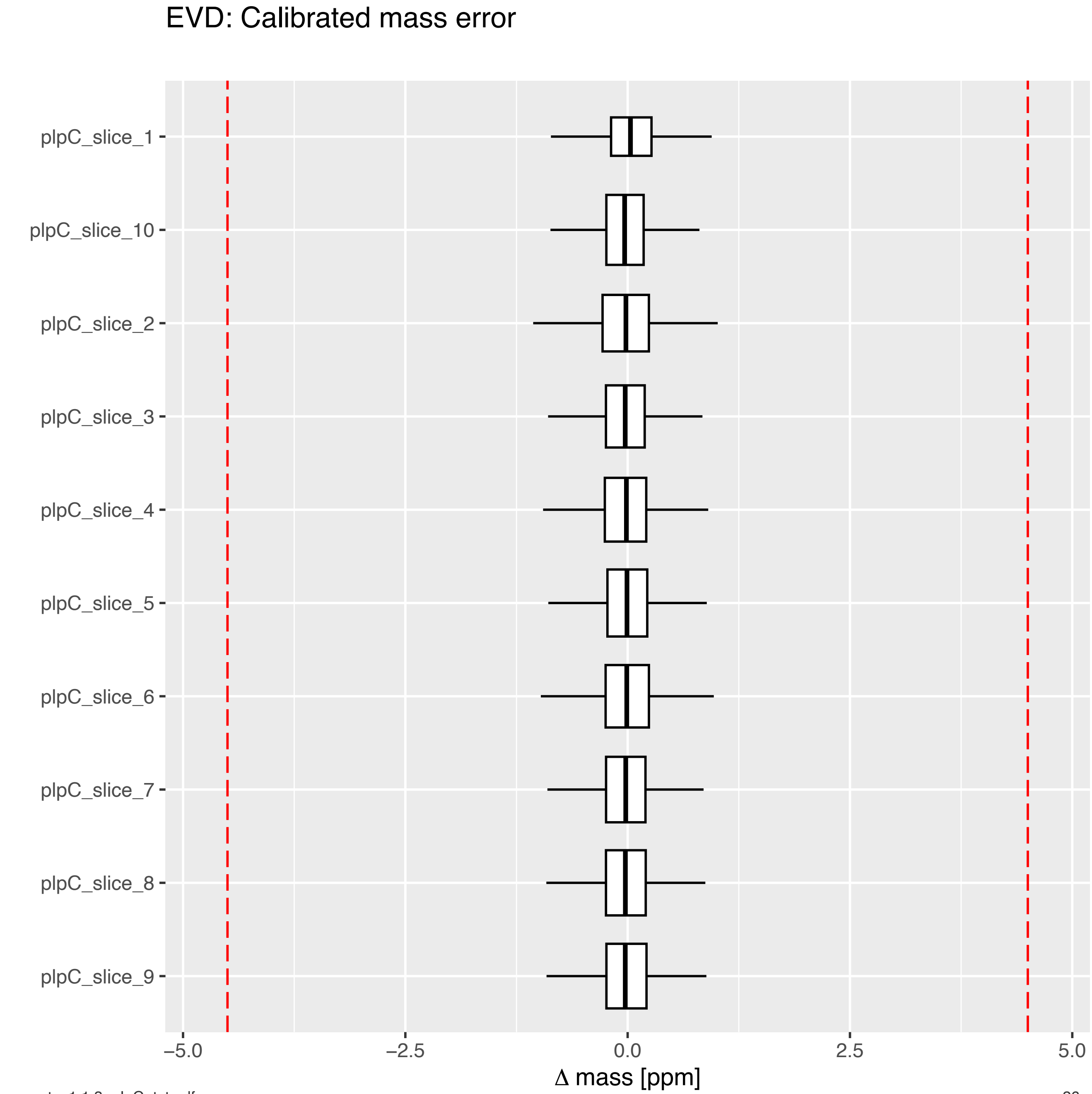
EVD: Calibrated mass error
plpC_slice_1
plpC_slice_10
plpC_slice_2
plpC_slice_3
plpC_slice_4
plpC_slice_5
plpC_slice_6
plpC_slice_7
plpC_slice_8
plpC_slice_9
−5.0
−2.5
0.0
2.5
5.0
Δ mass [ppm]

MSMS: Fragment mass errors per Raw file

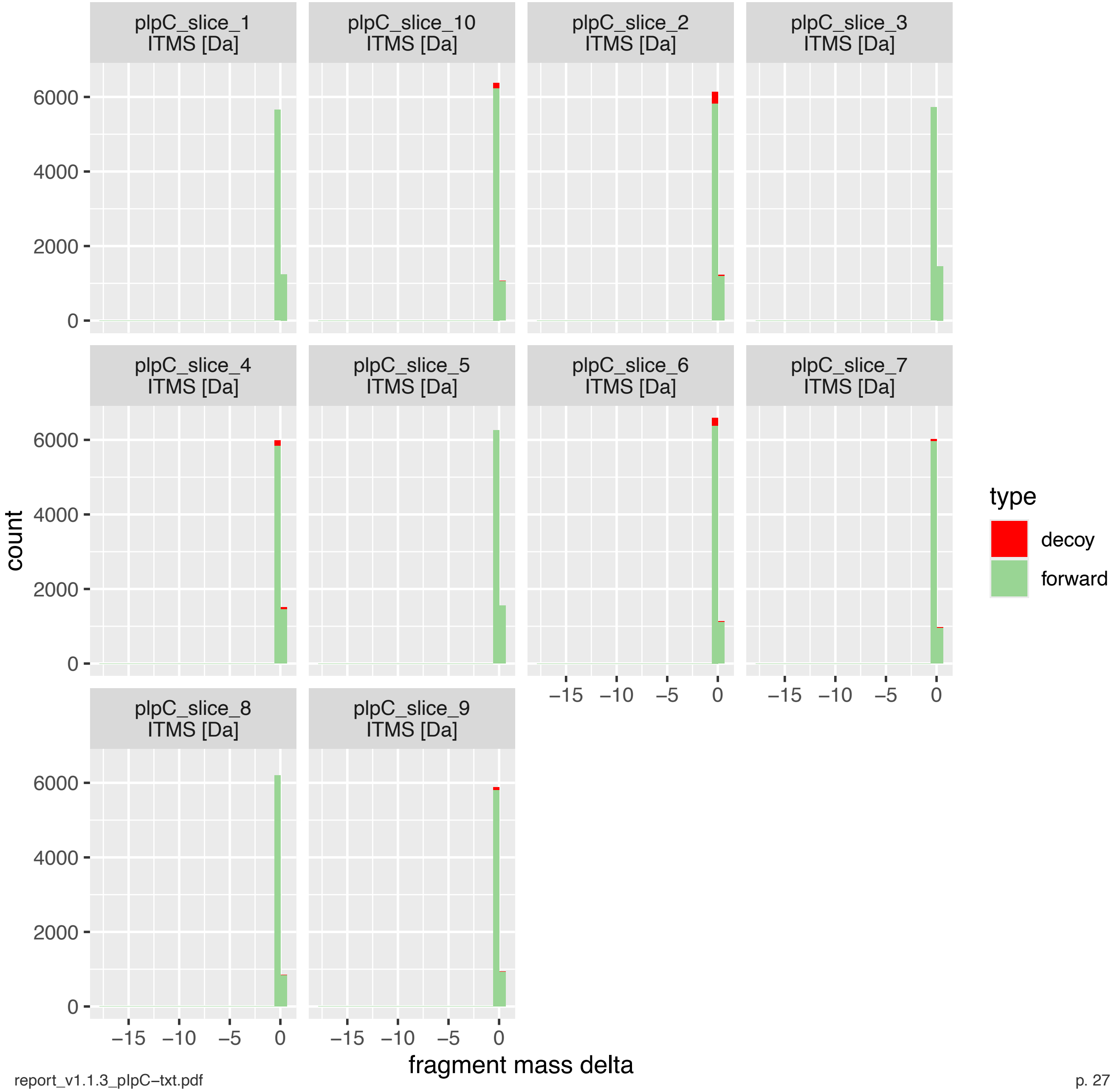

SM: MS/MS identified per Raw file

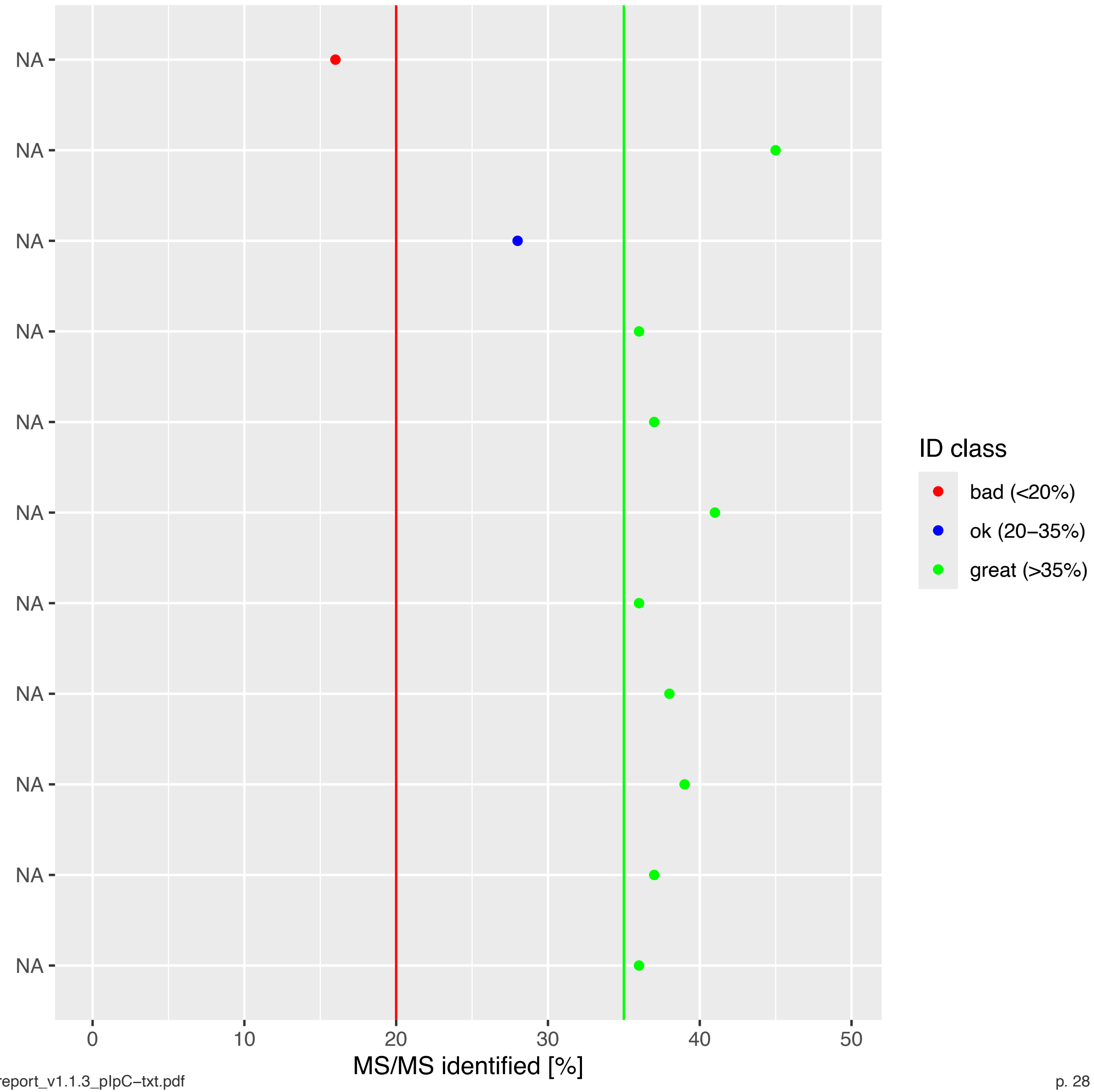

SM: Files with 'red' ID rate

Raw file	% identified
plpC_slice_1	16

9% of samples)

[experimental] EVD: Non−Missing Peptides

compared to all peptides seen in experiment

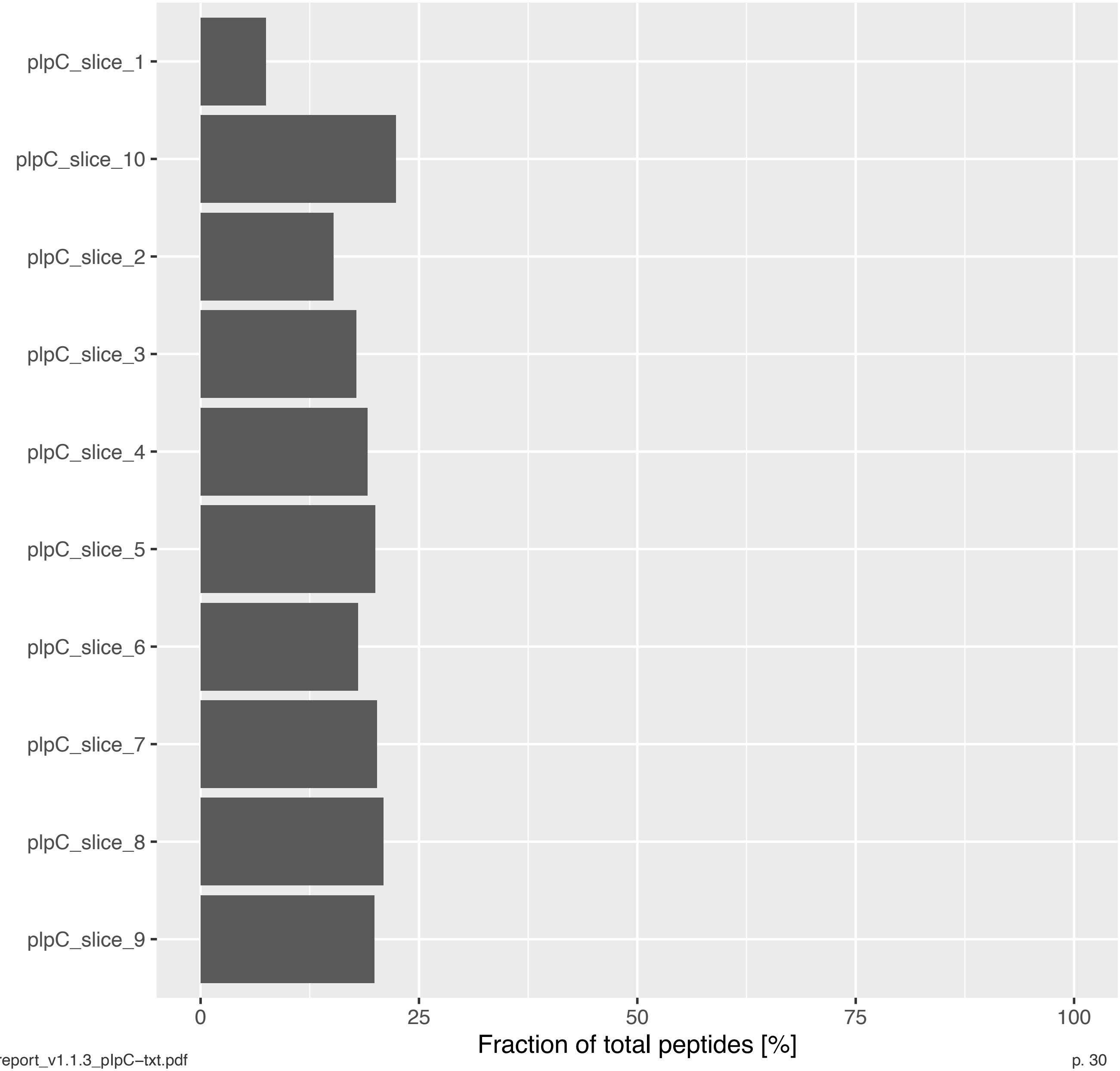

[experimental] EVD: Non−missing by set

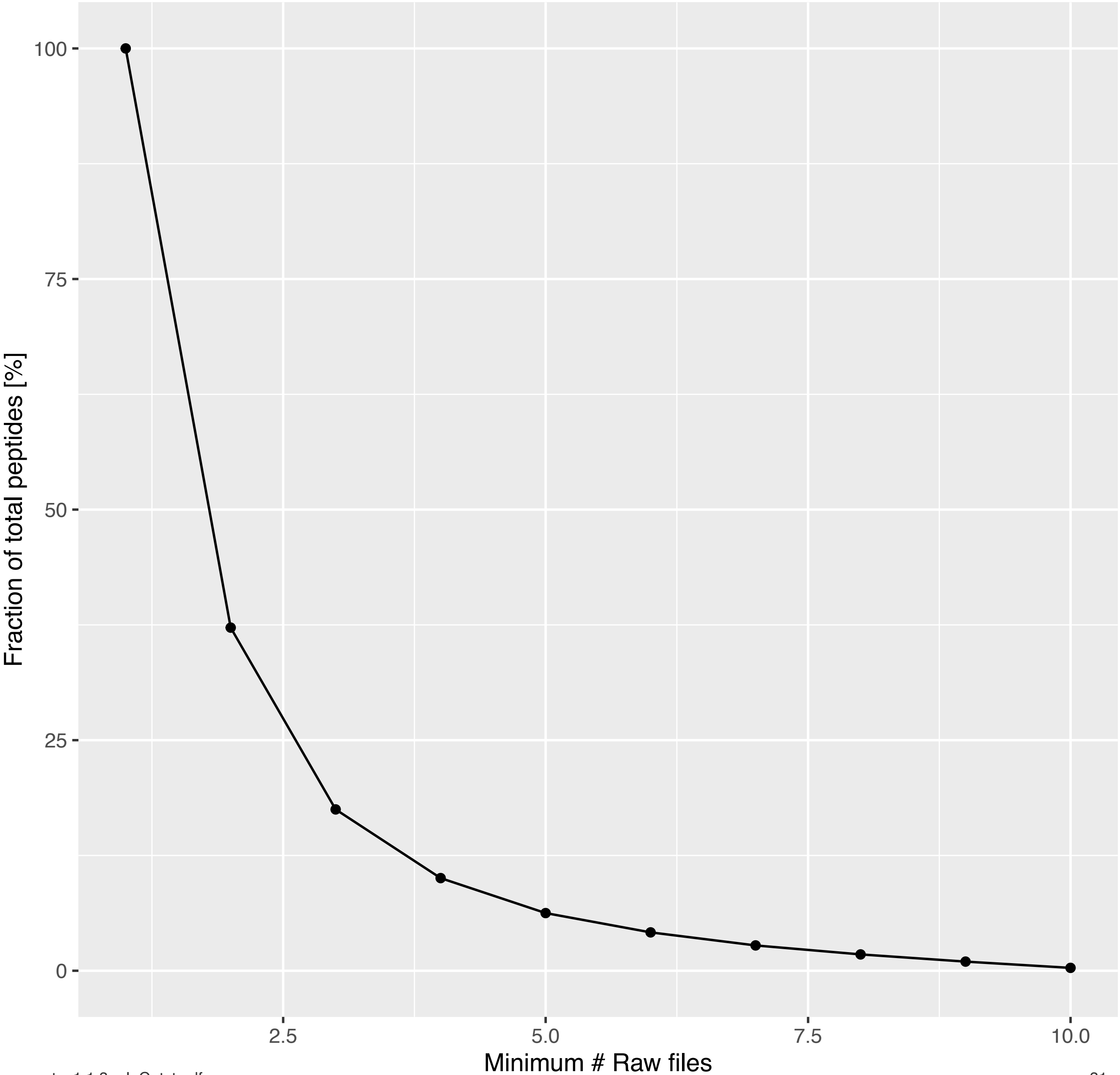

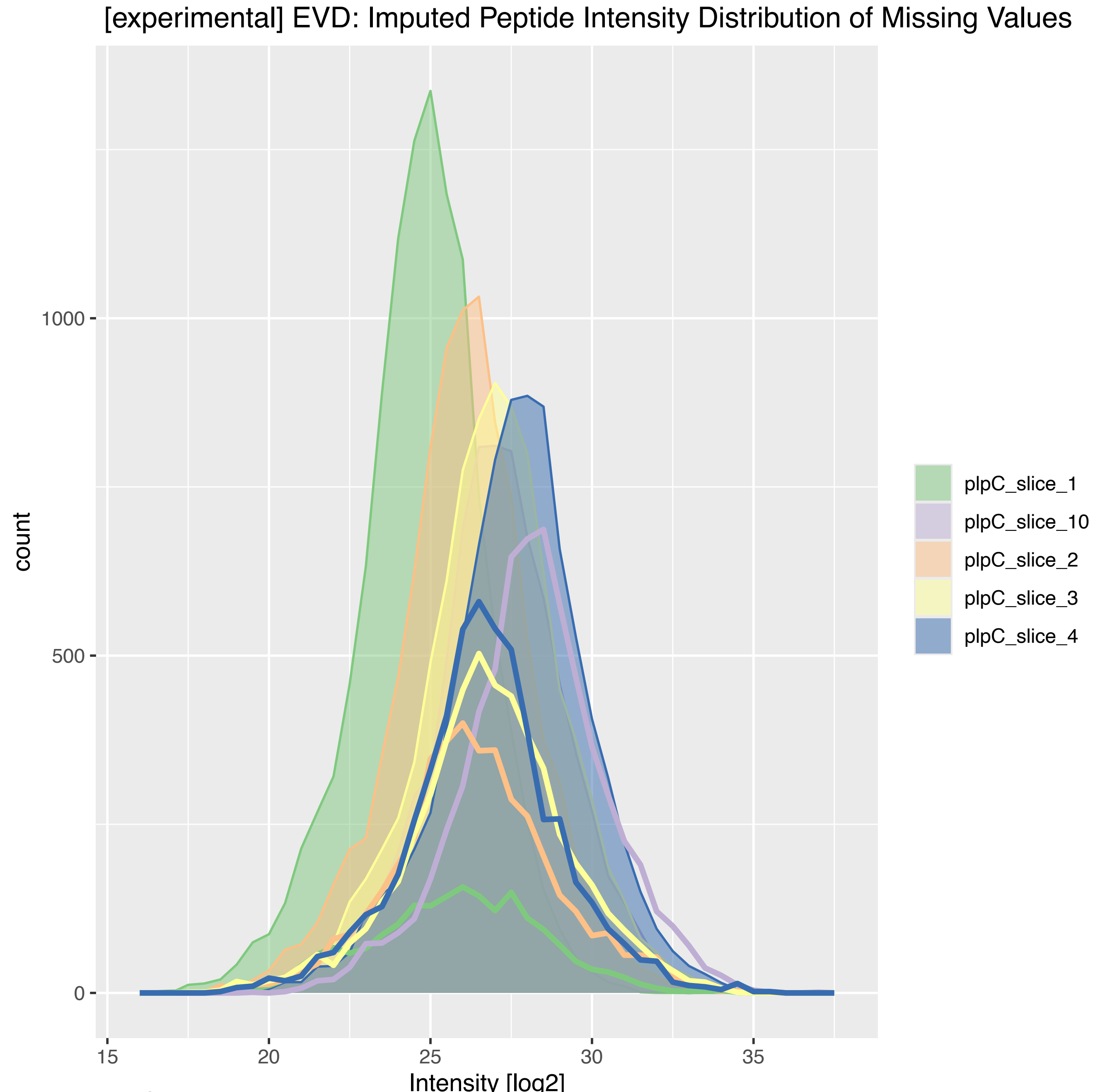
[experimental] EVD: Imputed Peptide Intensity Distribution of Missing Values
count
1000
500
0
15
20
25
30
35
Intensity [log2]
plpC_slice_1
plpC_slice_10
plpC_slice_2
plpC_slice_3
plpC_slice_4

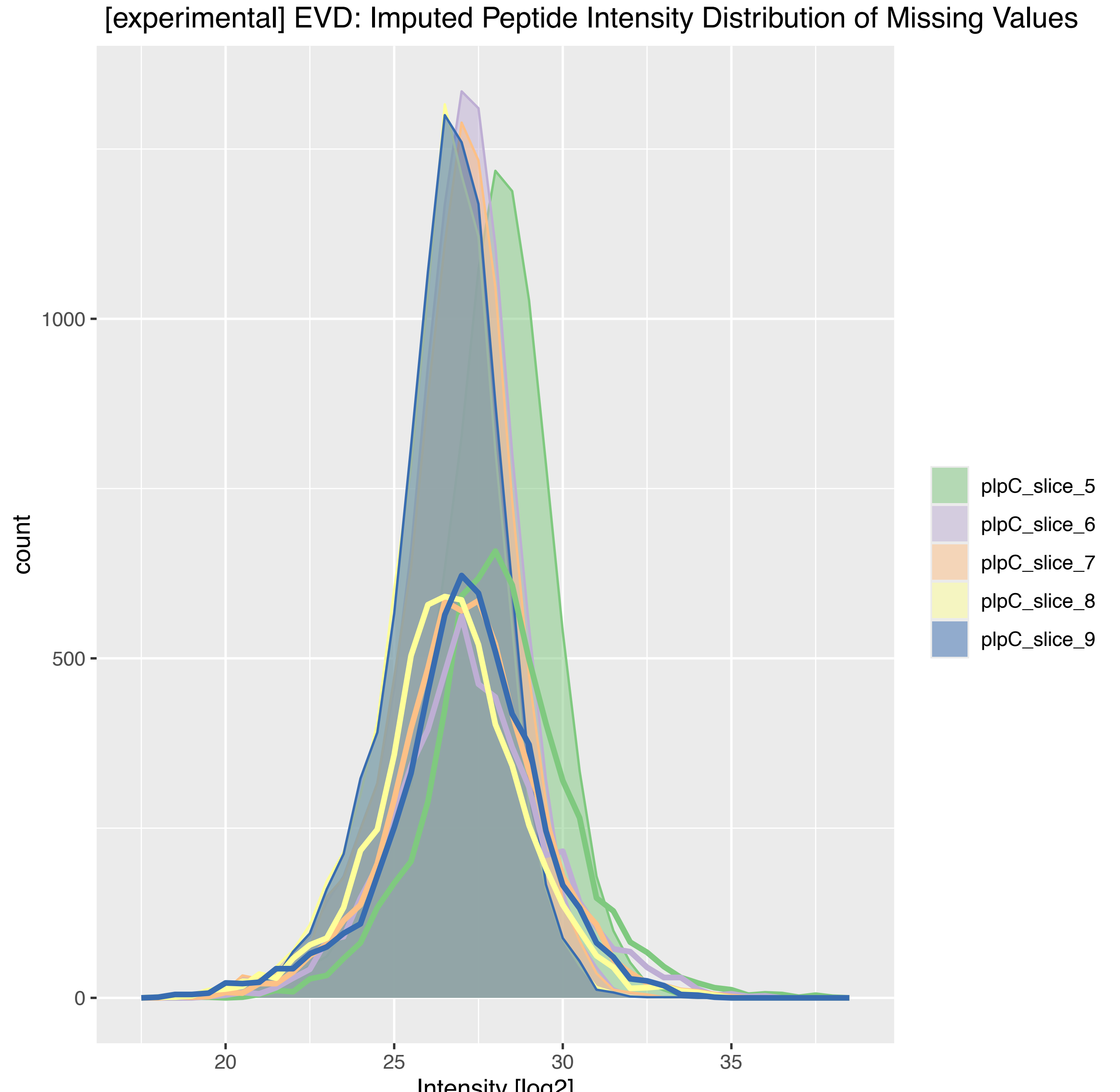
[experimental] EVD: Imputed Peptide Intensity Distribution of Missing Values
count
1000
500
0
20
25
30
35
Intensity [log2]
pIpC_slice_5
pIpC_slice_6
pIpC_slice_7
pIpC_slice_8
pIpC_slice_9

EVD: Peptide ID count

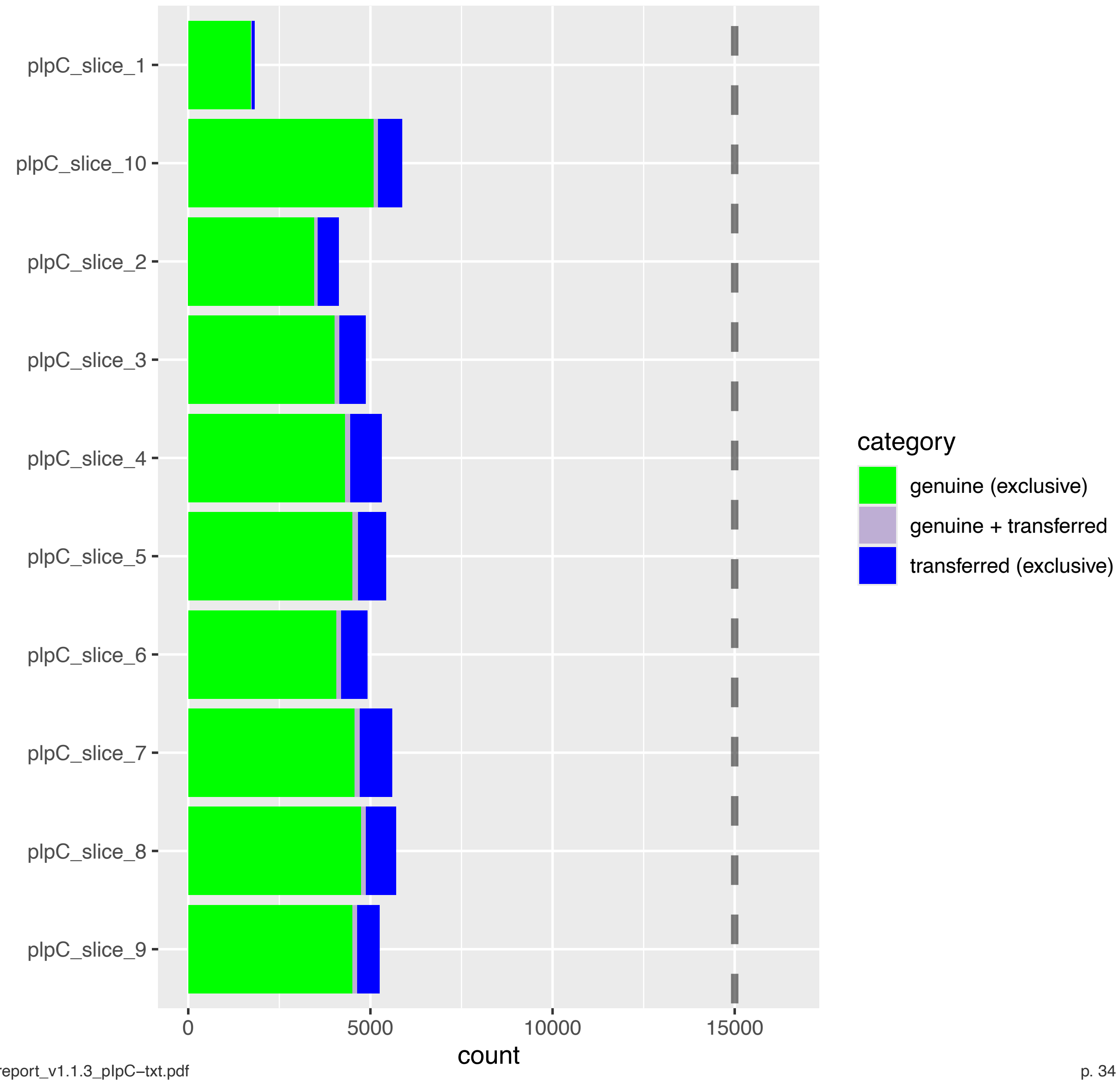

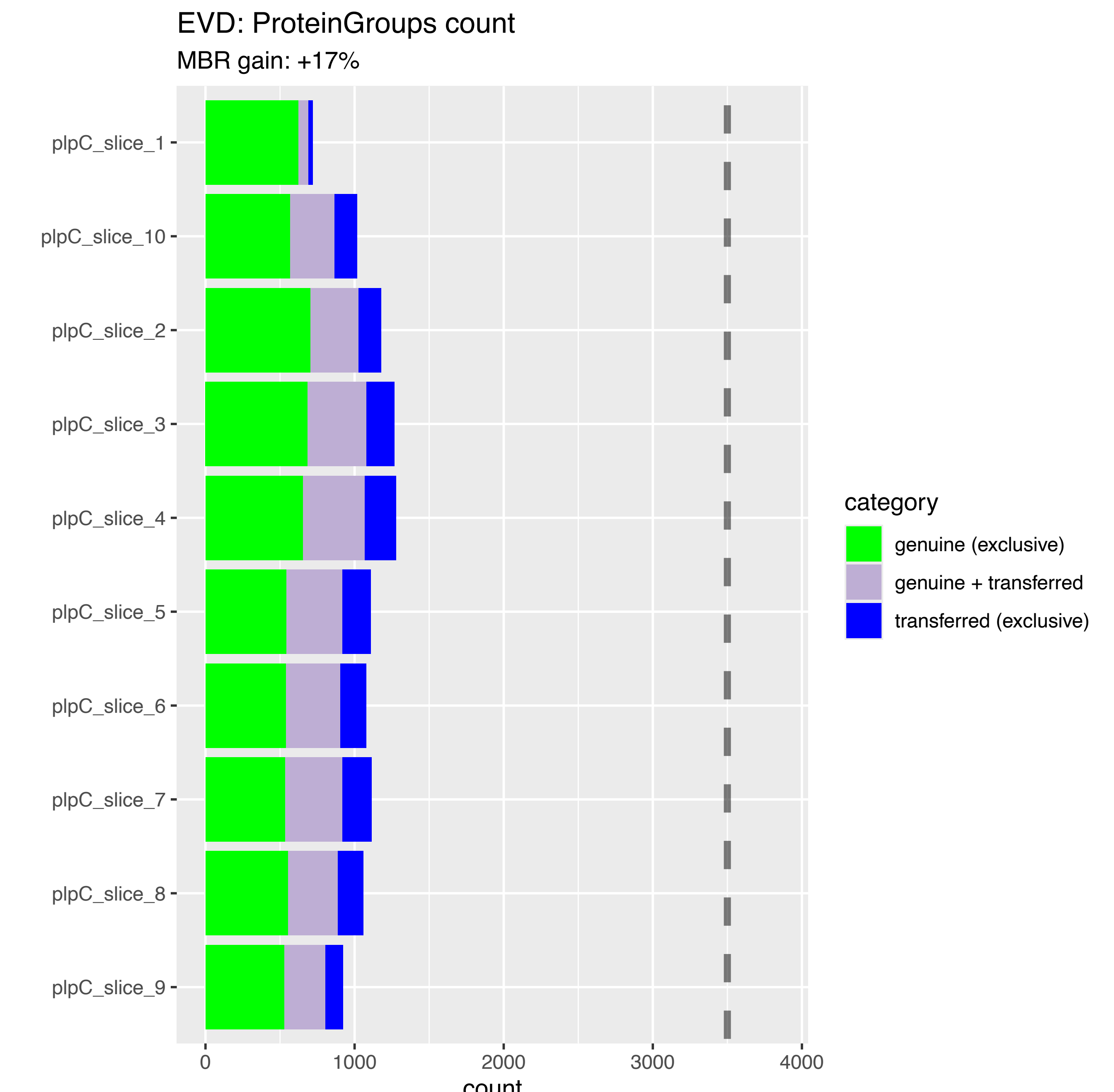

EVD: ProteinGroups count
MBR gain: +17%
plpC_slice_1
plpC_slice_10
plpC_slice_2
plpC_slice_3
plpC_slice_4
plpC_slice_5
plpC_slice_6
plpC_slice_7
plpC_slice_8
plpC_slice_9
0
1000
2000
3000
4000
count
category
genuine (exclusive)
genuine + transferred
transferred (exclusive)

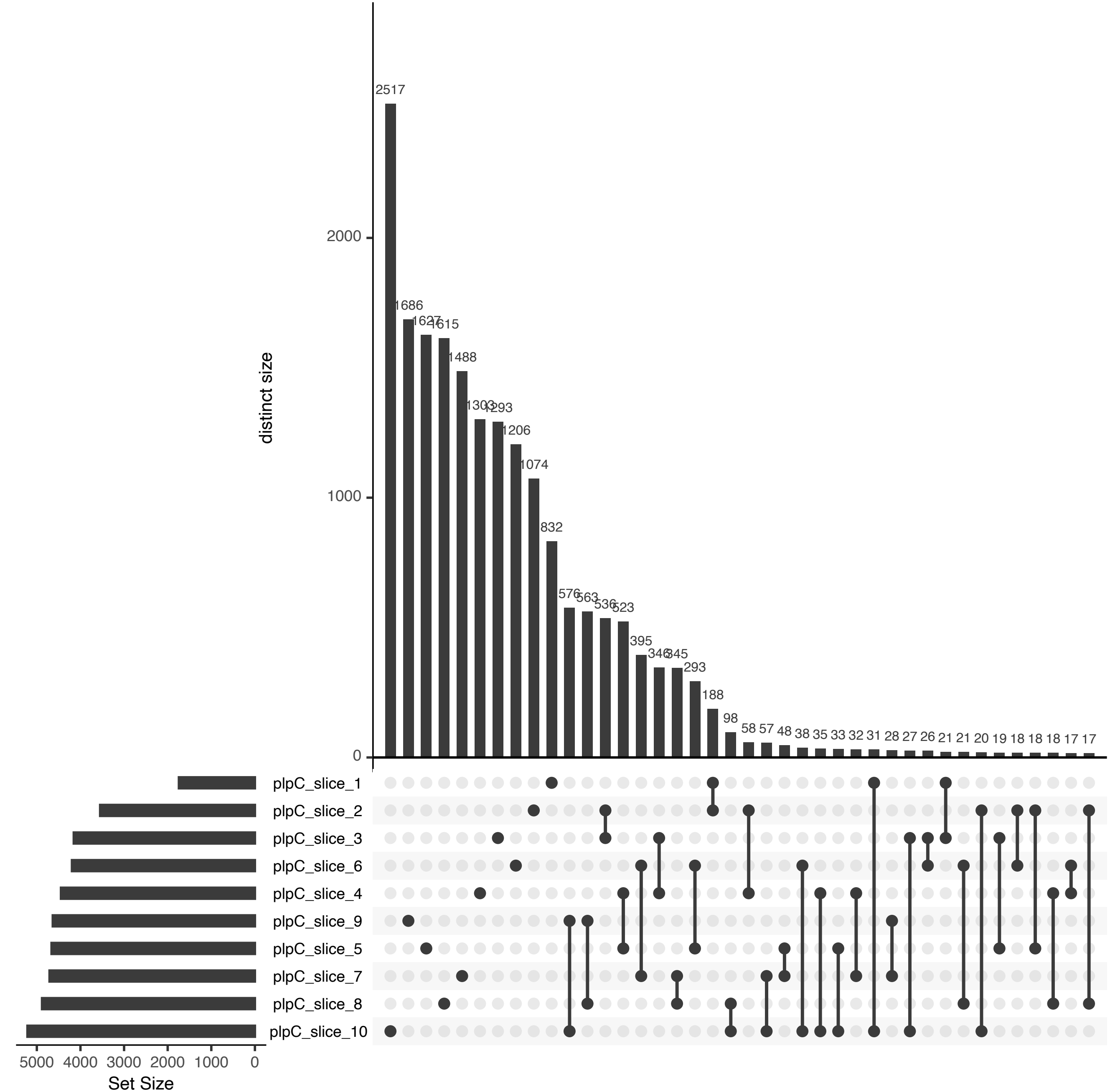
distinct size
2000
1000
0
2517
1686
1627
1615
1488
1303
1293
1206
1074
832
576
563
536
523
395
346
345
293
188
98
58
57
48
38
35
33
32
31
28
27
26
21
21
20
19
18
18
18
17
17
plpC_slice_1
plpC_slice_2
plpC_slice_3
plpC_slice_6
plpC_slice_4
plpC_slice_9
plpC_slice_5
plpC_slice_7
plpC_slice_8
plpC_slice_10
5000
4000
3000
2000
1000
0
Set Size

IBDV

Performance overview

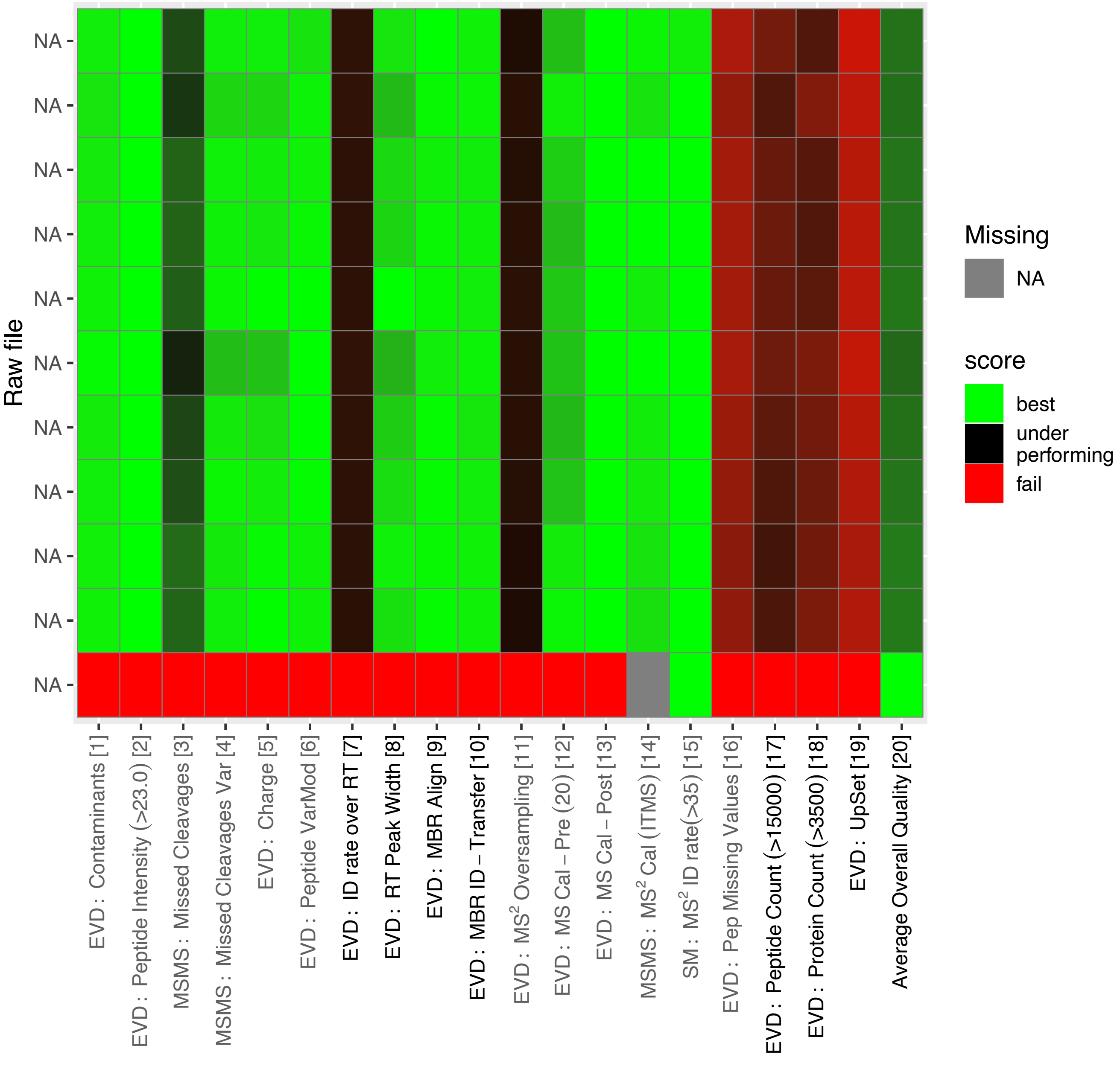

Mapping of Raw files to their short names
Mapping source: automatic

original	short name	best effort
PBG98_slice_1	PBG98_slice_1	PBG98_slice_1
PBG98_slice_10	PBG98_slice_10	PBG98_slice_10
PBG98_Slice_2	PBG98_Slice_2	PBG98_Slice_2
PBG98_slice_3	PBG98_slice_3	PBG98_slice_3
PBG98_slice_4	PBG98_slice_4	PBG98_slice_4
PBG98_slice_5	PBG98_slice_5	PBG98_slice_5
PBG98_slice_6	PBG98_slice_6	PBG98_slice_6
PBG98_slice_7	PBG98_slice_7	PBG98_slice_7
PBG98_slice_8	PBG98_slice_8	PBG98_slice_8
PBG98_slice_9	PBG98_slice_9	PBG98_slice_9
Total	Total	Total

PAR: parameters

parameter	value	parameter	value
Advanced ratios	True	MS/MS deisotoping tolerance ..	7
Advanced site intensities	True	MS/MS deisotoping tolerance ..	ppm
Alignment ion mobility windo..	1	MS/MS dependent losses (ASTR..	True
Alignment time window [min]	20	MS/MS dependent losses (FTMS..	True
Calculate peak properties	False	MS/MS dependent losses (ITMS..	True
Da interval. (ASTRAL)	100	MS/MS dependent losses (TOF)	True
Da interval. (FTMS)	100	MS/MS dependent losses (UNKN..	True
Da interval. (ITMS)	100	MS/MS higher charges (ASTRAL..	True
Da interval. (TOF)	100	MS/MS higher charges (FTMS)	True
Da interval. (UNKNOWN)	100	MS/MS higher charges (ITMS)	True
Date of writing	04/16/2025 04:33:08	MS/MS higher charges (TOF)	True
Decoy mode	revert	MS/MS higher charges (UNKNOW..	True
Disable MD5	False	MS/MS recalibration (ASTRAL)	False
Discard unmodified counterpa..	True	MS/MS recalibration (FTMS)	False
Epsilon score for mutations	True	MS/MS recalibration (ITMS)	False
Evaluate variant peptides se..	True	MS/MS recalibration (TOF)	False
Find dependent peptides	False	MS/MS recalibration (UNKNOWN..	False
Fixed andromeda index folder		MS/MS tol. (ASTRAL)	25 ppm
iBAQ	True	MS/MS tol. (FTMS)	20 ppm
iBAQ log fit	True	MS/MS tol. (ITMS)	0.5 Da
Include contaminants	True	MS/MS tol. (TOF)	25 ppm
Label min. ratio count	2	MS/MS tol. (UNKNOWN)	20 ppm

protein.faa

PAR: parameters

parameter	value	parameter	value
Machine name	DESKTOP−SUMVKIL	MS/MS water loss (ASTRAL for..	False
Main search max. combination..	200	MS/MS water loss (ASTRAL)	True
Match between runs	True	MS/MS water loss (FTMS for c..	False
Match ion mobility window [i..	0.05	MS/MS water loss (FTMS)	True
Match unidentified features	True	MS/MS water loss (ITMS for c..	False
Matching time window [min]	0.8	MS/MS water loss (ITMS)	True
Max mods in site table	3	MS/MS water loss (TOF for cr..	False
Max. peptide length for unsp..	25	MS/MS water loss (TOF)	True
Max. peptide mass [Da]	4600	MS/MS water loss (UNKNOWN fo..	False
Min. delta score for modifie..	6	MS/MS water loss (UNKNOWN)	True
Min. delta score for unmodif..	0	Peptides used for protein qu..	Razor
Min. peptide Length	7	Protein FDR	0.01
Min. peptide length for unsp..	8	PSM FDR	0.01
Min. peptides	1	PSM FDR Crosslink	0.01
Min. razor peptides	1	Razor protein FDR	True
Min. score for modified pept..	40	Require MS/MS for LFQ compar..	True
Min. score for unmodified pe..	0	Second peptides	True
Min. unique peptides	0	Separate LFQ in parameter gr..	False
Modifications included in pr..	Oxidation (M)<br>Acetyl (Protein N−term)	Site FDR	0.01
MS/MS ammonia loss (ASTRAL f..	False	Site tables	Oxidation (M)Sites.txt
MS/MS ammonia loss (ASTRAL)	True	Stabilize large LFQ ratios	True
MS/MS ammonia loss (FTMS for..	False	Top MS/MS peaks per Da inter..	12

protein.faa

PAR: parameters

parameter	value	parameter	value
MS/MS ammonia loss (FTMS)	True	Top MS/MS peaks per Da inter..	8
MS/MS ammonia loss (ITMS for..	False	Top MS/MS peaks per Da inter..	16
MS/MS ammonia loss (ITMS)	True	Top MS/MS peaks per Da inter..	16
MS/MS ammonia loss (TOF for ..	False	Top MS/MS peaks per Da inter..	12
MS/MS ammonia loss (TOF)	True	Use delta score	False
MS/MS ammonia loss (UNKNOWN ..	False	Use Normalized Ratios For Oc..	True
MS/MS ammonia loss (UNKNOWN)	True	Use only unmodified peptides..	True
MS/MS deisotoping (ASTRAL)	True	User name	tosor
MS/MS deisotoping (FTMS)	True	Variation mode	None
MS/MS deisotoping (ITMS)	False	Version	2.6.5.0
MS/MS deisotoping (TOF)	True	Write accumulatedMsmsScans t..	False
MS/MS deisotoping (UNKNOWN)	True	Write allPeptides table	False
MS/MS deisotoping tolerance ..	7	Write DIA fragments quant ta..	False
MS/MS deisotoping tolerance ..	ppm	Write DIA fragments table	False
MS/MS deisotoping tolerance ..	0.15	Write ms3Scans table	False
MS/MS deisotoping tolerance ..	Da	Write msmsScans table	False
MS/MS deisotoping tolerance ..	0.01	Write msScans table	False
MS/MS deisotoping tolerance ..	Da	Write mzRange table	False
MS/MS deisotoping tolerance ..	0.01	Write pasefMsmsScans table	False
MS/MS deisotoping tolerance ..	Da		

protein.faa

PG: PCA of 'raw intensity'

(excludes contaminants)

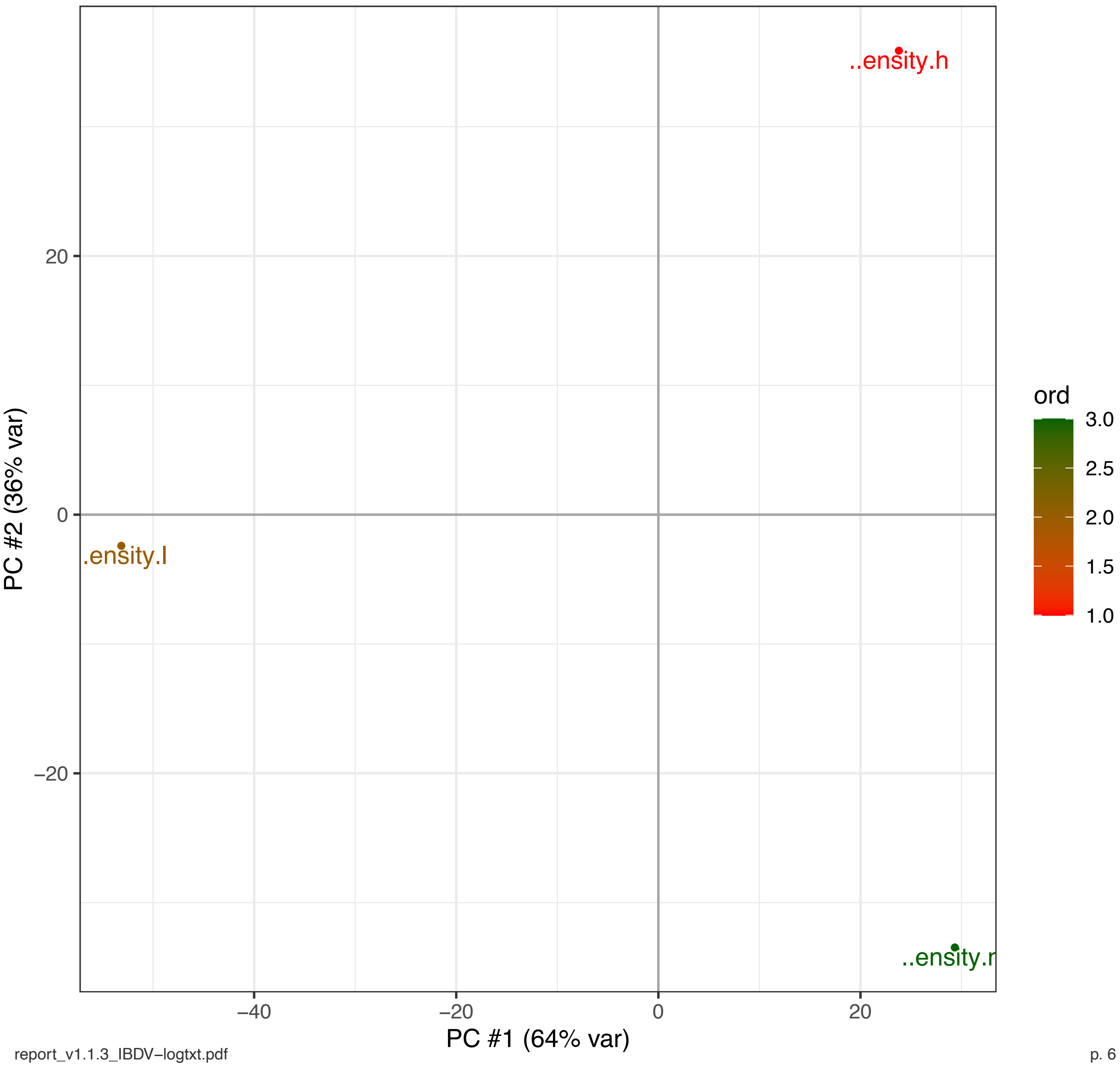

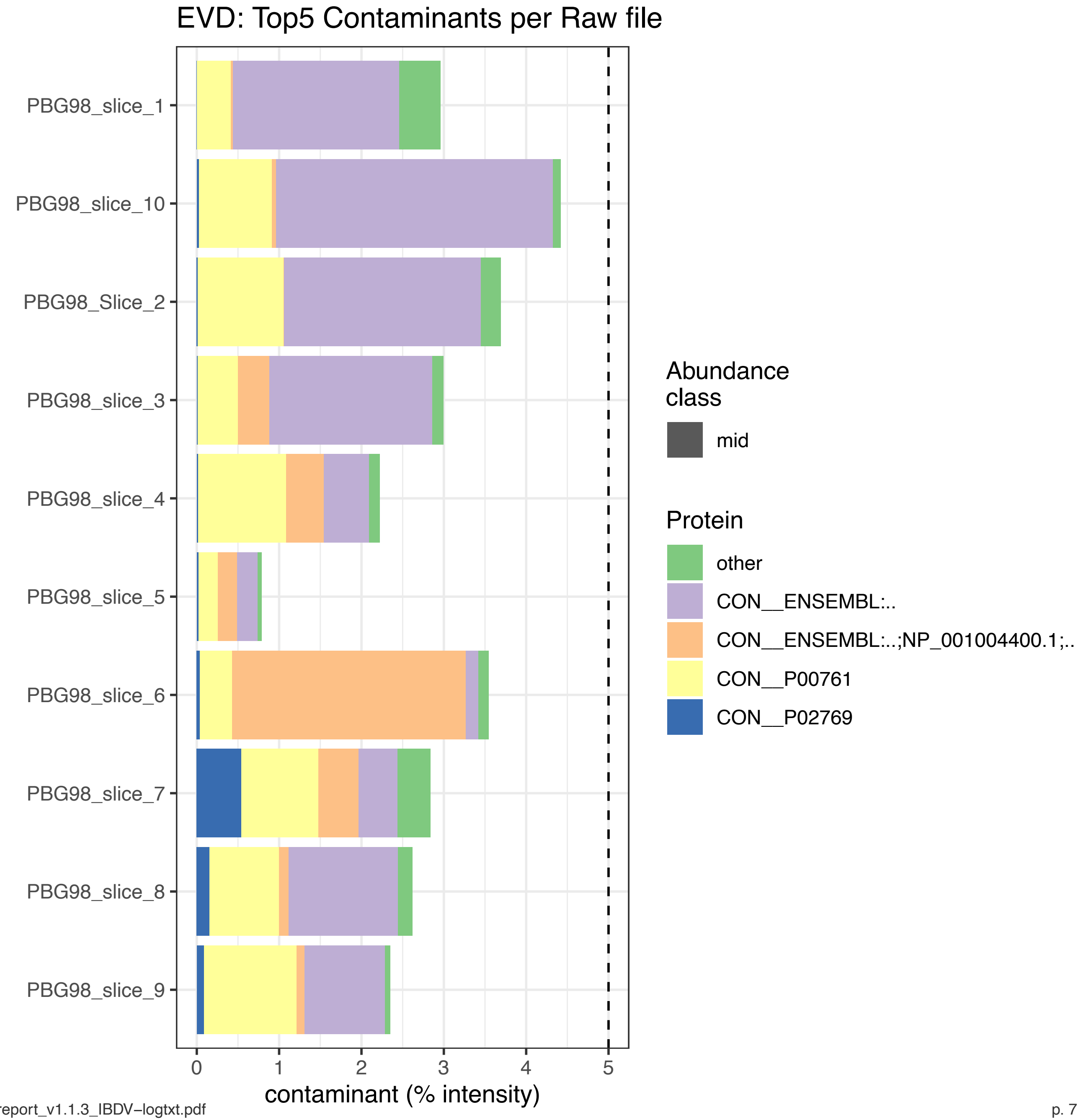
EVD: Top5 Contaminants per Raw file
PBG98_slice_1
PBG98_slice_10
PBG98_Slice_2
PBG98_slice_3
PBG98_slice_4
PBG98_slice_5
PBG98_slice_6
PBG98_slice_7
PBG98_slice_8
PBG98_slice_9
0
1
2
3
4
5
contaminant (% intensity)
Abundance class
mid
Protein
other
CON__ENSEMBL:..
CON__ENSEMBL:..;NP_001004400.1;..
CON__P00761
CON__P02769

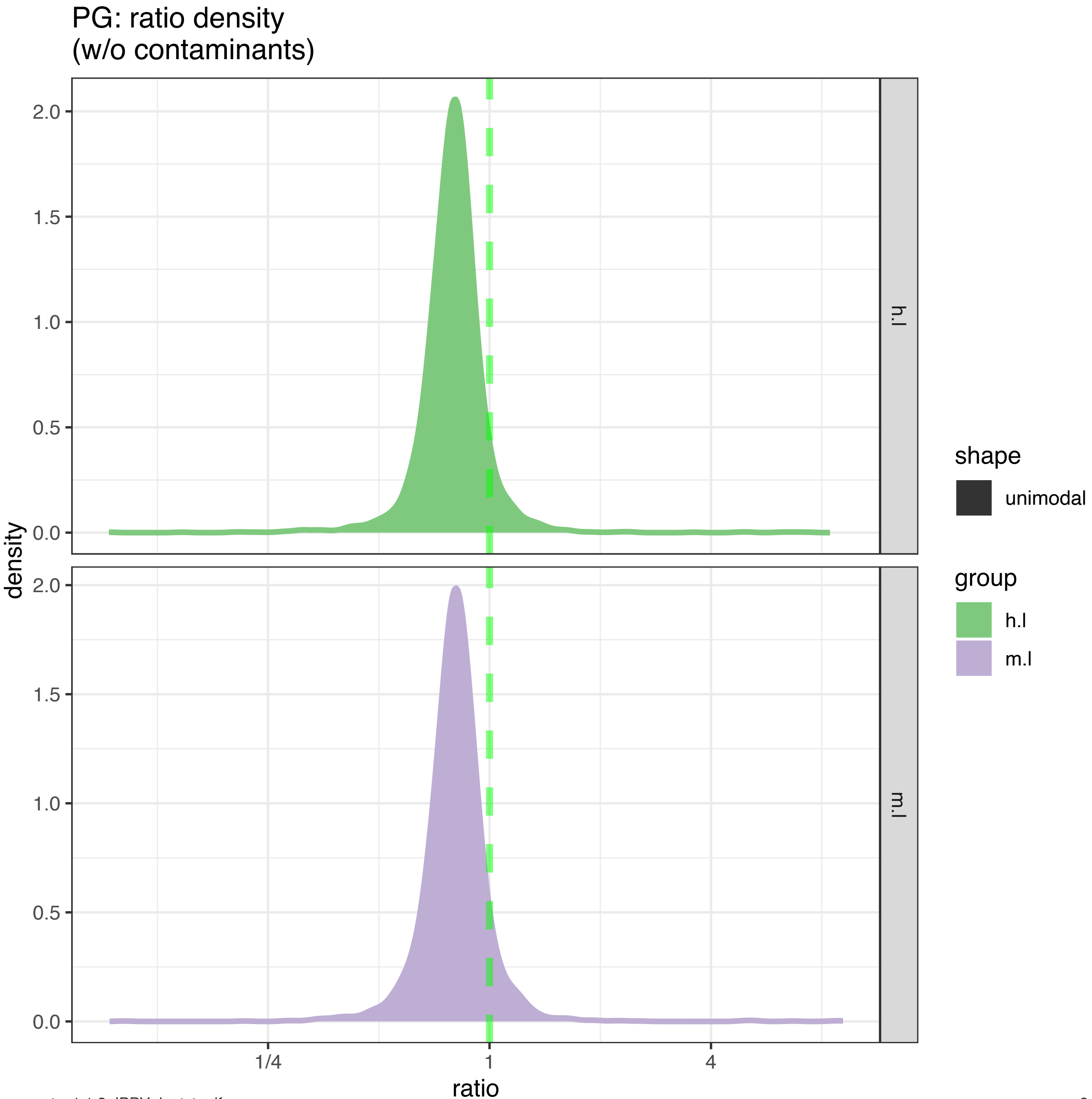

PG: ratio density
(w/o contaminants)
density
2.0
1.5
1.0
0.5
0.0
h.l
m.l
1/4
1
4
ratio
shape
unimodal
group
h.l
m.l

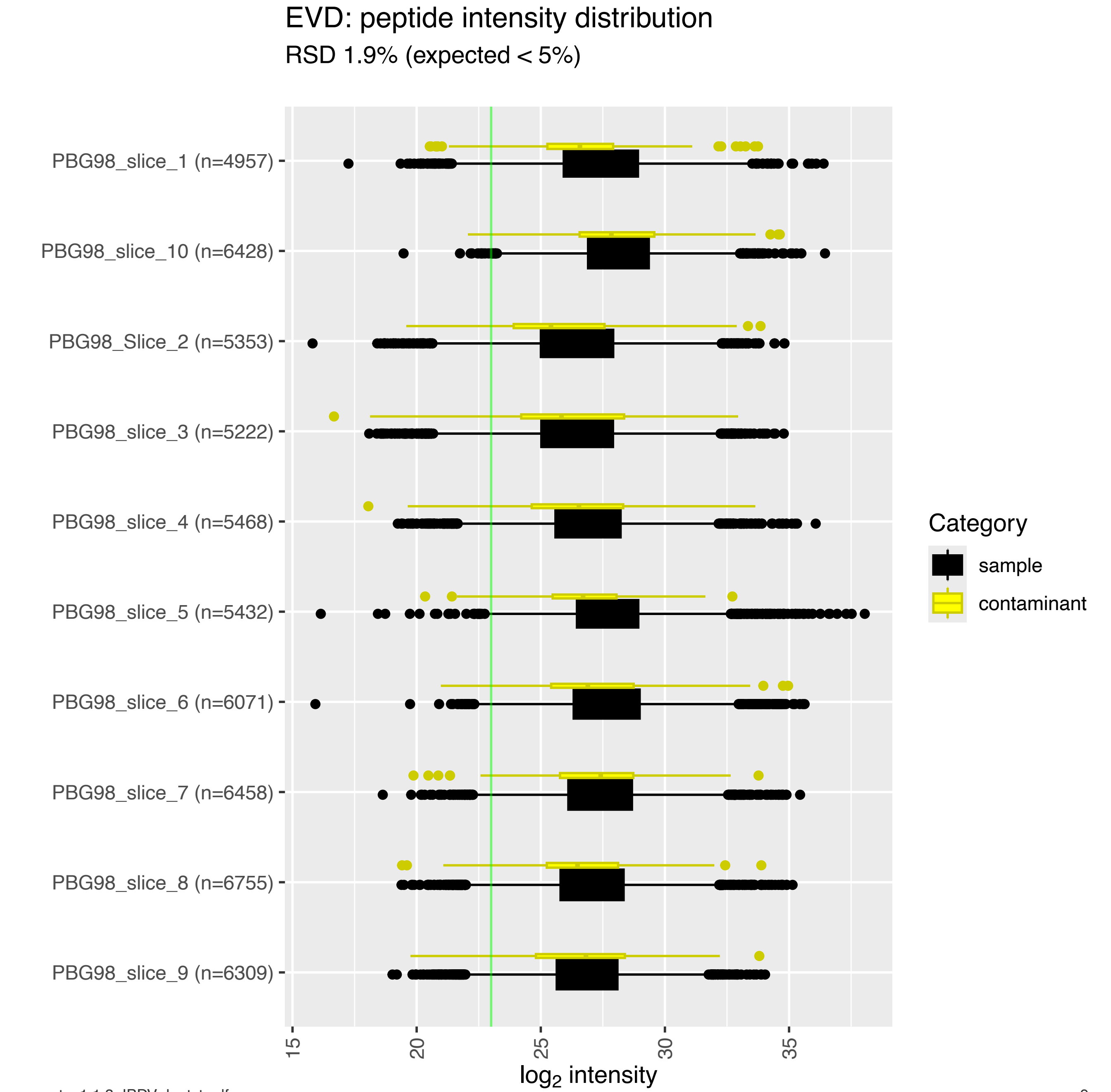

EVD: peptide intensity distribution
RSD 1.9% (expected < 5%)
PBG98_slice_1 (n=4957)
PBG98_slice_10 (n=6428)
PBG98_Slice_2 (n=5353)
PBG98_slice_3 (n=5222)
PBG98_slice_4 (n=5468)
PBG98_slice_5 (n=5432)
PBG98_slice_6 (n=6071)
PBG98_slice_7 (n=6458)
PBG98_slice_8 (n=6755)
PBG98_slice_9 (n=6309)
15
20
25
30
35
log2 intensity
Category
sample
contaminant

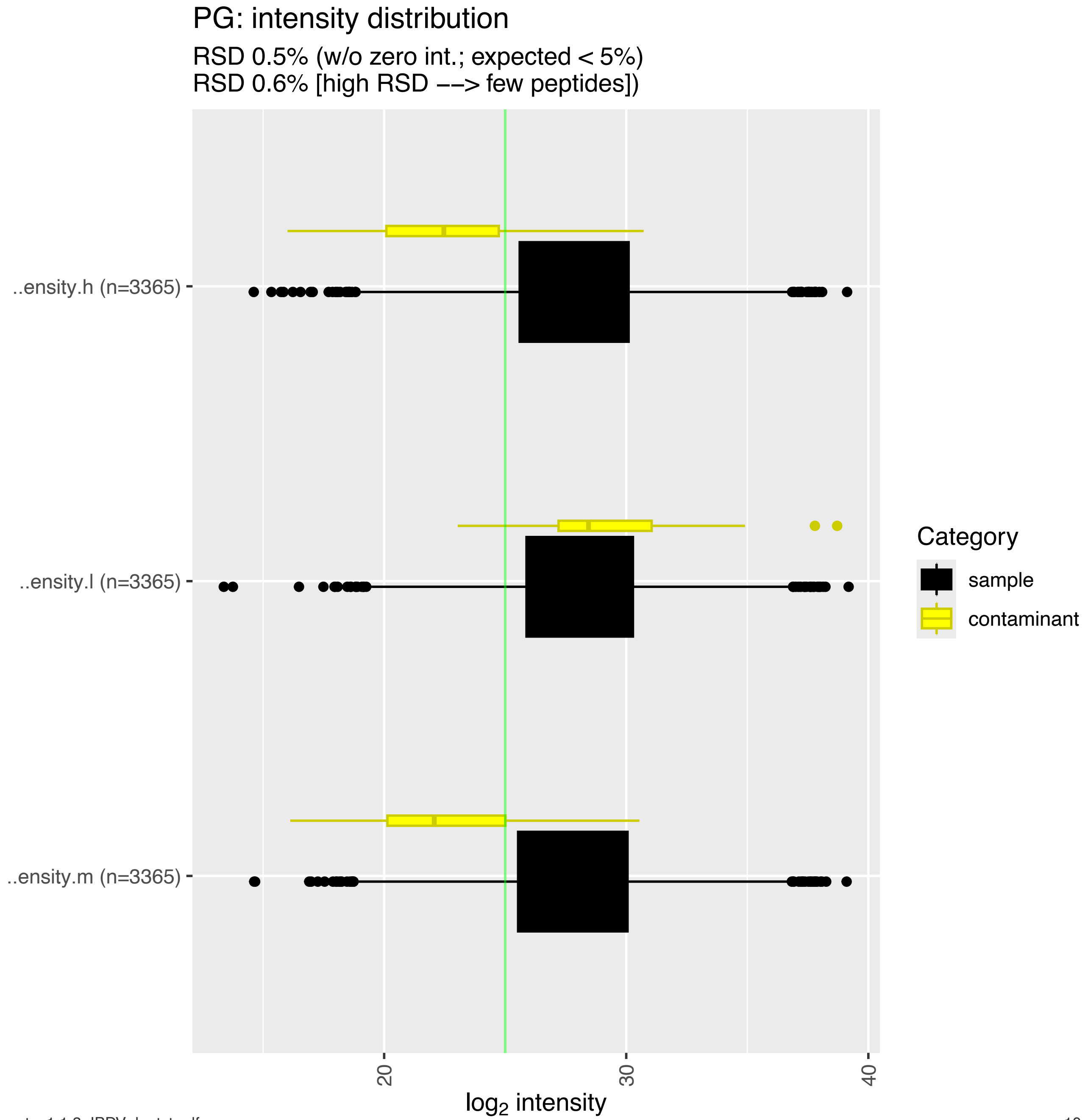
PG: intensity distribution
RSD 0.5% (w/o zero int.; expected < 5%)
RSD 0.6% [high RSD --> few peptides])
..ensity.h (n=3365)
..ensity.l (n=3365)
..ensity.m (n=3365)
20
30
40
log2 intensity
Category
sample
contaminant

MSMS: Missed cleavages per Raw file

(excludes contaminants)

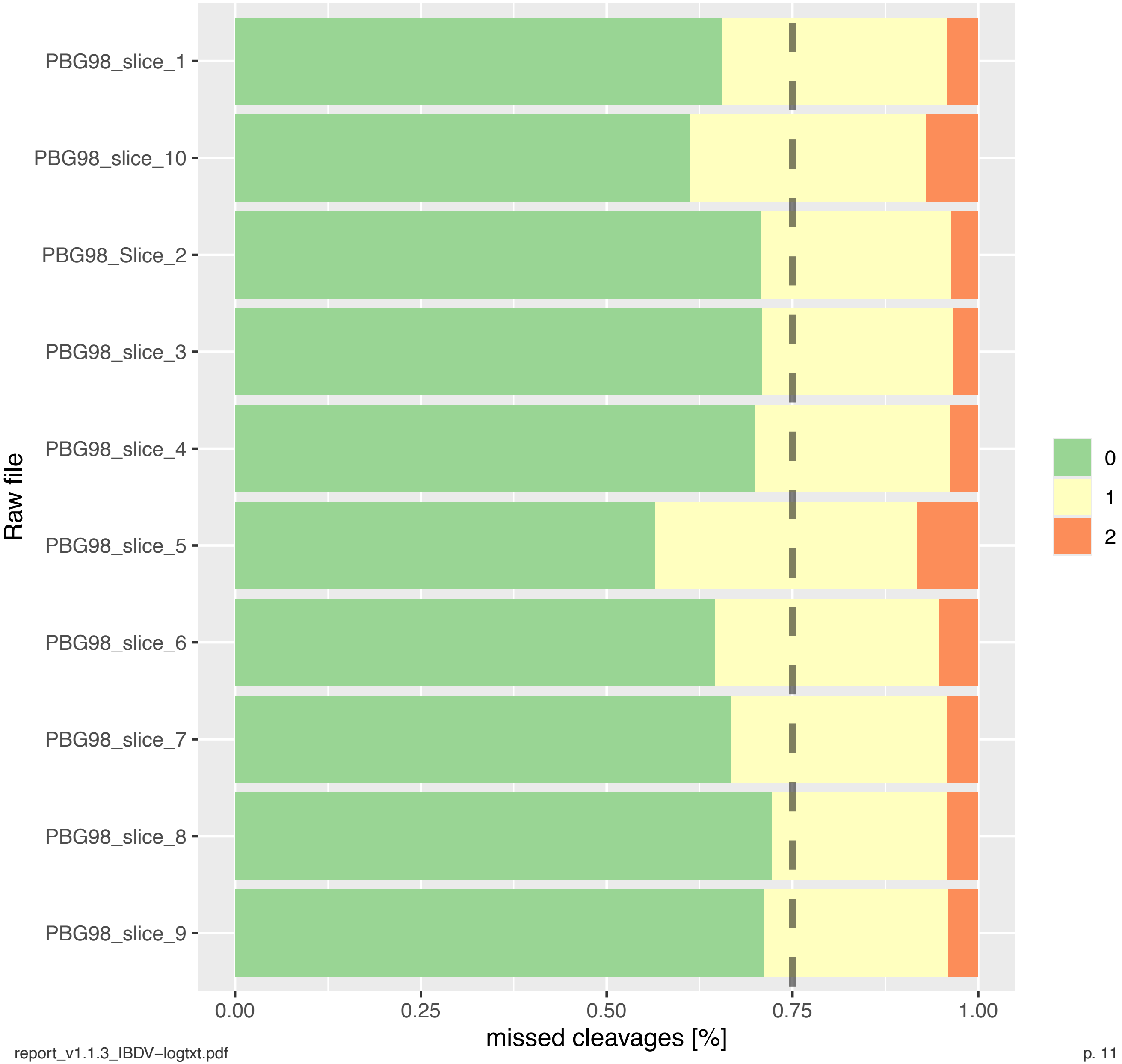

EVD: charge distribution

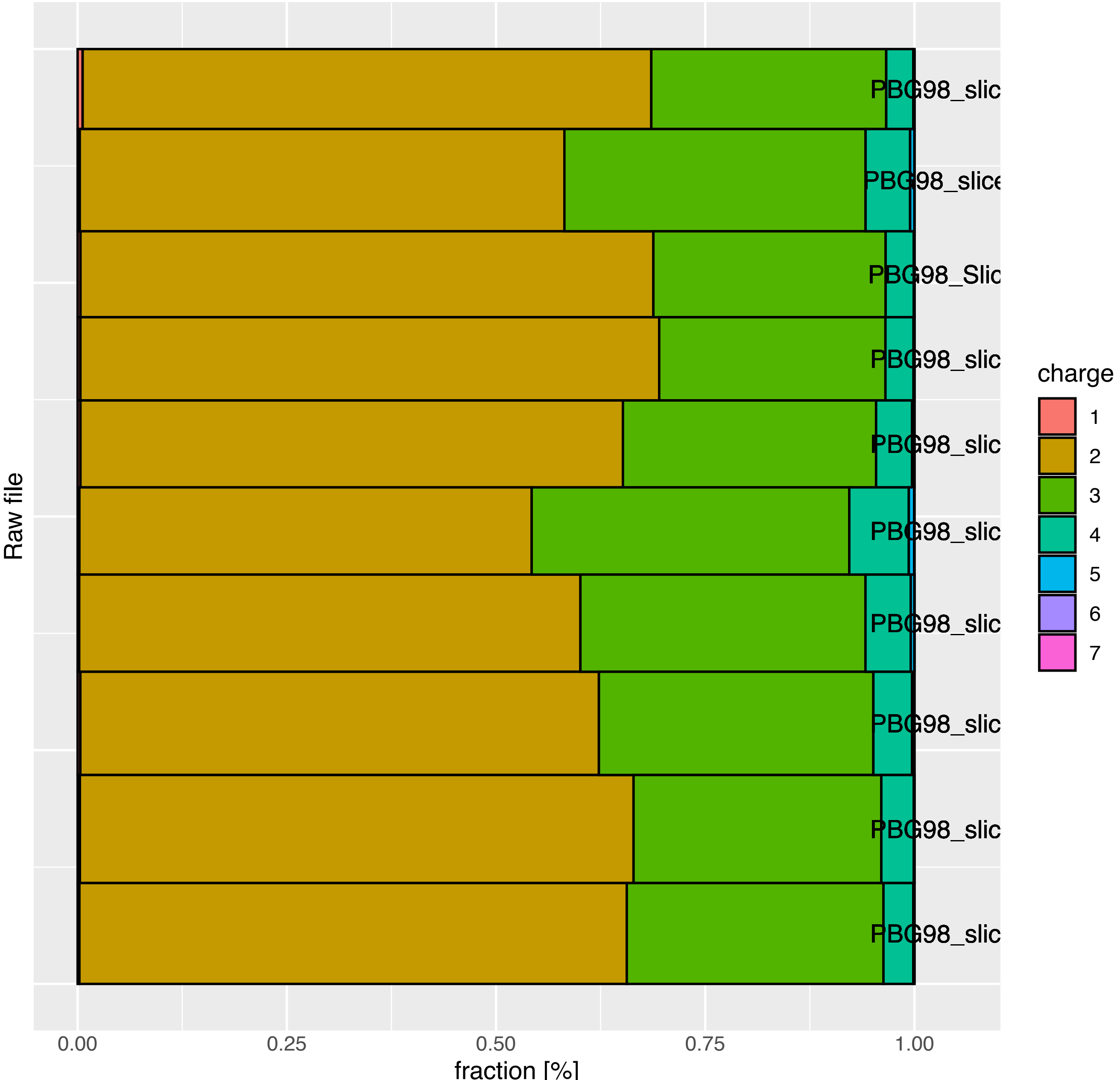

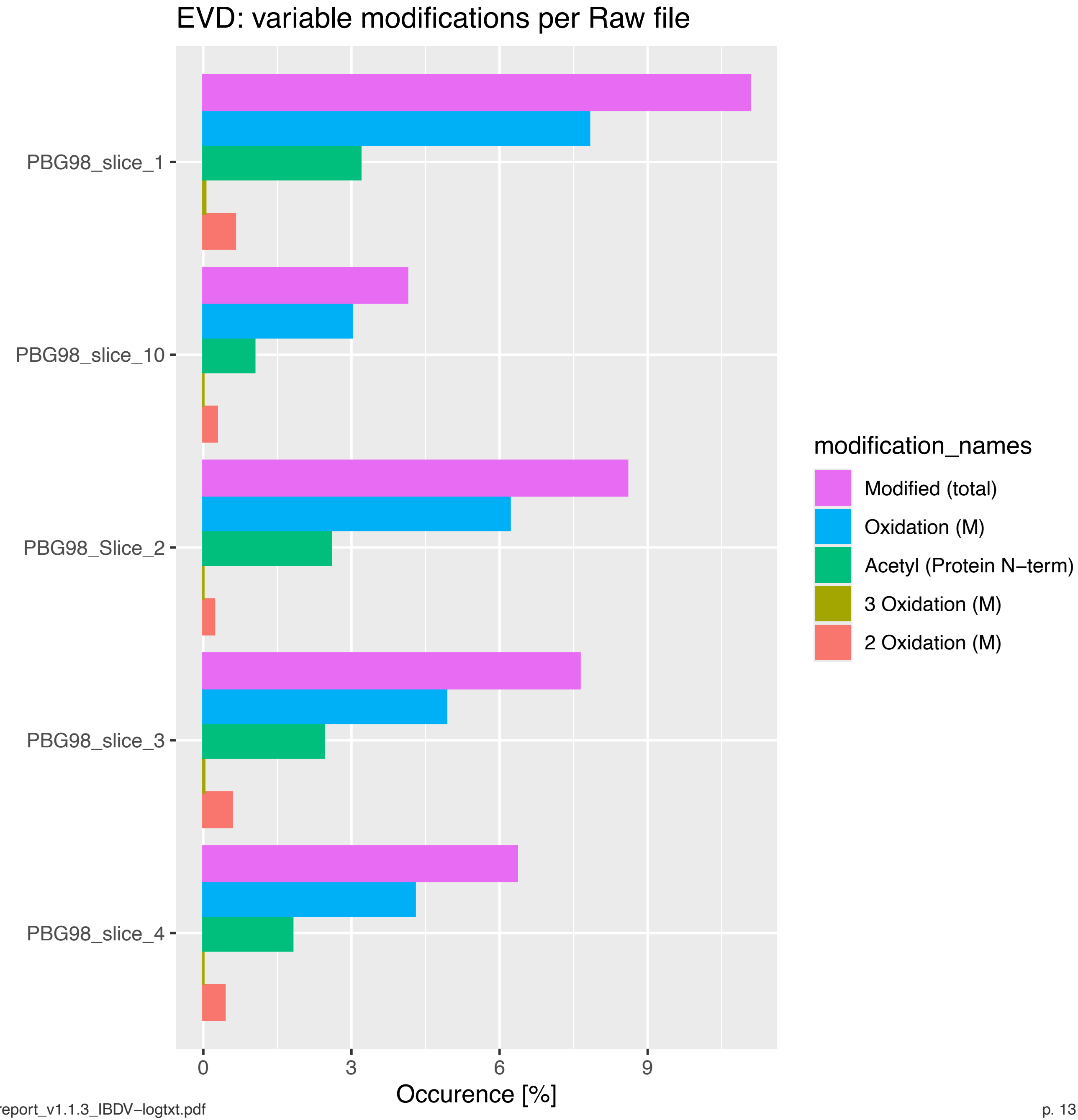

EVD: variable modifications per Raw file
PBG98_slice_1
PBG98_slice_10
PBG98_Slice_2
PBG98_slice_3
PBG98_slice_4
0
3
6
9
Occurence [%]
modification_names
Modified (total)
Oxidation (M)
Acetyl (Protein N−term)
3 Oxidation (M)
2 Oxidation (M)

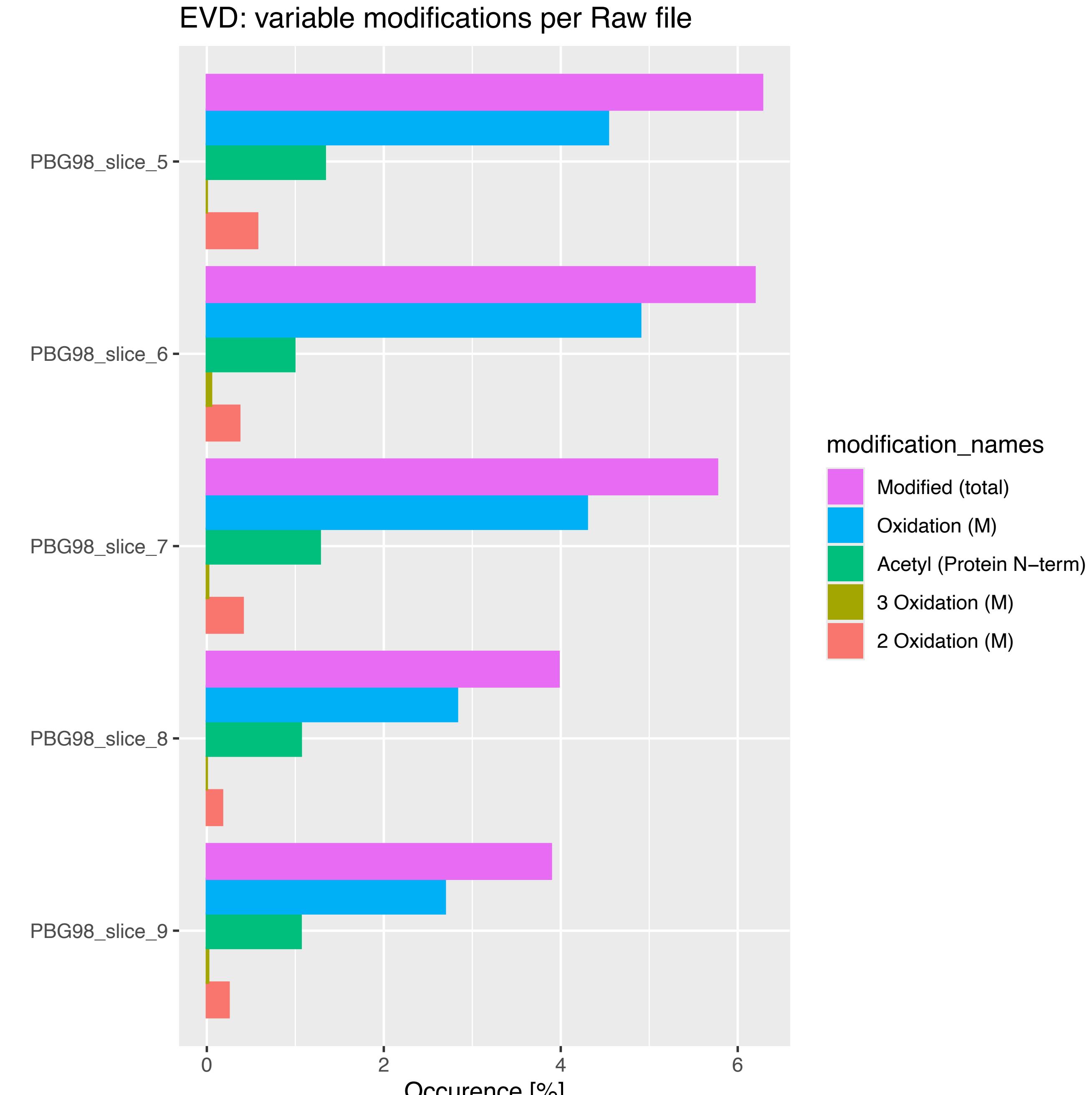
EVD: variable modifications per Raw file
PBG98_slice_5
PBG98_slice_6
PBG98_slice_7
PBG98_slice_8
PBG98_slice_9
0
2
4
6
Occurence [%]
modification_names
Modified (total)
Oxidation (M)
Acetyl (Protein N−term)
3 Oxidation (M)
2 Oxidation (M)

PG: Contaminant per condition

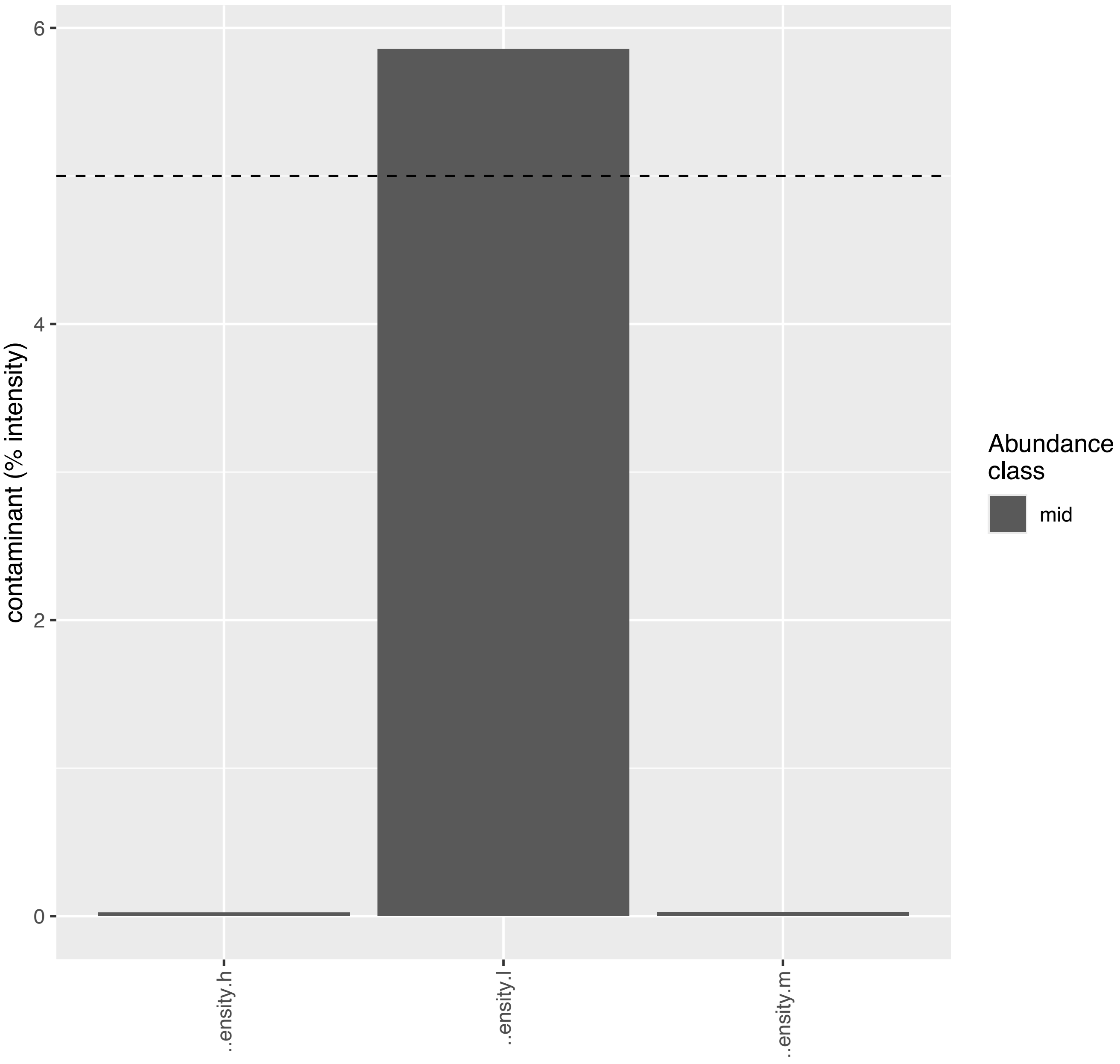

EVD: IDs over RT

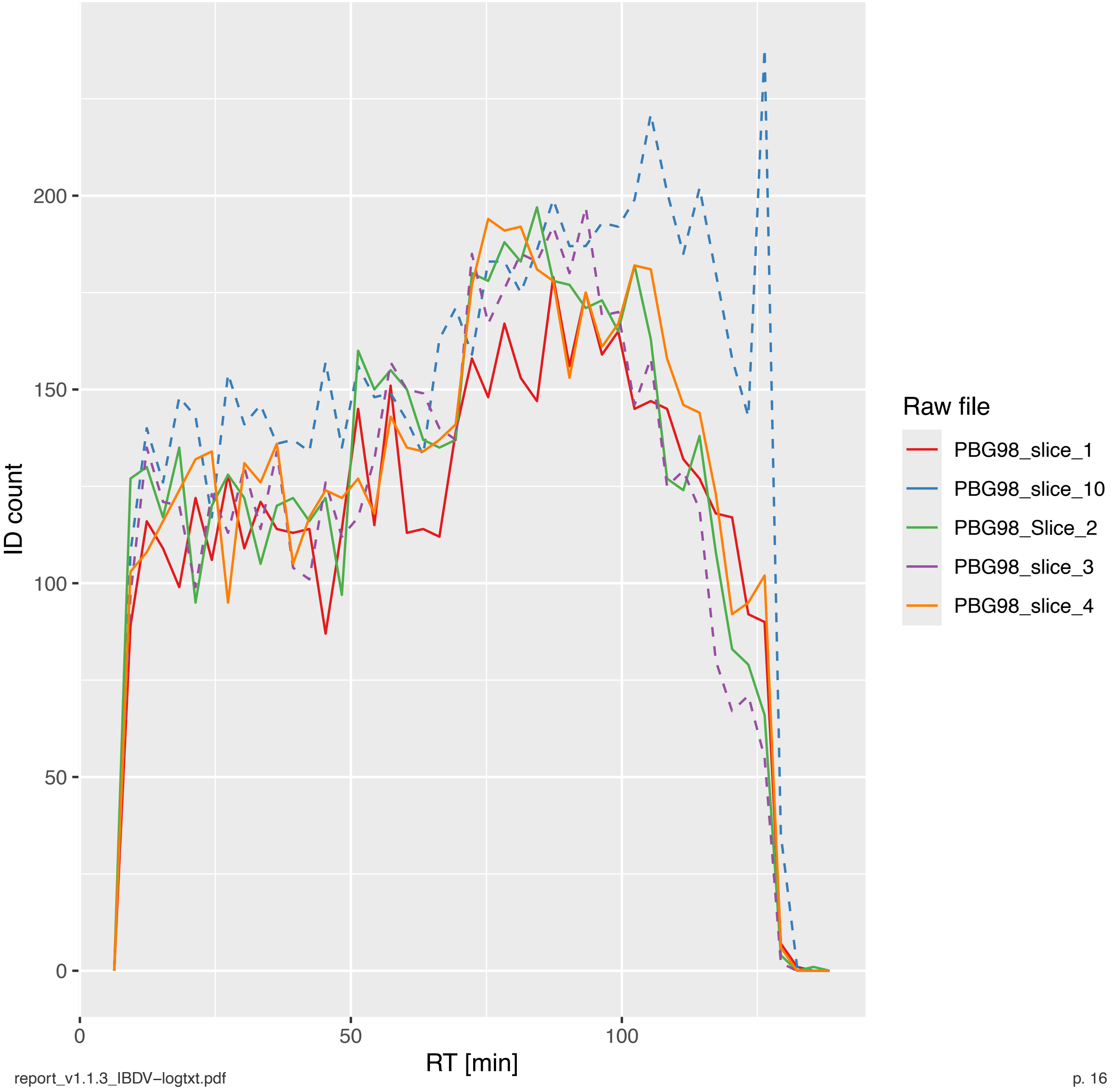

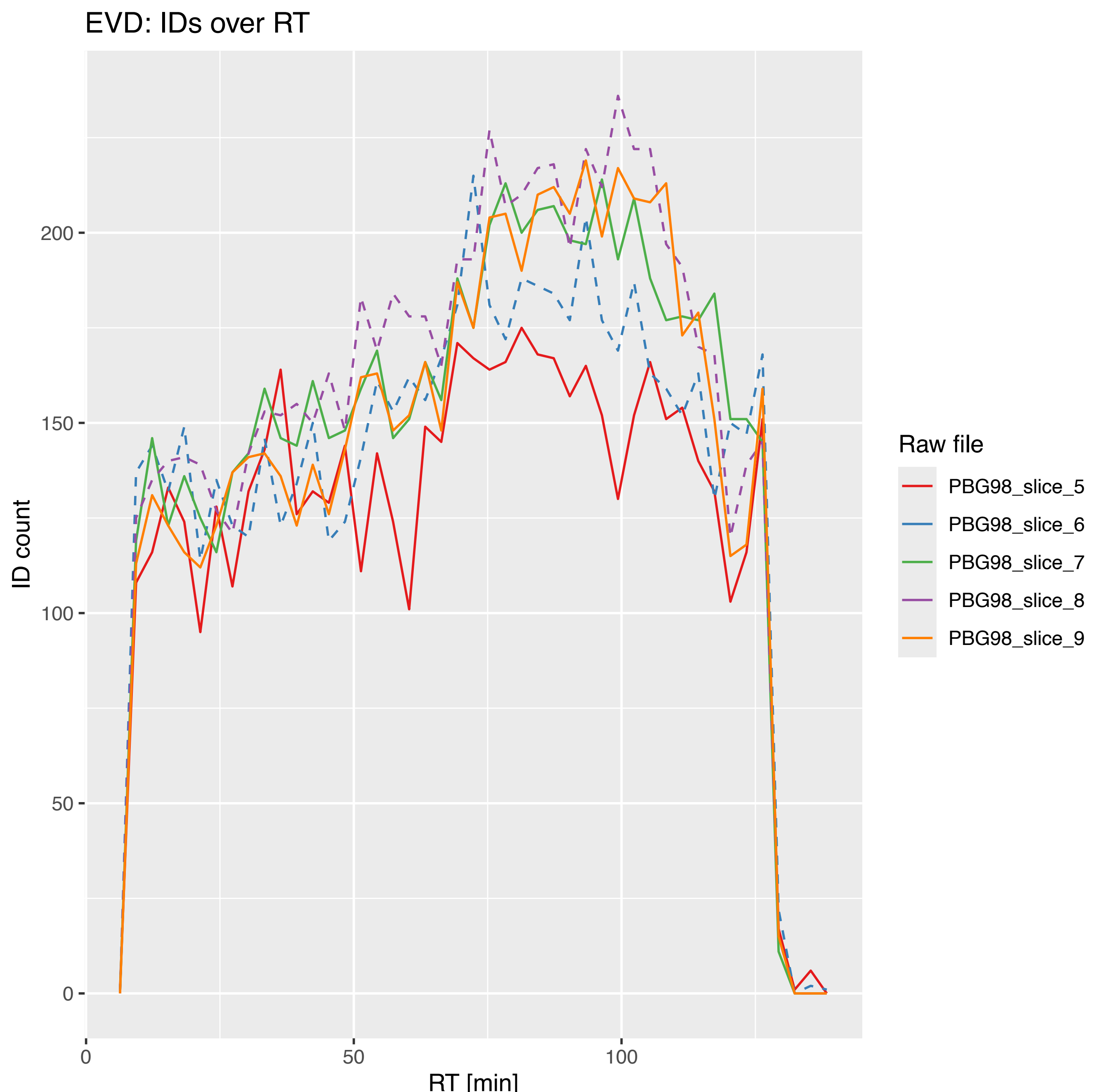
EVD: IDs over RT
ID count
RT [min]
0
50
100
150
200
Raw file
PBG98_slice_5
PBG98_slice_6
PBG98_slice_7
PBG98_slice_8
PBG98_slice_9

EVD: Peak width over RT

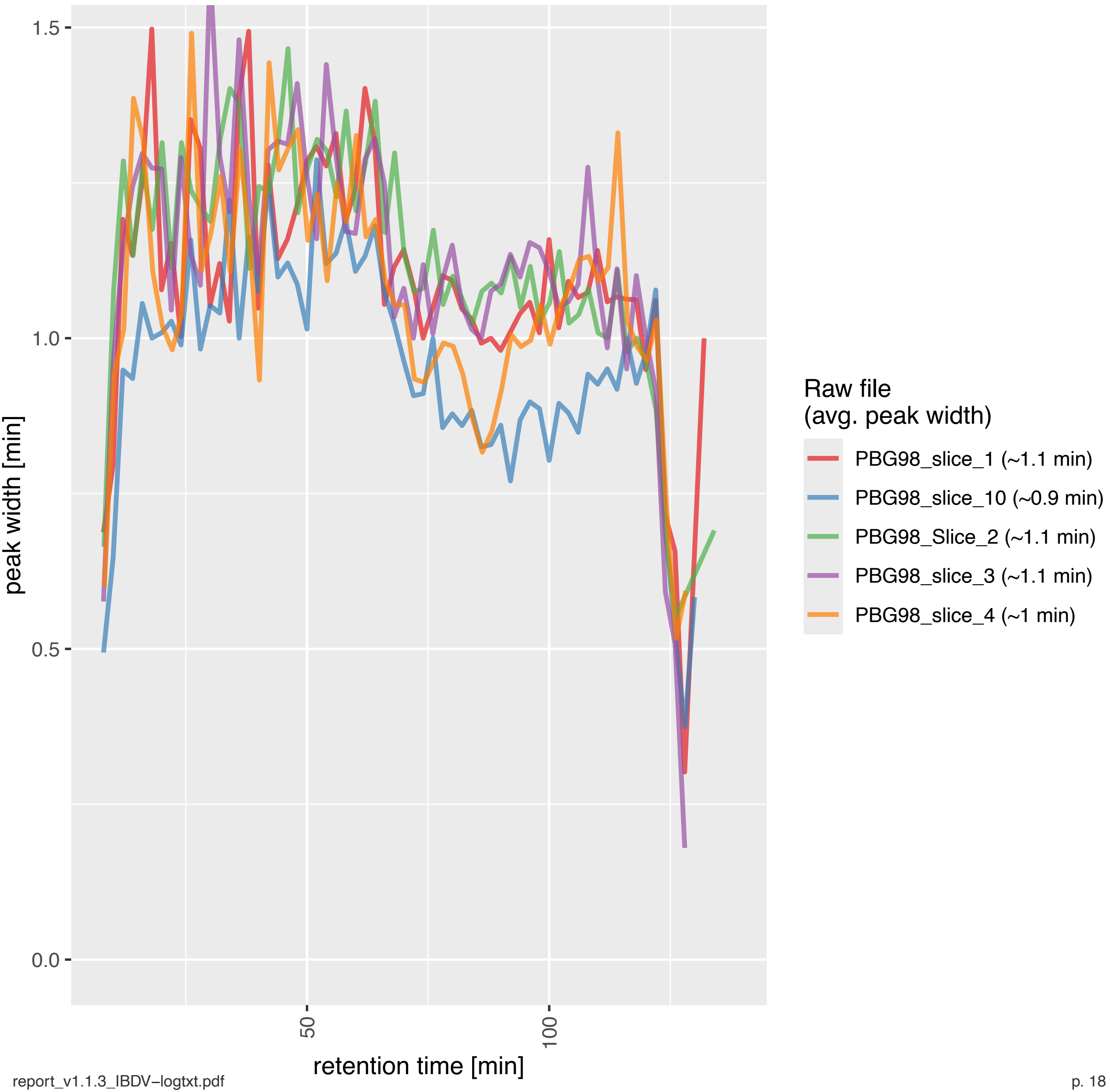

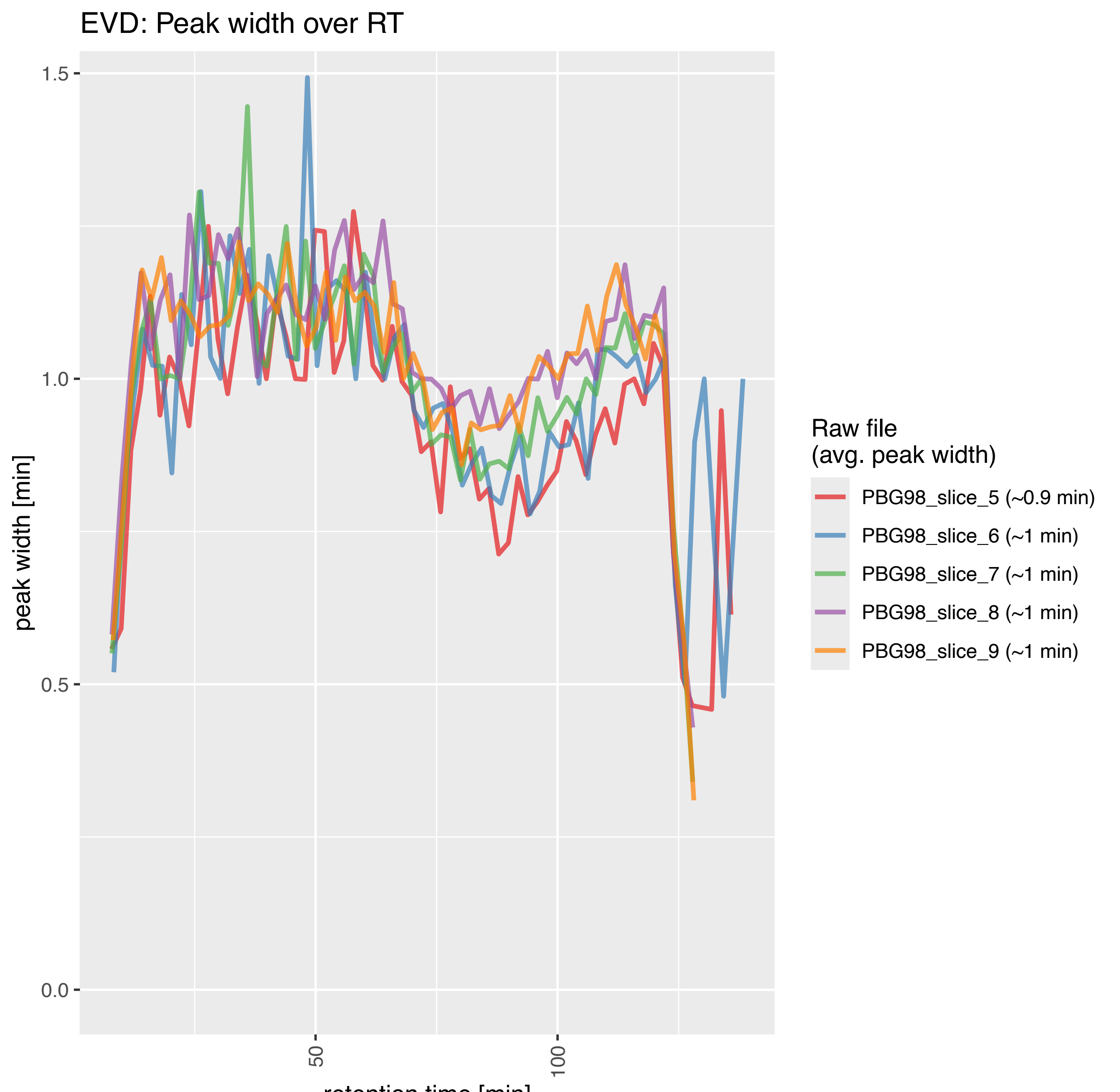
EVD: Peak width over RT
peak width [min]
retention time [min]
1.5
1.0
0.5
0.0
50
100
Raw file
(avg. peak width)
PBG98_slice_5 (~0.9 min)
PBG98_slice_6 (~1 min)
PBG98_slice_7 (~1 min)
PBG98_slice_8 (~1 min)
PBG98_slice_9 (~1 min)

EVD: MBR – alignment

alignment reference: PBG98_slice_1

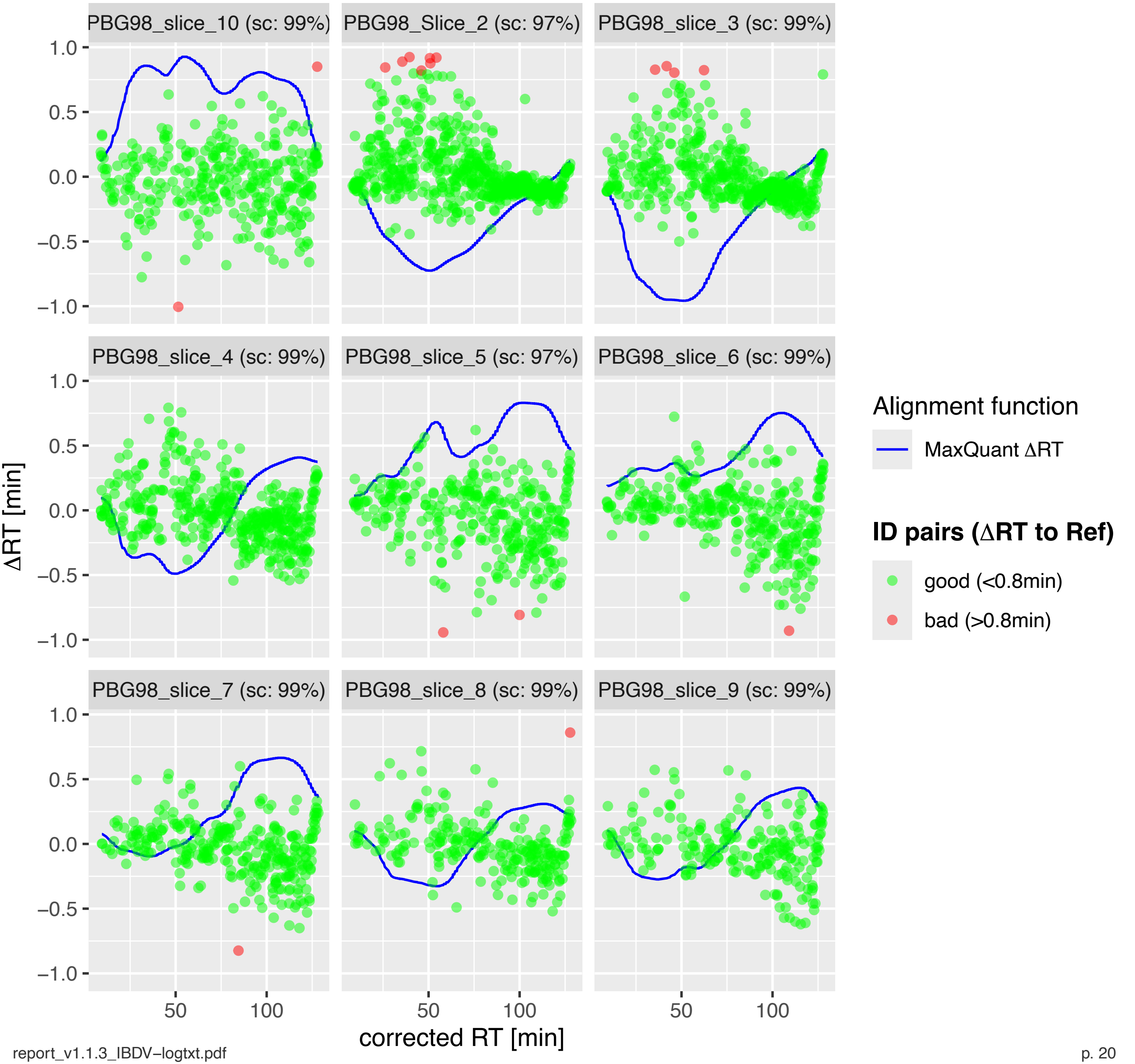

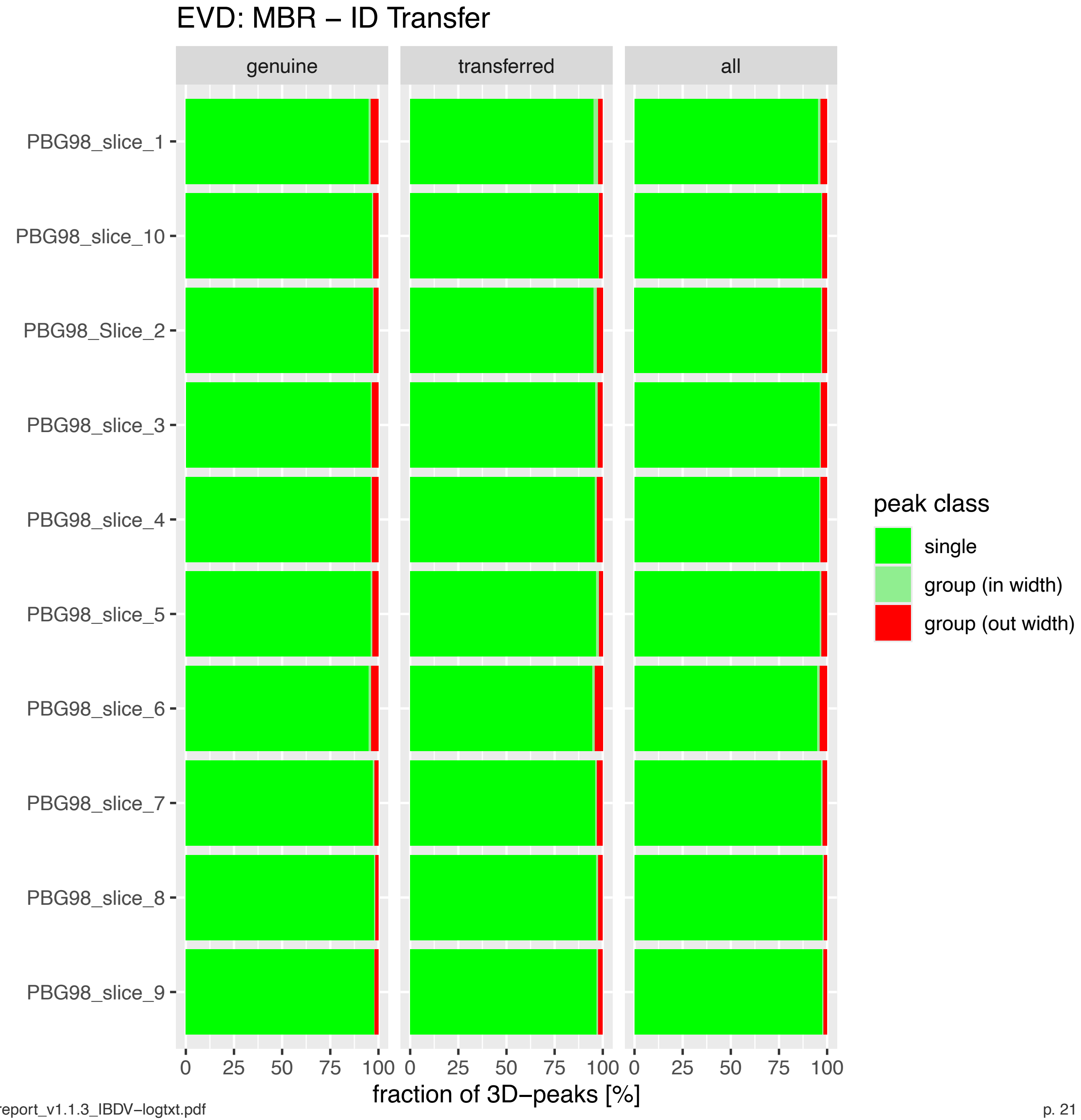
EVD: MBR – ID Transfer
genuine
transferred
all
PBG98_slice_1
PBG98_slice_10
PBG98_Slice_2
PBG98_slice_3
PBG98_slice_4
PBG98_slice_5
PBG98_slice_6
PBG98_slice_7
PBG98_slice_8
PBG98_slice_9
0 25 50 75 100
fraction of 3D−peaks [%]
peak class
single
group (in width)
group (out width)

[experimental] EVD: Clustering Tree of Raw files

by Correlation of Corrected Retention Times

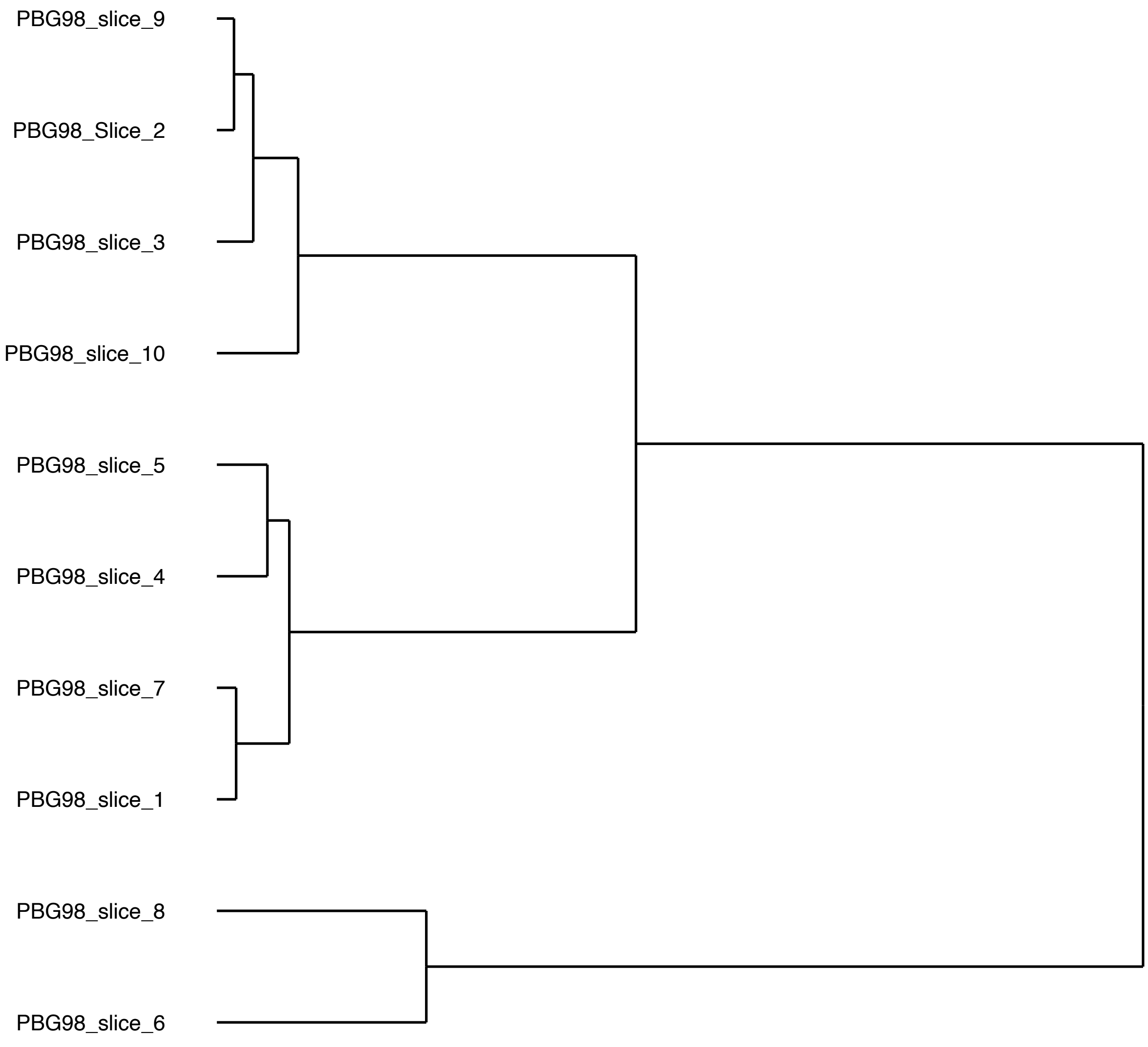

EVD: Peptides inferred by MBR

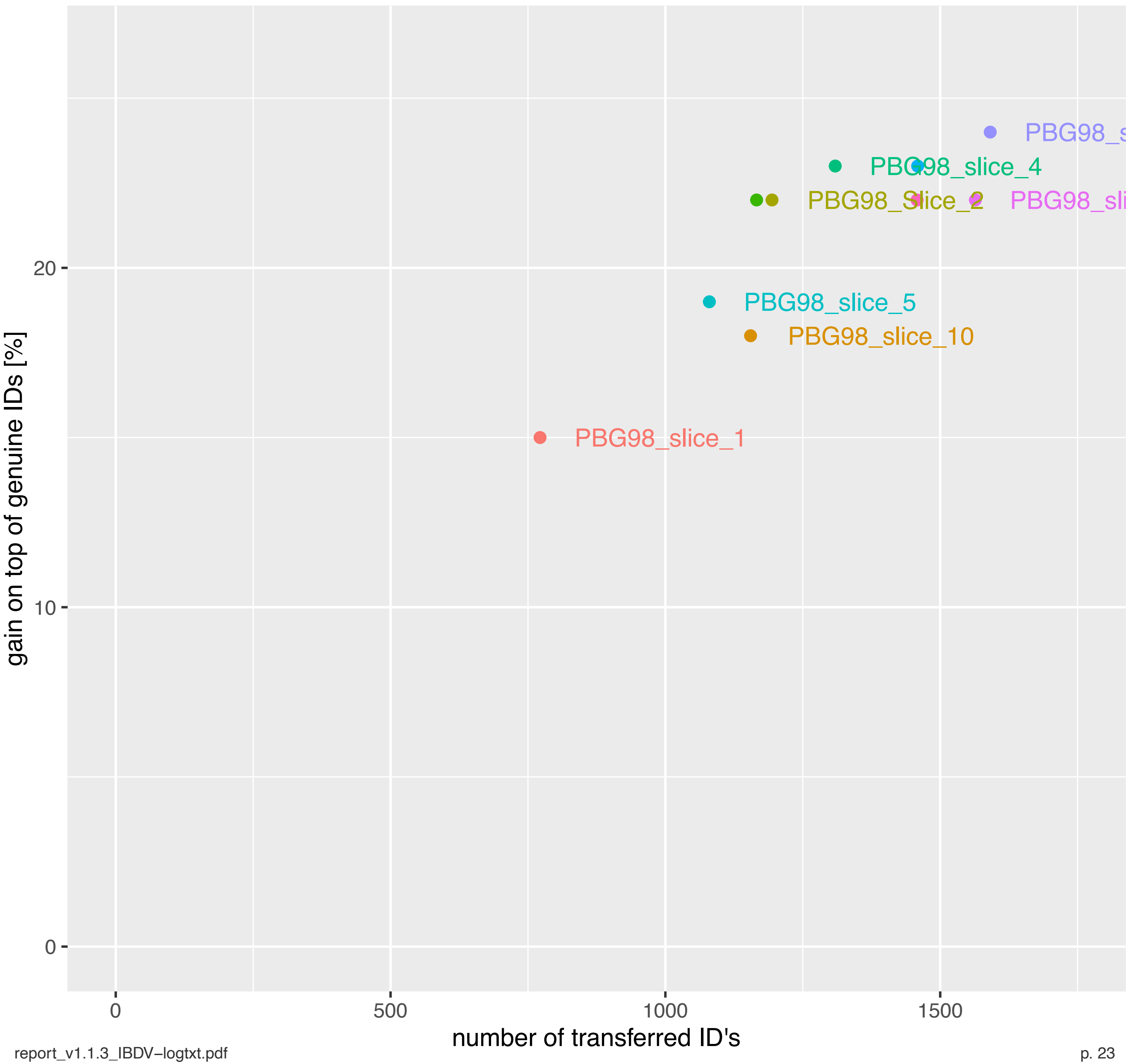

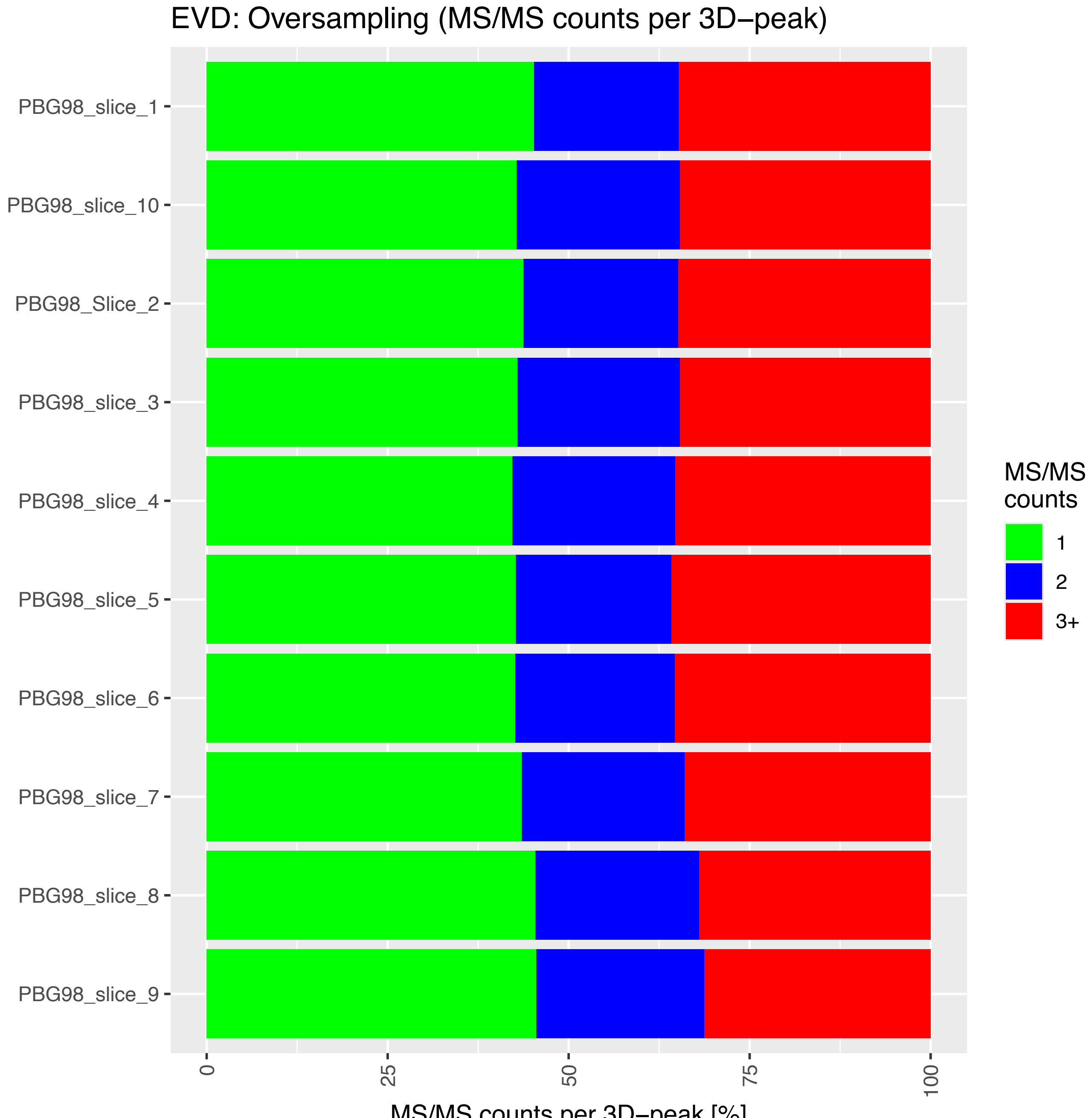
EVD: Oversampling (MS/MS counts per 3D−peak)
PBG98_slice_1
PBG98_slice_10
PBG98_Slice_2
PBG98_slice_3
PBG98_slice_4
PBG98_slice_5
PBG98_slice_6
PBG98_slice_7
PBG98_slice_8
PBG98_slice_9
0
25
50
75
100
MS/MS counts per 3D−peak [%]
MS/MS counts
1
2
3+

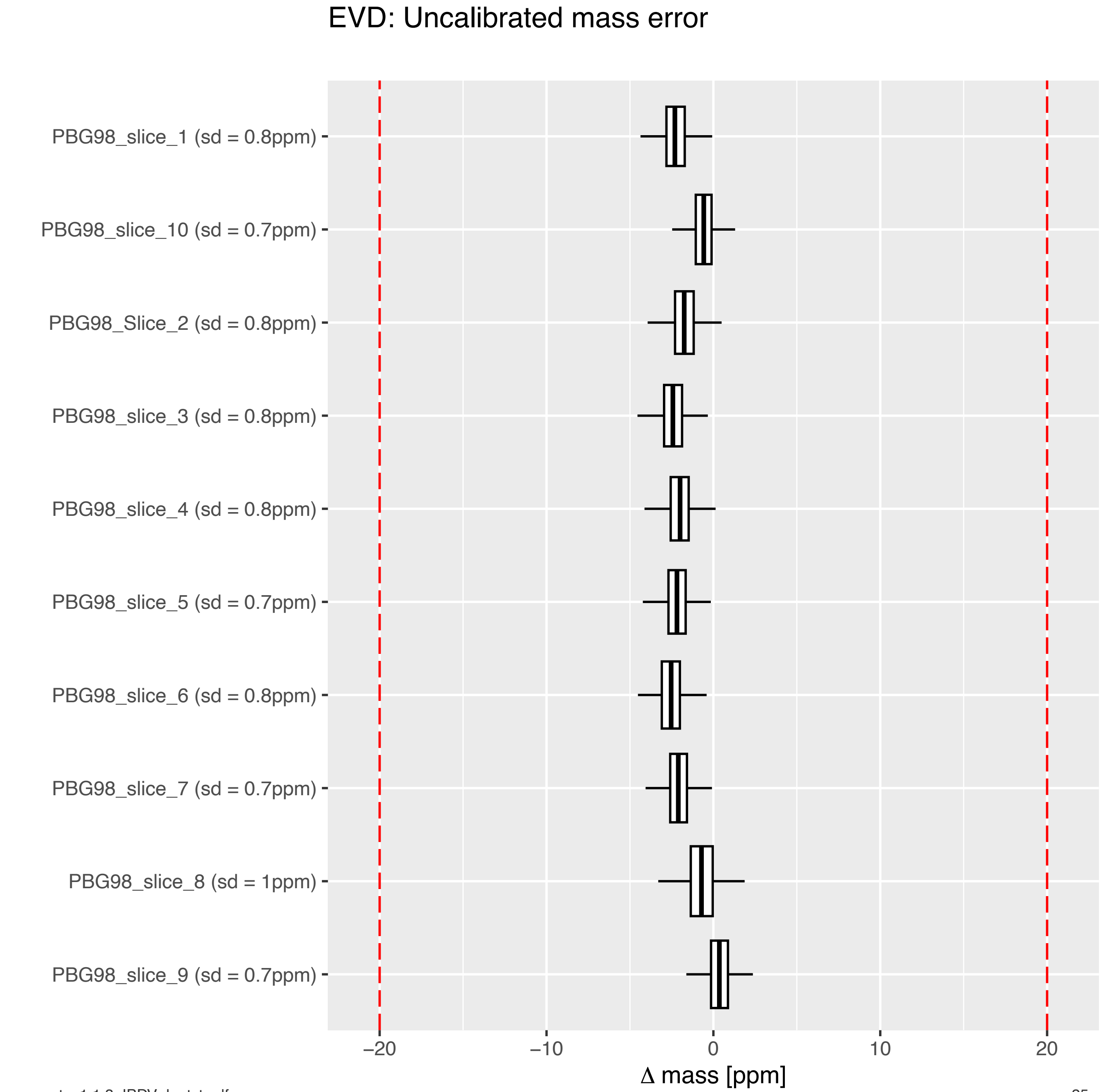
EVD: Uncalibrated mass error
PBG98_slice_1 (sd = 0.8ppm)
PBG98_slice_10 (sd = 0.7ppm)
PBG98_Slice_2 (sd = 0.8ppm)
PBG98_slice_3 (sd = 0.8ppm)
PBG98_slice_4 (sd = 0.8ppm)
PBG98_slice_5 (sd = 0.7ppm)
PBG98_slice_6 (sd = 0.8ppm)
PBG98_slice_7 (sd = 0.7ppm)
PBG98_slice_8 (sd = 1ppm)
PBG98_slice_9 (sd = 0.7ppm)
−20
−10
0
10
20
Δ mass [ppm]

EVD: Calibrated mass error

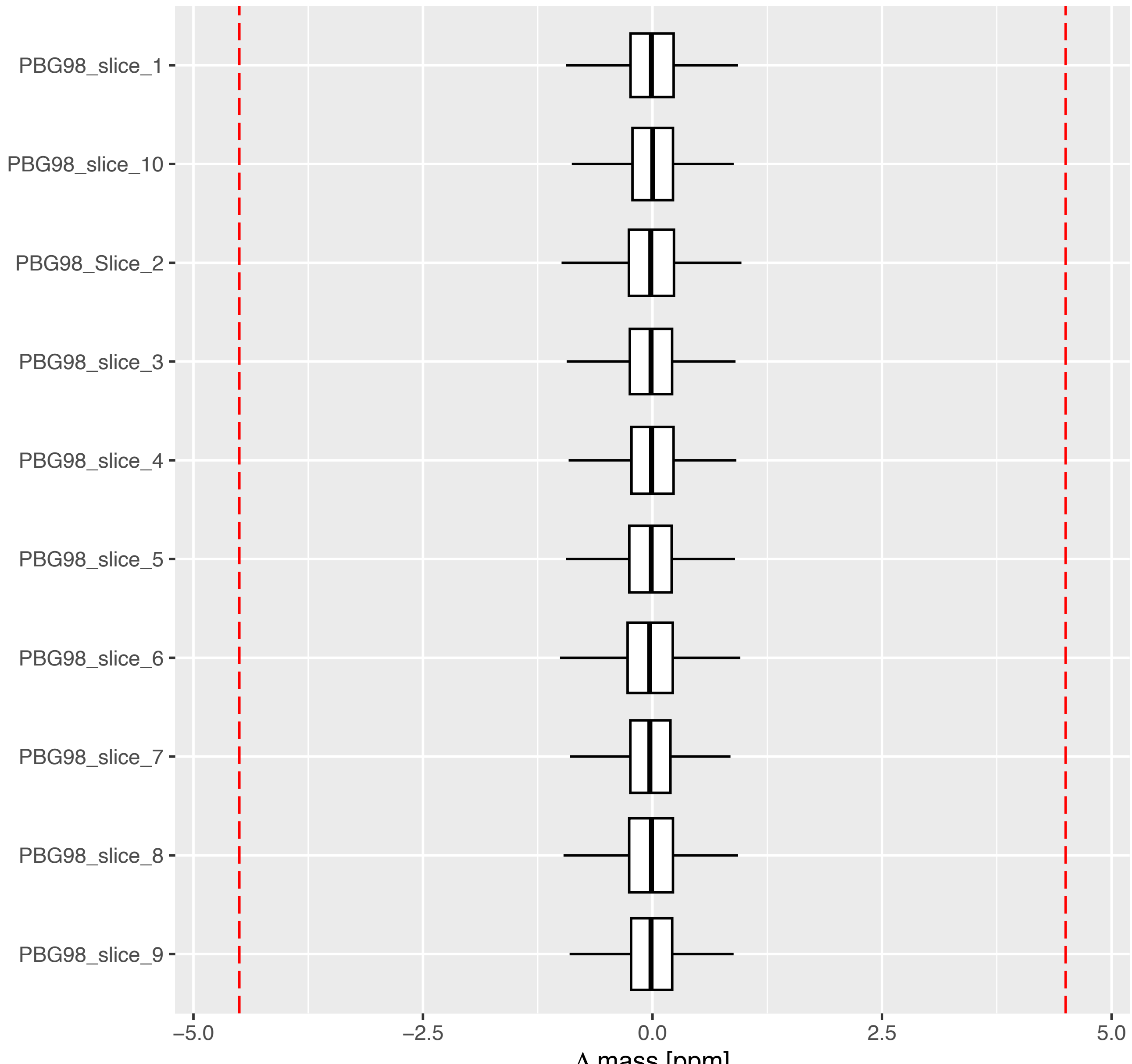

MSMS: Fragment mass errors per Raw file

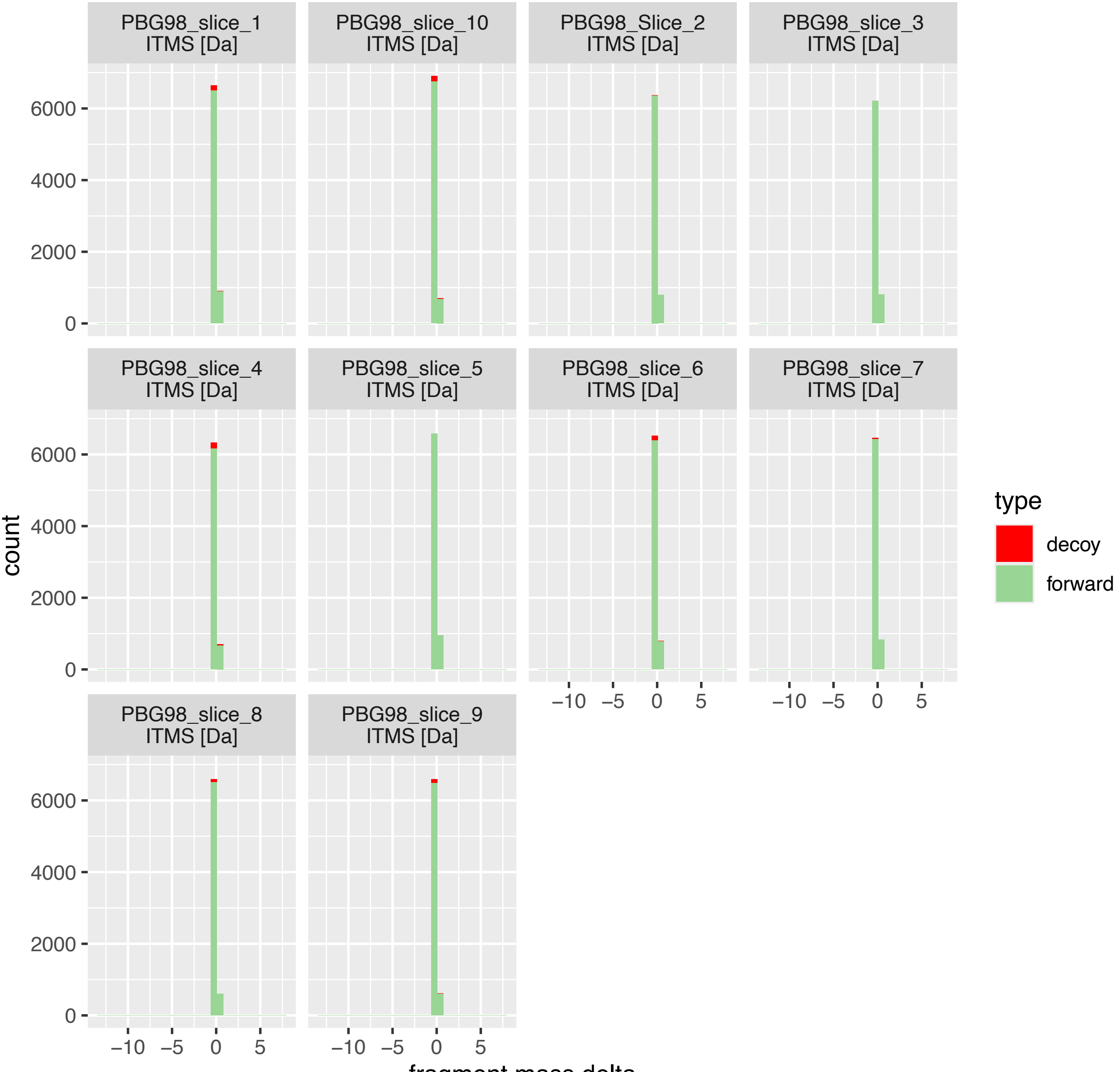

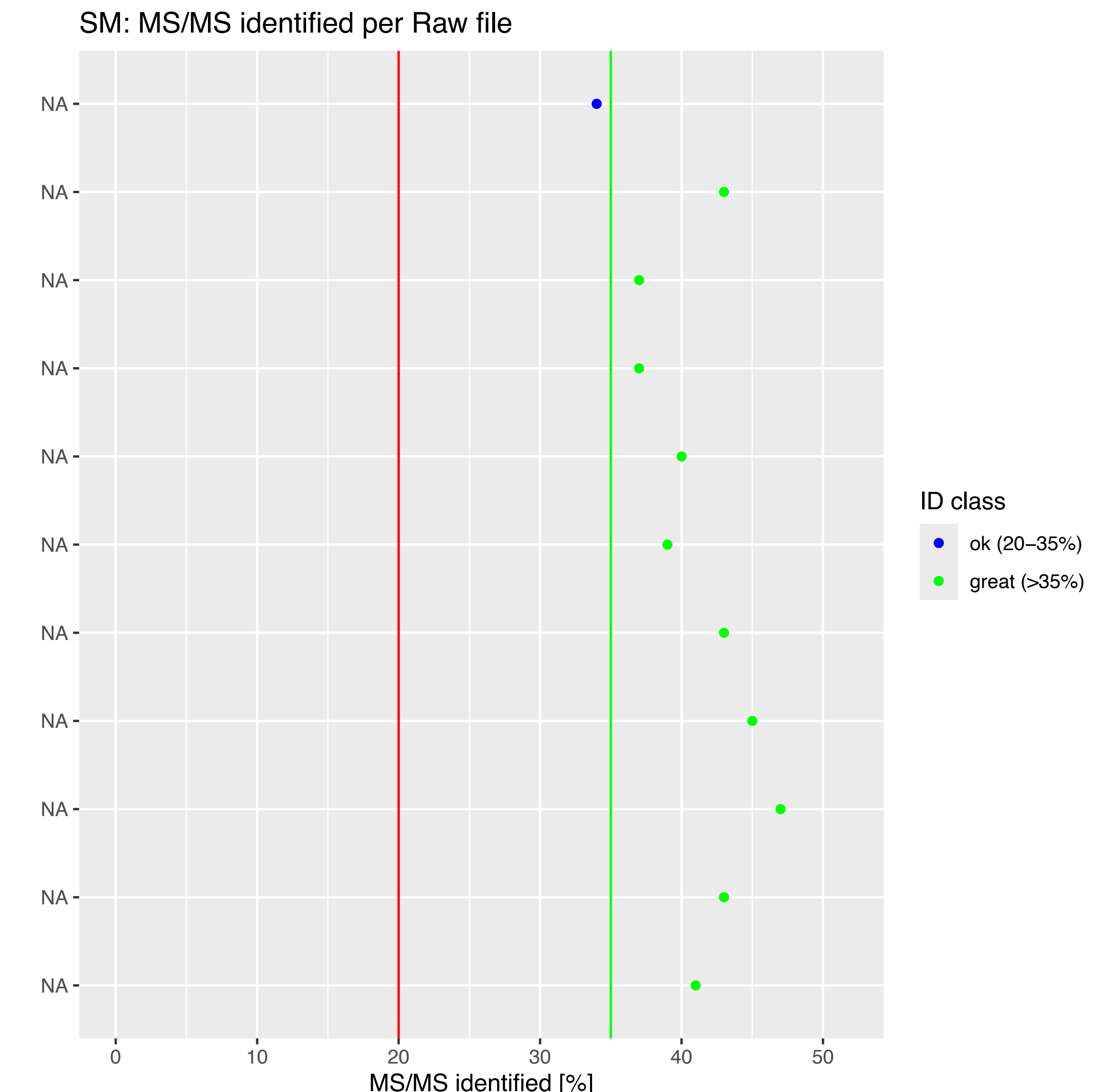
SM: MS/MS identified per Raw file
NA
NA
NA
NA
NA
NA
NA
NA
NA
NA
NA
0
10
20
30
40
50
MS/MS identified [%]
ID class
ok (20–35%)
great (>35%)

[experimental] EVD: Non−Missing Peptides

compared to all peptides seen in experiment

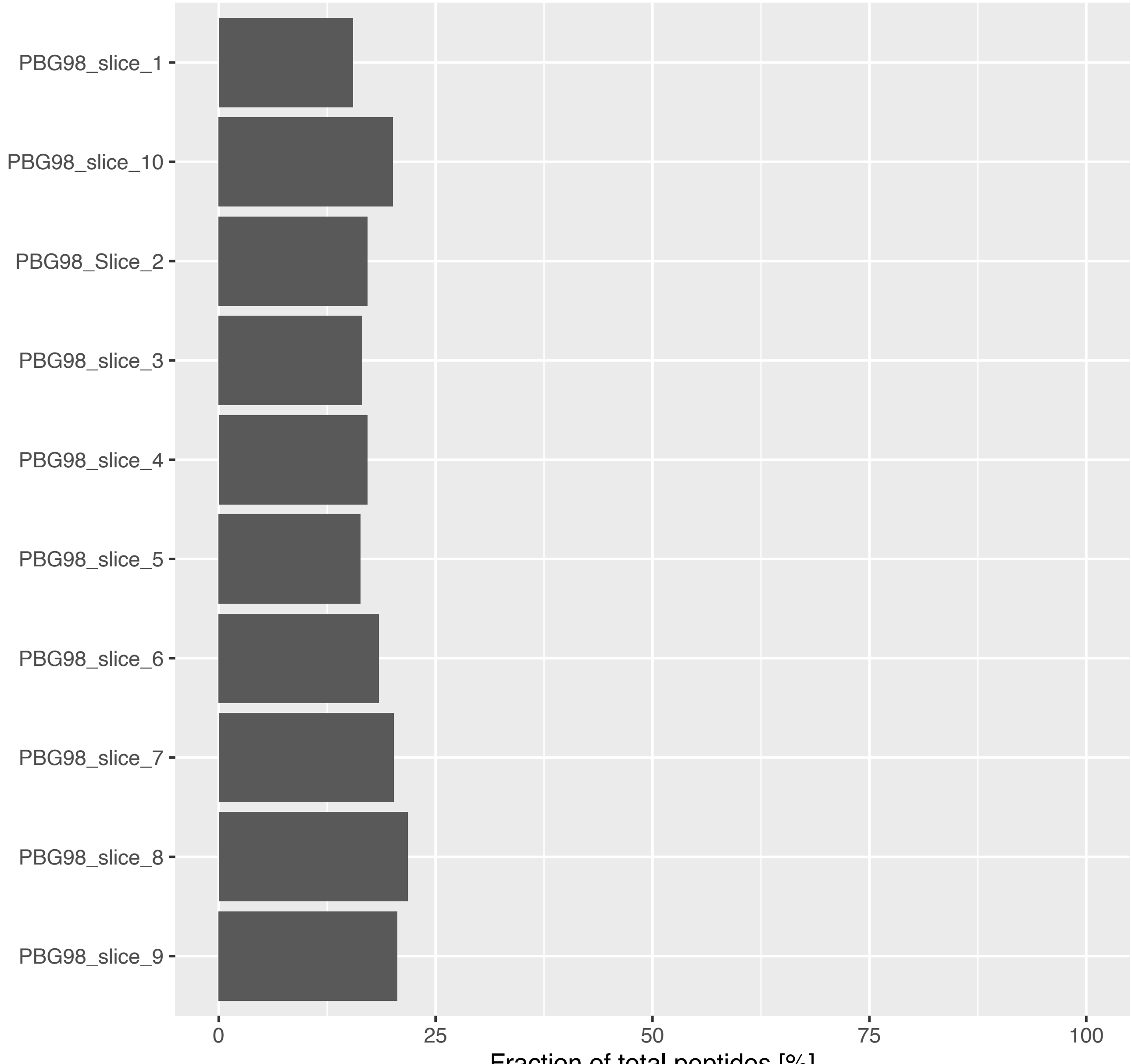

[experimental] EVD: Non−missing by set

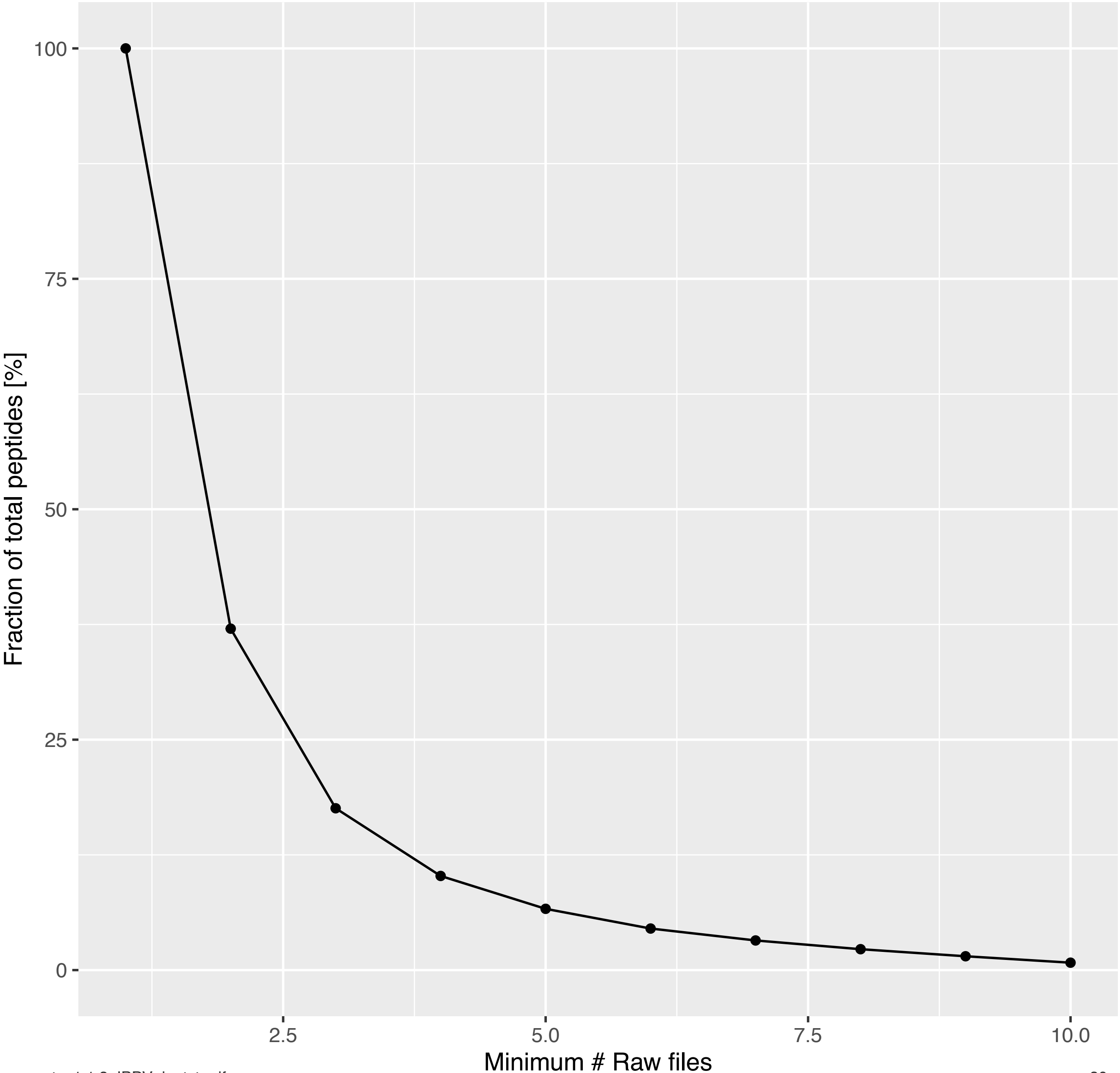

[experimental] EVD: Imputed Peptide Intensity Distribution of Missing Values

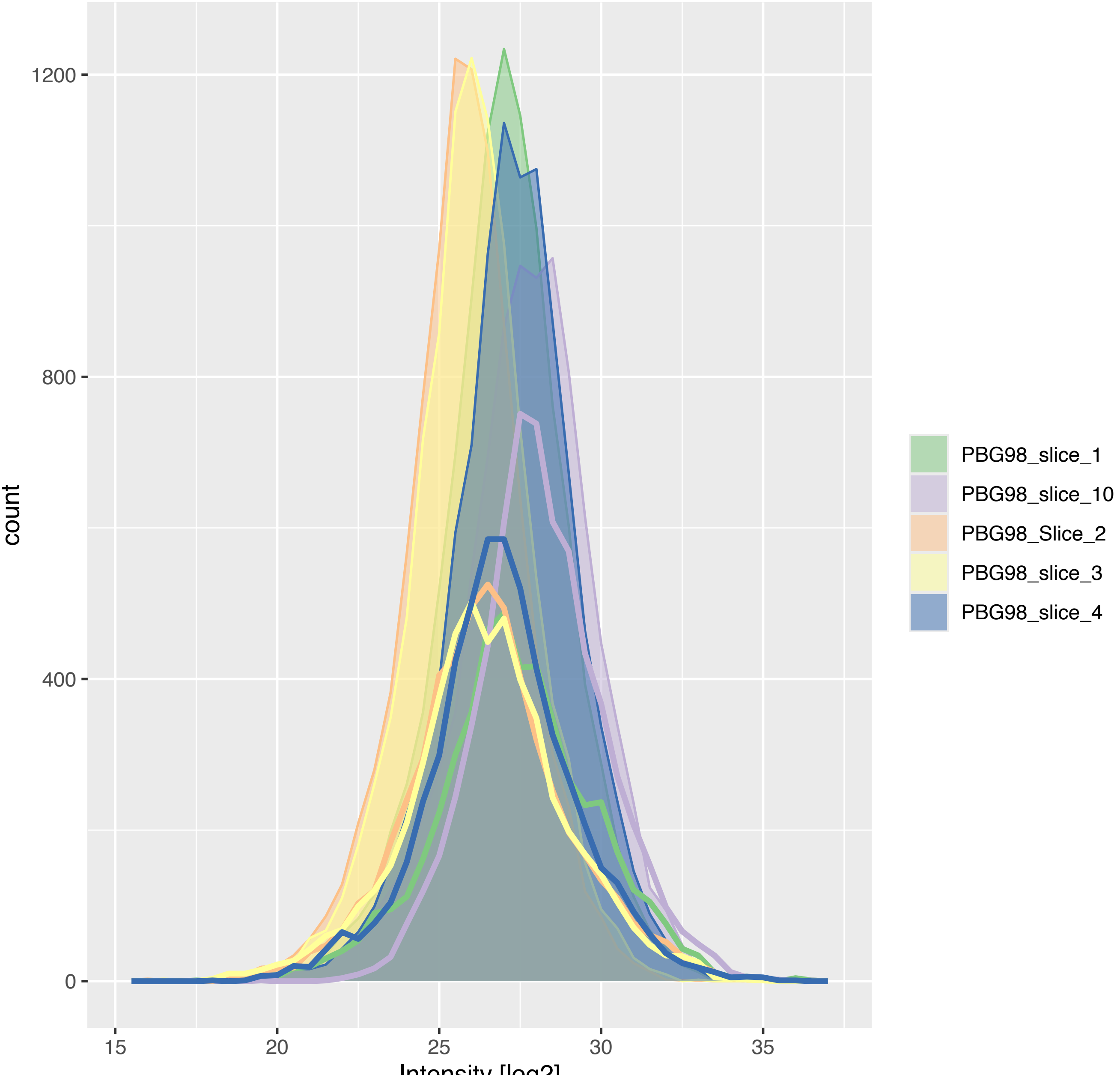

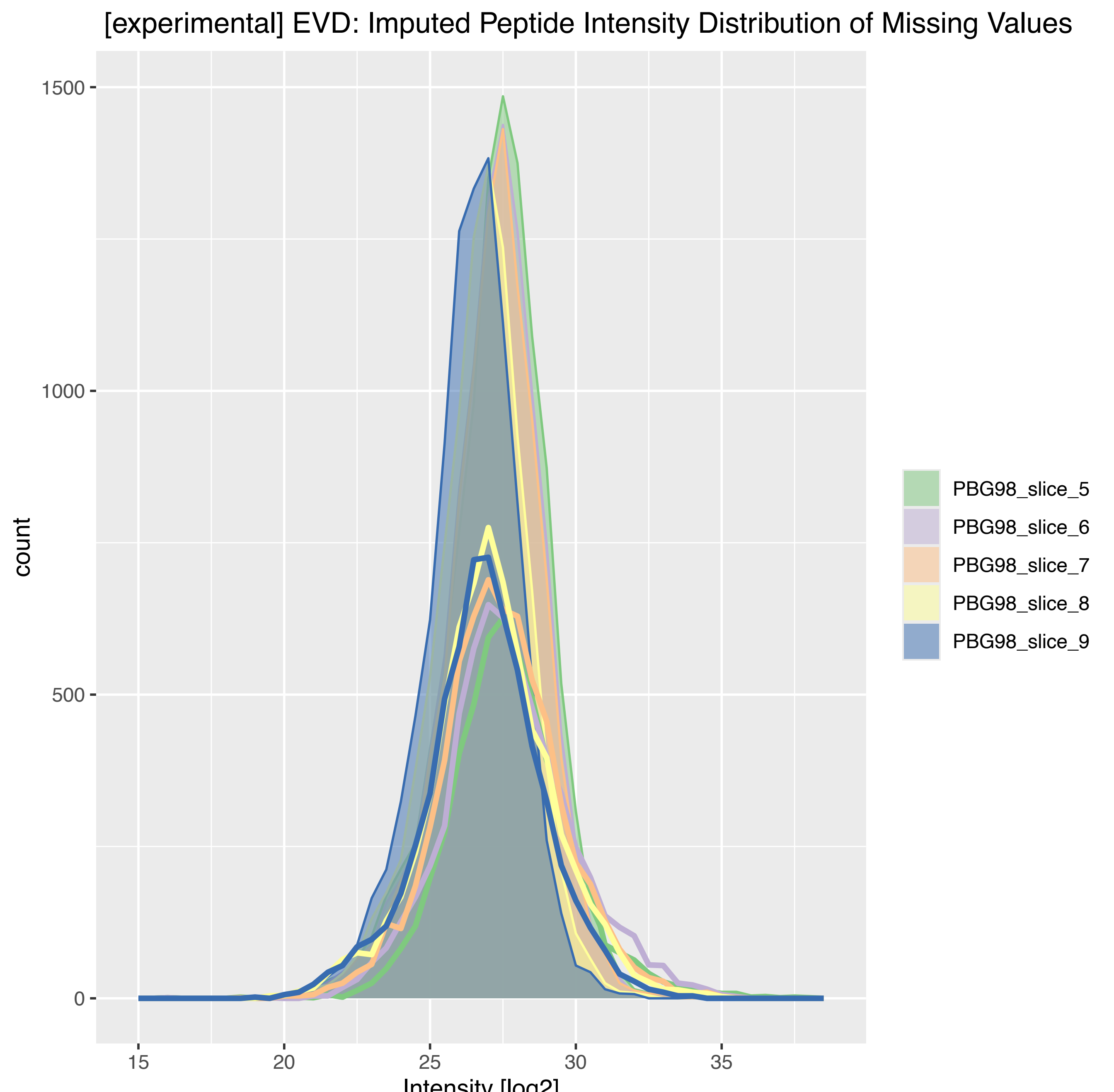

[experimental] EVD: Imputed Peptide Intensity Distribution of Missing Values
count
1500
1000
500
0
15
20
25
30
35
Intensity [log2]
PBG98_slice_5
PBG98_slice_6
PBG98_slice_7
PBG98_slice_8
PBG98_slice_9

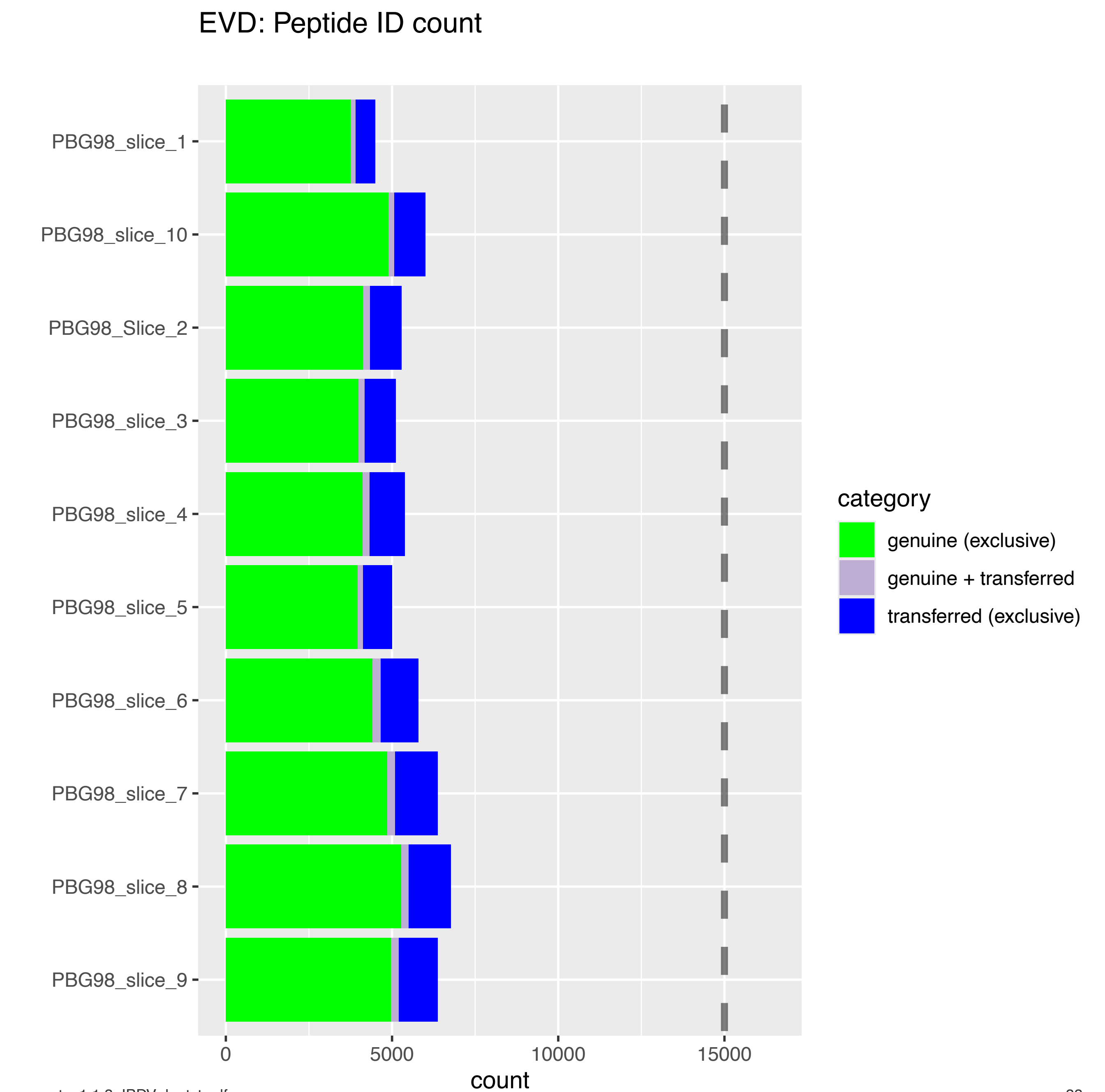
EVD: Peptide ID count
PBG98_slice_1
PBG98_slice_10
PBG98_Slice_2
PBG98_slice_3
PBG98_slice_4
PBG98_slice_5
PBG98_slice_6
PBG98_slice_7
PBG98_slice_8
PBG98_slice_9
0
5000
10000
15000
count
category
genuine (exclusive)
genuine + transferred
transferred (exclusive)

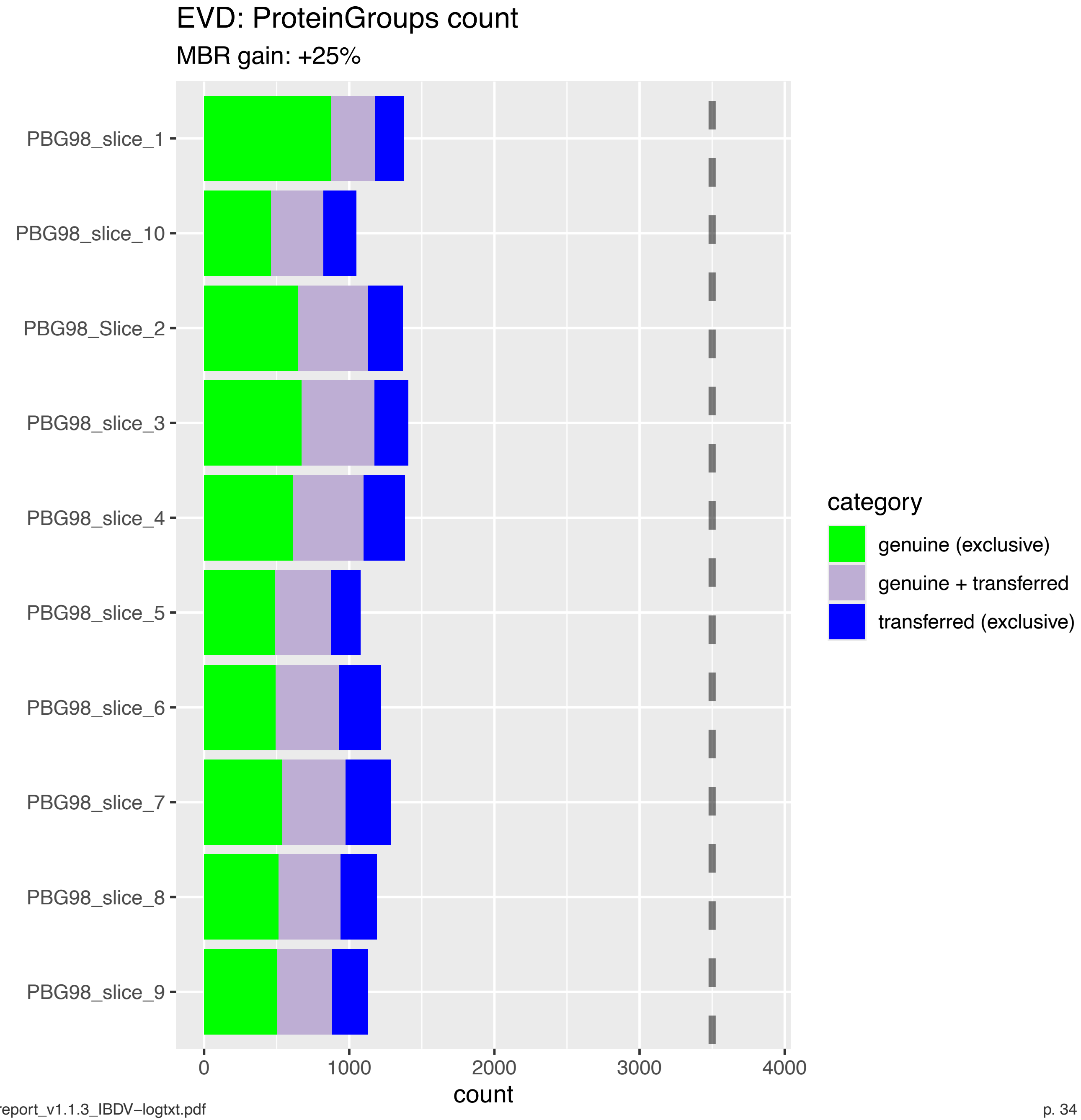

EVD: ProteinGroups count
MBR gain: +25%
PBG98_slice_1
PBG98_slice_10
PBG98_Slice_2
PBG98_slice_3
PBG98_slice_4
PBG98_slice_5
PBG98_slice_6
PBG98_slice_7
PBG98_slice_8
PBG98_slice_9
0
1000
2000
3000
4000
count
category
genuine (exclusive)
genuine + transferred
transferred (exclusive)

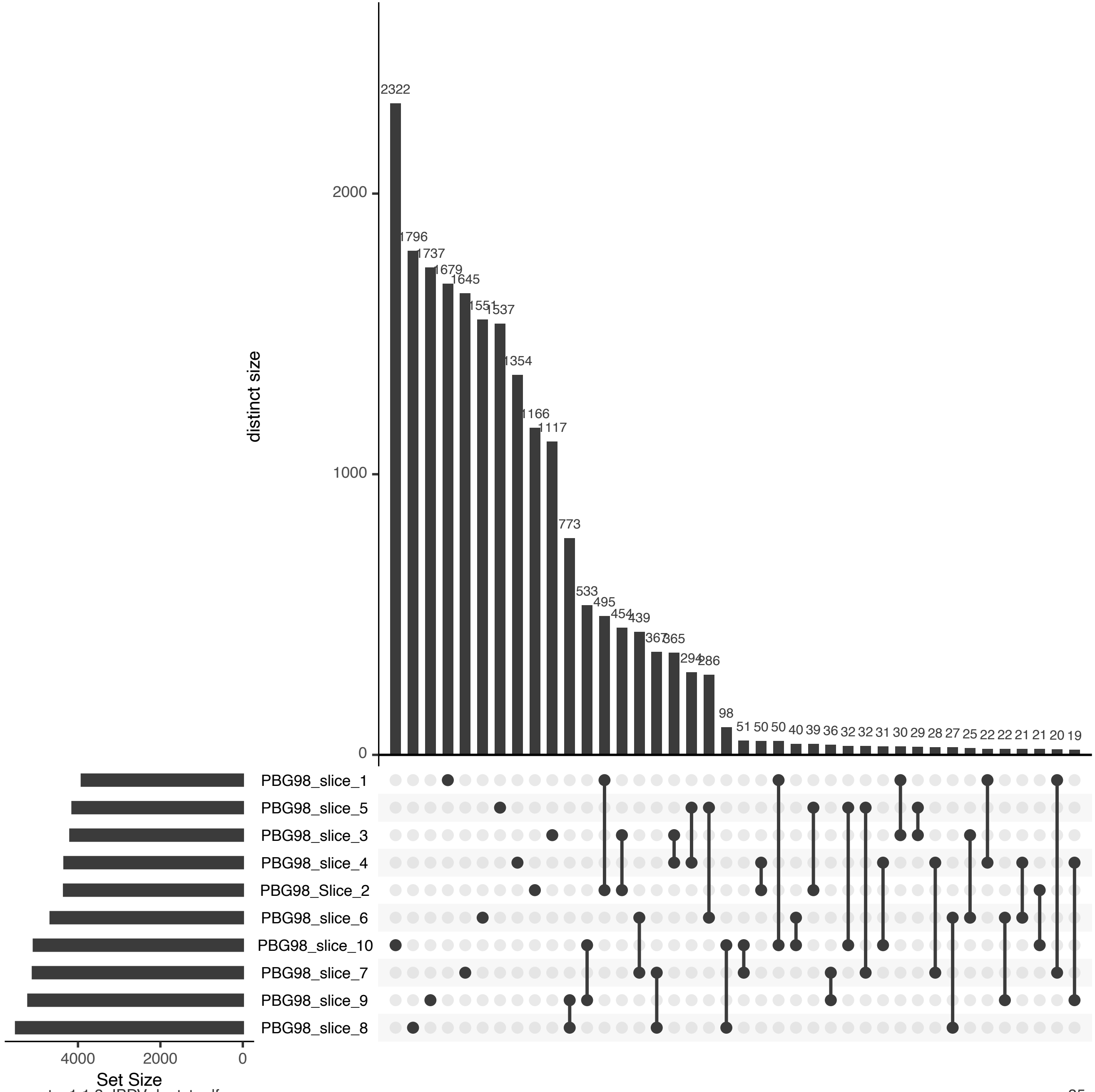
distinct size
Set Size
PBG98_slice_1
PBG98_slice_5
PBG98_slice_3
PBG98_slice_4
PBG98_Slice_2
PBG98_slice_6
PBG98_slice_10
PBG98_slice_7
PBG98_slice_9
PBG98_slice_8
2322
1796
1737
1679
1645
1551
1537
1354
1166
1117
773
533
495
454
439
367
365
294
286
98
51 50 50 40 39 36 32 32 31 30 29 28 27 25 22 22 21 21 20 19
